# Low-basicity 5-HT_7_ Receptor Agonists Synthesized Using the van Leusen Multicomponent Protocol

**DOI:** 10.1038/s41598-017-00822-4

**Published:** 2017-05-04

**Authors:** Adam S. Hogendorf, Agata Hogendorf, Rafał Kurczab, Grzegorz Satała, Tomasz Lenda, Maria Walczak, Gniewomir Latacz, Jadwiga Handzlik, Katarzyna Kieć-Kononowicz, Joanna M. Wierońska, Monika Woźniak, Paulina Cieślik, Ryszard Bugno, Jakub Staroń, Andrzej J. Bojarski

**Affiliations:** 1 Institute of Pharmacology, Polish Academy of Sciences, 12 Smętna Street, 31-343 Kraków, Poland; 2 Faculty of Pharmacy, Jagiellonian University Medical College, 9 Medyczna Street, 30-688 Kraków, Poland

## Abstract

A series of 5-aryl-1-alkylimidazole derivatives was synthesized using the van Leusen multicomponent reaction. The chemotype is the first example of low-basicity scaffolds exhibiting high affinity for 5-HT_7_ receptor together with agonist function. The chosen lead compounds 3-(1-ethyl-1*H*-imidazol-5-yl)-5-iodo-1*H*-indole (AGH-107, **1o**, *K*_i 5-HT7_ = 6 nM, EC_50_ = 19 nM, 176-fold selectivity over 5-HT_1A_R) and **1e** (5-methoxy analogue, *K*_i 5-HT7_ = 30 nM, EC_50_ = 60 nM) exhibited high selectivity over related CNS targets, high metabolic stability and low toxicity in HEK-293 and HepG2 cell cultures. A rapid absorption to the blood, high blood-brain barrier permeation and a very high peak concentration in the brain (C_max_ = 2723 ng/g) were found for **1o** after *i*.*p*. (5 mg/kg) administration in mice. The compound was found active in novel object recognition test in mice, at 0.5, 1 and 5 mg/kg. Docking to 5-HT_7_R homology models indicated a plausible binding mode which explain the unusually high selectivity over the related CNS targets. Halogen bond formation between the most potent derivatives and the receptor is consistent with both the docking results and SAR. 5-Chlorine, bromine and iodine substitution resulted in a 13, 27 and 89-fold increase in binding affinities, respectively, and in enhanced 5-HT_1A_R selectivity.

## Introduction

Within the serotonergic system, 5-HT_7_, the last identified serotonin receptor, is one of the most valuable drug targets^1^. Discovered independently by three laboratories in 1993 ^2–4^, 5-HT_7_R was thought to function by being coupled to G_s_ to stimulate intracellular production of cAMP, but it was later found to also be coupled to G_12_ which is an alternative signal transduction pathway^5^. 5-HT_7_R is expressed in the central nervous system (thalamus, hypothalamus, hippocampus and cortex) as well as peripherally (pancreas, spleen, coronary artery, ileum and intestine)^3–6^. Experiments using animal models have shown that the receptor is involved in many physiological processes, i.e. the regulation of body temperature^7^, smooth muscle relaxation of cerebral arteries^8^, circadian rhythm, learning and memory^9–13^, as well as pathophysiological processes such as mood disorders, anxiety^14^, inflammatory processes in the CNS^15^, schizophrenia^16^ and pain^17, 18^. 5-HT_7_R antagonists have been proposed as potential drugs targeting depression^19, 20^. There have been less conclusive reports on potential anxiolytic^21, 22^, analgesic^17^ and antipsychotic properties of the receptor ligands^23, 24^. It was established that 5-HT_7_R blockade can induce promnesic effects indicating the possibility of developing atypical procognitive antidepressants^25^.

There are several 5-HT_7_R agonists available to serve as molecular probes. These include small, low-weight molecules, e.g. AS-19^26^, RA-7^7^, 5-carboxyamidotryptamine (5-CT), 5-methoxytryptamine, 8-OH-DPAT^27^, and lysergic acid derivatives^28^ and long-chain diphenylpiperazines (LP-12, LP-44, LP-211) developed by Leopoldo *et al*.^29–31^. None of these compounds qualify as perfect radioligand candidates, mainly due to their poor selectivity. 5-HT_7_R agonists often bind to 5-HT_1A_R and 5-HT_5A_R^32^. Some promising 5-HT_7_R molecular probes suffer from poor metabolic stability^33^. In the case of larger molecules, membrane penetration may be an issue as it is considerably harder to find an optimal mass-lipophilicity ratio. Animal models suggest several possible indications of 5-HT_7_R agonists as therapeutics. The stimulation of 5-HT_7_R in adolescent rats by LP-211 causes plastic rearrangements within the forebrain networks, accounting for long-lasting behavioural changes in adulthood^34^. The activation of 5-HT_7_R was found to be beneficial in mouse models of Rett syndrome^35^ and fragile X syndrome^36^. Compound AS-19, which was the first selective agonist of the 5-HT_7_R, as well as LP-12, LP-44, LP-211 and E-55888 have been used in numerous *in vivo* experiments which led to the characterization of the receptor. It was found, that 5-HT_7_ agonists can enhance long-term memory formation. In animal models of pain, AS-19, LP-44 and LP-211 were found antinociceptive. The antinociceptive properties of morphine can be dramatically enhanced by the co-administration of the E-55888, while the compound does not produce any analgesic effect on its own^37^.

Non-basic aminergic GPCR ligands have recently emerged as pharmacological rule breakers. Despite lacking the capability of forming a salt bridge (strong, charge assisted hydrogen bond) with Asp3.32 such chemical entities have been shown to bind to 5-HT_2A_R^38^ and later to 5-HT_6_ and 5-HT_1B_ receptors with nanomolar and subnanomolar potencies^39, 40^. Interestingly, there have been no published examples of low-basicity ligands of the 5-HT_7_ receptor^41^. A hypothesis that an aromatic basic moiety can successfully serve as the aminergic fragment of a serotonin receptor ligand was not yet tested. Till date there were numerous basic scaffolds (piperazine, piperidine, pyrrolidine, polycyclic amines, alkylamines) incorporated into serotonin receptor ligands. The imidazole fragment, which is a privileged structure commonly used in medicinal chemistry, has not been tested as a basic fragment in the aminergic GPCR field and was hardly used as a part of serotonin receptor ligands^42^.

**Figure 1:**
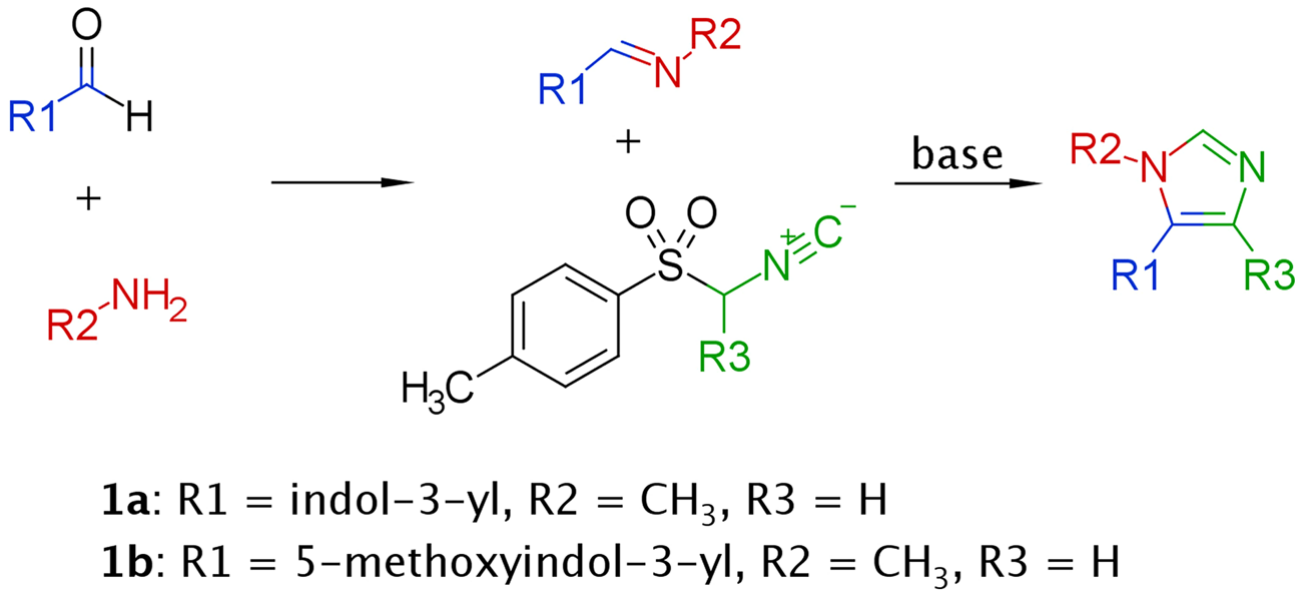
Van Leusen imidazole synthesis.

**Figure 2:**
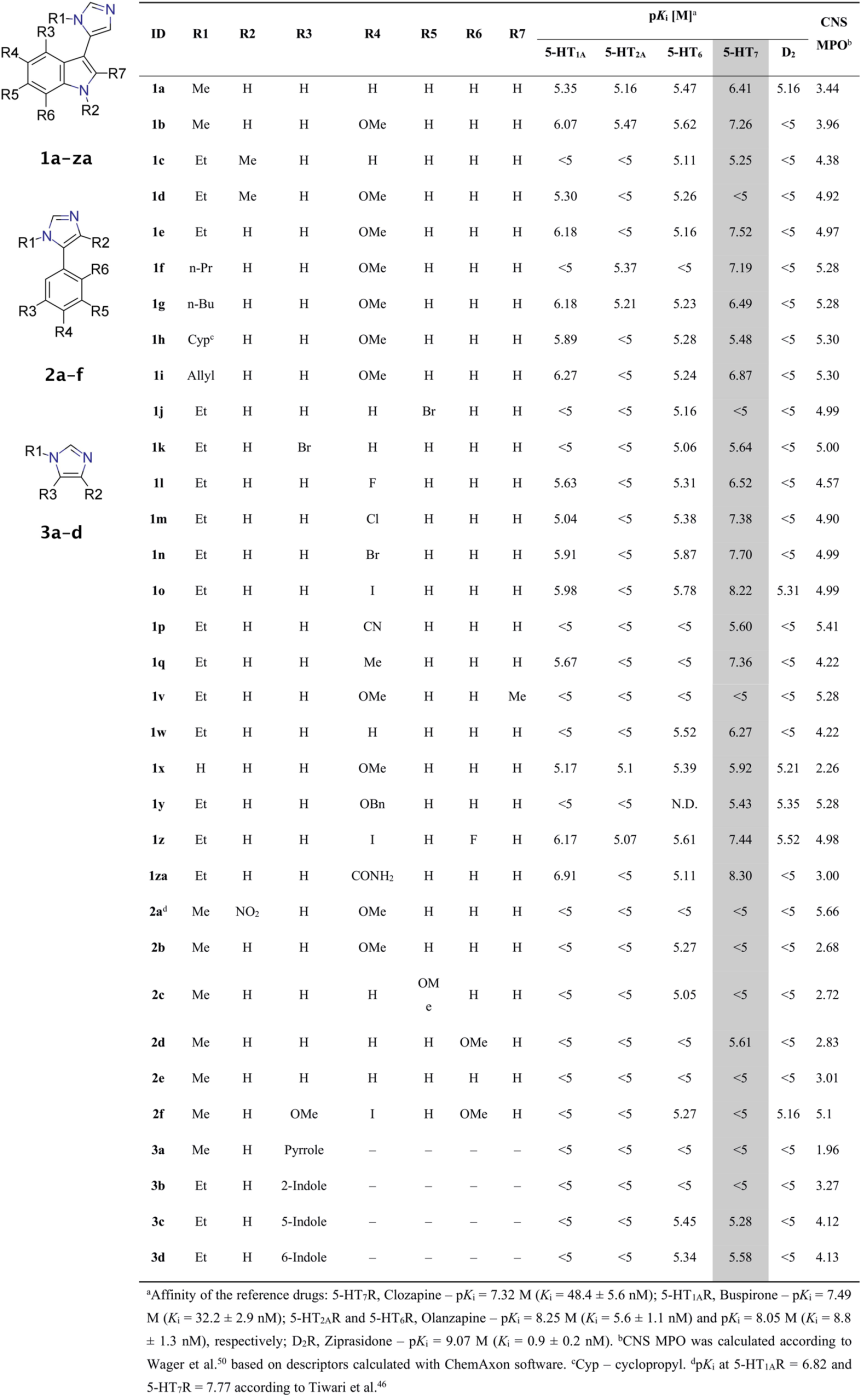
Structure, binding data and Central Nervous System Multiparameter Optimization score (CNS MPO) of compounds **1a**–**1za**, **2a**–**2f** and **3a**–**3d**.

Our initial hypothesis was that multicomponent reaction (MCR) protocols can be successfully applied to the field of GPCR ligand synthesis. We believed that the close serotonin analogues **1a** (AGH-38) and **1b** (AGH-39) (Figs 1 and 2), which can be concisely prepared via van Leusen tosylmethylisocyanide (TosMIC) imidazole synthesis^43, 44^, would exhibit activity at some serotonin receptors.

Owing to the click-chemistry properties of van Leusen imidazole synthesis, different 1-substituted, 5-arylated imidazoles were conveniently prepared and evaluated. The MCR protocol enabled the systematic SAR exploration by first optimizing the core containing carbonyl component, then optimizing the amine component for the most active entities. Over the course of the project it became clear that potent and selective, non-basic 5-HT_7_R ligands can arise via the SAR exploration of the close chemical space of arylimidazoles.

## Results and Discussion

### Chemistry

Compounds **1a**–**1q**, **1s**–**1w**, **1y**–**1za**, **2b**–**2f** and **3a**–**3d** (Fig. 2) were synthesized using a concise multicomponent protocol (Fig. 1). The van Leusen reaction proceeds via the stepwise cycloaddition of TosMIC to a polarized double bond of the preformed imine. The elimination of *p*-toluenesulfonic acid from the cyclic intermediate affords imidazole. Aromatic aldehydes were combined with appropriate amines in methanol. When the imine formation equilibrium was achieved, TosMIC and potassium carbonate were added to yield the desired products. Most of the indole-3-carboxaldehydes used were synthesized by the Vilsmeier-Haack formylation of the appropriate indoles. Derivatives **1r** and **1x** were synthesized by reductive debenzylation of **1y** and **1 s**, respectively. Compound **1za** was synthesized from **1p** by the controlled hydrolysis of the cyano group in basic conditions.

### Pharmacology

All the synthesized compounds were tested in a radioligand binding assays to determine their affinity for five receptors: 5-HT_1A_, 5-HT_2A_, 5-HT_6_, 5-HT_7_ and D_2_. The assays were performed via the displacement of the respective radioligands from cloned human receptors, all stably expressed in HEK293 cells (except for 5-HT_2A_ which was expressed in CHO cells): [^3^H]-8-OH-DPAT for 5-HT_1A_R, [^3^H]-ketanserin for 5-HT_2A_R, [^3^H]-LSD for 5-HT_6_R, [^3^H]-5-CT for 5-HT_7_R and [^3^H]-raclopride for D_2_R. Ligands with a high affinity for the 5-HT_7_ receptor were selected for a functional assay (as hydrochloride salts). The experiment was performed in HEK293 cells overexpressing 5-HT_7_R, and the ability of tested compounds to increase cAMP production was measured. The cAMP concentration was measured using TR-FRET and a cAMP specific antibody. Lead compounds **1e** (AGH-44) and **1o** (AGH-107) were screened toward several CNS targets (Eurofins Cerep, Le Bois l’Eveque, 86600 Celle L’Ev-escault, France, www.cerep.fr): α_1_, α_2C_, CB_1_, H_1_, D_3_, 5-HT_1B_, 5-HT_2B_ and 5-HT_5A_. The intrinsic clearance of **1e** and **1o** were determined using human liver microsomes according to Obach *et al*.^45^ (see Supporting Information for detailed results). The HEK-293 and HepG2 cell lines viabilities were tested after 72 h of incubation with tested compounds. Doxorubicin (DX), and carbonyl cyanide 3-chlorophenylhydrazone (CCCP) were used as internal standards (see Supporting Information). The pharmacokinetics of compound **1o** was tested on CD-1 mice after intraperitoneal injection at 5 mg/kg dose (see Supporting Information). The compound **1o** was administered at 0.5, 1 and 5 mg/kg to investigate the ability of the compound to reverse MK-801 induced impairment in novel object recognition test. The experiment was conducted on Albino Swiss mice (see Supporting Information).

### Structure-Activity Relationship

After acquiring highly encouraging initial results for compounds **1a** and **1b** (*K*_i 5-HT7_ = 385 nM and 50 nM, respectively, selectivity over 5-HT_1A_R > 11-fold, Fig. 2) a literature search made us realize that an arylimidazole 5-HT_7_R ligand was recently described, although no SAR studies were reported^46^. The compound, 5-(4-([^11^C]methoxyphenyl)-1-methyl-4-nitro-1*H*-imidazole, **2a**, has been developed as a PET radioligand. The high and rapid uptake of **2a** to the rat brain after injection was reported which would suggest at least decent ADME properties. The finding further encouraged us to explore the series, so **2a** was resynthesized to serve as a reference compound. However, we were not able to reproduce the results presented by Tiwari *et al*.^46^. In our radioligand displacement binding assays compound **2a** did not bind to 5-HT_7_R and 5-HT_1A_R (binding curves of compound **2a** can be found in Supporting Information). Moreover, a very close analogue of **2a** (the only structural difference being the lack of the nitro group) – **2b** – was also found to be completely inactive. A compound based on the 2,5-dimethoxy-4-iodophenyl core, which is a scaffold common to many 5-HT_2_R ligands^47, 48^, **2f** was expected to bind to 5-HT_2A_R, but this assumption was not validated. Compounds synthesized from unsubstituted and differently methoxy-substituted benzaldehydes (entries **2c**–**2e**) were also not active. Compounds **3a**–**3d** were synthesized to answer the question of whether methylimidazoles coupled to different aromatic cores (3-pyrrole, 2-indole, 5-indole, or 6-indole) could show any 5-HT_7_R activity. This group of compounds did not bind to 5-HT_7_R at all, even **3a** which is a close, simplified analogue of **1a** did not possess any activity. It was thus evident that indole-3-imidazole (the core structure of series **1**) is the molecular scaffold of choice.

The role of the indole nitrogen atom in the binding of the active compounds to 5-HT_7_R was determined by the synthesis of two derivatives. Entry **1c** (*K*_i 5-HT7_ = 5623 nM) which is a methylated analogue of **1a**, and **1d** (*K*_i 5-HT7_ > 10000 nM) which is a methylated counterpart of **1b** clearly showed that the unsubstituted pyrrole fragment plays an important role in receptor binding. Homologues **1b**, and **1e**–**1 g** and compounds **1 h**, and **1i** were synthesized to optimize the length and shape of the amine component. Apparently, the ethyl substituted derivative exhibited optimal binding affinity (**1e**, *K*_i 5-HT7_ = 30 nM). Interestingly, **1 h** (R1 = cyclopropyl) and **1 s** (R1 = benzyl) were found to be non-active which would suggest a very narrow space in the binding pocket reserved for an alkyl fragment. The modification of the indole containing component resulted in compounds **1j**–**1 v**. Forbidden positions 2-, 6-, and 7- (R7, R5, and R6) can be clearly observed in entries **1k**, **1 u**, and **1 v**, respectively. Compound **1t** exhibited moderate activity toward 5-HT_7_R (*K*_i_ = 122 nM) indicating that appropriate substituents are acceptable at the fourth position of the indole nucleus. Although **1j** and **1k** (4-bromo- and 6-bromoindole cores, respectively) were found to possess low affinity for 5-HT_7_R, **1n** (5-bromo) was a hit compound with *K*_i_ = 20 nM. Successively, a series of 5-substituted indole-containing derivatives were synthesized. The activity of compounds **1l**–**1q** and **1w** showed a very probable involvement of halogen bonding in the binding of the most active derivatives **1m**–**1o**. 5-HT_7_R affinities of methyl and fluoro derivatives **1q** and **1 l** (44 nM and 302 nM, respectively) were considerably lower than for the halogen series (42 nM, 20 nM, and 6 nM for Cl, Br and I, respectively), indicating the hydrophobicity to be of secondary importance. Compound **1o** (5-iodo) became the lead in the series exhibiting *K*_i_ = 6 nM at the human 5-HT_7_ receptor, and 176-fold selectivity over 5-HT_1A_R. A very close serotonin analogue **1r** bound to 5-HT_7_R with *K*_i_ = 39 nM, whereas an analogue of 5-CT, **1za** displayed *K*_i_ = 5 nM.

### *In vitro* pharmacology

Functional assay results for the chosen compounds are shown in Table 1. Compounds **1b**, **1e**, **1m**, **1n**, **1o**, **1q** and **1r** acted as full agonists at the human 5-HT_7_ receptor. The strongest agonist function was observed for **1o** (5-iodo) and **1r** (5-hydroxy).

**Table 1:** Results of functional activity for 5-HT_7_R, CYP3A4 inhibition assay and metabolic stability for the selected compounds.

Compound ID	R1	R4	*K*_i_	5-HT_7_REC_50_ [nM]	CYP3A4 IC_50_ [μM]	Cl_int_^a^ [ml/min/kg]
**1b**	Me	OMe	55	150	—	—
**1e**	Et	OMe	30	60	0.24	3.69
**1m**	Et	Cl	42	149	—	—
**1n**	Et	Br	20	154	—	—
**1o**	Et	I	6	19	0.17	6.3
**1q**	Et	Me	44	45	—	—
**1r**	Et	OH	39	22	—	—

^a^Metabolic clearance (human liver microsomes).

The receptor profile of the tested compounds shows high selectivity over the tested CNS targets. All compounds from chemotype **1** showed *K*_i_ > 1000 nM at the 5-HT_2A_, 5-HT_6_ and D_2_ receptors. **1e** and **1o** were found to be selective toward a set of CNS targets (Table 2). Within the extended receptor panel tested, **1e** and **1o** showed affinity only for 5-HT_2B_ (% of inhibition at 1 µM = 93 and 87, respectively), but at a 10 nM concentration, the level of receptor binding was very low (6 and 7%, respectively). Additionally, compounds **1e** and **1o** showed very promising metabolic stability with metabolic clearance 3.69 and 6.3 ml/min/kg, respectively.

**Table 2:** Anti-target profiles of selected compounds **1e** and **1o**.

Compound	Concentra-tion [M]	% Inhibition of control binding^a^							
		α_1_	α_2C_	CB_1_	D_3_	H_1_	5-HT_1B_	5-HT_2B_	5-HT_5A_
**1e**	10^−6^	5	9	8	8	6	24	93	9
	10^−8^	—	—	—	—	—	—	6	—
**1o**	10^−6^	—	—	—	—	—	40	87	7
	10^−8^	—	—	—	—	—	—	7	—

^a^Experiments performed at Eurofins Cerep.

**Figure 3:**
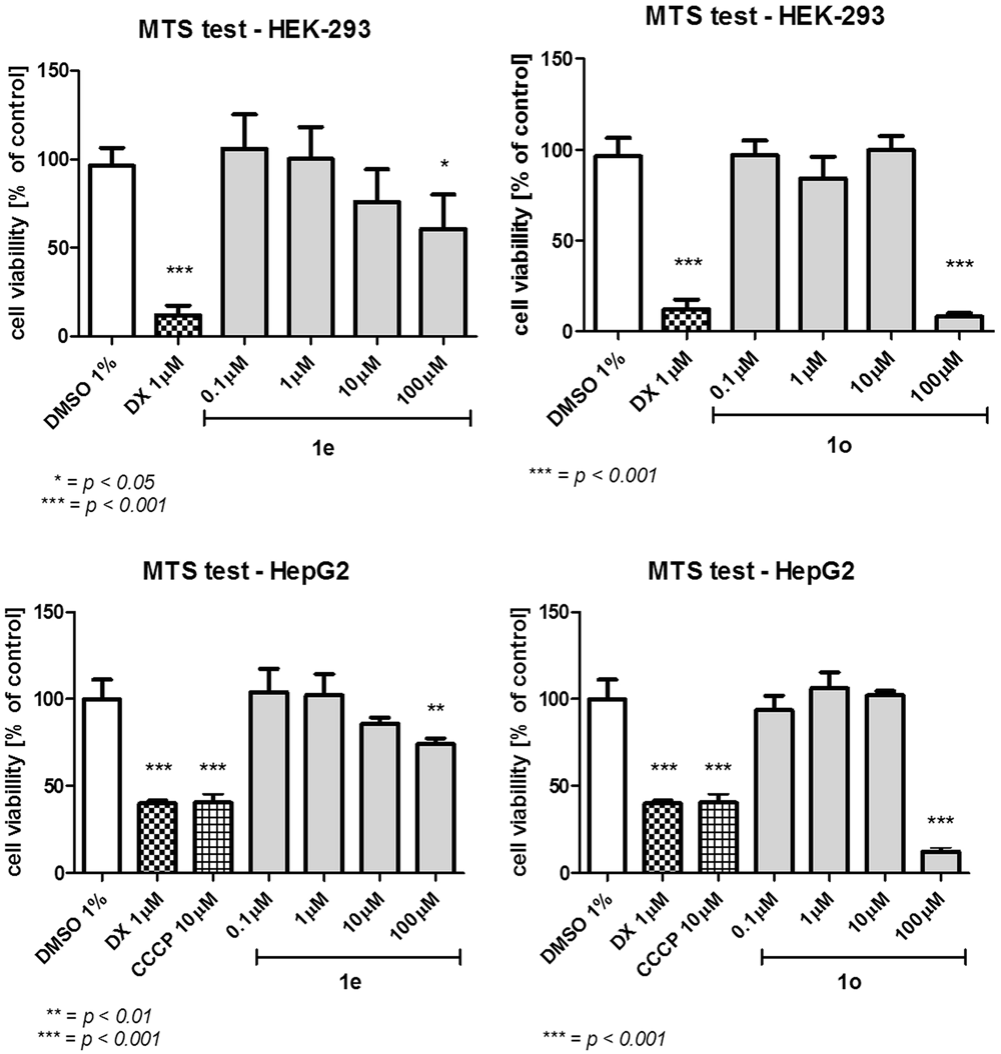
The HEK-293 and HepG2 cell lines viability in the presence of **1e** and **1o** after 72 h of incubation.

The cytotoxicity of the compounds **1e** and **1o** was tested by checking the viability of HEK-293 and HepG2 cells after incubation with different concentrations of the compounds in the medium. **1o** was found toxic for the cells only in the highest concentration (100 μM), while with **1e** the cells remained viable in all tested concentrations (Fig. 3).

### Preliminary pharmacokinetics and behavioural study

**Figure 4:**
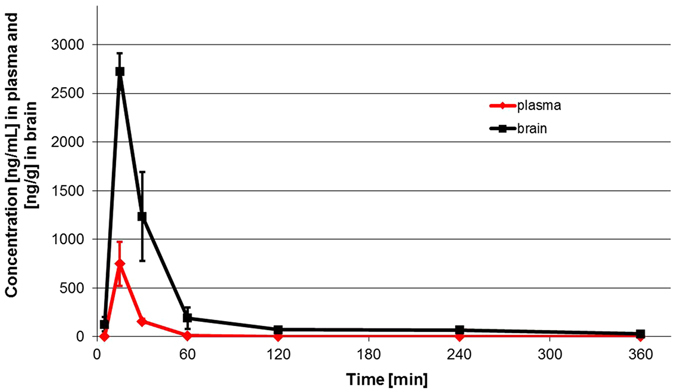
Concentration – time profiles for **1o** in plasma and brain after intraperitoneal administration to mice at a dose of 5 mg/kg (linear plots). Concentrations of **1o** in plasma and brain are expressed as ng/mL and ng/g, respectively.

The pharmacokinetic analysis performed by a non-compartmental approach has shown a rapid absorption of **1o** after *i*.*p*. administration (t_max_ = 15 min) with maximum plasma concentration of 749 ng/mL (~2.2 μM) (Fig. 4). Half of the compound was eliminated from the body within 90 min but brain concentrations of compound **1o** decreased slowly with t_0.5_ = 130 min. Large volume of distribution (V_d_/F = 46.6 L/kg) indicated the penetration of **1o** into the peripheral compartments. The compound **1o** was measurable to the last sampling time (6 h) both in plasma and brain. The compound penetrated the blood-brain barrier after *i*.*p*. administration and peaked in the brain and plasma at the same time but the maximum concentration in the brain was higher (C_max_ = 2723 ng/g, approx. 8 μM). The mean residence time (MRT) for compound **1o** in brain was longer than in plasma and equalled 60 min and 27 min, respectively. The brain to plasma ratio was high and equal 6.34. The detailed description of the pharmacokinetic experiment can be found in the Supporting Information. In a recent paper by Leopoldo *et al*.^31^ a disposition study for 5-HT_7_R agonists LP-44 and LP-211 after *i*.*p*. administration at a dose of 10 mg/kg in mice was reported. Despite **1o** reached the C_max_ in approx. 15 min, which is roughly the same for both LP compounds, the maximum concentrations of LP-44 and LP-211 in the brain were found several-fold lower than for **1o**, despite the dose used was twice as high as that of **1o**. The LP compounds were measurable in plasma for a very short time compared to over 6 hours for **1o** which can be attributed to their very low metabolic stability. The blood/plasma ratio for **1o** was three-fold higher compared to LP-44 and 9 times higher than for LP-211.

**Figure 5:**
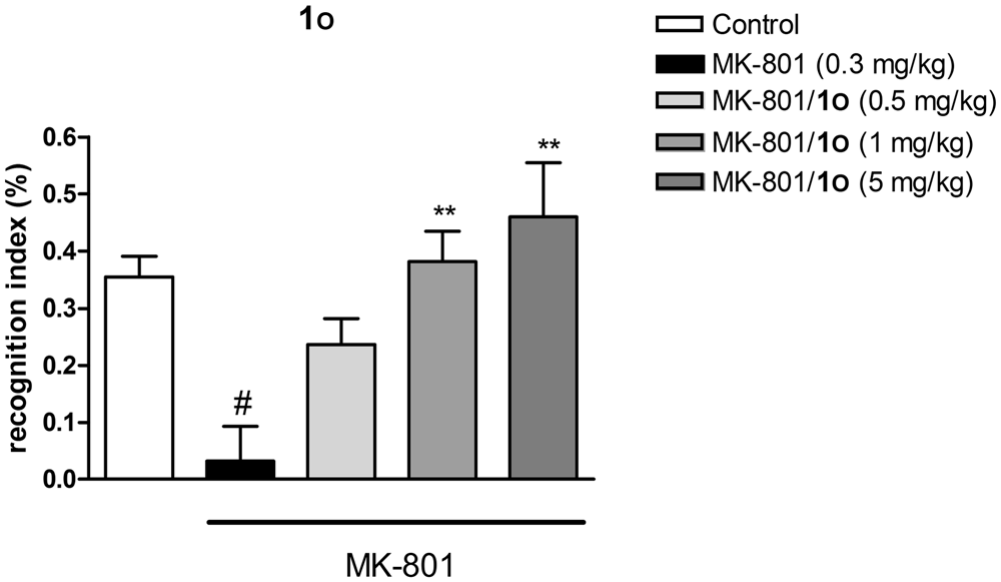
Reversal of MK-801 induced disruption in novel object recognition. Statistically significant effect was found at 1 mg/kg and 5 mg/kg doses.

Considering the PK study and suitable brain penetration we concluded that **1o** has desirable pharmacokinetic profile to be characterized as a compound of choice to study the *in vivo* pharmacology. The compound dose-dependently reversed MK-801-induced disruption in novel object recognition (NOR) in mice (Fig. 5). This finding is in line with the results obtained earlier for 5-CT^49^. MK-801-induced disruption of novel object recognition in rodents reflects impairment of working memory, regarded as one of the cognitive symptoms of schizophrenia.

### Molecular modelling

For all compounds in the series, Multiparameter Optimization (MPO) based on six factors (molecular weight, number of hydrogen bond donors, calculated: basic p*K*_a_, topological polar surface area, logP, logD) was performed. MPO parameter which was calculated according to Wager *et al*.^50^ was found to lie between 4–6 for most of the compounds in the series (Fig. 2).

**Figure 6:**
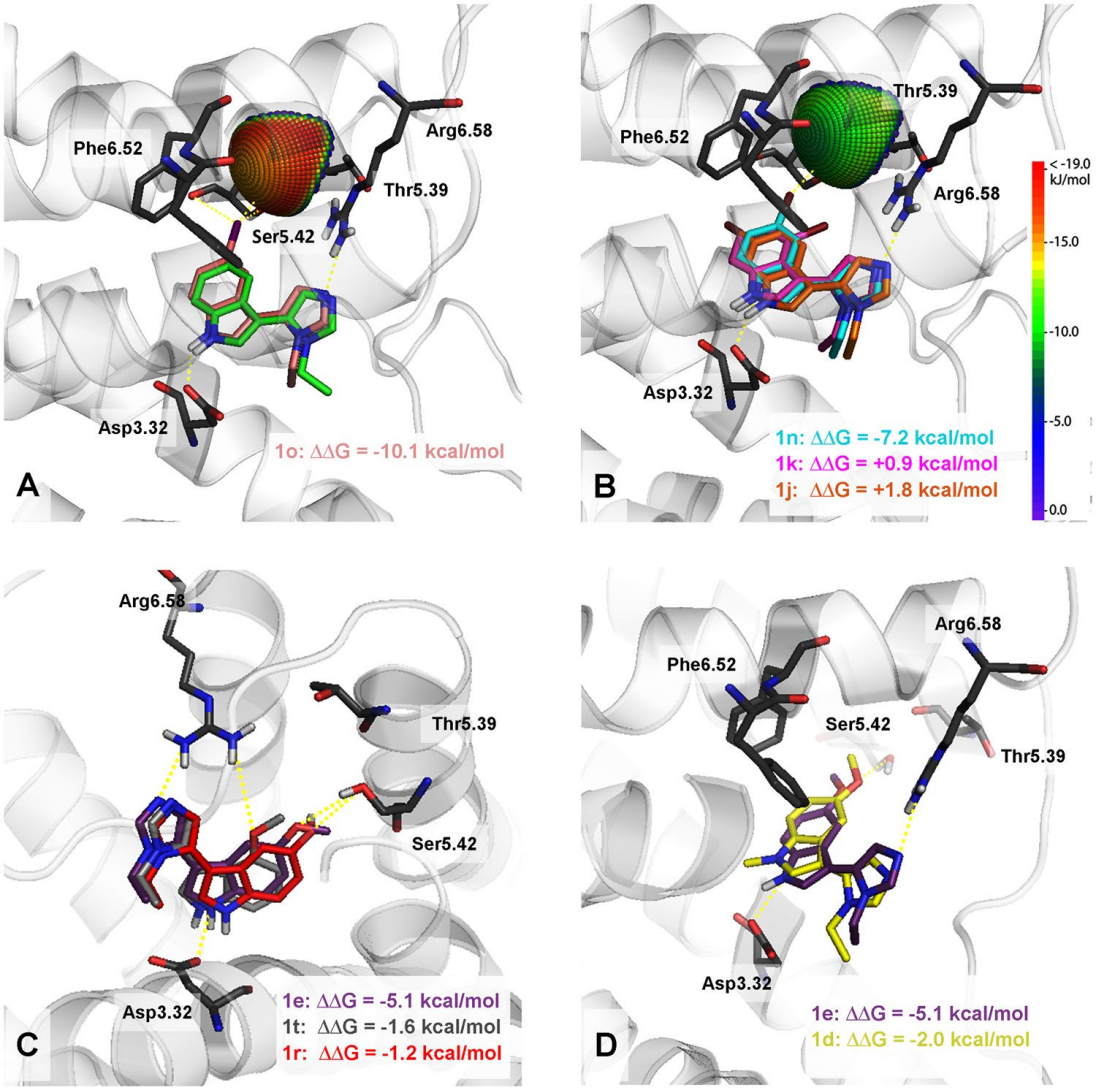
Representative complexes (top scores based on ΔG) of selected ligands with the 5-HT_7_ receptor model. Amino acids that were selected as crucial for the binding of the presented compounds are shown as sticks. The ∆∆G [kcal/mol] value shows the difference between the ∆G of complexes of a particular compound (**1d**, **1 f**, **1j**, **1k**, **1n**, **1r**, **1t**) and an unsubstituted analogue **1w** (green in Fig. A). In all cases except **1d** (R2 = Me, yellow in Fig. D), the ∆∆G values correspond to the binding affinity. (**A**) – halogen bond formation between the iodine atom of **1o** and the receptor. (**B**) – **1j** (R5 = Br) and **1k** (R3 = Br) which do not pass geometrical parameters needed for halogen bond formation and **1n** halogen bonded to the receptor. (**C**) – differences in binding modes of compounds **1e** (R4 = OMe), **1r** (R4 = OH) and **1t** (R3 = OMe), the lack of interaction of **1t** with Ser5.42 is compensated by the formation of additional hydrogen bond with Arg6.58. (**D**) – the inability of **1d** (R2 = Me) to interact with Asp3.32.

A very promising group of 5-HT_7_ receptor agonists has been developed. To make the search for highly active molecules more rational and explain the unusually high selectivity, a binding mode model had to be explored. The proposed binding mode was based on the analysis of the mutual spatial arrangement of particular ligands at the 5-HT_7_ receptor homology models. The final analysis and visualizations were performed using a model built on a 5-HT_1B_R (4IAR, complex with agonist, ergotamine) template, because it showed the most coherent binding mode, combined with low binding free energies (see Experimental Section). The observed structure-activity relationships were in agreement with the docking results. Quantum chemical calculations of p*K*_a_ for **1w** (performed in Jaguar using DFT functional B3PW91 and the cc-pVTZ basis set) showed an imidazole fragment p*K*_a_ = 6.9, indicating that the molecules at physiological pH = 7.4 occur in protonated (33%) as well as deprotonated (67%) forms. Two protonation states for each ligand were considered in docking study. The virtual ligand-receptor complexes of the protonated states did not follow the observed SAR, in addition, a more coherent binding mode was found for the unprotonated forms of the compounds. Thus in discussion and visualizations only deprotonated ligand-receptor complexes were used. The indole nucleus in all the docked ligands formed a hydrogen bond with Asp3.32 and aromatic interactions (CH-π or π-π stacking) with the Phe6.51 or Phe6.52 residues (Fig. 6A), whereas the imidazole ring was frequently hydrogen bonded to Arg6.58. It should be emphasized, that among all targets tested *in vitro*, the arginine in position 6.58 is a unique feature of 5-HT_7_R (Table 3)^51^, which could explain the high selectivity of the discussed compounds.

**Table 3:** Amino acid residue at 6.57, 6.58 and 6.59 positions in relevant targets.

Target	5-HT_1A_R	5-HT_2A_R	5-HT_2B_R	5-HT_6_R	5-HT_7_R	D_2_R
6.57 residue	Val	Met	Thr	Val	Ala	Leu
6.58 residue	Leu	Ala	Leu	Gln	Arg	Asn
6.59 residue	—	Val	Val	Ala	—	Ile

The substitution of hydrogen to a halogen atom in position 5 of the indole ring, proportionally increased the 5-HT_7_R affinity to the size of the halogen atom. The analysis of binding modes, showed that a significant contribution to the binding can be attributed to the halogen bond created between the ligand and carbonyl oxygen of one or several amino acids of transmembrane helix 5 (TMH5). The geometric parameters for the 5-iodine derivative showed the significant contribution of Ser5.42 (d(I···O) = 3.1 Å, sigma hole angle (C–I···O) = 155°) and Thr5.39 (d(I···O) = 4.4 Å, sigma hole angle (C-I···O) = 171°) (Fig. 6A). Additionally, the order of the binding free energy changes (after chlorine, bromine and iodine substitution ∆∆G was −6.6, −7.2 and −10.1 kcal/mol, respectively) and the interaction sphere plotted onto the relevant backbone carbonyl oxygen (all X···O=C interactions were within the energetically favourable areas of the sphere, Fig. 6A and B) confirmed the role of halogen bonding in the formation of the L-R complexes. The interaction spheres plotted onto the backbone carbonyl oxygen of Ser5.42 were also used to explain the decreased activity upon shifting the bromine atom from position 5 to 4 or 6 in the indole ring (Fig. 6B). The obtained interaction sphere showed that 5-Br derivative (d(Br···O) = 3.2 Å, sigma hole angle (C-Br···O) = 159°) lies within the energetically favourable areas of the sphere, while the 4- and 6-Br derivatives bromine atoms were pointed outside the sphere, ruling out the formation of halogen bond. This observation is consistent with calculated binding free energy values: only 5-bromo derivative **1n** showed a gain in the binding energy (ΔΔG = −7.6 kcal/mol), compared to the 4-bromo and 6-bromo analogues (ΔΔG = +0.9 and +1.8 kcal/mol, respectively).

The significance of the hydrogen bond formed between the indole NH group and the Asp3.32 side chain can be observed by comparing compounds **1e** and **1d** (Figs 2 and 6D). The substitution of hydrogen with a −CH_3_ group resulted in an approximately 580-fold decrease in binding affinity because no hydrogen bond can be formed with Asp3.32. The calculated ΔΔG for the methylated derivative (**1d**) showed the stabilization of the L-R complex which is in sharp contrast with the experimental *K*_i_ value (>10000 nM).

**Figure 7:**
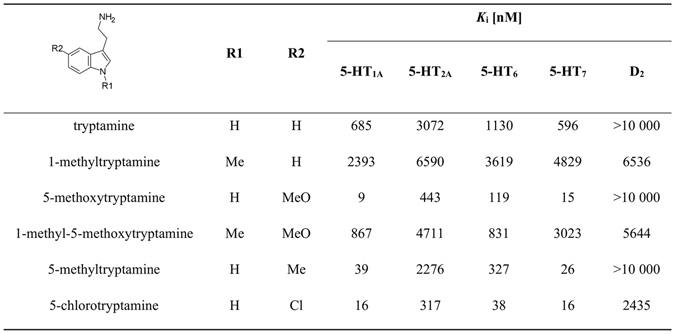
Structure and pharmacological profile of tryptamine analogues.

Because the discussed compounds are structurally very similar to the class of tryptamines that are known to be promiscuous serotonergic agents, representative tryptamine derivatives were purchased and their binding affinities for 5-HT_7_, 5-HT_1A_, 5-HT_2A_, 5-HT_6_ and D_2_ receptors were determined (Fig. 7).

**Figure 8:**
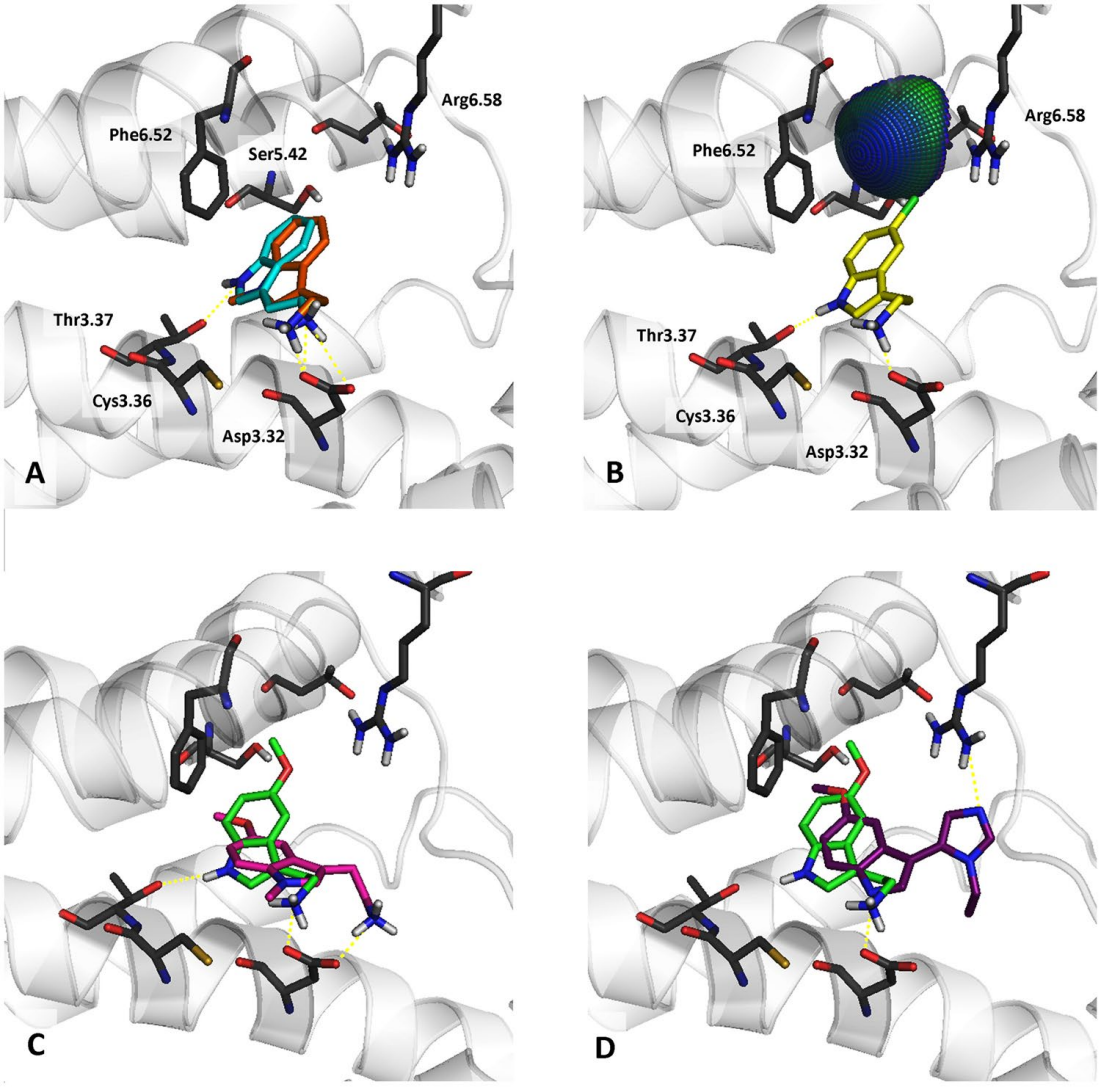
Representative complexes of selected tryptamines with 5-HT_7_ receptor. Amino acids that were selected as crucial for the binding of the presented compounds are shown as sticks. (**A**) – the superposition of tryptamine (cyan) and 1-methyltryptamine (orange). (**B)** – the halogen bond formed between Ser5.42 and 5-chlorotryptamine (yellow). (**C**) – the superposition of 5-methoxytryptamine (green) and 1-methyl-5-methoxytryptamine (magenta). (**D**) – a comparison of the binding modes of **1f** (violet) and the analogous 5-methoxytryptamine.

It is clear that tryptamines (tryptamine as an analogue for **1a**; 5-methoxytryptamine for **1b**, **1e**; 5-chlorotryptamine for **1m**, and 5-methyltryptamine for **1q**) exhibit multi-receptor profiles, i.e. a high affinity for 5-HT_7_, 5-HT_1A_, 5-HT_2A_, and 5-HT_6_ receptors. The introduction of halogen at the 5^th^ position of indole in tryptamines resulted in an enhancement of the affinity for 5-HT_7_R, but not nearly as much as the introduction of a methoxy group (introduction of the chlorine and methoxy groups had very similar impacts on the binding affinity of the title imidazoles). The methylation of the indole NH group in tryptamines resulted in a dramatic drop in potency for all four tested serotonin receptors. It was rather unusual that the tryptamines exhibited very similar 5-HT_7_R SARs while exhibiting distinctive multi-receptor profiles. The proposed binding mode for the imidazole compounds was compared with several tryptamines, which were docked to the best 5-HT_7_R homology models using the same procedure (Fig. 8). The binding mode of the tryptamine derivatives was similar to the findings of Vermeulen *et al*.^52^ and was different from the one found for the title compounds. The tryptamines formed a salt bridge (charge assisted hydrogen bond) between the protonated amine moiety and Asp3.32, but did not interact with Arg6.58. Vermeulen suggested Thr5.43 to be the anchoring point of 5^th^ position substituent of tryptamines. The sharp drop in potencies of 1-methylated tryptamines at 5-HT_7_R contributes to the significance of indole NH – Thr3.37 interaction (Fig. 8A–D) Despite different binding modes (Fig. 8D), the 5-HT_7_R SARs of tryptamines and imidazoles are very similar due to involvement of indole 5-substitutent interaction with Ser5.42 (or Thr5.39) in both series (compare Figs 6C and 8B). Ser5.42 can act as a donor/acceptor of hydrogen bonding through the side chain hydroxyl group. The docking results of the imidazole derivatives substituted in position 5 of the indole by –OCH_3_ and –OH (**1 f** (AGH-87) and **1r**, Fig. 6C) indicated the formation of a hydrogen bond between the hydroxyl group of Ser5.42 and the oxygen atom of substituents. Interestingly, shifting the –OCH_3_ group from position 5 to 4, resulted in only a slight decrease in activity, which can be linked to creation of a hydrogen bond with Arg6.58 (Fig. 6C).

Apparently, it is possible to synthesize heterocyclic analogues of tryptamines that maintain high affinity and agonist function at 5-HT_7_R. Modelling studies revealed that the incorporation of an amine moiety into an imidazole ring resulted in a distinctive binding mode. The difference may be attributed partially to the lower basicity of imidazoles versus tryptamines. An interaction with Arg6.58, which is a residue unique for 5-HT_7_R may underlay the observed dramatic effect on the selectivity.

### Possible application as a PET radioligand

5-HT_7_ receptor is yet to be PET imaged in humans^53^. No suitable 5-HT_7_R agonist radioligand candidates were reported till date^54, 55^. Serotonin receptors exist in high and low agonist affinity states. The antagonist ligands bind to the high affinity (HA) and low affinity (LA) conformations with similar affinity, whereas agonist ligands bind preferentially to the HA state of the receptor, which is coupled to G-protein and therefore agonist binding provides a more meaningful functional measure of the serotonin receptors activity. Although antagonist PET tracers can measure the total receptor binding, they cannot detect changes in the high affinity receptor binding in disease states. Antagonist binding is insensitive to the changes in the intra-synaptic 5-HT concentration and antagonist PET tracers are less sensitive for measuring agonist receptor occupancy in clinical studies to guide new drug development in dose-finding^56^.

**Figure 9:**
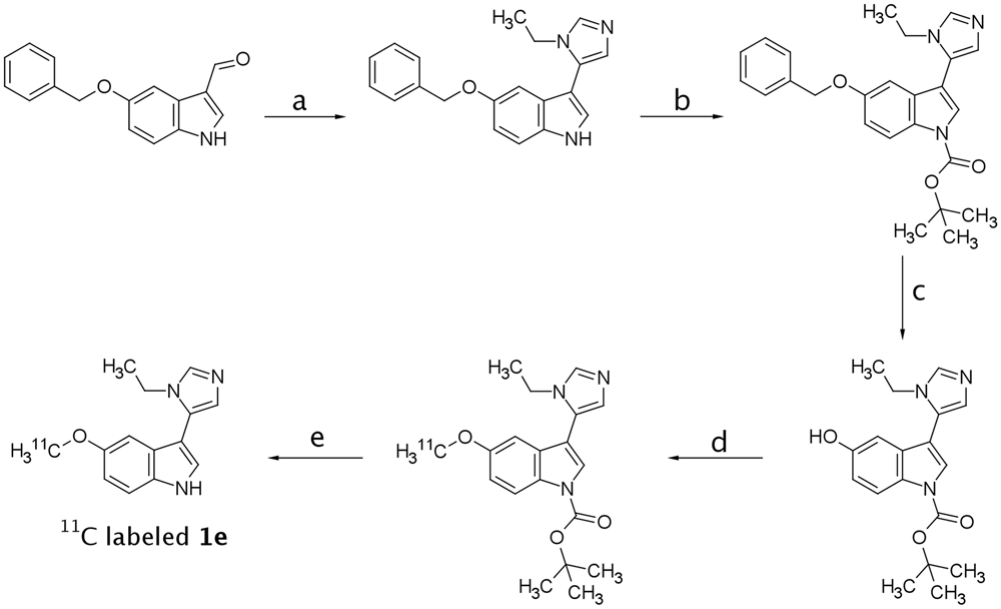
Proposed synthetic scheme for the synthesis of radiolabeled **1e**. Conditions: (a) – EtNH_2_, TosMIC, K_2_CO_3_, MeOH (85% - yield); (b) – Boc_2_O, DMAP, THF (85% yield); (c) – Pd/C, H_2_, MeOH (36% yield); (d) – ^11^CH_3_I, NaOH, toluene, H_2_O, TBAB (25% yield); (e) – HCl, MeOH (97% yield).

Because of rapid penetration to the brain and quick elimination from the CNS, **1o** showed desirable properties as perspective PET radiotracer. Therefore, a potential synthetic pathway leading to the ^11^C-labeled version of one of the lead compounds (**1e**) starting with commercially available substrates with only two concise steps involving “hot” chemicals has been developed (Fig. 9). The developed synthetic scheme included the sequential introduction of protecting groups to achieve the desired regioselectivity (Fig. 9). 5-Benzyloxyindole-3-carboxaldehyde was subjected to the MCR protocol to yield **1y**. This compound was *N*-Boc substituted to protect the indole NH group from alkylation during one of the last steps. The derivative was reductively debenzylated yielding *tert*-butyl 3-(1-ethyl-1*H*-imidazol-5-yl)-5-hydroxy-1*H*-indole-1-carboxylate, ready for alkylation with ^11^CH_3_I. After alkylation with a non-radioactive CH_3_I, Boc group was easily cleaved using either hydrochloric or trifluoroacetic acid. A similar strategy, involving synthesis of a *N*-Boc protected indole derivative, ready for the introduction of an isotopically labelled alkyl fragment to the 1-position of imidazole, may be employed for the synthesis of **1o**, or other 5-HT_7_R agonists.

## Conclusions

To date there have been no examples of low-basicity agonists of serotonin receptors. We report a series of highly drug-like, low-basicity 5-HT_7_R agonists synthesized using MCR imidazole synthesis. The procedure described by van Leusen proceeded under various conditions: a range of solvents and bases are tolerable. Notably, the scope of the carbonyl and amine reagents is very broad. Hydroxy-, amino-, amido-, ester and other reactive groups do not interfere with the reaction.

There are several highly active, selective 5-HT_7_R ligands within the presented series. Hit compound **1o** exhibits high affinity towards the receptor (*K*_i_ = 6 nM), intrinsic activity (EC_50_ = 19 nM), low intrinsic clearance (Cl_int_ = 6.3 ml/min/kg, human liver microsomes) and high predicted drug-likeness. These facts combined with an exceptional ease of synthesis may contribute to the use of radiolabeled **1o** and **1e** as potential molecular probes. **1e** has 5-HT_1A_/5-HT_7_ receptor selectivity comparable to known 5-HT_7_R agonists, whereas **1o** is 176-fold selective. Both compounds are very selective towards 5-HT_5A_ which is not a common feature of 5-HT_7_R agonists. The compounds are metabolically stable in human liver microsomes, exhibit very low toxicity in HEK-293 and HepG2 cells and are water soluble. The blood-brain barrier permeation of **1o** was found very high, which is consistent with its high activity *in vivo*, where all three tested *i*.*p*. doses (0.5, 1 and 5 mg/kg) reversed MK-801 induced novel object recognition impairment in mice.

The proposed binding mode was developed by SAR analysis and homology modelling and involves the formation of a hydrogen bond between the indole NH group and Asp3.32. The imidazole fragment is proposed to interact with Arg6.58 which could explain the unusually high selectivity that is not observed in tryptamines. The similar 5-HT_7_R SAR patterns of tryptamines and the described compounds can be attributed to the interaction of the substituents of indole at the 5^th^ position with Ser5.42 leading to comparable binding affinity gains for the same substituents in the two series.

Structures of NCE’s in the area of medicinal chemistry have become increasingly complex due to the widespread application of the hit-to-lead strategy and the rising palette of structural modifications that enable ADME optimization. We suppose that a better strategy for radioligand searches may be to find simple scaffolds exhibiting moderate to high activity that upon decoration with substituents would retain or improve their affinity for a given target, rather then finding sophisticated structures via virtual or high-throughput screening that upon simplification may not yield any active scaffolds. Because the available chemical space of compounds with low molecular mass (MW < 250 Da) that pass drug likeness filters can be considered inexhaustible taking current synthetic methods into account, we suppose that there are still numerous privileged structures to be discovered. Quoting Richard Feynman: “There’s plenty of room at the bottom”.

## Methods

### Chemicals

All organic reagents were purchased from Sigma-Aldrich, Apollo Scientific Apollo Scientific Ltd, or Combi-Blocks and were used without purification. Solvents and inorganic reagents were acquired from Chempur. Reaction progress was monitored by TLC on Merck Silica Gel 60 F 254 on aluminium plates. Column chromatography was performed on Merck Silica Gel 60 (0.063–0.200 mm; 70–230 mesh ASTM).

### Analytical Methods

UPLC/MS analysis was performed on Waters TQD spectrometer combined with UPLC Acquity H-Class with PDA eLambda detector. Waters Acquity UPLC BEH C18 1.7 μm 2.1 × 100 mm chromatographic column was used, at 40 °C, 0.300 ml/min flow rate and 1.0 μL injection volume (the samples were dissolved in LC-MS grade acetonitrile, typically at a concentration of 0.1–1 mg/ml prior to injection). All mass spectra were recorded under electrospray ionization in positive mode (ESI+) and chromatograms were recorded with UV detection in the range of 190–300 nm. The gradient conditions used were: 80% phase A (water+ 0.1% formic acid) and 20% phase B (acetonitrile + 0.1% formic acid) to 100% phase B (acetonitrile + 0.1% formic acid) at 3.0 minutes, kept till 3.5 minutes, then to initial conditions until 4.0 minutes and kept for additional 2.0 minutes. Total time of analysis – 6.0 minutes.

^1^H and ^13^C NMR spectra were recorded on a Bruker Avance III HD 400 NMR spectrometer. All samples were dissolved in DMSO-d_6_ with TMS as the internal standard. The spectral data of the compounds refer to their free bases.

All presented compounds were of at least 95% purity as determined by LC-MS. Syntheses and characterization details for intermediate products and final compounds as well as the spectral data for all compounds is included in the Supporting Information.

### *In vivo* experiments

The pharmacokinetic experimental procedures were carried out in accordance with EU Directive 2010/63/EU and approved by the I Local Ethics Committee for Experiments on Animals of the Jagiellonian University in Krakow, Poland (approval number: 123/2015). The behavioural experiments were carried out in accordance with EU Directive 2010/63/EU and approved by the Local Ethics Committee for Experiments on Animals of the Institute of Pharmacology, Polish Academy of Sciences (approval number: 181/2016).

### General Procedure 1 for the Synthesis of Compounds 1a–1q, 1s–1 v, 1y–1za, 2b–2f, and 3a–3d

Aromatic aldehyde (3 mmol) was mixed with amine (15 mmol) in 20 ml dry methanol. Reaction mixture was left overnight to complete the imine formation although it can be TLC monitored (SiO_2_/CHCl_3_). Anhydrous K_2_CO_3_ (3 mmol) and TosMIC (tosylmethylisocyanide, 3 mmol) were subsequently added. The mixture was stirred for an additional 8 hours, diluted with 50 ml H_2_O, and extracted three times with 20 ml ethyl acetate. The combined extracts were washed twice with 20 ml H_2_O, and once with 20 ml brine, treated with anhydrous magnesium sulfate and evaporated. The final products were purified either by trituration under a 2:1 hexane:isopropanol mixture, or chromatography on a short silicagel bed. The unreacted aldehydes were eluted with ethyl acetate or chloroform, and then a mixture of AcOEt:MeOH or CHCl_3_:MeOH was applied to elute the product.

### General Procedure 2 for the Synthesis of Substituted Indole-3-carboxaldehydes

The Vilsmeier-Haack reagent was generated by the addition of 2 ml of POCl_3_ over the course of 15 minutes to 8 ml of dry DMF cooled in an ice-salt bath. After the addition was complete, the ice bath was removed and the contents of the flask were left to warm to room temperature over approx. 30 minutes. The substituted indole (21.9 mmol) was dissolved in 10 ml of DMF and added over a period of 15 minutes to the formylating mixture. The stirring was continued for an hour during which the flask contents were heated to 40 °C in a hot water bath. A total of 20 ml of 5 M NaOH was added, and the mixture was diluted with H_2_O, quickly brought to a boil and left to cool slowly. The crystals were removed by filtration, washed with cold water and vacuum dried. The products thus obtained were in most cases sufficiently pure for the subsequent reactions. And the impure aldehydes were recrystallized from ethanol-water mixtures. The yields varied from 48 to 90%.

### General Procedure 3 for the Synthesis of Substituted 1-Alkylindole-3-carboxaldehydes

A total of 10 mmol of an appropriate indole-carboxaldehyde, 50 ml of toluene, 36 ml of 30% NaOH solution, 1 mmol (322 mg) tetrabutylammonium bromide (TBAB) and 10.5 mmol alkyl iodide (0.66 ml of methyl iodide, for instance) were placed in a stoppered flask. The mixture was stirred until the substrate disappeared on TLC (on average 12 h). After completion of the reaction, the phases were separated, next the organic extract was washed with 30 ml of H_2_O followed by 30 ml of brine, and was dried over MgSO_4_, and the toluene was stripped off in a rotary evaporator. The crude product was triturated twice with 5 ml of hexane:isopropanol 2:1 mixture, and was vacuum filtered and dried. The yields varied from 85 to 97%.

Unsubsituted indole-3-carboxaldehyde was synthesized at full scale according to the OrgSyn procedure^56^.

### General Procedure 4 for the Synthesis of 1r and 1x (Reductive Debenzylation)

The benzyl protected compound was dissolved in methanol in a pressure reactor, and 10 mol% Pd/C was added. The reactor was sealed and pressurized to 7 bar with H_2_, and the completion of the reaction was monitored with LC-MS. The mixture was then filtered through Celite which was then washed with MeOH. The solvent was stripped off on a rotary evaporator and the resulting solid was triturated under a hexane:isoporopanol:acetone 3:1:1 mixture. The yields were not lower than 90%.

### 3-(1-Ethyl-1*H*-imidazol-5-yl)-1*H*-indole-5-carboxamide (1za)

The compound was synthesized from **1p**, the nitrile group hydrolysis was accomplished according to a modified procedure outlined by Agarwal *et al*.^57^.

A suspension of **1p** (3.4 mmol, 0.8 g) in 1 ml MeOH and 5 ml THF was stirred in an ice-salt bath for 15 minutes until 0 °C was reached. Hydrogen peroxide (30% solution, 5.4 ml) was added dropwise keeping the temperature below 10 °C. Stirring was continued for 15 minutes and sodium hydroxide (20% solution, 5.4 ml) was added dropwise keeping temperature below 10 °C. The mixture was allowed to warm to room temperature and stirred for 24 hours. The product was extracted from the reaction mixture with chloroform and purified by trituration with acetone. Yield: 81 mg (9%).

### Radiosynthesis of 1e

#### *Tert*-butyl 5-(benzyloxy)-3-(1-ethyl-1*H*-imidazol-5-yl)-1*H*-indole-1-carboxylate (4)

To a round bottom flask there was added: THF (15 ml), **1y** (410 mg, 1.29 mmol), di-*tert*-butyl dicarbonate (590 mg, 2.7 mmol, 2.1 eq) and a catalytic amount of DMAP. The mixture was stirred until disappearance of the substrate on TLC. H_2_O (20 ml) and ethyl acetate (20 ml) were added, the phases separated, the aquous phase extracted twice with 10 ml ethyl acetate. The organic phase was washed with brine, dried over MgSO_4_ and evaporated. Crude product was of >95% purity and was used in the next step without purification. Yield: 485 mg (85%). An analytical sample was obtained via column chromatography (SiO2, ethyl acetate:hexane 2:1, then ethyl acetate).

#### *Tert*-butyl 3-(1-ethyl-1*H*-imidazol-5-yl)-5-hydroxy-1*H*-indole-1-carboxylate (5)

**4** (200 mg, 0.48 mmol) was dissolved in 50 ml MeOH in a glass reactor. 10% Pd/C (50 mg) was added and the reactor was pressurized to 7 bar with hydrogen. The mixture was stirred overnight, filtered through celite and the celite bed was washed thrice with 20 ml MeOH. The solvent was evaporated and the residue was subjected to column chromatography (SiO_2_, ethyl acetate). Yield: 57 mg (36%).

#### *Tert*-butyl 3-(1-ethyl-1*H*-imidazol-5-yl)-5-methoxy-1*H*-indole-1-carboxylate (6)

To a round bottom flask there were added: 3 ml toluene, **5** (100 mg, 0.305 mmol), CH_3_I (20 μL, 0.32 mmol, 1.05 eq), TBAB (10 mg, 0.0305 mmol, 0.1 eq.) and 1 ml 30% NaOH solution. The mixture was stirred and the progress of the reaction was TLC monitored. The mixture was diluted with 10 ml H_2_O, extracted three times with 10 ml ethyl acetate, the combined organic phase was washed with 10 ml H_2_O, 10 ml brine, dried over MgSO_4_ and evaporated. The crude product was purified via column chromatography (SiO_2_, ethyl acetate). Yield: 26 mg (25%).

#### 3-(1-Ethyl-1*H*-imidazol-5-yl)-5-methoxy-1*H*-indole hydrochloride (1e)

**6** (10 mg, 0.0293 mmol) was suspended in 2 ml MeOH and one drop of concentrated hydrochloric acid was added. The mixture was refluxed for 3 h, and the solvent was evaporated. Yield: 7.9 mg (97%).

### ***In Vitro*** Pharmacology. Cell Culture

HEK293 cells with stable expression of human serotonin 5-HT_1A_R, 5-HT_6_ and 5-HT_7b_R or dopamine D_2L_R (obtained using of Lipofectamine 2000, Invitrogen) or CHO-K1 cells with plasmid containing the sequence coding for the human serotonin 5-HT_2A_ receptor (Perkin Elmer) were maintained at 37 °C in a humidified atmosphere with 5% CO_2_ and were grown in Dulbecco’s Modified Eagle’s Medium containing 10% dialyzed fetal bovine serum and 500 μg/ml G418 sulfate. For membranes preparations, cells were subcultured into 150 cm^2^ cell culture flasks, grown to 90% confluence, washed twice with phosphate buffered saline (PBS) prewarmed to 37 °C, pelleted by centrifugation (200 g) in PBS containing 0.1 mM EDTA and 1 mM dithiothreitol, and stored at −80 °C^57–59^.

### 5-HT_1A_/5-HT_2A_/5-HT_6_/5-HT_7_/D_2_ Radioligand Binding Assays

The membrane preparation and general assay procedures for the cloned receptors were adjusted to 96-microwell format as described in our former papers^58–61^. The cell pellets were thawed and homogenized in 10 volumes of assay buffer using an Ultra Turrax tissue homogenizer and were centrifuged twice at 35,000 g for 15 min at 4 °C, with incubation for 15 min at 37 °C in between the rounds of centrifugation. The composition of the assay buffers was as follows: for 5-HT_1A_R: 50 mM Tris–HCl, 0.1 mM EDTA, 4 mM MgCl_2_, 10 μM pargyline and 0.1% ascorbate; for 5-HT_2A_R: 50 mM Tris–HCl, 0.1 mM EDTA, 4 mM MgCl_2_ and 0.1% ascorbate; for 5-HT_6_R: 50 mM Tris–HCl, 0.5 mM EDTA and 4 mM MgCl_2_, and for 5-HT_7b_R: 50 mM Tris–HCl, 4 mM MgCl_2_, 10 μM pargyline and 0.1% ascorbate; for dopamine D_2L_R: 50 mM Tris–HCl, 1 mM EDTA, 4 mM MgCl_2_, 120 mM NaCl, 5 mM KCl, 1.5 mM CaCl_2_ and 0.1% ascorbate.

All assays were incubated in a total volume of 200 μL in 96-well microtiter plates for 1 h at 37 °C, except for 5-HT_1A_R and 5-HT_2A_R, which were incubated at room temperature and 27 °C, respectively. The equilibration process was terminated by rapid filtration through Unifilter plates with a 96-well cell harvester and the radioactivity retained on the filters was quantified using a Microbeta plate reader (PerkinElmer, USA).

For the displacement studies, the assay samples contained the following as radioligands (PerkinElmer, USA): 1.5 nM [^3^H]-8-OH-DPAT (135.2 Ci/mmol) for 5-HT_1A_R; 2 nM [^3^H]-ketanserin (53.4 Ci/mmol) for 5-HT_2A_R; 2 nM [^3^H]-LSD (83.6 Ci/mmol) for 5-HT_6_R, 0.6 nM [^3^H]-5-CT (39.2 Ci/mmol) for 5-HT_7_R or [^3^H]-Raclopride (74.4 Ci/mmol). Non-specific binding was defined using 10 μM of 5-HT in 5-HT_1A_R and 5-HT_7_R binding experiments, whereas 20 µM of mianserin, 10 μM of methiothepine or 1 μM of (+) butaclamol was used in the 5-HT_2A_R, 5-HT_6_R and D_2L_R assays, respectively. Each compound was tested in triplicate at 7–8 concentrations (10^–11^–10^–4^ M). The inhibition constants (*K*_i_) were calculated using the Cheng-Prusoff equation^62^ and the results were expressed as the means of at least two independent experiments.

### Functional Assay

The functional properties of compounds in HEK293 cells overexpressing 5-HT_7_R were evaluated for their ability to increase cAMP production for the agonists or to inhibit 10 nM 5-CT at a concentration producing 90% (EC_90_) of the maximum agonist activation for the antagonists.

Each compound was tested at 8 concentrations in the range of 10^−11^–10^−4^ M. Cells (prepared with the use of Lipofectamine 2000) were maintained at 37 °C in a humidified atmosphere with 5% CO_2_ and were grown in DMEM containing 10% dialyzed (FBA) and 500 µg/ml G418 sulfate. For the functional experiments, the cells were subcultured in 25 cm^2^ diameter dishes, grown to 90% confluence, washed twice with PBS prewarmed to 37 °C and centrifuged for 5 min (160 × g). The supernatant was aspirated, and the cell pellet was resuspended in stimulation buffer (1 × HBSS, 5 mM HEPES, 0.5 mM IBMX, and 0.1% BSA). The total cAMP was measured using the LANCE cAMP detection kit (PerkinElmer), according to the manufacture’s directions. For the cAMP level quantification, cells (5 µl) were incubated with compounds (5 µl) for 30 min at room temperature in a 384-well white opaque microtiter plate. After incubation, 10 µl working solution (5 µl Eu-cAMP and 5 µl ULight-anti-cAMP) was added to stop the reaction and induced cells lysis. The assay plate was incubated for 1 h at room temperature and time-resolved fluorescence resonance energy transfer (TR-FRET) was detected by an Infinite M1000 Pro (Tecan, Männedorf, Switzerland) using instrument settings from the LANCE cAMP detection kit manual.

### Molecular Modeling. Homology Modeling

Homology models of the 5-HT_7_ receptor were built on a GPCR crystal structures co-crystalized with agonist molecules, i.e. 5-HT_1B_ (PDB ID: 4IAR, 4IAQ), 5-HT_2B_ (PDB ID: 4IB4), A_2A_ (PDB ID: 3QAK, 2YDV, 4UHR), β2 (PDB ID: 3P0G, 4LDE, 3PDS) and M_2_ (PDB ID: 4MQS) receptors, retrieved from the Protein Data Bank^63^. Sequences of the modeled receptor and selected templates were aligned manually using Accelrys Discovery Studio^64^, making sure that the most conserved amino acid in each helix, and the motif characteristic for class A GPCRs were in equivalent positions. The ranges of helices were determined on the basis of the crystal structures, and the loop regions were modeled, but not refined. For each template, 20 models were generated using Modeller software^65^, and were tested by the docking of active and inactive compounds, and enrichment calculation. For every model, ROC curves were calculated based on the Glide Score values of the docked compounds (the undocked actives and inactives were considered as false negatives and true negatives, respectively). The quality of the models was determined by the Area Under the ROC curve (AUROC). Three models per template with the highest AUROC value were selected for further studies. The final validation of the selected receptor models was performed by using the QM/MM molecular docking (QPLD Protocol) of the whole set of studied molecules. The models for which binding modes coherent for the whole set of compounds and explaining the main structure-activity relationships were observed were kept.

### CNS MPO Calculation

The Central Nervous System Multiparameter Optimization (CNS MPO) scores were calculated based on the six basic physicochemical molecular descriptors: logP, logD, molecular weight (MW), topological polar surface area (TPSA), the count of hydrogen bond donor (HBD), and the strongest basic pKa. All descriptors were calculated using Chemaxon’s Calculator Plugins^66^. A monotonically decreasing function was used to transform the values of the descriptors into dimensionless scale logP, logD, MW, HBD, and pKa whereas a hump function was used to transform TPSA. The most desirable and least desirable ranges for each physicochemical descriptor were adopted from the original paper^50^. Transformed values (desirability score) of the six descriptors were determined and summed up for each compound, to obtain the final CNS MPO, which can range from 0 (the worst CNS drug) to 6 (best CNS drug).

### QM/MM Docking Protocol

The Quantum Mechanics/Molecular Mechanics (QM/MM) docking of all synthesized compounds to 5-HT_7_R homology models were performed by means of the QM-Polarized Ligand Docking Protocol (QPLD)^67^ from Schrödinger Suite. At the first stage of the QPLD procedure the ligands were initially docked into a rigid protein using Glide. The resulting binding modes of the ligands were then used for the calculation of the partial charges of the ligand by a single-point calculation in QSite treating the ligand with the ab initio (B3PW91/cc-pVTZ) and the receptor with MM (OPLS-2005) level of theory. The partial atomic charges of the ligand molecules were recalculated using the electrostatic potential fitting method under the influence of external fields exerted by the surrounding protein atoms within and near the binding site. At the final stage, Glide re-docked the ligands using new charges, and the 10 most energetically favorable poses were returned for each ligand.

### Binding Free Energy Calculation

MM-GBSA (Generalized-Born/Surface Area) was used to calculate the binding free energy for all ligand-receptor complexes to select the correct poses between possible binding modes. The binding free energy was calculated using ligand charges obtained via the QM/MM calculations using Prime software from Schrodinger. To assess the influence of a given substituent on the binding, the ΔΔG was calculated as a difference between binding free energy (ΔG) of unsubstituted (**1w**) and substituted analogues.

### Plotting Interaction Spheres for Halogen Bonding

To visualize (plotting interaction spheres) the possible contribution of halogen bonding for the resulting ligand-receptor complexes, the halogen bonding webserver was used (access 1.4.2016, http://www.halogenbonding.com/).

### Associated Content

Syntheses and characterization details for intermediate products and final compounds, cell culture and transfection methods, pharmacological data for compound **2a**, detailed description of: metabolic stability assays for compound **1e** and **1o**, cytotoxicity assays, metabolic stability assays, pharmacokinetic study, novel object recognition experiment, ^1^H NMR, ^13^C NMR and LC-MS spectra (PDF).

Molecular formula strings (CSV).

## Electronic supplementary material

Supporting information
