# Supplementary material for: Low-basicity 5-HT7 Receptor Agonists Synthesized Using the van Leusen Multicomponent Protocol
Source: Sci Rep. 2017 May 4;7:1444. doi: 10.1038/s41598-017-00822-4 (PMC5431432; doi:10.1038/s41598-017-00822-4)
Supplement: Supplementary file 1 — Supporting information [file 41598_2017_822_MOESM1_ESM.pdf]

Supporting Information

Low-basicity 5-HT_7 Receptor Agonists synthesized Using the van Leusen Multicomponent Protocol

*Adam S. Hogendorf,[a,] Agata Hogendorf,[a] Rafał Kurczab,[a] Grzegorz Satała,[a] Tomasz Lenda,[a] Maria Walczak,[b] Gniewomir Latacz,[b] Jadwiga Handzlik,[b] Katarzyna Kieć-Kononowicz,[b] Joanna M. Wierońska,[a] Monika Woźniak,[a] Paulina Cieślik,[a] Ryszard Bugno,[a] Jakub Staroń,[a] Andrzej J. Bojarski[a,]**

[a]Institute of Pharmacology, Polish Academy of Sciences, 12 Smętna Street, 31-343 Kraków, Poland,

[b]Faculty of Pharmacology, Jagiellonian University Medical College, 9 Medyczna Street, 30-688 Krakow, Poland,

Corresponding author email: andrzej.bojarski@if-pan.krakow.pl

Table of contents:

I. Characterization data of intermediate products.. S2

II. Characterization data of final products.. S4

III. Cell culture and transfection.. S20

IV. Pharmacological data for compound **2a** (AGH-54)... S21

V. Metabolic stability assay for compounds **1e** (AGH-44) and **1o** (AGH-107)............................... S22

VI. Cytotoxicity assay and CYP3A4 binding for for compounds **1e** and **1o**................................... S29

VII. Pharmacokinetic analysis for compound **1o**.. S33

VIII. Novel Object recognition experiment for compound **1o**... S37

IX. ^{1}H NMR, ^{13}C NMR and LC-MS spectra of intermediates and key products............................. S38

I. Characterization data of intermediate products:

5-methoxy-1*H*-indole-3-carboxaldehyde

The compound was synthesized from 5-methoxyindole (170 mmol) according to the general procedure 2, with the exception that it was scaled up proportionally to the 5-methoxyindole (69% yield).

MS (m/z ESI+) = 176.11, LCMS t_R = 2.20 min.

5-fluoro-1*H*-indole-3-carboxaldehyde

The compound was synthesized from 5-fluoroindole (22 mmol) according to the general procedure 2 (70% yield).

MS (m/z ESI+) = 163.78, LCMS t_R = 1.97 min

5-iodo-1*H*-indole-3-carboxaldehyde

The compound was synthesized from 5-iodoindole (22 mmol) according to the general procedure 2 (90% yield).

MS (m/z ESI+) = 271.75, LCMS t_R = 2.62 min

5-methyl-1*H*-indole-3-carboxaldehyde

The compound was synthesized from 5-methylindole (22 mmol) according to the general procedure 2 (88% yield).

MS (m/z ESI+)= 160.05,LCMS t_R = 2.15 min

5-iodo-7-fluoro-1*H*-indole-3-carboxaldehyde

The compound was synthesized from 5-iodo-7-fluoroindole (1.88 mmol) according to the general procedure 2, with the exception that it was scaled down proportionally to 5-iodo-7-fluoroindole (75% yield).

MS (m/z ESI+) = 289.82, LCMS t_R = 2.88 min.

4-bromo-1*H*-indole-3-carboxaldehyde

The compound was synthesized from 4-bromoindole (22 mmol) according to the general procedure 2 (53% yield).

MS (m/z ESI+) = 223.97 and 226.03, LCMS t_R = 2.49 min.

5-cyano-1*H*-indole-3-carboxaldehyde

The compound was synthesized from 5-cyanoindole (22 mmol) according to the general procedure 2 with an exception – no NaOH solution was added after quenching the Vilsmeier adduct with water to avoid nitrile hydrolysis (84% yield).

MS (m/z ESI+)= 170.9, LCMS t_R = 1.66 min.

5-chloro-1*H*-indole-3-carboxaldehyde

The compound was synthesized from 5-chloroindole (22 mmol) according to the general procedure 2 (48% yield).

MS (m/z ESI+) = 179.71, LCMS t_R = 2.37 min.

1-methyl-1H-indole-3-carboxaldehyde

The compound was synthesized from indole-3-carboxaldehyde (68.9 mmol) according to the general procedure 3. 80% yield.

MS (m/z ESI+) = 159.83 LCMS t_R = 2.09 min.

Tert-butyl-5-(benzyloxy)-3-(1-ethyl-1*H*-imidazol-5-yl)-1*H*-indole-1-carboxylate

(4)

MS (m/z ESI+) = 418.19 LCMS t_R = 2.93 min.

^{1}H NMR (500 MHz, DMSO-d_6, TMS, ppm) δ 1.22 (t, *J* = 7.2 Hz, 3H), 1.63 (s, 9H), 3.98 (q, *J* = 7.3 Hz, 2H), 5.15 (s, 2H), 7.04 – 7.15 (m, 3H), 7.28 – 7.35 (m, 1H), 7.34 – 7.43 (m, 2H), 7.42 – 7.48 (m, 2H), 7.77 (s, 1H), 7.86 (d, *J* = 1.0 Hz, 1H), 8.00 (d, *J* = 9.0 Hz, 1H).

^{13}C NMR (126 MHz, DMSO, TMS, ppm) δ 16.10, 27.65, 39.74, 69.54, 84.14, 103.47, 109.70, 114.65, 115.81, 123.20, 124.49, 127.54, 127.69, 128.36, 128.58, 129.14, 129.96, 137.19, 138.10, 148.85, 154.91.

Tert-butyl-3-(1-ethyl-1*H*-imidazol-5-yl)-5-hydroxy-1*H*-indole-1-carboxylate (5)

MS (m/z ESI+) = 328.01 LCMS t_R = 2.3 min.

^{1}H NMR (500 MHz, DMSO-d_6, TMS, ppm) δ 1.20 (dt, *J* = 34.1, 7.2 Hz, 3H), 1.63 (s, 9H), 4.01 (dq, *J* = 11.2, 7.2 Hz, 2H), 6.81 – 6.90 (m, 2H), 7.07 (d, *J* = 1.1 Hz, 1H), 7.71 (s, 1H), 7.82 – 7.92 (m, 2H), 9.35 (s, 1H).

^{13}C NMR (126 MHz, DMSO, TMS, ppm) δ 16.21, 27.72, 83.85, 104.23, 109.37, 114.05, 115.62, 115.66, 123.39, 124.27, 128.18, 128.40, 130.37, 138.04, 148.93, 153.82.

II. Characterization data of final products:

1a: 3-(1-methyl-1*H*-imidazol-5-yl)-1*H*-indole (AGH-38)

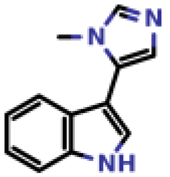

The compound was synthesized according to general procedure 1, purified via chromatography (SiO_2, $CHCl_3$, then 19:1 $CHCl_3$:MeOH). White solid, 0.357 g, 1.8 mmol, 91% yield. Mp = 88-90°C. MS (m/z ESI+) = 198.17, LCMS t_R= 0.77 min.

^{1}H NMR (500 MHz, DMSO-d_6, TMS, ppm) δ 11.48 (s, 1H), 7.74 (d, *J* = 1.1 Hz, 1H), 7.65 – 7.55 (m, 2H), 7.46 (dt, *J* = 8.1, 0.9 Hz, 1H), 7.21 – 7.03 (m, 3H), 3.66 (s, 3H).

^{13}C NMR (126 MHz, DMSO, TMS, ppm) δ 32.09, 103.74, 111.77, 119.01, 119.58, 121.69, 123.96, 126.06, 126.15, 126.86, 136.03, 138.09.

1b: 5-methoxy-3-(1-methyl-1*H*-imidazol-5-yl)-1*H*-indole (AGH-39)

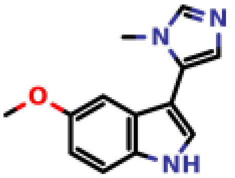

The compound was synthesized according to general procedure 1, purified by trituration with 2:1 hexane:isopropyl alcohol mixture.Off-white solid, 0.25 g, 1.1 mmol, 55% yield. Mp = 236°C. MS (m/z ESI+) = 228.1, LCMS t_R = 0.74 min.

^{1}H NMR (500 MHz, DMSO-d_6, TMS, ppm) δ 11.34 – 11.29 (m, 1H), 7.72 – 7.66 (m, 1H), 7.54 (d, *J* = 2.6 Hz, 1H), 7.35 (dd, *J* = 8.8, 0.6 Hz, 1H), 7.09 (d, *J* = 1.2 Hz, 1H), 7.01 (d, *J* = 2.5 Hz, 1H), 6.81 (dd, *J* = 8.8, 2.4 Hz, 1H), 3.76 (s, 3H), 3.65 (d, *J* = 0.5 Hz, 3H).

^{13}C NMR (126 MHz, DMSO, TMS, ppm) δ 31.96, 39.00, 55.26, 100.40, 103.66, 112.03, 112.51, 119.49, 124.51, 126.38, 126.50, 126.89, 131.07, 138.08, 153.90.

Elemental analysis: found C:68.5%, H: 5.76%, N: 18.41% $C_{13}H_{13}N_3O$ requires C:68.7%, H: 5.77%, N: 18.49%.

1c: 3-(1-ethyl-1*H*-imidazol-5-yl)-1-methyl-1*H*-indole (AGH-61)

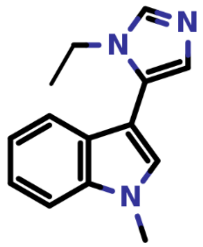

The compound was synthesized according to general procedure 1, purified via chromatography (SiO_2, AcOEt, then 49:1 AcOEt:MeOH). Dark orange oil which crystallized upon storage, 0.552 g, 2.45 mmol, 82% yield. MS (m/z ESI+) = 226.1, LCMS t_R = 0.42 min.

^{1}H NMR (500 MHz, DMSO-d_6, TMS, ppm) δ 7.78 (d, *J* = 1.2 Hz, 1H), 7.56 – 7.45 (m, 3H), 7.23 (ddd, *J* = 8.2, 7.0, 1.2 Hz, 1H), 7.11 (ddd, *J* = 8.0, 7.0, 1.0 Hz, 1H), 7.02 (d, *J* = 1.2 Hz, 1H), 4.00 (q, *J* = 7.2 Hz, 2H), 3.85 (s, 3H), 1.23 (t, *J* = 7.3 Hz, 3H).

^{13}C NMR (126 MHz, DMSO, TMS, ppm) δ 16.30, 32.57, 39.02, 56.13, 89.44, 103.02, 110.07, 119.10, 119.74, 121.78, 125.39, 126.78, 127.21, 128.17, 136.50, 137.06.

1d: 3-(1-ethyl-1*H*-imidazol-5-yl)-5-methoxy-1-methyl-1*H*-indole (AGH-68)

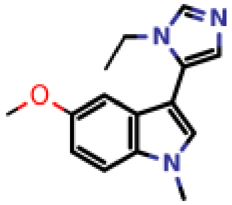

The compound was synthesized according to general procedure 1, purified via chromatography (SiO_2, AcOEt, then 49:1 AcOEt:MeOH). Pale brown solid, 0.153 g, 0.6 mmol, 20% yield. Mp = 91-93°C. MS (m/z ESI+) = 256.22, LCMS t_R = 1.83 min.

^{1}H NMR (500 MHz, DMSO-d_6, TMS, ppm) δ 7.77 (d, *J* = 1.1 Hz, 1H), 7.47 (s, 1H), 7.40 (dd, *J* = 8.9, 0.6 Hz, 1H), 7.01 (d, *J* = 1.1 Hz, 1H), 6.94 (dd, *J* = 2.4, 0.5 Hz, 1H), 6.87 (dd, *J* = 8.8, 2.4 Hz, 1H), 3.99 (q, *J* = 7.3 Hz, 2H), 3.81 (s, 3H), 3.75 (s, 3H), 1.30 – 1.19 (m, 3H).

^{13}C NMR (126 MHz, DMSO, TMS, pm) δ 16.31, 32.71, 55.29, 100.46, 102.59, 110.95, 112.00, 125.50, 127.05, 127.15, 128.65, 131.73, 136.96, 154.10.

1e: 3-(1-ethyl-1*H*-imidazol-5-yl)-5-methoxy-1*H*-indole (AGH-44)

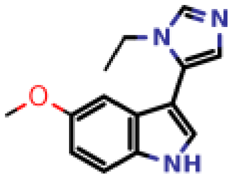

The compound was synthesized according to general procedure 1, purified via chromatography (SiO_2, AcOEt, then 19:1 AcOEt:MeOH) and additional trituration with hexane:acetone 9:1. Pale brown solid, 0.236 g, 0.98 mmol, 49% yield. Mp = 51°C. MS (m/z ESI+) = 242.16, LCMS t_R = 0.5-1.01 min.

^{1}H NMR (500 MHz, DMSO-d_6, TMS, ppm) δ 11.30 (s, 1H), 7.77 (d, *J* = 1.1 Hz, 1H), 7.47 (d, *J* = 2.6 Hz, 1H), 7.35 (dd, *J* = 8.8, 0.6 Hz, 1H), 7.02 (d, *J* = 1.1 Hz, 1H), 6.93 (d, *J* = 2.4 Hz, 1H), 6.80 (dd, *J* = 8.7, 2.4 Hz, 1H), 4.00 (q, *J* = 7.3 Hz, 2H), 3.74 (s, 3H), 1.23 (t, *J* = 7.2 Hz, 3H).

^{13}C NMR (126 MHz, DMSO, TMS, ppm) δ 153.87, 136.88, 131.05, 127.02, 126.85, 125.86, 124.71, 112.55, 112.02, 103.59, 100.14, 55.23, 39.42, 16.24.

Elemental analysis: found C:69.44%, H: 6.26%, N: 17.27% $C_{14}H_{15}N_3O$ requires C:69.69%, H: 6.27%, N: 17.41%

1f: 3-(1-propyl-1*H*-imidazol-5-yl)-5-methoxy-1*H*-indole (AGH-87)

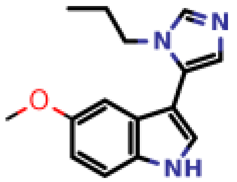

The compound was synthesized according to general procedure 1, purified via chromatography (SiO_2, AcOEt, then 49:1 AcOEt:MeOH). Dark orange oil which crystallized upon storage, 0.59 g, 2.3 mmol, 77% yield. MS (m/z ESI+) = 256.22, LCMS t_R = 1.44 min.

^{1}H NMR (500 MHz, DMSO-d_6, TMS, ppm) δ 11.30 – 11.26 (m, 1H), 7.74 (d, *J* = 1.1 Hz, 1H), 7.46 (d, *J* = 2.6 Hz, 1H), 7.35 (dd, *J* = 8.7, 0.6 Hz, 1H), 7.01 (d, *J* = 1.1 Hz, 1H), 6.91 (d, *J* = 2.4 Hz, 1H), 6.80 (dd, *J* = 8.8, 2.5 Hz, 1H), 3.97 – 3.90 (m, 2H), 3.74 (s, 3H), 1.63 – 1.52 (m, 2H), 0.74 (t, *J* = 7.4 Hz, 3H).

^{13}C NMR (126 MHz, DMSO, TMS, ppm) δ 172.06, 153.87, 137.59, 131.05, 127.09, 126.92, 124.70, 112.55, 111.99, 103.67, 100.14, 55.23, 46.06, 23.51, 10.83.

1g: 3-(1-butyl-1*H*-imidazol-5-yl)-5-methoxy-1*H*-indole (AGH-45)

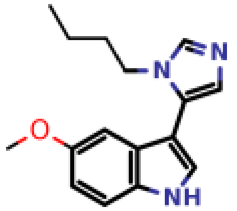

The compound was synthesized according to general procedure 1,purified via chromatography (SiO_2, $CHCl_3$, then 19:1 $CHCl_3$:MeOH). Dark orange oil which crystallized upon storage, 0.212 g, 0.79 mmol, 40% yield. MS (m/z ESI+) = 270.15, LCMS t_R= 1.76 min.

^{1}H NMR (500 MHz, DMSO-d_6, TMS, ppm) δ 0.75 (t, *J* = 7.4 Hz, 3H), 1.15 (h, 2H), 1.54 (p, 2H), 3.74 (s, 3H), 3.97 (t, *J* = 7.3 Hz, 2H), 6.80 (dd, *J* = 8.8, 2.5 Hz, 1H), 6.91 (d, *J* = 2.4 Hz, 1H), 7.01 (s, 1H), 7.34 (dd, *J* = 8.8, 0.6 Hz, 1H), 7.46 (d, *J* = 2.6 Hz, 1H), 7.75 (s, 1H), 11.27 (s, 1H).

^{13}C NMR (126 MHz, DMSO, TMS, ppm) δ 13.31, 19.12, 32.23, 44.15, 55.22, 100.12, 103.63, 111.99, 112.54, 124.74, 126.91, 127.01, 131.04, 137.53, 153.86, 172.01.

1h: 3-(1-cyclopropyl-1*H*-imidazol-5-yl)-5-methoxy-1*H*-indole (AH-427)

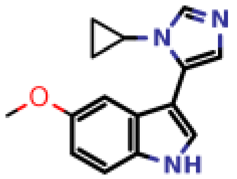

The compound was synthesized according to general procedure 1, purified via column chromatography (SiO_2, $CHCl_3$, then 19:1 $CHCl_3$:MeOH). Beige solid, 0.2 g, 0.79 mmol, 40% yield. Mp = 176°C. MS (m/z ESI+) = 254.09, LCMS t_R = 0.98 min.

^{1}H NMR (500 MHz, DMSO-d_6, TMS, ppm) δ 11.29 (s, 1H), 7.71 – 7.65 (m, 2H), 7.35 (dd, *J* = 8.7, 0.5 Hz, 1H), 7.11 (dd, *J* = 19.9, 1.8 Hz, 2H), 6.81 (dd, *J* = 8.7, 2.4 Hz, 1H), 3.77 (s, 3H), 3.46 (tt, *J* = 7.0, 4.1 Hz, 1H), 0.99 – 0.87 (m, 4H).

^{13}C NMR (126 MHz, DMSO, TMS, ppm) δ 153.84, 136.95, 130.96, 128.21, 126.19, 126.06, 124.29, 112.44, 111.91, 104.08, 100.63, 55.26, 27.14, 25.49, 6.99.

1i: 5-methoxy-3-[1-(prop-2-en-1-yl)-1*H*-imidazol-5-yl]-1*H*-indole (AGH-56)

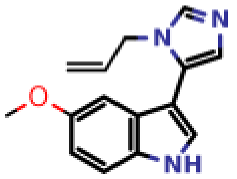

The compound was synthesized according to general procedure 1, purified via crystalization (AcOEt) and trituration with hexane:isopropanol 2:1 mixture. Off-white solid, 0.02 g, 0.08 mmol, 3% yield. Mp = 111°C. MS (m/z ESI+) = 254.09, LCMS t_R = 0.49-1.22 min.

^{1}H NMR (500 MHz, DMSO-d_6, TMS, ppm) δ 11.24 (s, 1H), 7.69 (d, *J* = 1.2 Hz, 1H), 7.41 (d, *J* = 2.6 Hz, 1H), 7.33 (dd, *J* = 8.7, 0.6 Hz, 1H), 7.08 (d, *J* = 1.1 Hz, 1H), 6.95 (d, *J* = 2.4 Hz, 1H), 6.79 (dd, *J* = 8.7, 2.4 Hz, 1H), 6.03 – 5.92 (m, 1H), 5.13 (dq, *J* = 10.4, 1.6 Hz, 1H), 4.89 (dq, *J* = 17.1, 1.7 Hz, 1H), 4.66 – 4.60 (m, 2H), 3.74 (s, 3H).

^{13}C NMR (126 MHz, DMSO, TMS, ppm) δ 153.88, 137.61, 134.86, 131.02, 126.81, 126.73, 126.31, 124.51, 116.50, 112.53, 112.04, 103.37, 100.23, 55.26, 46.60.

1j: 6-bromo-3-(1-ethyl-1*H*-imidazol-5-yl)-1*H*-indole (AGH-76)

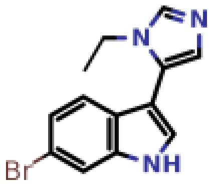

The compound was synthesized according to general procedure 1, purified via chromatography (SiO_2, AcOEt, then 19:1 $CHCl_3$:MeOH). Pale yellow sold, 0.452 g, 1.56 mmol, 70% yield. Mp = 162°C. MS (m/z ESI+) = 289.95 and 291.88 (for both bromine isotopes), LCMS t_R = 1.82 min.

^{1}H NMR (500 MHz, DMSO-d_6, TMS, ppm) δ 11.57 (s, 1H), 7.77 (d, *J* = 1.1 Hz, 1H), 7.64 (dd, *J* = 1.8, 0.5 Hz, 1H), 7.57 (d, *J* = 2.5 Hz, 1H), 7.45 (d, *J* = 8.5, 0.6 Hz, 1H), 7.19 (dd, *J* = 8.5, 1.8 Hz, 1H), 7.01 (d, *J* = 1.1 Hz, 1H), 3.99 (q, *J* = 7.2 Hz, 2H), 1.20 (t, 3H).

^{13}C NMR (126 MHz, DMSO, TMS, ppm) δ 137.21, 136.88, 127.41, 125.52, 125.10, 125.02, 122.46, 120.68, 114.38, 114.32, 104.18, 40.02, 39.49, 16.18.

1k: 4-bromo-3-(1-ethyl-1*H*-imidazol-5-yl)-1*H*-indole (AGH-84)

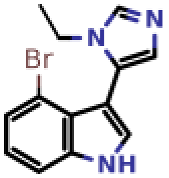

The compound was synthesized according to general procedure 1, purified via chromatography (SiO_2, AcOEt, then 19:1 $CHCl_3$:MeOH) and additional trituration with hexane:isopropanol 2:1 mixture. Orange solid, 0.075 g, 0.26 mmol, 9% yield. Mp = 207°C. MS (m/z ESI+) = 289.95 and 291.88 (for both bromine isotopes), LCMS t_R= 1.00 min.

^{1}H NMR (500 MHz, DMSO-d_6, TMS, ppm) δ 11.78 (s, 1H), 7.76 (d, *J* = 1.2 Hz, 1H), 7.59 – 7.45 (m, 2H), 7.22 (dd, *J* = 7.6, 0.8 Hz, 1H), 7.05 (t, 1H), 6.83 (s, 1H), 3.72 (q, *J* = 7.3 Hz, 2H), 1.13 (t, *J* = 7.3 Hz, 3H).

^{13}C NMR (126 MHz, DMSO, TMS, ppm) δ 137.19, 136.44, 129.72, 128.64, 125.47, 124.40, 123.46, 122.69, 112.48, 111.73, 103.29, 15.95.

1l: 3-(1-ethyl-1*H*-imidazol-5-yl)-5-fluoro-1*H*-indole (AGH-80)

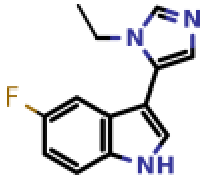

The compound was synthesized according to general procedure 1, purified via trituration with hexane:isopropanol 2:1 mixture. White solid, 0.163 g, 0.71 mmol, 24% yield. Mp = 163°C. MS (m/z ESI+) = 230.10, LCMS t_R = 1.47 min.

^{1}H NMR (500 MHz, DMSO-d_6, TMS, ppm) δ 11.56 (s, 1H), 7.77 (s, 1H), 7.62 (d, *J* = 2.6 Hz, 1H), 7.46 (ddd, *J* = 8.8, 4.7, 0.6 Hz, 1H), 7.23 – 7.17 (m, 1H), 7.05 – 6.97 (m, 2H), 4.00 (q, *J* = 7.2 Hz, 2H), 1.22 (t, *J* = 7.3 Hz, 3H).

^{13}C NMR (126 MHz, DMSO, TMS, ppm) δ 158.30, 156.46, 137.11, 132.69, 127.22, 126.78, 126.17, 125.22, 112.94, 110.05, 104.14, 103.57, 16.19.

1m: 5-chloro-3-(1-ethyl-1H-imidazol-5-yl)-1H-indole (AGH-79)

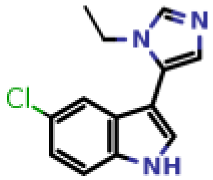

The compound was synthesized according to general procedure 1, purified via trituration with hexane:isopropanol 2:1 mixture. Pale orange solid, 0.369 g, 1.5 mmol, 50% yield. Mp = 221°C. MS (m/z ESI+) = 246.09, LCMS t_R = 1.75 min.

^{1}H NMR (500 MHz, DMSO-d$_6$, TMS, ppm) δ 11.67 (s, 1H), 7.78 (d, *J* = 1.1 Hz, 1H), 7.63 (s, 1H), 7.53 – 7.41 (m, 2H), 7.16 (dd, *J* = 8.6, 2.1 Hz, 1H), 7.03 (d, *J* = 1.1 Hz, 1H), 4.00 (p, *J* = 7.3 Hz, 2H), 1.27 – 1.11 (m, 3H).

^{13}C NMR (126 MHz, DMSO, TMS, ppm) δ 16.19, 39.40, 103.66, 113.41, 117.88, 121.71, 124.30, 124.90, 125.98, 127.45, 127.66, 134.48, 137.21.

1n: 5-bromo-3-(1-ethyl-1*H*-imidazol-5-yl)-1*H*-indole (AGH-75)

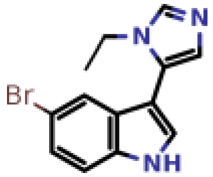

The compound was synthesized according to general procedure 1, purified via trituration with hexane:isopropanol 2:1 mixture. White solid, 0.29 g, 1 mmol, 45% yield. Mp = 231°C. MS (m/z ESI+) = 289.95 and 291.88 (for both bromine isotopes), LCMS t_R = 1.87 min.

^{1}H NMR (500 MHz, DMSO-d_6, TMS, ppm) δ 11.78 (s, 1H), 7.76 (d, *J* = 1.2 Hz, 1H), 7.59 – 7.45 (m, 2H), 7.22 (dd, *J* = 7.6, 0.8 Hz, 1H), 7.10 – 7.02 (m, 1H), 6.83 (d, *J* = 1.1 Hz, 1H), 3.72 (q, *J* = 7.3 Hz, 2H), 1.13 (t, *J* = 7.3 Hz, 3H).

^{13}C NMR (126 MHz, DMSO, TMS, ppm) δ 137.24, 134.71, 128.38, 127.50, 125.84, 124.85, 124.24, 120.90, 113.88, 112.22, 103.54, 39.46, 16.21.

1o: 5-iodo-3-(1-ethyl-1H-imidazol-5-yl)-1H-indole (AGH-107)

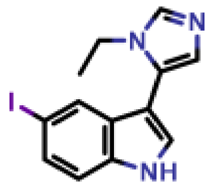

The compound was synthesized according to general procedure 1, purified via trituration with hexane:isopropanol 2:1 mixture. Off-white solid, 0.425 g, 1.26 mmol, 42% yield. Mp = 248°C. MS (m/z ESI+) = 337.81, LCMS t_R = 2.16 min.

^{1}H NMR (500 MHz, DMSO-d_6, TMS, ppm) δ 11.64 (s, 1H), 7.81 – 7.75 (m, 2H), 7.56 (d, *J* = 2.2 Hz, 1H), 7.42 (dd, *J* = 8.5, 1.7 Hz, 1H), 7.33 (dd, *J* = 8.5, 0.5 Hz, 1H), 7.00 (d, *J* = 1.1 Hz, 1H), 3.98 (q, *J* = 7.3 Hz, 2H), 1.20 (t, *J* = 7.2 Hz, 3H).

^{13}C NMR (126 MHz, DMSO, TMS, ppm) δ 137.23, 135.06, 129.67, 129.26, 127.53, 127.07, 125.35, 124.88, 114.32, 103.15, 83.42, 16.23.

1p: 3-(1-ethyl-1*H*-imidazol-5-yl)-1*H*-indole-5-carbonitrile (AGH-85)

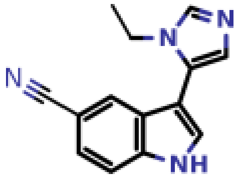

Aldehyde used as a substrate for this compound was synthesized according to general procedure 2 with an exception - no NaOH solution was added after quenching Vilsmeier adduct with water to avoid nitrile hydrolysis. The compound was synthesized according to general procedure 1, purified via trituration with hexane:isopropanol 2:1 mixture. Orange solid, 0.426 g, 1.8 mmol, 60% yield. Mp = 203°C. MS (m/z ESI+) = 237.10, LCMS t_R = 0.82 min.

^{1}H NMR (500 MHz, DMSO-d_6, TMS, ppm) δ 11.64 (s, 1H), 7.81 – 7.75 (m, 2H), 7.56 (d, *J* = 2.2 Hz, 1H), 7.42 (dd, *J* = 8.5, 1.7 Hz, 1H), 7.33 (dd, *J* = 8.5, 0.5 Hz, 1H), 7.00 (d, *J* = 1.1 Hz, 1H), 3.98 (q, *J* = 7.3 Hz, 2H), 1.20 (t, *J* = 7.2 Hz, 3H).

^{13}C NMR (126 MHz, DMSO, TMS, ppm) δ 137.76, 137.51, 127.86, 126.62, 126.24, 124.55, 124.51, 124.32, 120.48, 113.16, 105.00, 101.83, 39.57, 16.12.

1q: 3-(1-ethyl-1H-imidazol-5-yl)-5-methyl-1H-indole (AGH-108)

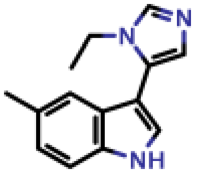

The compound was synthesized according to general procedure 1, purified via trituration with hexane:isopropanol 2:1 mixture. Pale yellow solid, 0.347 g, 1.54 mmol, 51% yield. Mp = 167°C. MS (m/z ESI+) = 225.97, LCMS t_R = 1.61 min.

^{1}H NMR (500 MHz, DMSO-d_6, TMS, ppm) δ 3.84 (s, 3H), 6.82 – 6.89 (m, 1H), 7.29 (d, J = 2.4 Hz, 1H), 7.39 (dd, J = 8.8, 0.5 Hz, 1H), 8.03 – 8.11 (m, 2H), 9.19 (d, J = 1.3 Hz, 1H), 11.60 – 11.64 (m, 1H), 14.87 (s, 2H).

^{13}C NMR (126 MHz, DMSO, TMS, ppm) δ 25.50, 55.65, 62.00, 101.04, 102.18, 112.42, 112.93, 124.15, 125.38, 128.28, 131.41, 133.23, 154.39.

1r: 3-(1-ethyl-1*H*-imidazol-5-yl)-1*H*-indol-5-ol (AGH-110)

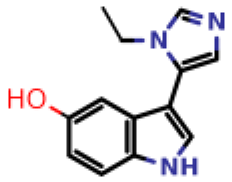

The compound was synthesized by hydrogenation of **1y** (**AGH-101**) according to general procedure 4 with the exception that the raw product was triturated under hexane:isoporopanol:acetone 3:1:1 mixture. Brown solid, 0.09 g, 0.39 mmol, 65% yield. Mp = 183°C. MS (m/z ESI+) = 228.16, LCMS t_R = 0.45 min.

^{1}H NMR (500 MHz, DMSO-d_6, TMS, ppm) δ 11.13 (s, 1H), 8.82 (s, 1H), 7.75 (s, 1H), 7.40 (d, J = 2.6 Hz, 1H), 7.24 (d, J = 8.6 Hz, 1H), 6.93 (s, 1H), 6.80 (d, J = 2.3 Hz, 1H), 6.67 (dd, J = 8.6, 2.3 Hz, 1H), 3.98 (q, J = 7.3 Hz, 2H), 1.22 (t, J = 7.2 Hz, 3H).

^{13}C NMR (126 MHz, DMSO, TMS, ppm) δ 151.26, 130.43, 127.31, 126.77, 124.47, 112.19, 112.01, 102.86, 102.55, 73.71, 39.40, 25.49, 16.27.

1s: 3-(1-benzyl-1*H*-imidazol-5-yl)-5-methoxy-1*H*-indole (AGH-58)

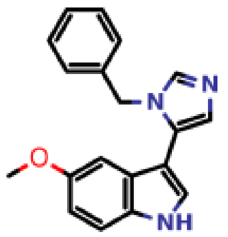

The compound was synthesized according to general procedure 1, purified via chromatography (SiO_2, AcOEt). White solid, 0.335 g, 1.1 mmol, 37% yield. MS (m/z ESI+) = 304.15, LCMS t_R = 1.86 min.

^{1}H NMR (500 MHz, DMSO-d_6, TMS, ppm) δ 11.21 (s, 1H), 7.83 (s, 1H), 7.35 – 7.24 (m, 3H), 7.26 – 7.18 (m, 2H), 7.14 (s, 1H), 7.02 – 6.95 (m, 2H), 6.93 (d, J = 2.4 Hz, 1H), 6.78 (dd, J = 8.7, 2.4 Hz, 1H), 5.27 (s, 2H), 3.70 (s, 3H), 1.92 (s, 1H).

^{13}C NMR (126 MHz, DMSO, TMS, ppm) δ 21.08, 39.01, 47.64, 55.18, 100.18, 103.36, 112.12, 112.53, 124.56, 126.33, 126.55, 126.77, 127.21, 127.29, 128.60, 130.97, 138.16, 138.21, 153.89.

1t: 3-(1-ethyl-1H-imidazol-5-yl)-4-methoxy-1H-indole (AGH-102)

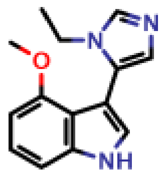

The compound was synthesized according to general procedure 1, purified via trituration with hexane:isopropanol 2:1 mixture. Pale orange solid, 0.334 g, 1.38 mmol, 46% yield. Mp = 197°C. MS (m/z ESI+) = 241.83, LCMS t_R = 1.34 min.

^{1}H NMR (500 MHz, DMSO-d_6, TMS, ppm) δ 11.38 (s, 1H), 7.69 (s, 1H), 7.23 (s, 1H), 7.09 – 7.01 (m, 2H), 6.75 (d, *J* = 1.2 Hz, 1H), 6.52 (dd, *J* = 5.7, 2.9 Hz, 1H), 3.84 (q, *J* = 7.3 Hz, 2H), 3.68 (s, 3H), 2.08 (s, 1H), 1.14 (t, *J* = 7.3 Hz, 3H).

^{13}C NMR (126 MHz, DMSO, TMS, ppm) δ 153.75, 138.39, 136.72, 128.02, 127.53, 125.20, 122.95, 117.08, 105.68, 103.09, 100.33, 55.46, 31.17, 16.30.

1u: 3-(1-ethyl-1H-imidazol-5-yl)-7-methyl-1H-indole (AGH-104)

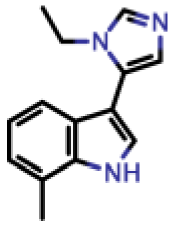

The compound was synthesized according to general procedure 1, purified via trituration with hexane:isopropanol 2:1 mixture. Beige solid, 0.322 g, 1.43 mmol, 47% yield. Mp = 171°C. MS (m/z ESI+) = 225.90, LCMS t_R= 1.85 min.

^{1}H NMR (500 MHz, DMSO-d_6, TMS, ppm) δ 11.40 (s, 1H), 7.76 (s, 1H), 7.50 (d, *J* = 2.6 Hz, 1H), 7.34 – 7.28 (m, 1H), 7.01 – 6.92 (m, 3H), 4.00 (q, *J* = 7.3 Hz, 2H), 2.50 (s, 3H), 1.21 (s, 3H).

^{13}C NMR (126 MHz, DMSO, TMS, ppm) δ 136.93, 135.53, 127.17, 126.21, 125.87, 123.85, 122.12, 120.98, 119.75, 116.43, 104.26, 39.46, 16.78, 16.22.

1v: 3-(1-ethyl-1H-imidazol-5-yl)-5-methoxy-2-methyl-1H-indole (AGH-89)

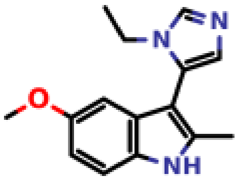

The compound was synthesized according to general procedure 1, purified via chropamography (SiO_2, AcOEt:MeOH 49:1). Pale brown solid, 0.342 g, 1.34 mmol, 45% yield. Mp = 168-169°C. MS (m/z ESI+) = 225.89, LCMS t_R = 1.22 min.

^{1}H NMR (500 MHz, DMSO-d_6, TMS, ppm) δ 11.16 (s, 1H), 7.81 (d, *J* = 1.1 Hz, 1H), 7.23 (dd, *J* = 8.7, 0.6 Hz, 1H), 6.82 (d, *J* = 1.2 Hz, 1H), 6.69 (dd, *J* = 8.7, 2.5 Hz, 1H), 6.59 (d, *J* = 2.4 Hz, 1H), 3.78 (q, *J* = 7.2 Hz, 2H), 3.68 (s, 3H), 2.26 (s, 3H), 1.12 (t, *J* = 7.3 Hz, 3H).

^{13}C NMR (126 MHz, DMSO, TMS, ppm) δ 153.70, 137.10, 135.91, 130.20, 128.79, 128.32, 125.19, 111.60, 110.42, 100.44, 99.56, 55.22, 39.30, 16.37, 11.90.

1w: 5-(1-ethyl-1H-imidazol-5-yl)-1H-indole (AGH-96)

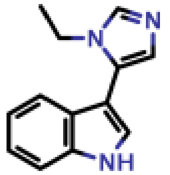

The compound was synthesized according to general procedure 1, purified via flash chromatography (SiO_2, DCM). Pale orange solid, 0.951 g, 4.5 mmol, 88% yield. Mp = 124°C. MS (m/z ESI+) = 211.90, LCMS t_R = 0.49-0.79 min.

^{1}H NMR (500 MHz, DMSO-d_6, TMS, ppm) δ 11.52 (s, 1H), 7.81 (d, *J* = 1.1 Hz, 1H), 7.58 – 7.46 (m, 3H), 7.18 (ddd, *J* = 8.1, 6.9, 1.1 Hz, 1H), 7.12 – 7.02 (m, 2H), 4.03 (q, *J* = 7.2 Hz, 2H), 1.24 (t, *J* = 7.3 Hz, 3H).

^{13}C NMR (126 MHz, DMSO, TMS, ppm) δ 137.45, 136.54, 127.60, 126.96, 126.27, 124.63, 122.15, 120.05, 119.98, 119.33, 112.30, 104.28, 16.70.

1x: 3-(1H-imidazol-5-yl)-5-methoxy-1H-indole (AGH-117)

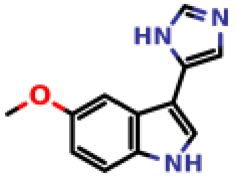

The compound was synthesized by hydrogenation of **AGH-58** (**1s**) according to general procedure 4 with the exception that the raw product was triturated under hexane:isoporopanol:acetone 3:1:1 mixture. Beige solid, 0.12 g, 0.56 mmol, 95% yield. Mp = 234°C. MS (m/z ESI+) = 213.9, LCMS t_R = 0.46-0.94 min.

^{1}H NMR (500 MHz, DMSO-d_6, TMS, ppm) δ 1.21 (t, *J* = 7.2 Hz, 3H), 2.37 (t, *J* = 0.6 Hz, 3H), 3.99 (q, *J* = 7.2 Hz, 2H), 6.94 – 7.02 (m, 2H), 7.27 (dq, *J* = 1.6, 0.8 Hz, 1H), 7.33 (dd, *J* = 8.2, 0.8 Hz, 1H), 7.46 (d, *J* = 2.6 Hz, 1H), 7.75 (d, *J* = 1.1 Hz, 1H), 11.27 – 11.31 (m, 1H).

^{13}C NMR (126 MHz, DMSO, TMS, ppm) δ 16.25, 21.23, 103.27, 111.47, 118.39, 119.50, 123.25, 124.15, 125.82, 126.76, 127.16, 128.11, 134.36, 136.87.

1y: 5-(benzyloxy)-3-(1-ethyl-1H-imidazol-5-yl)-1H-indole (AGH-101)

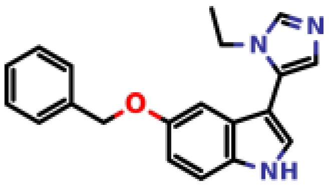

The compound was synthesized according to general procedure 1, except for the fact that it participated out of solution during reaction and the reaction mixture was vacuum filtered instead of being extracted. Obtained solid was washed with 2*5 ml cold MeOH. White solid, 2.73 g, 8.6 mmol, 85% yield. Mp = 191°C. MS (m/z ESI+) = 318.01, LCMS t_R = 2.29 min.

^{1}H NMR (500 MHz, DMSO-d$_6$, TMS, ppm) δ 11.32 – 11.27 (m, 1H), 7.75 (d, *J* = 1.1 Hz, 1H), 7.52 – 7.46 (m, 2H), 7.46 – 7.41 (m, 1H), 7.43 – 7.34 (m, 3H), 7.34 – 7.26 (m, 1H), 7.01 (d, *J* = 2.4 Hz, 1H), 6.97 (d, *J* = 1.1 Hz, 1H), 6.89 (dd, *J* = 8.7, 2.4 Hz, 1H), 5.09 (s, 2H), 3.96 (q, *J* = 7.2 Hz, 2H), 1.20 (t, *J* = 7.2 Hz, 3H).

^{13}C NMR (126 MHz, DMSO, TMS, ppm) δ 16.20, 39.01, 69.62, 101.89, 103.64, 112.58, 112.68, 124.76, 125.82, 126.75, 126.76, 127.01, 127.48, 127.54, 128.30, 131.21, 136.88, 137.72, 152.82, 152.82.

1z: 3-(1-ethyl-1H-imidazol-5-yl)-7-fluoro-5-iodo-1H-indole (AGH-116)

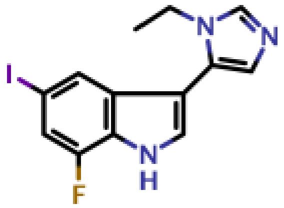

The compound was synthesized according to general procedure 1, purified via trituration with hexane: isopropanol:acetone 10:5:1 mixture. White solid, 0.23 g, 0.65 mmol, 75% yield. Mp = 245°C. MS (m/z ESI+) = 356.0, LCMS t_R = 2.19 min.

^{1}H NMR (500 MHz, DMSO-*d*$_6$, TMS, ppm) δ 12.19 (s, 1H), 7.79 (d, *J* = 1.1 Hz, 1H), 7.64 (s, 1H), 7.60 (d, *J* = 1.3 Hz, 1H), 7.34 (dd, *J* = 10.4, 1.3 Hz, 1H), 7.02 (d, *J* = 1.1 Hz, 1H), 3.97 (q, *J* = 7.3 Hz, 2H), 1.19 (t, 3H).

^{13}C NMR (126 MHz, DMSO, TMS, ppm) δ 149.99, 148.00, 137.46, 132.51, 127.87, 126.40, 123.59, 123.50, 115.03, 104.23, 81.44, 39.48, 16.15.

1za: 3-(1-ethyl-1H-imidazol-5-yl)-1H-indole-5-carboxamide (AH-494)

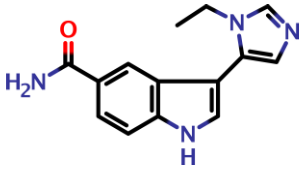

The compound was synthesized according to a modified procedure outlined by Agarwal et. al. The product was purified by trituration with acetone. Pale yellow, 0.08 g, 0.3 mmol, 9% yield. Mp = 228°C. MS (m/z ESI+) = 255.16, LCMS t_R = 0.5 min.

^{1}H NMR (500 MHz, DMSO-d_6, TMS, ppm) δ 1.22 (t, J = 7.2 Hz, 2H), 2.08 (s, 3H), 4.02 (q, J = 7.2 Hz, 1H), 7.10 (d, J = 7.9 Hz, 1H), 7.47 (dd, J = 8.5, 0.6 Hz, 1H), 7.61 (d, J = 1.7 Hz, 1H), 7.74 (dd, J = 8.6, 1.7 Hz, 1H), 7.79 (s, 1H), 7.95 (s, 1H), 8.11 (d, J = 1.7 Hz, 1H), 11.62 – 11.66 (m, 1H).

^{13}C NMR (126 MHz, DMSO, TMS, ppm) δ 16.20, 30.70, 105.05, 111.22, 119.09, 121.67, 125.20, 125.24, 125.90, 125.99, 127.69, 137.17, 137.59, 168.84.

2a: 5-(4-methoxyphenyl)-1-methyl-4-nitro-1H-imidazole (AGH-54)

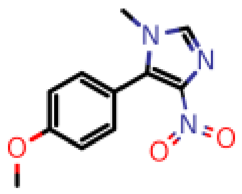

The compound was synthesized according to Tiwari et al. Pale yellow solid, 0.095 g, 0.4 mmol, 12% yield. Mp = 171°C. MS (m/z ESI+) = 234.10, LCMS t_R = 2.17 min.

^{1}H NMR (500 MHz, DMSO-d_6) δ 3.50 (s, 3H), 3.83 (s, 3H), 7.05 – 7.12 (m, 2H), 7.43 – 7.50 (m, 2H), 7.91 (s, 1H).

^{13}C NMR (126 MHz, DMSO, TMS, ppm) δ 32.82, 55.27, 113.95, 118.59, 128.69, 128.79, 131.75, 132.77, 136.75, 143.35, 160.19.

Elemental analysis: found C: 56.82%, H: 4.63%, N: 17.39% $C_{11}H_{11}N_3O_3$ requires C:56.65%, H: 4.75%, N: 18.02%.

2b: 5-(4-methoxyphenyl)-1-methyl-1H-imidazole (AH-445)

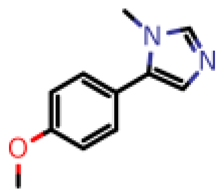

The compound was synthesized according to general procedure 1, purified via trituration with hexane:isopropanol 2:1 mixture. White solid, 0.04 g, 0.2 mmol, 5% yield. Mp = 123°C. MS (m/z ESI+) = 189.11, LCMS t_R = 0.5 - 0.7 min.

^{1}H NMR (500 MHz, DMSO-d_6, TMS, ppm) δ 7.66 (s, 1H), 7.40 (d, 2H), 7.02 (d, 2H), 6.95 (s, 1H), 3.79 (s, 3H), 3.63 (s, 3H).

^{13}C NMR (126 MHz, DMSO, TMS, ppm) δ 32.15, 55.16, 114.20, 122.06, 126.63, 129.32, 132.44, 139.10, 158.77.

Elemental analysis: found C: 70.11%, H: 6.42%, N: 14.88% $C_{11}H_{12}N_2O$ requires C:70.19%, H: 6.43%, N: 14.88%.

2c: 5-(3-methoxyphenyl)-1-methyl-1H-imidazole (AGH-47)

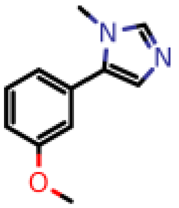

The compound was synthesized according to general procedure 1, purified via column chromatography (SiO_2, AcOEt). Yellow oil, 0.065 g, 0.35 mmol, 9% yield. MS (m/z ESI+) = 188.98, LCMS t_R = 0.5 - 0.67 min.

^{1}H NMR (500 MHz, DMSO-d$_6$, TMS, ppm) δ 7.70 (s, 1H), 7.36 (t, 1H), 7.08 – 7.00 (m, 3H), 6.94 (dd, *J* = 8.3, 2.6, 0.9 Hz, 1H), 3.80 (s, 3H), 3.68 (s, 3H).

^{13}C NMR (126 MHz, DMSO, TMS, ppm) δ 32.40, 55.14, 113.10, 113.25, 120.03, 127.60, 129.84, 131.03, 131.47, 132.48, 159.41.

2d: 5-(2-methoxyphenyl)-1-methyl-1*H*-imidazole (AH-444)

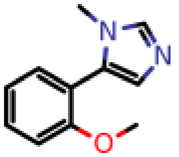

The compound was synthesized according to general procedure 1, purified via column chromatography (SiO_2, $CHCl_3$). Yellow oil, 0.03 g, 0.16 mmol, 4% yield. MS (m/z ESI+) = 189.04, LCMS t_R = 0.5 - 0.69 min.

^{1}H NMR (500 MHz, DMSO-*d*$_6$, TMS, ppm) δ 7.67 (s, 1H), 7.42 (ddd, *J* = 8.3, 7.4, 1.8 Hz, 1H), 7.23 (dd, *J* = 7.4, 1.7 Hz, 1H), 7.12 (dd, *J* = 8.4, 1.0 Hz, 1H), 7.01 (td, *J* = 7.4, 1.0 Hz, 1H), 6.85 (d, *J* = 1.2 Hz, 1H), 3.78 (s, 3H), 3.44 (d, *J* = 0.5 Hz, 3H).

^{13}C NMR (126 MHz, DMSO, TMS, ppm) δ 156.85, 138.56, 131.61, 130.10, 127.84, 120.52, 120.49, 118.40, 111.39, 55.29, 31.63.

2e: 1-methyl-5-phenyl-1*H*-imidazole (AH-441)

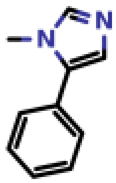

The compound was synthesized according to general procedure 1, purified via trituration with diethyl ether:acetone 20:1 mixture. Yellow solid, 0.03 g, 0.19 mmol, 5% yield. Mp = 93°C. MS (m/z ESI+) = 159.05, LCMS t_R = 0.5 - 0.6 min.

^{1}H NMR (500 MHz, DMSO-d_6, TMS, ppm) δ 7.70 (s, 1H), 7.53 – 7.41 (m, 4H), 7.40 – 7.33 (m, 1H), 7.05 (s, 1H), 3.68 (s, 3H).

^{13}C NMR (126 MHz, DMSO, TMS, ppm) δ 32.34, 127.45, 127.46, 127.48, 127.76, 128.74, 128.75, 129.78, 132.58, 139.76.

Elemental analysis: found C: 75.7%, H: 6.33%, N: 17.71% $C_{10}H_{10}N_2$ requires C:75.92%, H: 6.37%, N: 17.71%.

2f: 5-(4-iodo-2,5-dimethoxyphenyl)-1-methyl-1*H*-imidazole (AH-438)

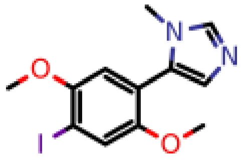

The compound was synthesized according to general procedure 1, purified via flash chromatography (SiO_2, AcOEt). Pale bown solid, 0.08 g, 0.23 mmol, 17% yield. Mp = 120°C. MS (m/z ESI+) = 345.07, LCMS t_R = 1.78 min.

^{1}H NMR (500 MHz, DMSO-d_6, TMS, ppm) δ 7.69 (d, *J* = 1.1 Hz, 1H), 7.47 (s, 1H), 6.93 (d, *J* = 1.1 Hz, 1H), 6.87 (s, 1H), 3.76 (d, *J* = 23.9 Hz, 6H), 3.47 (s, 3H).

^{13}C NMR (126 MHz, DMSO, TMS, ppm) δ 31.78, 56.12, 56.89, 86.47, 114.37, 119.23, 122.00, 128.20, 129.14, 138.83, 151.30, 152.00.

3a: 1-methyl-5-(1*H*-pyrrol-3-yl)-1*H*-imidazole (AH-426)

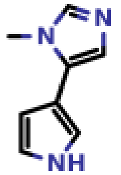

The compound was synthesized according to general procedure 1, purified via column chromatography (SiO_2, AcOEt). Brown solid, 0.125 g, 0.85 mmol, 21% yield. Decomposition upon heating. MS (m/z ESI+) = 148.12, LCMS t_R = 0.5 min.

^{1}H NMR (500 MHz, DMSO-d$_6$, TMS, ppm) δ 11.12 (s, 1H), 7.59 (d, *J* = 1.4 Hz, 1H), 7.02 (d, *J* = 1.2 Hz, 1H), 6.85 (td, *J* = 2.7, 1.5 Hz, 1H), 6.24 (ddd, *J* = 3.8, 2.5, 1.5 Hz, 1H), 6.13 (dt, *J* = 3.4, 2.5 Hz, 1H), 3.66 (s, 3H).

^{13}C NMR (126 MHz, DMSO, TMS, ppm) δ 32.33, 106.59, 108.52, 118.66, 120.48, 125.62, 126.47, 138.37.

3b: 2-(1-methyl-1*H*-imidazol-5-yl)-1*H*-indole (AGH-51)

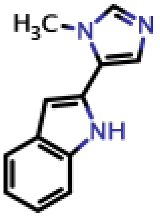

The compound was synthesized according to general procedure 1, purified via column chromatography (SiO_2, $CHCl_3$:MeOH 19:1). Brown solid, 0.05 g, 0.25 mmol, 10% yield. Mp = 172°C. MS (m/z ESI+) = 198.04, LCMS t_R = 1.07 min.

^{1}H NMR (500 MHz, DMSO-d$_6$, TMS, ppm) δ 11.34 (s, 1H), 7.76 – 7.71 (m, 1H), 7.54 (ddt, *J* = 7.8, 1.3, 0.8 Hz, 1H), 7.40 – 7.30 (m, 2H), 7.10 (ddd, *J* = 8.1, 7.0, 1.2 Hz, 1H), 7.00 (ddd, *J* = 7.9, 7.1, 1.0 Hz, 1H), 6.66 (dd, *J* = 2.2, 0.9 Hz, 1H), 3.83 (d, *J* = 0.5 Hz, 3H).

^{13}C NMR (126 MHz, DMSO, TMS, ppm) δ 32.92, 39.00, 40.01, 98.99, 110.93, 119.28, 119.87, 121.58, 125.77, 127.57, 127.63, 128.42, 136.29, 139.92.

3c: 5-(1-ethyl-1*H*-imidazol-5-yl)-1*H*-indole (AGH-98)

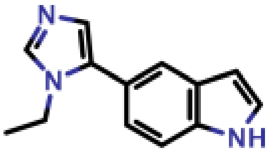

The compound was synthesized according to general procedure 1, purified via flash chromatography (SiO_2, DCM:MeOH 49:1). Dark orange oil which crystallized upon the storage, 0.193 g, 0.9 mmol, 23% yield. MS (m/z ESI+) = 211.9, LCMS t_R = 0.49 - 0.79 min.

^{1}H NMR (500 MHz, DMSO-*d*$_6$, TMS, ppm) δ 11.27 (s, 1H), 7.75 (s, 1H), 7.60 (s, 1H), 7.56 – 7.32 (m, 3H), 7.21 – 7.12 (m, 1H), 6.92 (d, *J* = 1.2 Hz, 1H), 6.50 (s, 1H), 4.11 – 3.94 (m, 3H), 1.21 (t, *J* = 7.2 Hz, 4H).

^{13}C NMR (126 MHz, DMSO, TMS, ppm) δ 137.25, 135.36, 133.52, 127.41, 126.70, 126.17, 121.87, 120.58, 120.08, 111.63, 101.33, 39.43, 16.26.

3d: 6-(1-ethyl-1*H*-imidazol-5-yl)-1*H*-indole (AGH-99)

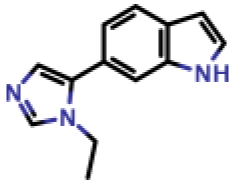

The compound was synthesized according to general procedure 1, purified via flash chromatography (SiO_2, DCM:MeOH 19:1). Dark red solid which crystalized upon the storage, 0.17 g, 0.8 mmol, 20% yield. MS (m/z ESI+) = 211.77, LCMS t_R = 1.46 min.

^{1}H NMR (500 MHz, DMSO-d_6, TMS, ppm) δ 11.20 (s, 1H), 7.75 (d, *J* = 1.1 Hz, 1H), 7.61 (dd, *J* = 8.1, 0.8 Hz, 1H), 7.46 – 7.37 (m, 2H), 7.05 (dd, *J* = 8.1, 1.5 Hz, 1H), 6.93 (d, *J* = 1.2 Hz, 1H), 6.47 (ddd, *J* = 2.9, 1.9, 0.9 Hz, 1H), 4.05 (q, *J* = 7.2 Hz, 1H), 1.22 (t, *J* = 7.2 Hz, 3H).

^{13}C NMR (126 MHz, DMSO, TMS, ppm) δ 16.33, 39.01, 101.06, 111.21, 119.64, 120.25, 122.56, 126.28, 126.90, 127.17, 133.35, 135.92, 137.58.

III. Cell culture and transfection

The full lenght human $5HTR_{1A}$, $5HTR_{6,}$ $5HTR_{7B}$ and DRD2 cDNAs cloned into mammalian expression vector pcDNA3.1(+) were obtained from the cDNA Resource Center (www.cdna.org). The cDNAs were stably transfected into human embryonic kidney cells (HEK293, ATCC) with use of Lipofectamine 2000 (Invitrogen). G418-resistant clones yielding the highest expression level of $5HTR_{1A}$, $5HTR_{6}$, $5HTR_{7B}$ or DRD2 were selected during preliminary experiments including RT-PCR and Western Blot analysis as well as ligand binding and cAMP accumulation assays. CHO-K1 cells expressing $5HTR_{2A}$ were purchased from PerkinElmer BioSignal.

All cell lines were maintained at 37°C in a humidified atmosphere with 5% CO_2 and were grown in Dulbeco's Modifier Eagle Medium (Lonza) containing 10% dialysed foetal bovine serum (Biowest) and 500 μg/ml G418 sulphate (Lonza).

For cAMP accumulation assays, cells were subcultured in T75 flasks and grown to 90% confluence. 2 hours prior to the assay, cells were washed and incubated in serum-free DMEM. Cells were then collected in PBS containing 0.1 mM EDTA.

IV. Pharmacological data for compound 2a (AGH-54)

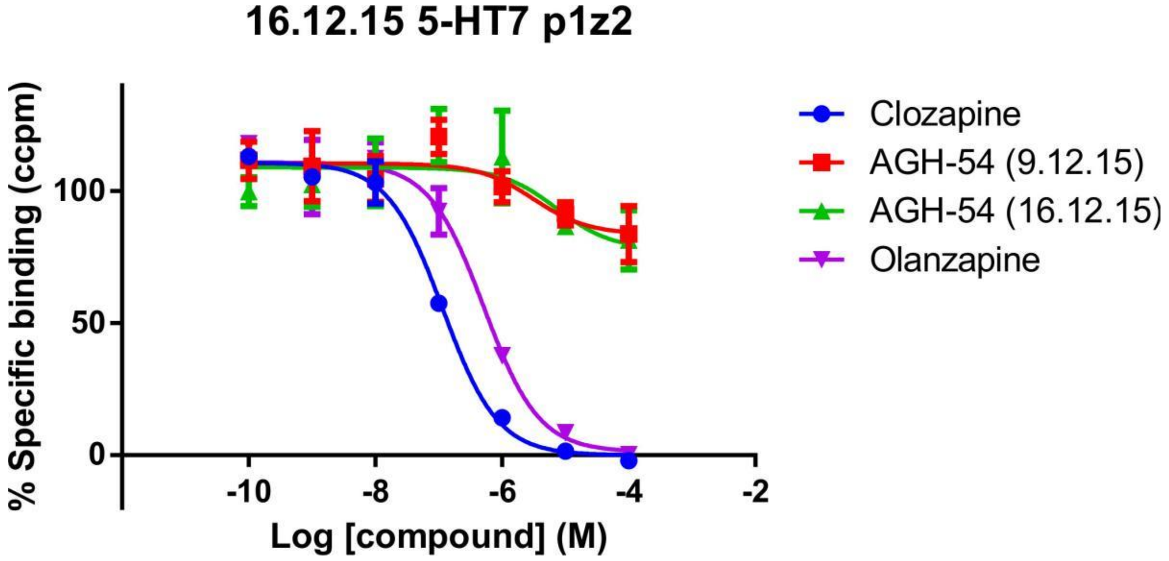

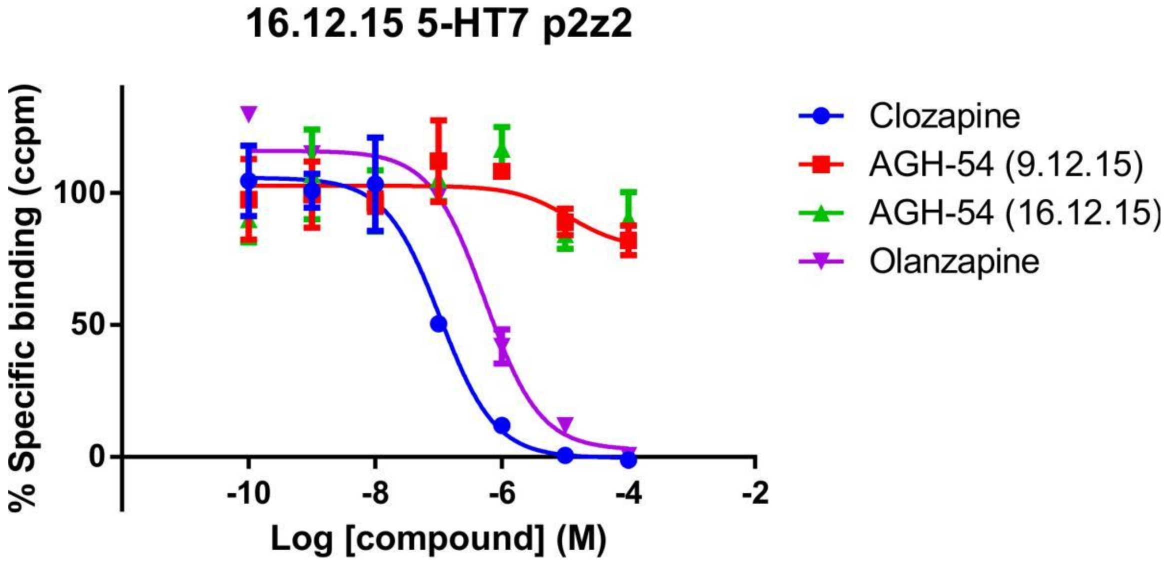

V. Metabolic stability assays for compound 1e (AGH-44) and 1o (AGH-107)

The *in vitro* evaluation of metabolic stability was performed by using human liver microsomes (HLMs) (Sigma-Aldrich, St. Louis, MO, USA). The reaction mixtures consisted of 50 µM of tested compounds with microsomes (1 mg/ml) in 10 mM tris-HCl buffer. After 5 min (37°C) preincubation, the 50 µL of NADPH Regeneration System (Promega, Madison, WI, USA) was added to initiate the reaction. The reactions were terminated by the addition of the cold methanol containing internal standard (IS) at different time points (5, 15, 30, 45 min). The additional reaction terminated after 120 min was also performed for compound **1o**. The mixtures were centrifuged 14000 rpm for 15 min before the analysis. The course of reaction was followed by using the analyte/IS peak height ratio values.

Mass spectra were recorded on a UPLC-MS/MS system consisted of a Waters ACQUITY® UPLC® (Waters Corporation, Milford, MA, USA) coupled to a Waters TQD mass spectrometer (electrospray ionization mode ESI-tandem quadrupole). Chromatographic separations were carried out using the Acquity UPLC BEH (bridged ethyl hybrid) C18 column; 2.1 × 100 mm, and 1.7 µm particle size, equipped with Acquity UPLC BEH C18 VanGuard pre-column; 2.1 × 5 mm, and 1.7 µm particle size. The column was maintained at 40 °C, and eluted under gradient conditions from 95% to 0% of eluent A over 10 min, at a flow rate of 0.3 mL min^{-1}. Eluent A: water/formic acid (0.1%, v/v); eluent B: acetonitrile/formic acid (0.1%, v/v). Chromatograms were made using Waters eλ PDA detector. Spectra were analyzed in 200–700 nm range with 1.2 nm resolution and sampling rate 20 points/s. MS detection settings of Waters TQD mass spectrometer were as follows: source temperature 150 °C, desolvation temperature 350 °C, desolvation gas flow rate 600 L h^{-1}, cone gas flow 100 L h^{-1}, capillary potential 3.00 kV, cone potential 40 V. Nitrogen was used for both nebulizing and drying gas. The data were obtained in a scan mode ranging from 50 to 2000 m/z in time 1.0 s intervals. Data acquisition software was MassLynx V 4.1 (Waters). The UPLC/MS purity of all the final compounds was confirmed to be 95% or higher.

<u>Compound 1e</u>

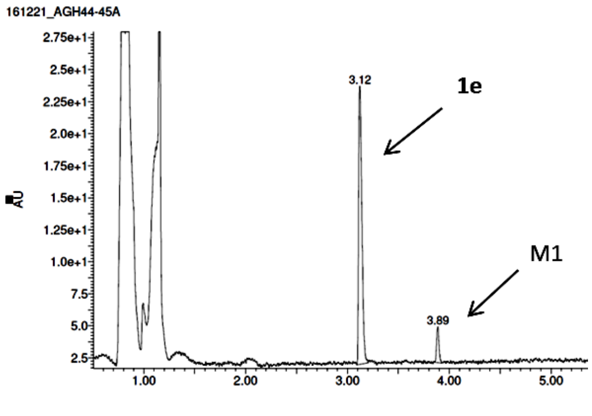

Fig. 1 The UPLC spectrum after 45 min reaction of **1e** with HLMs.

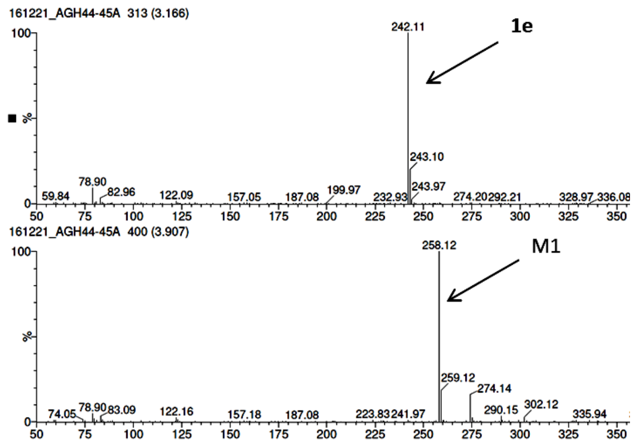

Fig. 2 MS spectra of compound **1e** and its two metabolites. The molecular mass of obtained metabolite **M1** suggests hydroxylation as the main metabolic pathway of **1e**.

The determination of 1e Intrinsic Clearance CL_{int}

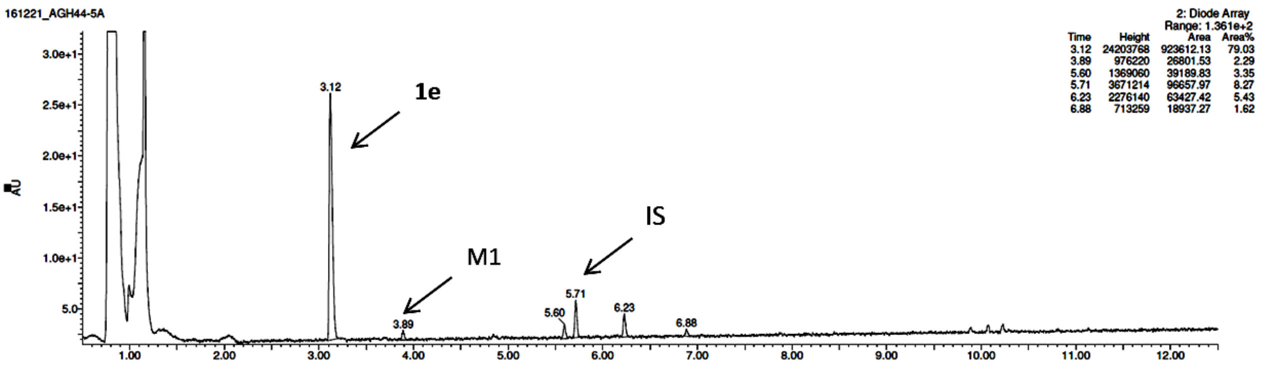

Fig. 3 The UPLC spectrum after 5 min reaction of **1e** with HLMs. IS = internal standard.

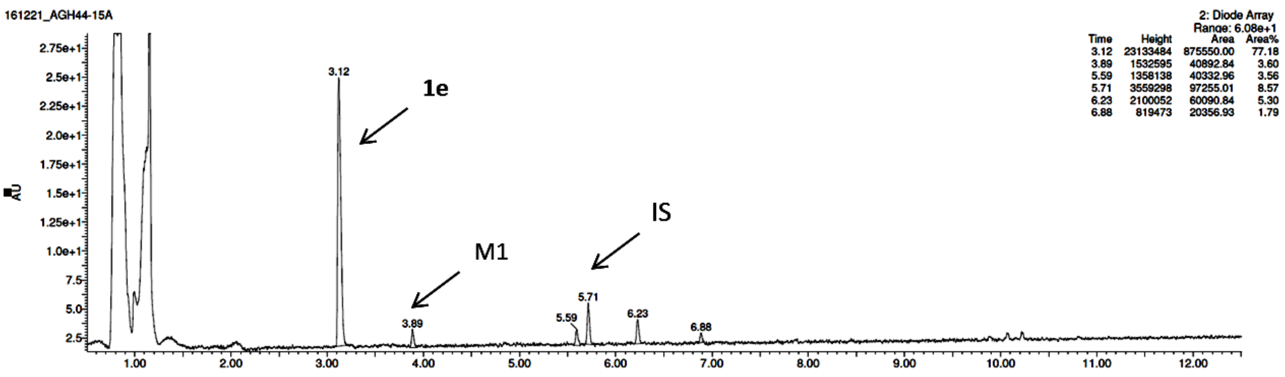

Fig. 4 The UPLC spectrum after 15 min reaction of **1e.** IS = internal standard.

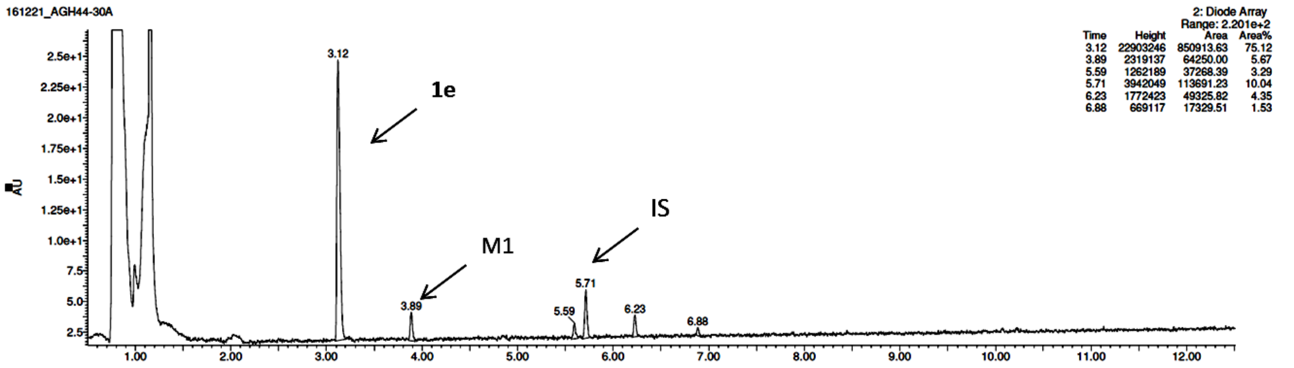

Fig. 5 The UPLC spectrum after 30 min reaction of **1e** with HLMs. IS = internal standard.

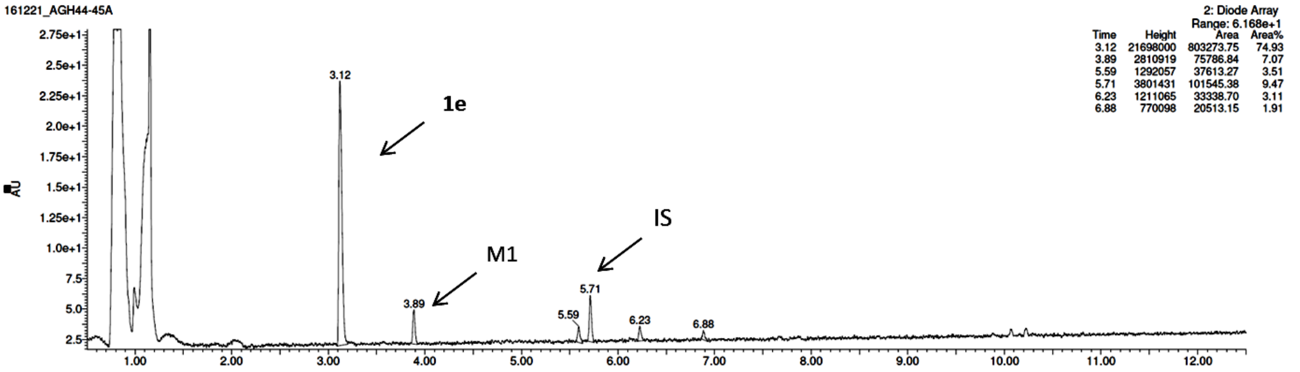

Fig. 6 The UPLC spectrum after 45 min reaction of **1e** with HLMs. IS = internal standard.

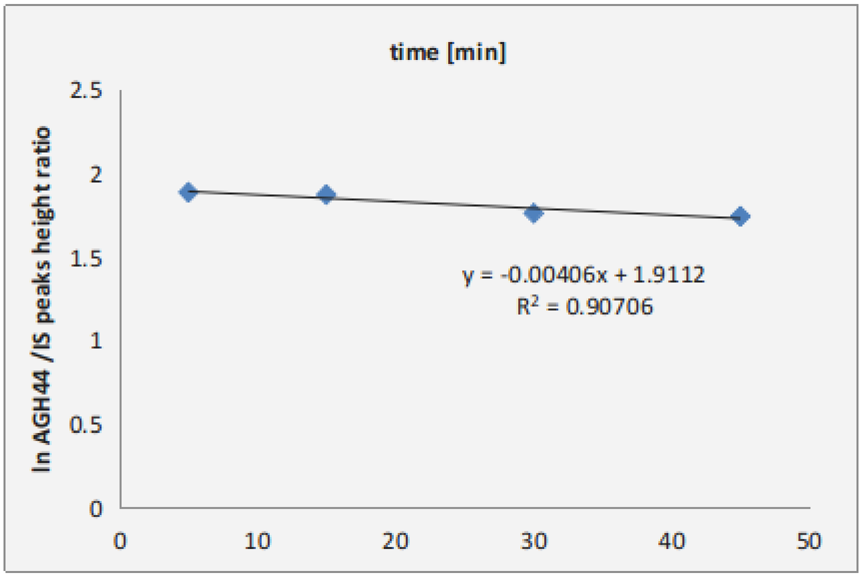

Fig. 7 disappearance of **1e** in the presence of HLMs

$$CL_{int} = \frac{-0.693}{in\ vitro\ t_{1/2}} \times \frac{ml\ incubation}{mg\ microsomes} \times \frac{45\ mg\ microsomes}{gm\ liver} \times \frac{20\ gm\ liver}{kg\ b.w.}$$

Cl_{int} = 3,69 ml/min/kg

Compound 1o

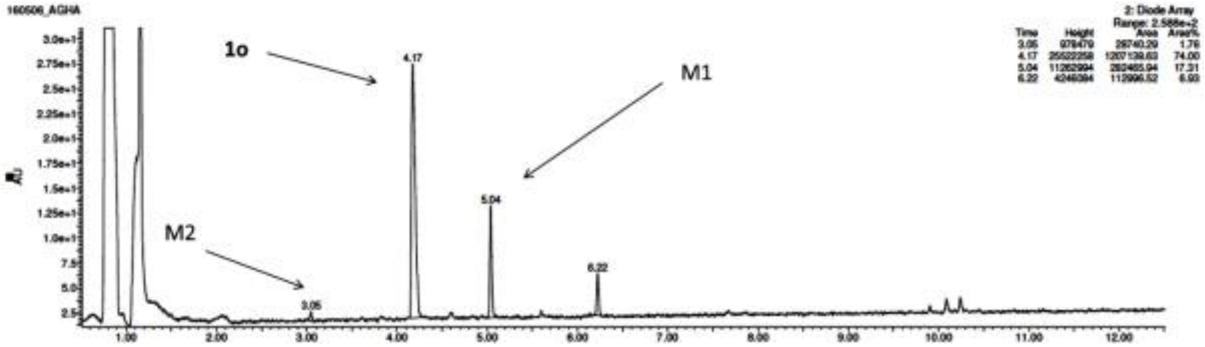

Fig. 8 The UPLC spectrum after 120 min. reaction of **1o** with HLMs.

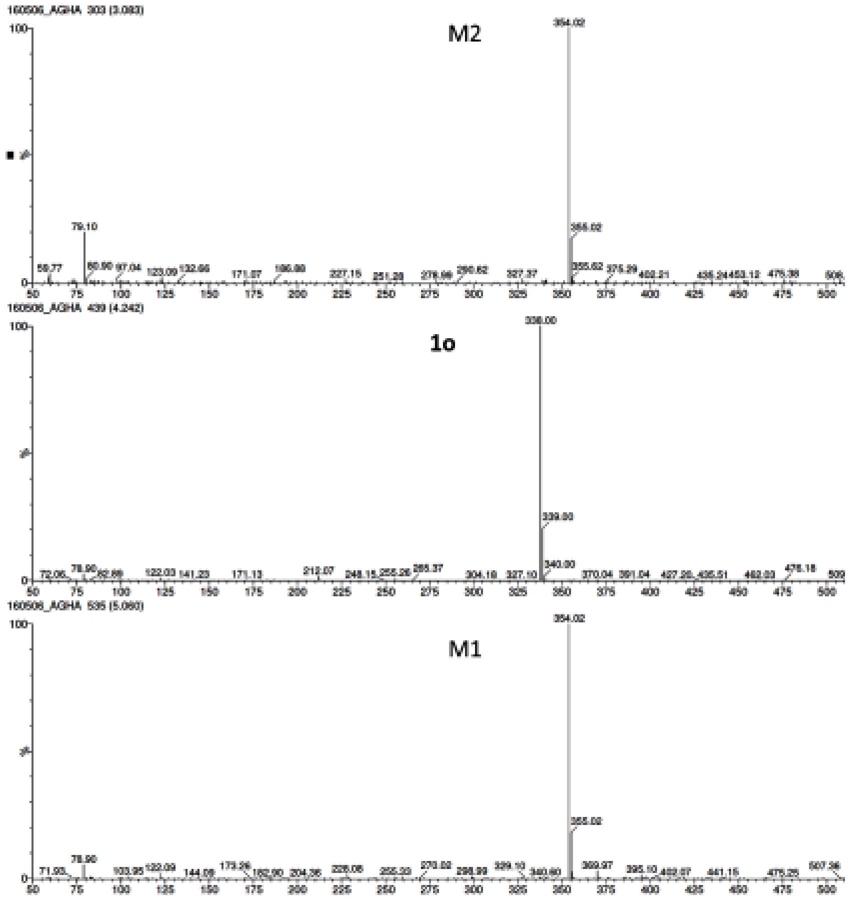

Fig.9 The molecular mass of obtained metabolite M1 suggest the hydroxylation as a main metabolic pathway of **1o**.

The determination of 1o Intrinsic Clearance CLint

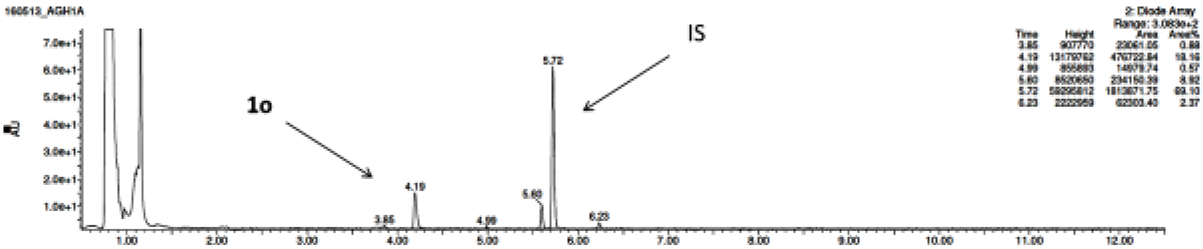

Fig.10 The UPLC spectrum after 0 min reaction of **1o** with HLMs. IS = internal standard.

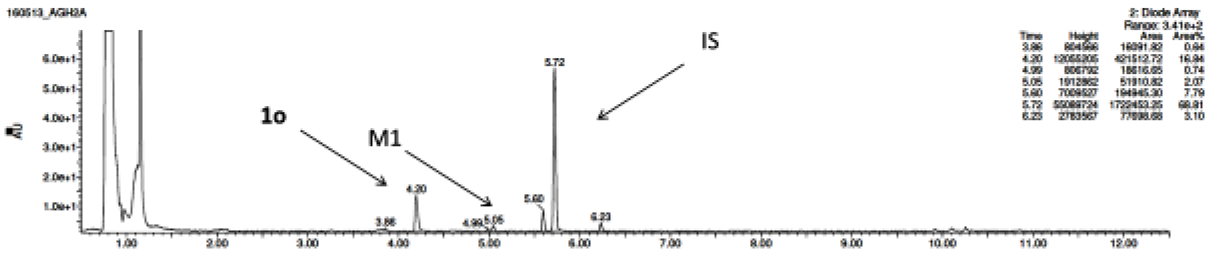

Fig.11 The UPLC spectrum after 15 min reaction of **1o** with HLMs. IS = internal standard.

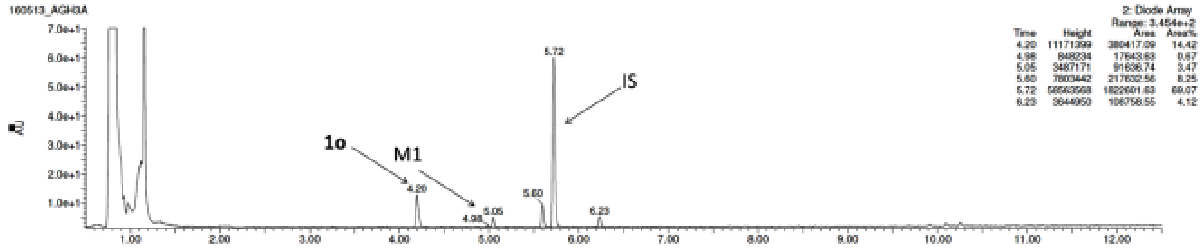

Fig.12 The UPLC spectrum after 30 min reaction of **1o** with HLMs. IS = internal standard.

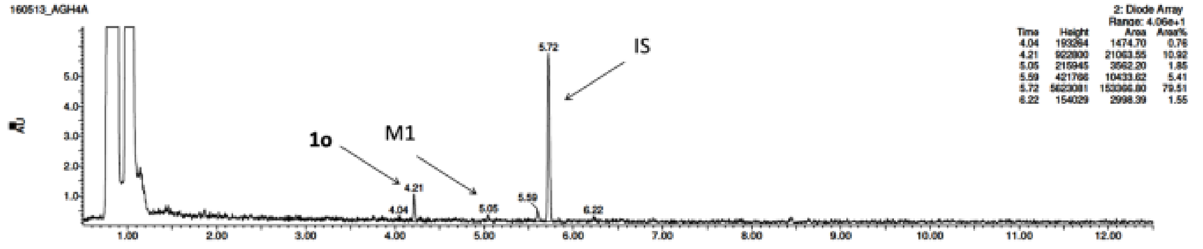

Fig.13 The UPLC spectrum after 45 min reaction of **1o** with HLMs.

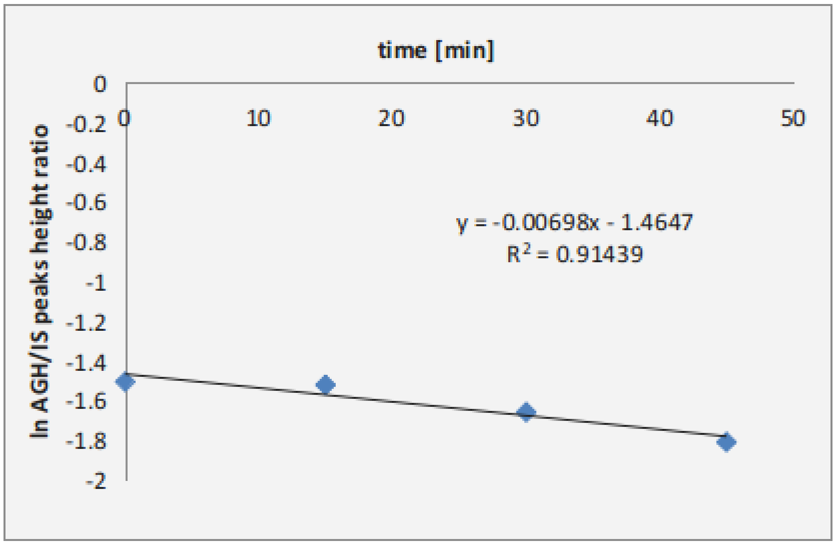

Fig.14 The disappearance of **1o** in the presence of HLMs

$$CL_{int} = \frac{-0.693}{in\ vitro\ t_{1/2}} \times \frac{ml\ incubation}{mg\ microsomes} \times \frac{45\ mg\ microsomes}{gm\ liver} \times \frac{20\ gm\ liver}{kg\ b.w.}$$

$CL_{int\ (\mathbf{1o})}$ = 6.3 ml/min/kg

VI. Cytotoxicity assay and CYP3A4 binding for compounds 1e and 1o

Methods

Cell lines

Human embryonic kidney HEK-293 cell line (ATCC CRL-1573) was kindly donated by Prof. Dr. Christa Müller (Pharmaceutical Institute, Pharmaceutical Chemistry I, University of Bonn).

The cell line was cultured in Dulbecco's Modified Eagle's Medium DMEM (Gibco) with 10% fetal bovine serum (FBS), 100 mg/ml streptomycin and 100 U/ml penicillin at 37°C in an atmosphere containing 5% of CO_2.

Hepatoma HepG2 (ATCC HB-8065) cell line was kindly donated by the Department of Pharmacological Screening, Jagiellonian University Medical College. The cells were cultured in Modified Eagle's Medium MEM with 100 mg/ml streptomycin and 100 U/ml penicillin, 1%, Non Essential Amino Acids (NEAA) and 10% FBS (Gibco) at 37°C in an atmosphere containing 5% of CO_2.

MTS assay

The HEK-293 and HepG2 cells were seeded in 96-well plates at a concentration of 1×10^4 cells/well in 200 µl of respective culture medium and cultured for 24 h to reach 50 % confluence. Next, the 10 mM stock solutions of examined compounds in DMSO were diluted into fresh growth medium and added into the microplates at the final concentrations 0.1 µM – 100 µM. The well-known cytostatic compound doxorubicin (DX) 1 µM was used as a positive control for both cell lines. Additionally, carbonyl cyanide 3-chlorophenylhydrazone (CCCP) 10 µM was used as positive control mitochondrial toxin for hepatocytes. After 72 h of incubation, 20 µl of MTS labeling mixture (CellTiter 96® AQueous One Solution Cell Proliferation Assay, Promega) was added to the each well and the cells were incubated under the same conditions for 4 h. The absorbance of the samples was measured using a microplate reader (PerkinElmer) at 490 nm. Statistical significance was analyzed by GraphPad Prism™ software (version 5.01, San Diego, CA, USA) using One-way ANOVA and Bonferroni's Multiple Comparison Post Test.

Drug-drug interactions (DDI) – the influence on CYP3A4 activity

The luminescent CYP3A4 P450-Glo™ assay and protocol were provided by Promega (Madison, WI, USA) [J.J Cali et al., Expert Opin Drug Metab Toxicol, 2 (2006), 629–645].The reference drug ketoconazole (KE) was obtained from Sigma-Aldrich (St. Louis, USA). The enzymatic reactions were performed in white polystyrene, flat-bottom Nunc™ MicroWell™ 96-Well Microplates (Thermo Scientific, Waltham, MA USA). The luminescence signal was measured with a microplate reader in luminescence mode (EnSpire, PerkinElmer, Waltham, MA USA). The IC_{50} value of KE was determinate and calculated as reported previously [D. Łażewska et al, Eur J Med Chem. (2014), 838, 534–546]. The final concentrations of **1e** and **1o** were from 0.1 µM to 25 µM. GraphPad Prism™ software (version 5.01, San Diego, CA, USA) was used to calculate IC_{50} values.

Results

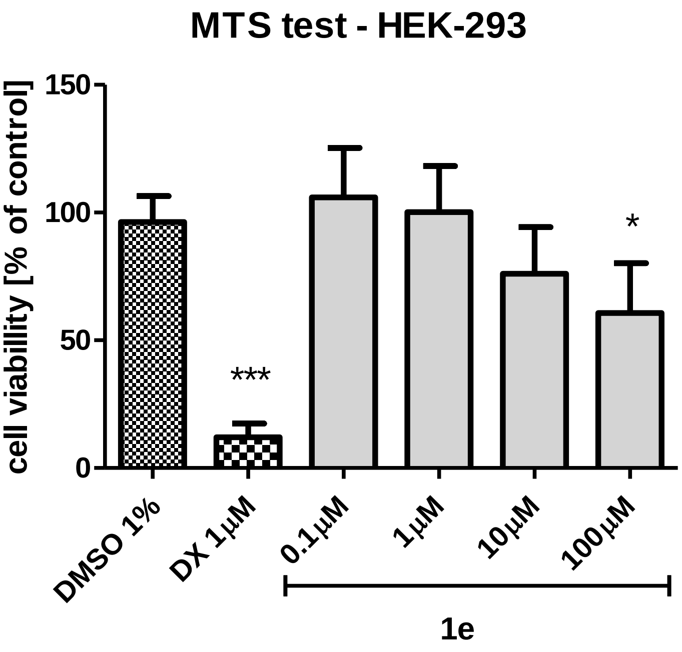

* = $p < 0.05$
*** = $p < 0.001$

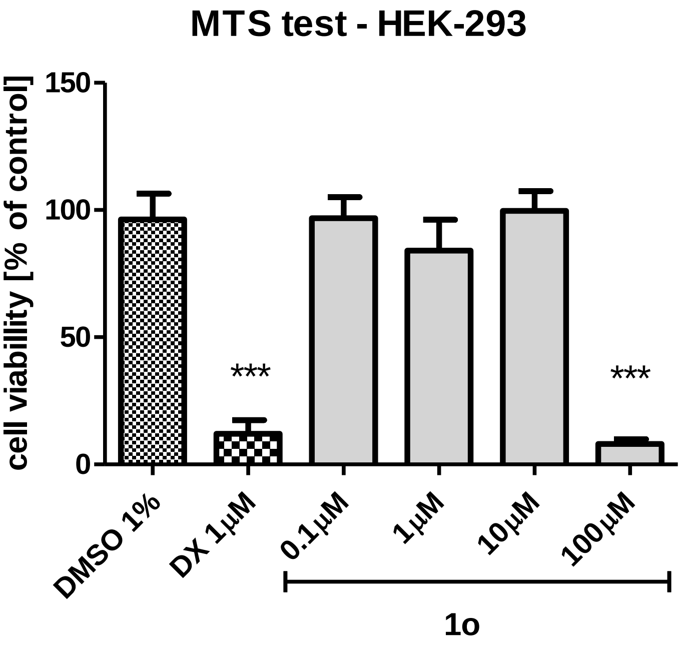

*** = $p < 0.001$

Fig. The HEK-293 cell line viability in the presence of **1e** and **1o** after 72h of incubation. DMSO 1% (vehicle), DX (doxorubicin).

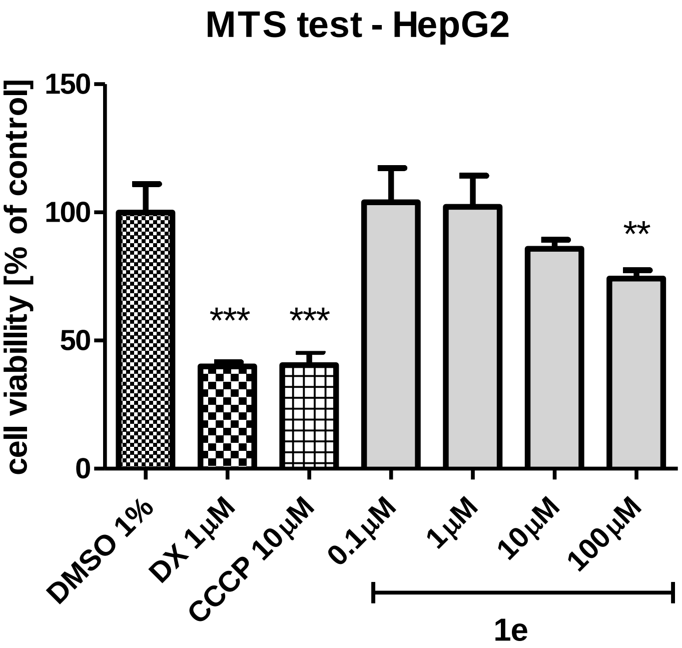

** = *p* < 0.01
*** = *p* < 0.001

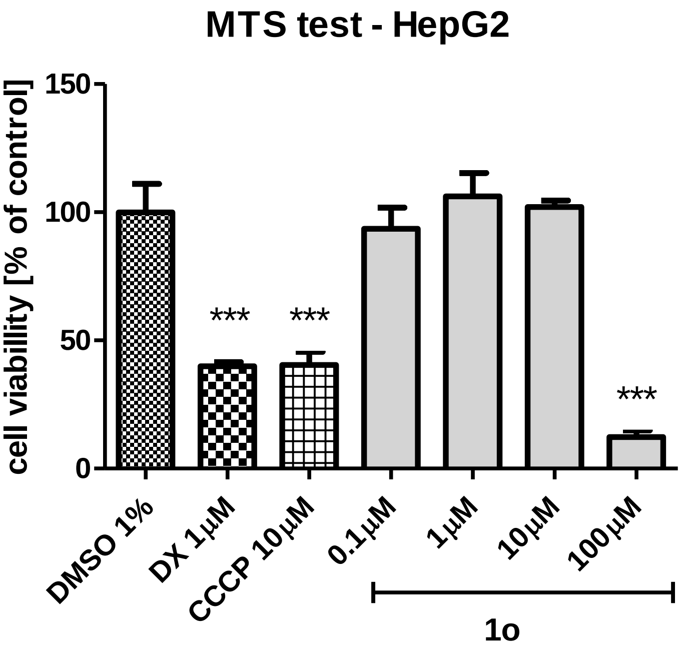

*** = *p* < 0.001

Fig. The HepG2 cell line viability in the presence of **1e** and **1o** after 72h of incubation. DMSO 1% (vehicle), DX (doxorubicin), CCCP carbonyl cyanide 3-chlorophenylhydrazone.

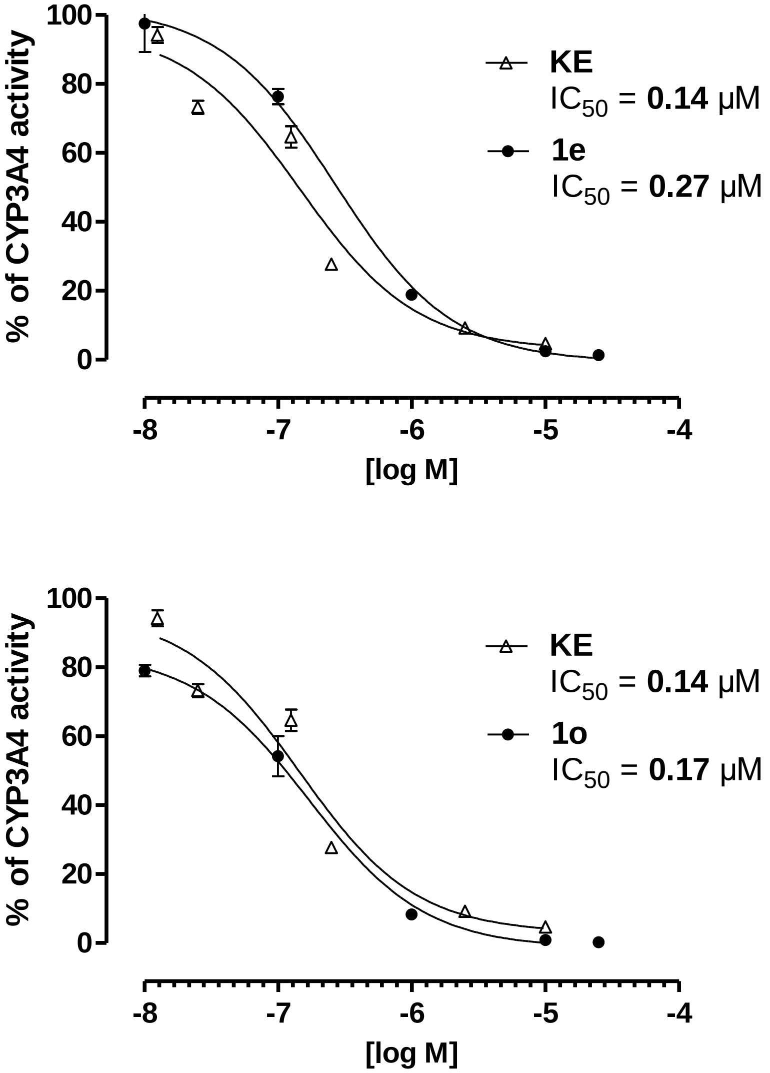

Fig. Effect of ketoconazole (KE), **1e** and **1o** on CYP3A4 activity

VII. Pharmacokinetic analysis for compound 1o

Materials and Methods

Animals

A group of 32 adult male mice (CD-1, 18-21 g) were used in the experiment. The animals were purchased from the Animal House at the Faculty of Pharmacy, Jagiellonian University Medical College, Krakow, Poland. During the habituation period the groups of 4 mice were kept in a plastic cage (252 mm x 167 cm x 140 cm) at a controlled room temperature (22±2°C), humidity (55±10%), full spectrum cold white light (350–400 lx), on 12 h light/dark cycles (the lights on at 7:00 a.m., and off at 19:00 p.m.), and had free access to standard laboratory pellet and tap water. For pharmacokinetic study 1o dissolved in saline was administered by an intraperitoneal (*i.p.*) administration at a dose of 5 mg/kg. Blood samples were collected at 0 min (predose), 5 min, 15 min, 30 min, 60 min, 120 min, 240 min and 360 min after compound administration. The blood and brain samples were collected under general anesthesia induced by *i.p*. injections of 50 mg/kg ketamine plus 8 mg/kg xylazine. The blood samples were taken into heparinized tubes, immediately centrifuged at 3500 rpm for 10 min and plasma was collected. The brain and plasma samples were immediately frozen at - 80°C for LC/MS/MS analysis.

All experimental procedures were carried out in accordance with EU Directive 2010/63/EU and approved by the I Local Ethics Committee for Experiments on Animals of the Jagiellonian University in Krakow, Poland (approval numbers: 123/2015).

Pharmacokinetic study

Pharmacokinetic parameters were calculated by a non-compartmental approach from the average concentration values, using Phoenix WinNonlin software (Certara, Princeton, NJ 08540 USA). First order elimination rate constant (λ_z) was calculated by linear regression of time versus log concentration. Next, the area under the mean serum and tissue concentration versus time curve ($AUC_{0\rightarrow t}$) was estimated using the log-linear trapezoidal rule (Eq. 1), where C_n is the concentration of last sampling of each compound.

(Eq. 1)

Area under the first-moment curve ($AUMC_{0\rightarrow t}$) was estimated by calculation of the total area under the first-moment curve using the equation 2, where t_n is the time of last sampling.

(Eq. 2)

Mean residence time (MRT) was calculated as:

$$MRT = \frac{AUMC_{0\rightarrow t}}{AUC_{0\rightarrow t}}$$

(Eq. 3)

Total clearance (Cl_T) was calculated as:

$$Cl_T = \frac{F \cdot D_{i.p.}}{AUC_{0\rightarrow t}}$$

(Eq. 4)

Volume of distribution (V_d) was calculated as:

$$V_d = \frac{F \cdot D}{\lambda_z \cdot AUC_{0\rightarrow t}}$$

(Eq. 5)

where $D_{i.p.}$ is an *i.p.* dose of **1o**.

Analytical method

The quantification of studied compound in plasma and brain samples was done using UPLC Agilent 1100 system (Agilent Technologies, Waldbronn, Germany) coupled to the triple quadrupole mass spectrometer API 2000 (ABSciex, Framingham, Massachusetts, USA) equipped with electrospray ion source. After preparation, the samples were injected (20 µL) onto XBridge C_{18} (2.1 mm x 30 mm, 3.5 µm, Waters, Milford, Massachusetts, USA) analytical column. The mobile phase consisted of ACN with 0.1% formic

acid (A) and water with 0.1% formic acid (B) were delivered in gradient elution started with 90% of eluent B, increasing during 2 min to 90% of eluent A, maintained to 3 min, and then returned during 5 min to 90% of eluent B, and maintained 90% of eluent B during 5 min at a flow rate of 300 μL/min. The total time of analysis was 10 min. Electrospray ionization process was performed in positive ionization and the data acquisition was carried out in multiple reaction monitoring mode (MRM) for 1o and its internal standard (nebivolol). The ion spray source settings were as follows: spray voltage: 5.5 kV, heater temperature: 400°C, curtain gas: 20 psi, source gas 1: 40 psi, source gas 2: 20 psi. The ions measured were: m/z 338.1 (Q1) and m/z 211.1 (Q3) for 1o, and m/z 406.2 (Q1) and m/z 151.1 (Q3) for IS, Table 3.

Standard solutions preparation

An amount of 10 mg of 1o was accurately weighted and quantitatively transferred into the 10 mL volumetric flask using MeOH. After the salts dissolving flask was filled to the 10 mL mark with MeOH obtaining 1 mg/mL of an analyte. Further dilutions were performed using MeOH to prepare working standard solutions of analyte at following concentrations: 0.025, 0.05, 0.1, 0.25, 0.5, 1.0, 2.5, 5.0, 10, 25 and 50 μg/mL for calibration curve samples (CC) and 0.025, 0.075, 2.2, 4.5 μg/mL for quality control samples (QC). To prepare samples for calibration curve or quality control samples 45 μL of matrix (plasma or brain homogenate) were spiked with 5 μL of internal standard solution (IS) obtaining the final concentration of 100 ng/mL and 5 μL of standards working solutions at needed CC or QC concentration levels. After standards solution addition, samples were mixed and purified.

Sample preparation

A sample volume of 50 μL of plasma or brain homogenate was transferred into the clean Eppendorf tube and spiked with 5 μL of IS solution (1μg/mL) obtaining the final concentration of 100 ng/mL. After 5 min of mixing (1500 rpm) proteins were precipitated using 150 μL ACN. After 10 min of samples shaking (1500 rpm), the incubation step was performed (10 min, 4°C). Next, samples were centrifuged (10 000 rpm, 10 min, 4°C) and the supernatant was transferred into chromatographic vial for LC/MS/MS analysis.

The brain homogenate was prepared maintaining the tissue : phosphate-buffered saline (PBS) at a ratio of 1:5. The homogenization was carried out employing IKA® T10 Basic ULTRA-TURRAX disperser (IKA

Werke GmbH & Co. KG, Staufen, Germany). After homogenization 50 μL of sample was collected for further preparation. All samples were stored on ice during the preparation process.

VIII Novel Object recognition experiment for compound 1o

The method was performed according to Nilsson et al. (2007) and Woźniak et al. (2016 b). The animals were trained and tested in a black, plastic, open field (50 × 30 cm, 35 cm high) with the floor divided into 20-cm square sections. The open field was in a dark room illuminated only by a 25 W bulb. On the first day (adaptation) the animals were allowed to explore the open field for 10 min. On the next day (training, T1) the animals were administered the tested drugs, placed in the apparatus and allowed to explore two identical objects (cylindrical objects with walls painted white, 7 cm in diameter, 11 cm high) for the time required to complete 15 s of exploration of either object. For the retention trial (T2) conducted one h later, one of the objects presented in T1 was replaced with a novel object (a prism-shaped object with walls painted black, 5 cm wide, 14 cm high). The duration of exploration of each object (i.e., sitting in close proximity to the objects or sniffing or touching them) during 5 min time was video recorded and measured separately by a trained observer. All drugs were administered before the training (T1) session. MK-801 (0.3 mg/kg, i.p.) was given 30 min before the session. Investigated compounds were administered 60 min before MK-801.

VI. ^{1}H NMR, ^{13}C NMR and LC-MS spectra of key products

1a: 3-(1-methyl-1*H*-imidazol-5-yl)-1*H*-indole (AGH-38)

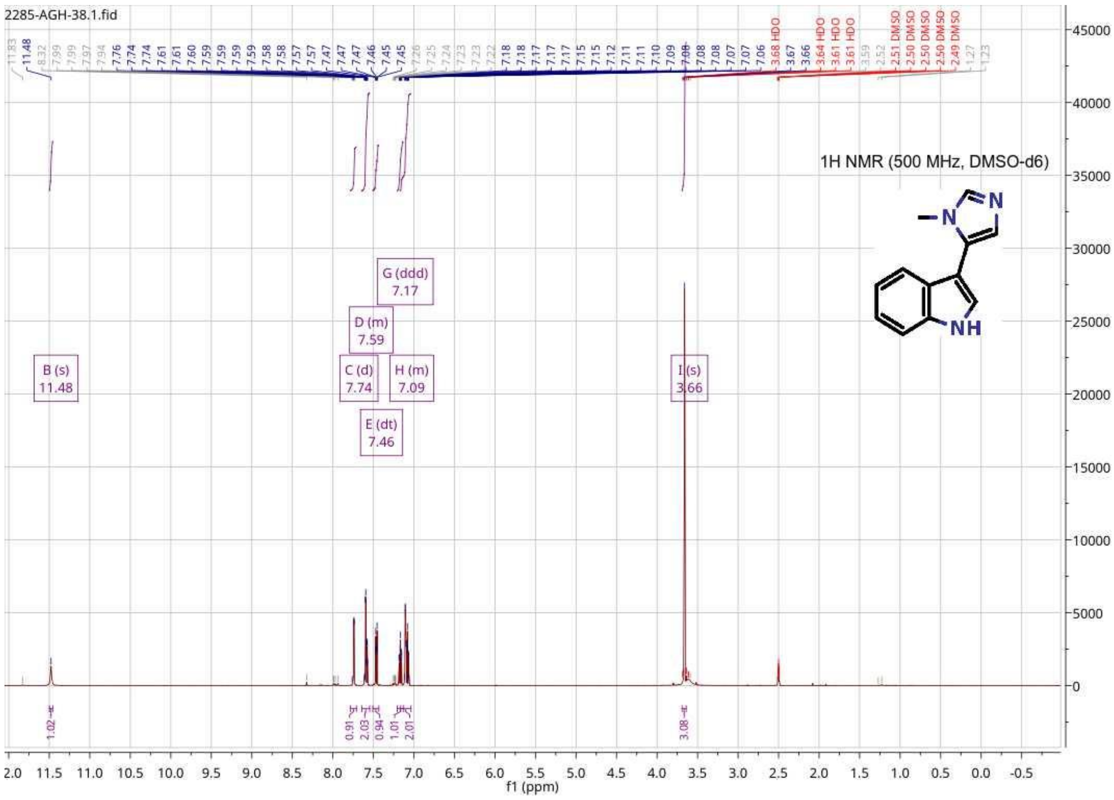

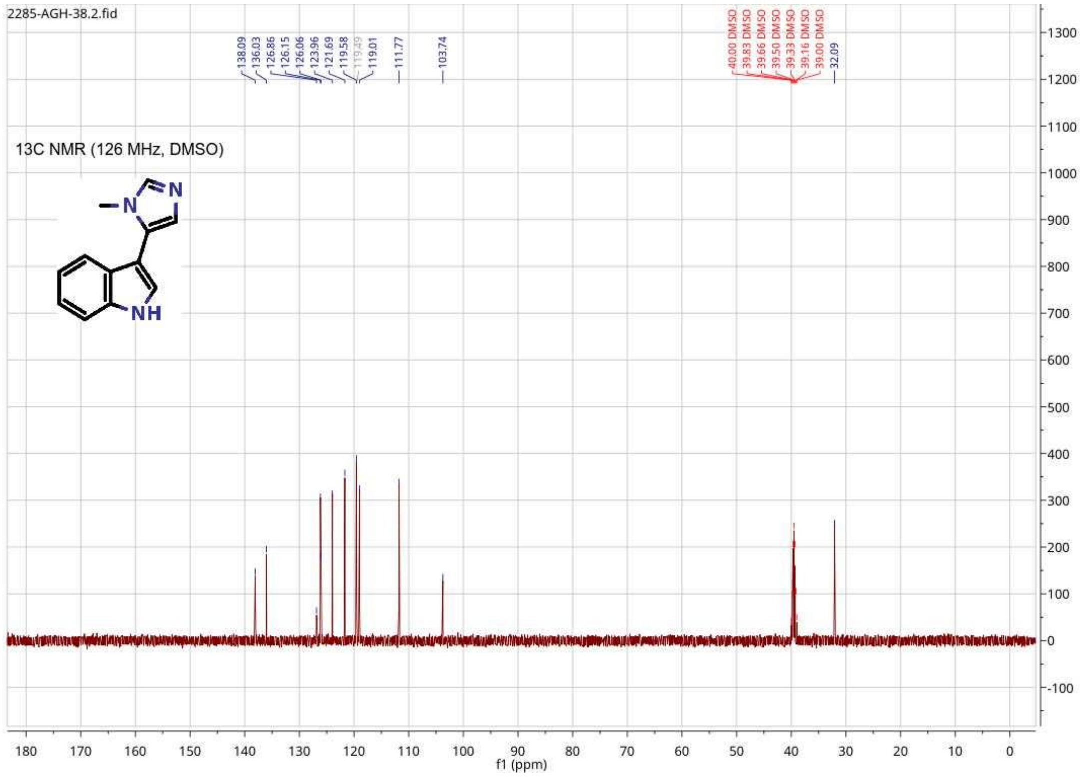

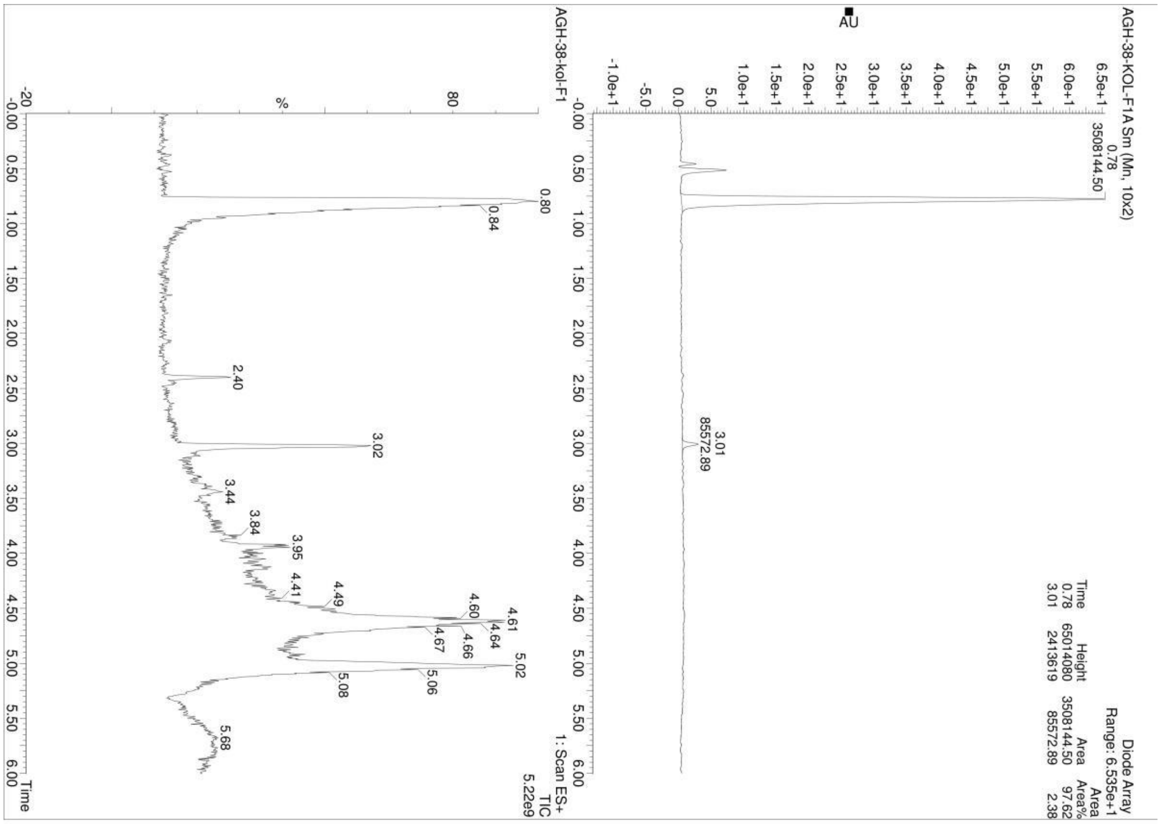
AGH-38-KOL-F1A Sm (Mn, 10x2)
Diode Array
Range: 6.535e+1
Time Height Area Area%
0.78 65014080 3508144.50 97.62
3.01 2413619 85572.89 2.38
AGH-38-kol-F1
1: Scan ES+
TIC
5.22e9
Time

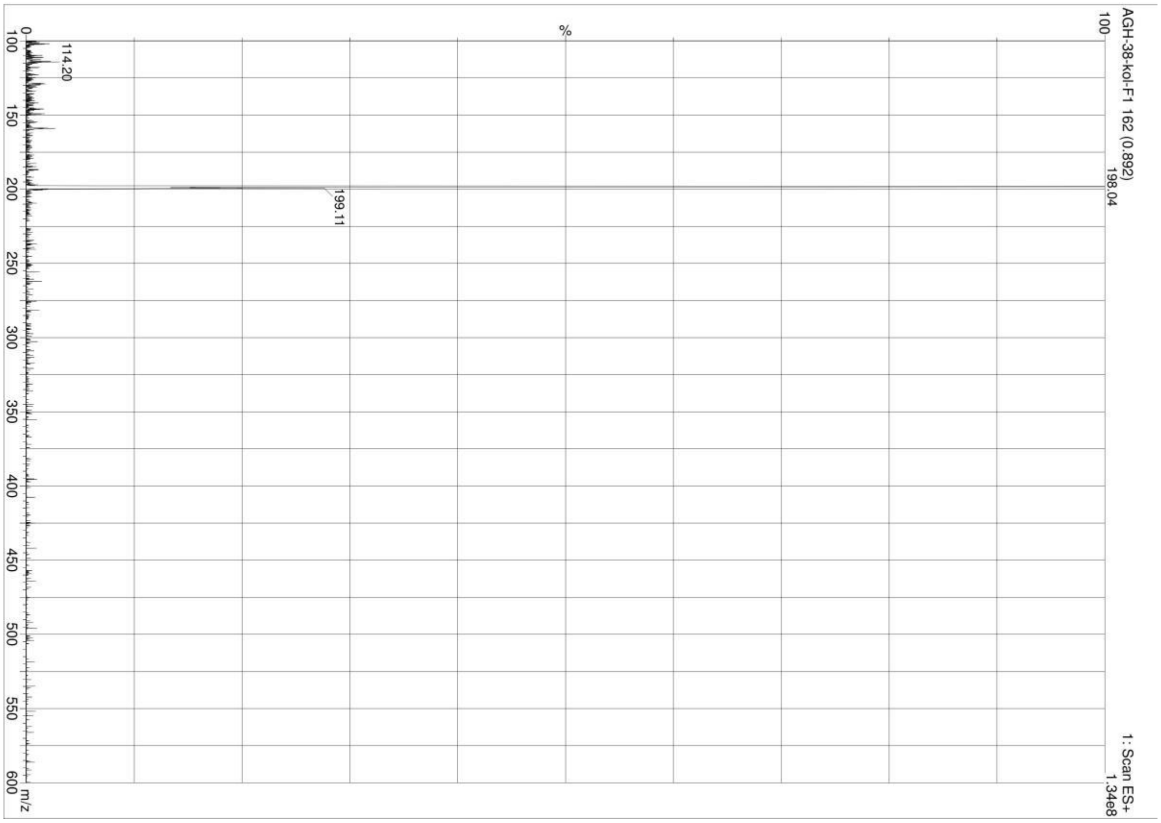
AGH-38-kol-F1 162 (0.892)
198.04
199.11
114.20
1: Scan ES+
1.34e8
m/z

1b: 5-methoxy-3-(1-methyl-1*H*-imidazol-5-yl)-1*H*-indole (AGH-39)

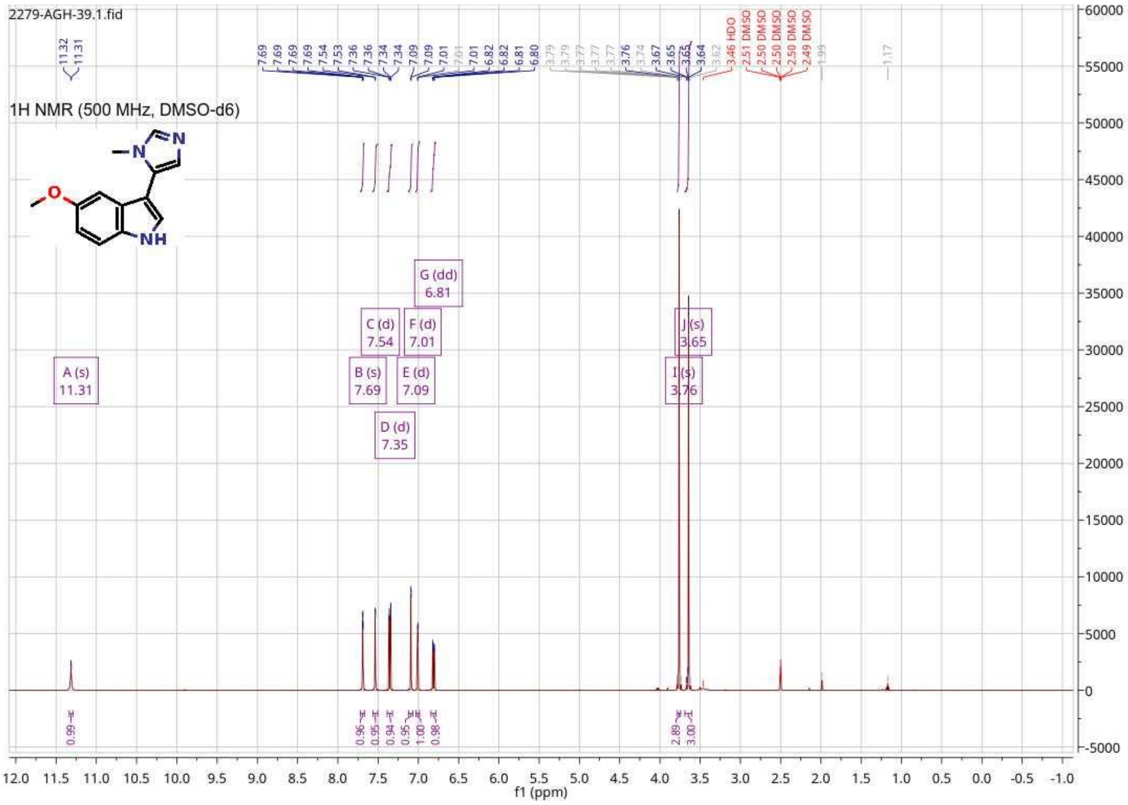

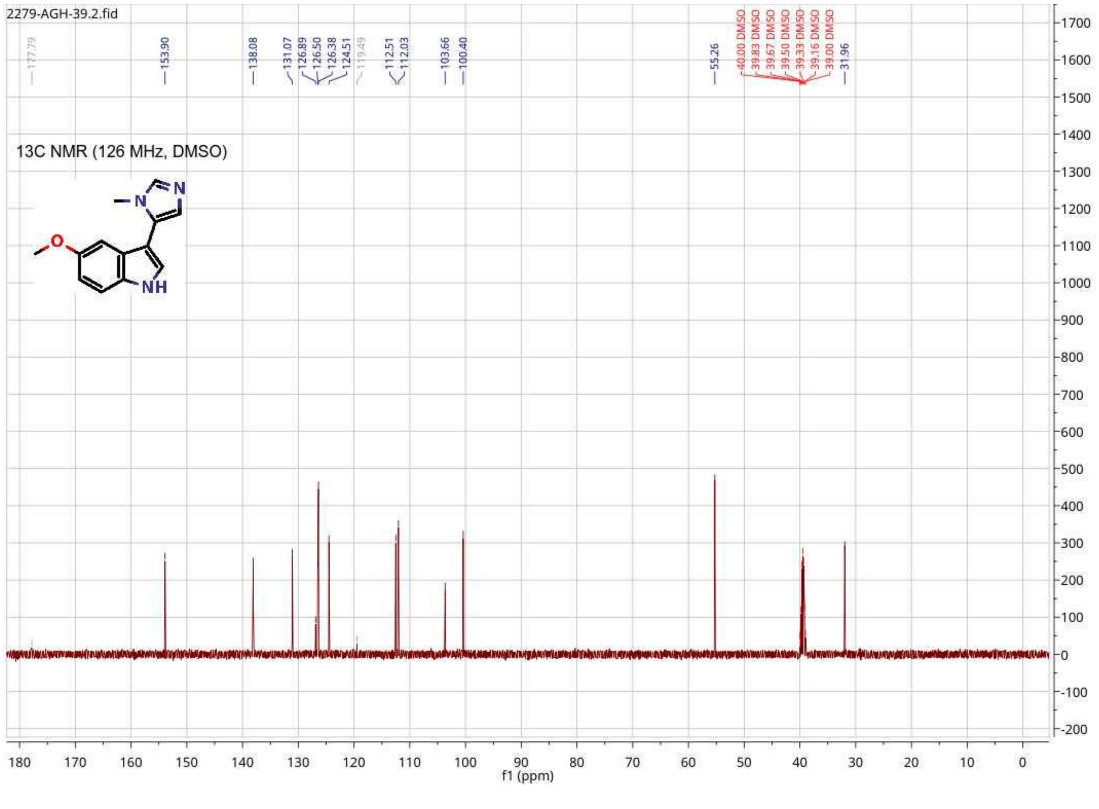

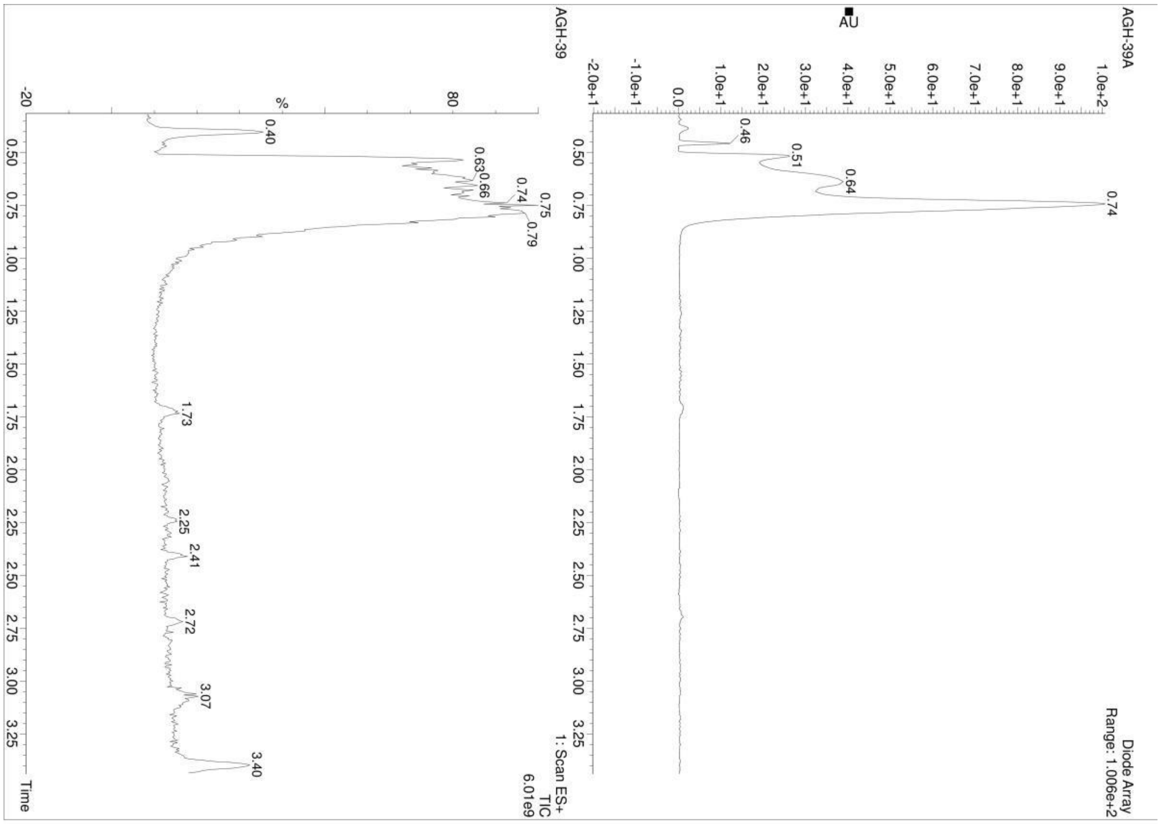
AGH-39A
Diode Array
Range: 1.006e+2
AU
AGH-39
1: Scan ES+
TIC
6.01e9
Time
0.40
0.46
0.51
0.63
0.64
0.66
0.74
0.75
0.79
1.73
2.25
2.41
2.72
3.07
3.40

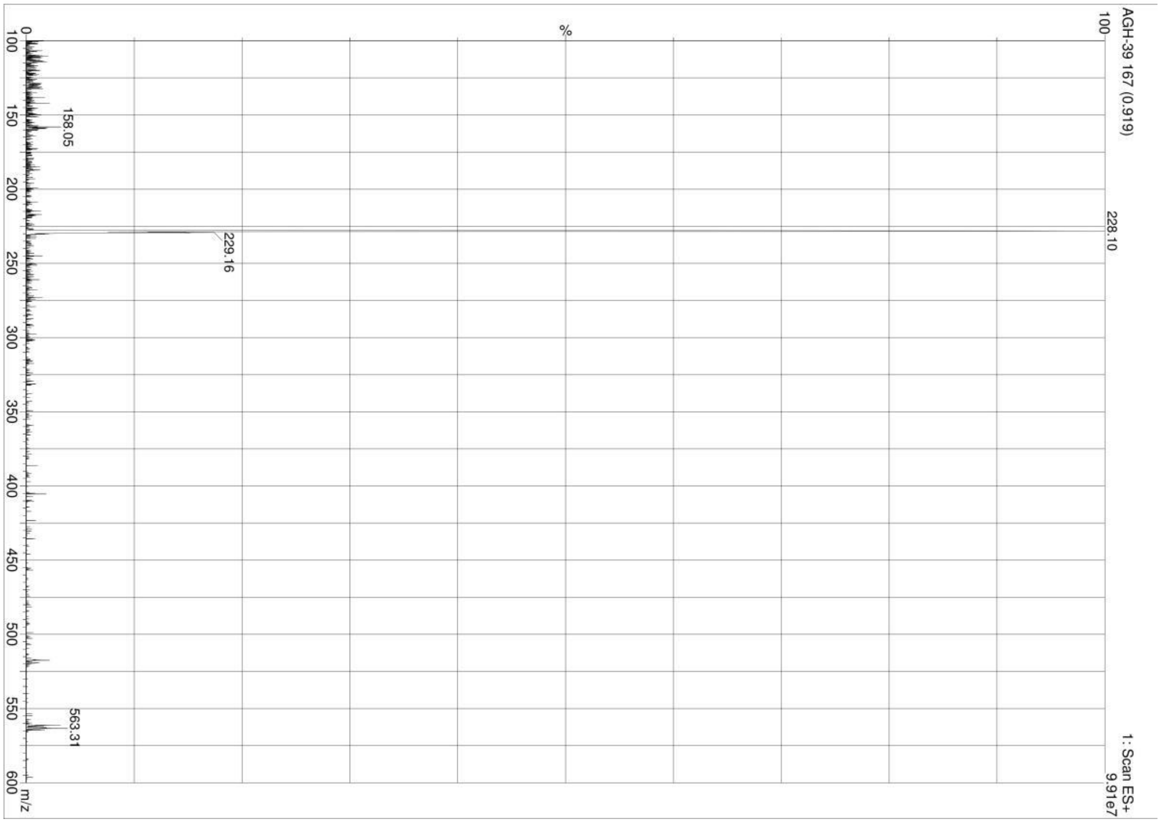
AGH-39 167 (0.919)
1: Scan ES+
9.91e7
%
m/z
228.10
229.16
158.05
563.31

1c: 3-(1-ethyl-1*H*-imidazol-5-yl)-1-methyl-1*H*-indole (AGH-61)

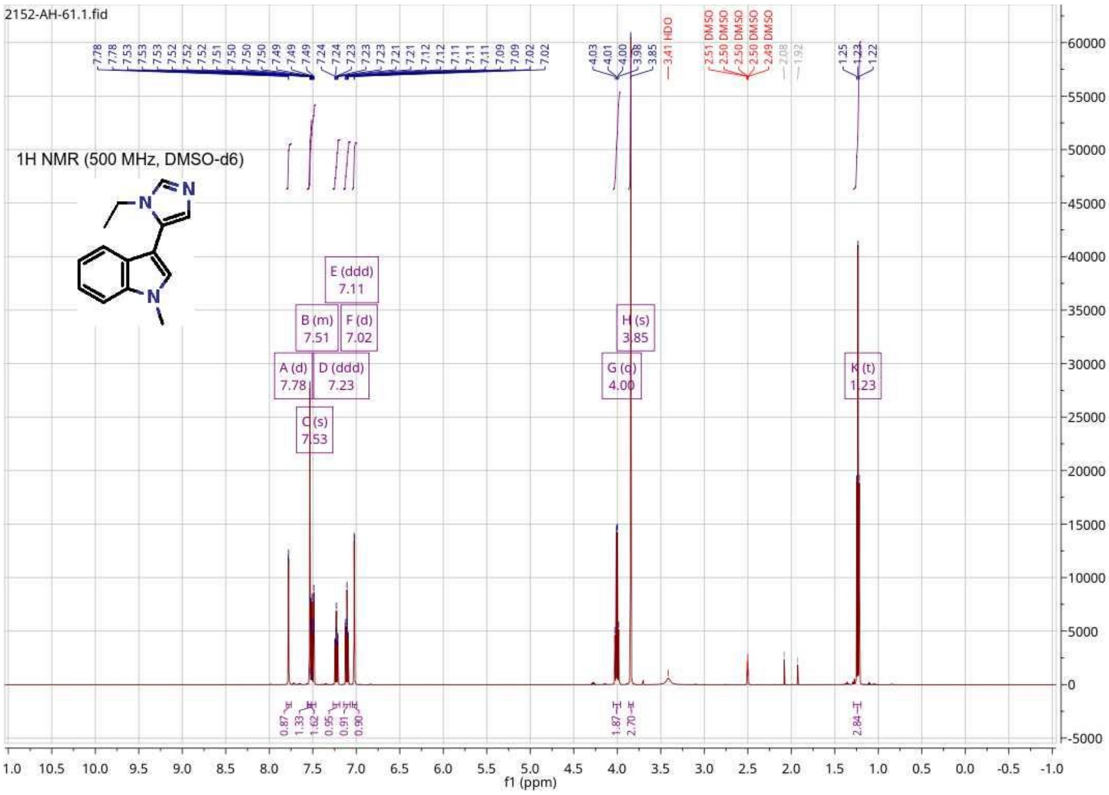

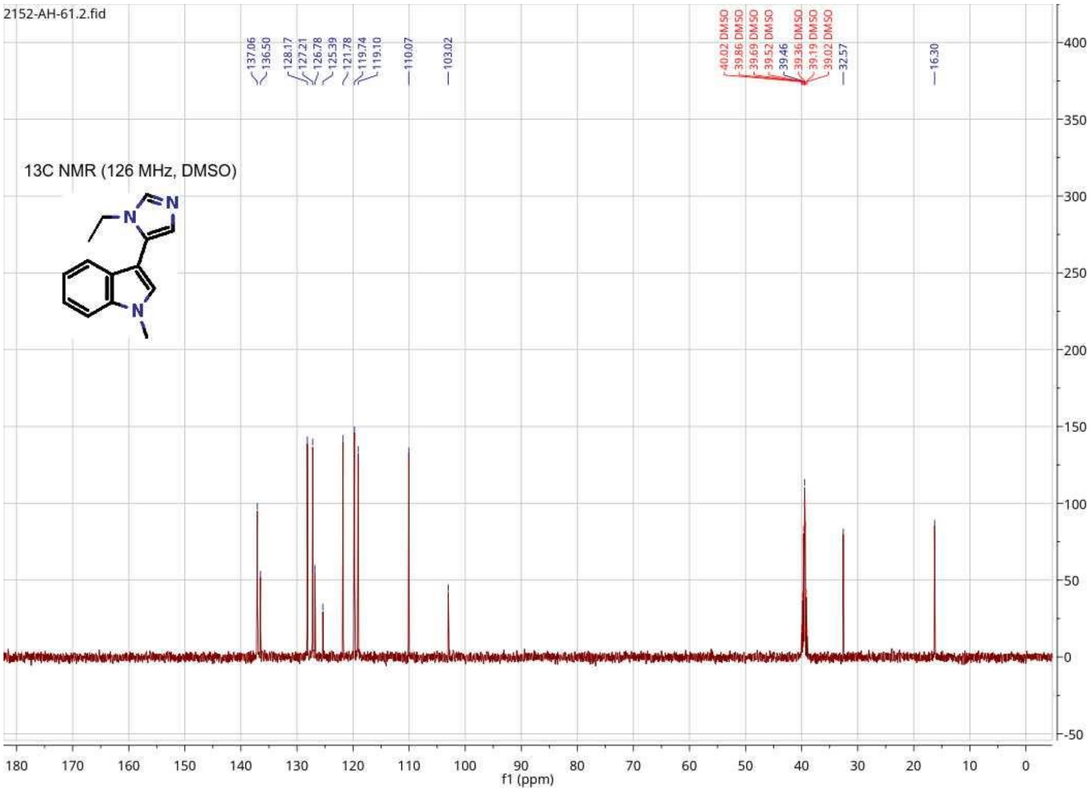

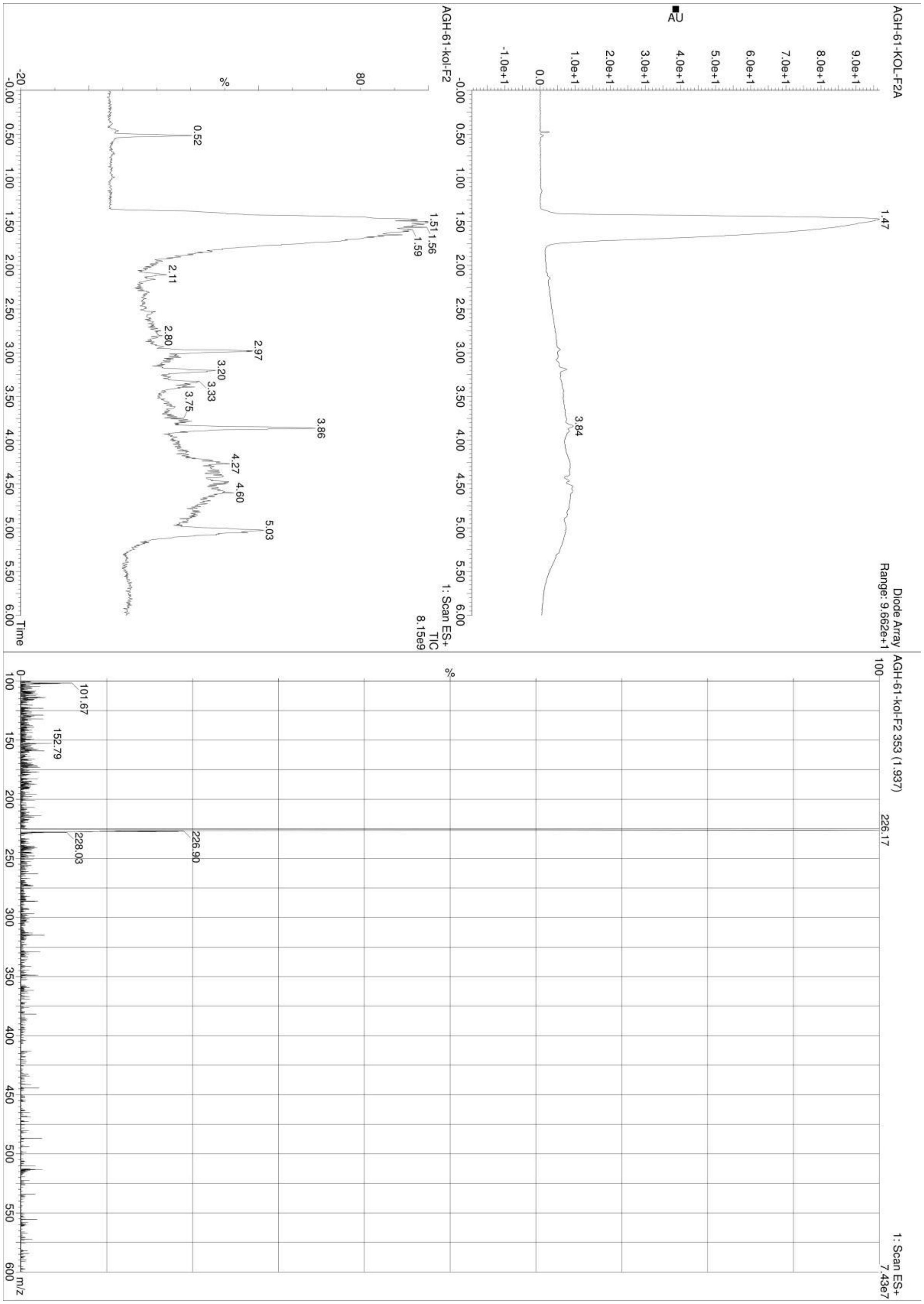
AGH-61-KOL-F2A
AU
Diode Array
Range: 9.662e+1
1.47
3.84
AGH-61-kol-F2
1: Scan ES+
TIC
8.15e9
0.52
1.51
1.56
1.59
2.11
2.80
2.97
3.20
3.33
3.75
3.86
4.27
4.60
5.03
Time
AGH-61-kol-F2 353 (1.937)
1: Scan ES+
7.43e7
226.17
101.67
152.79
226.90
228.03
m/z

1d: 3-(1-ethyl-1*H*-imidazol-5-yl)-5-methoxy-1-methyl-1*H*-indole (AGH-68)

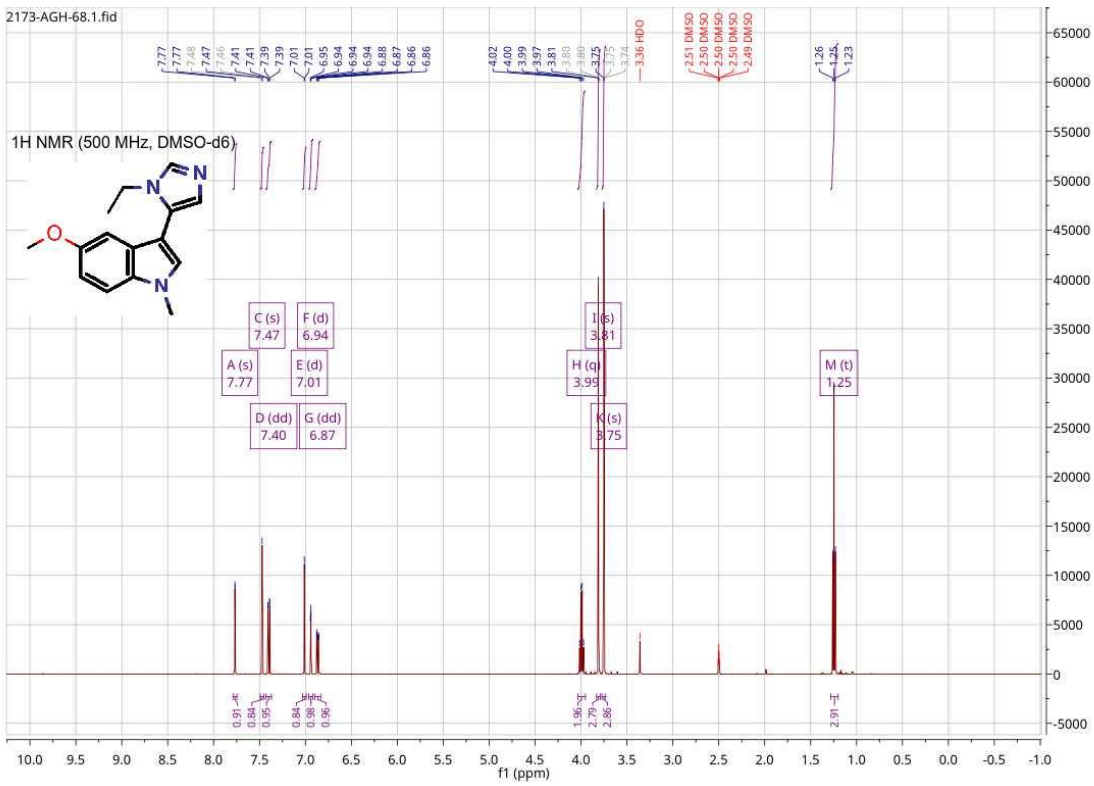

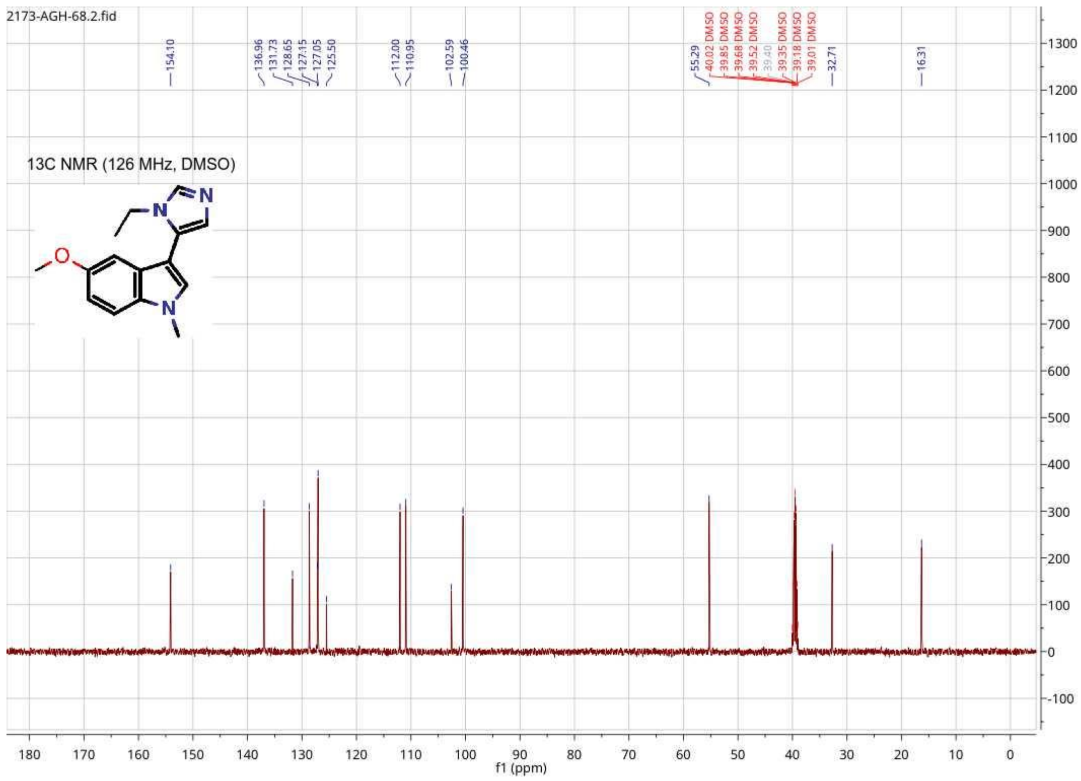

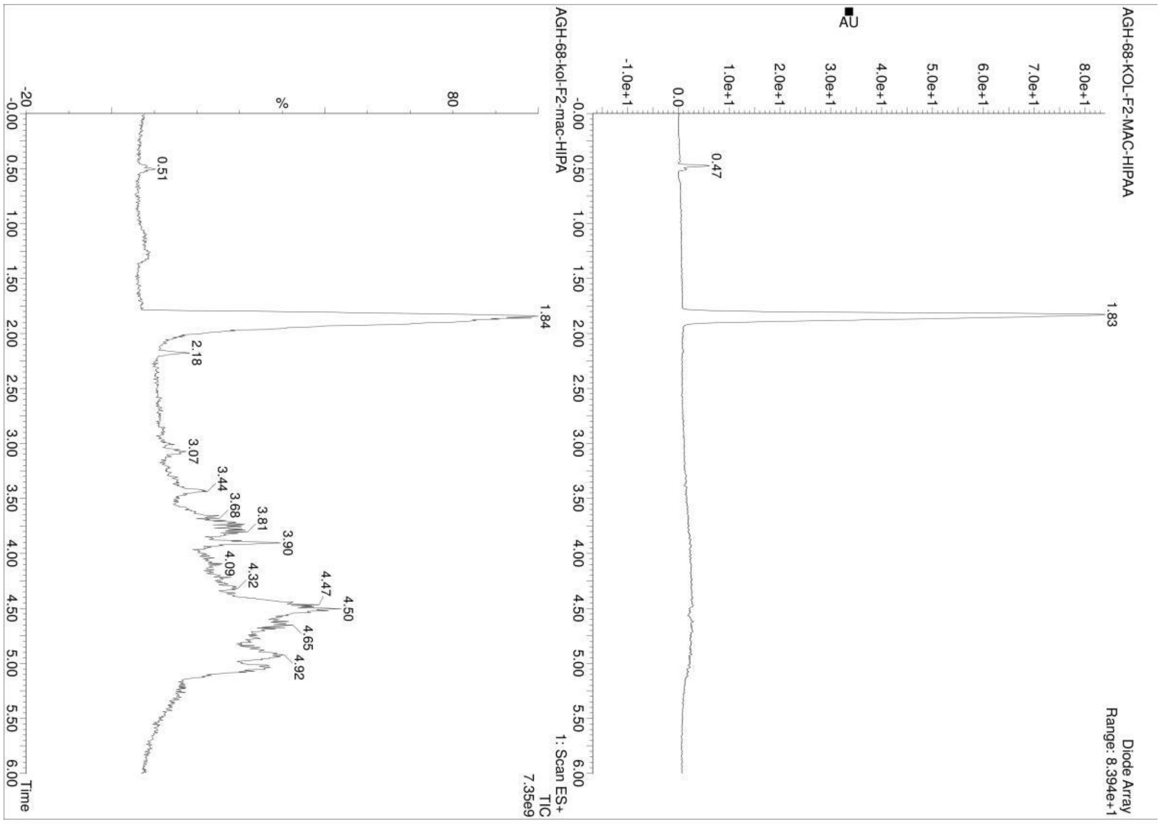
AGH-68-KOL-F2-MAC-HIPAA
Diode Array
Range: 8.394e+1
AU
1.83
0.47
AGH-68-kol-F2-mac-HIPA
1: Scan ES+
TIC
7.35e9
1.84
0.51
2.18
3.07
3.44
3.68
3.81
3.90
4.09
4.32
4.47
4.50
4.65
4.92
Time

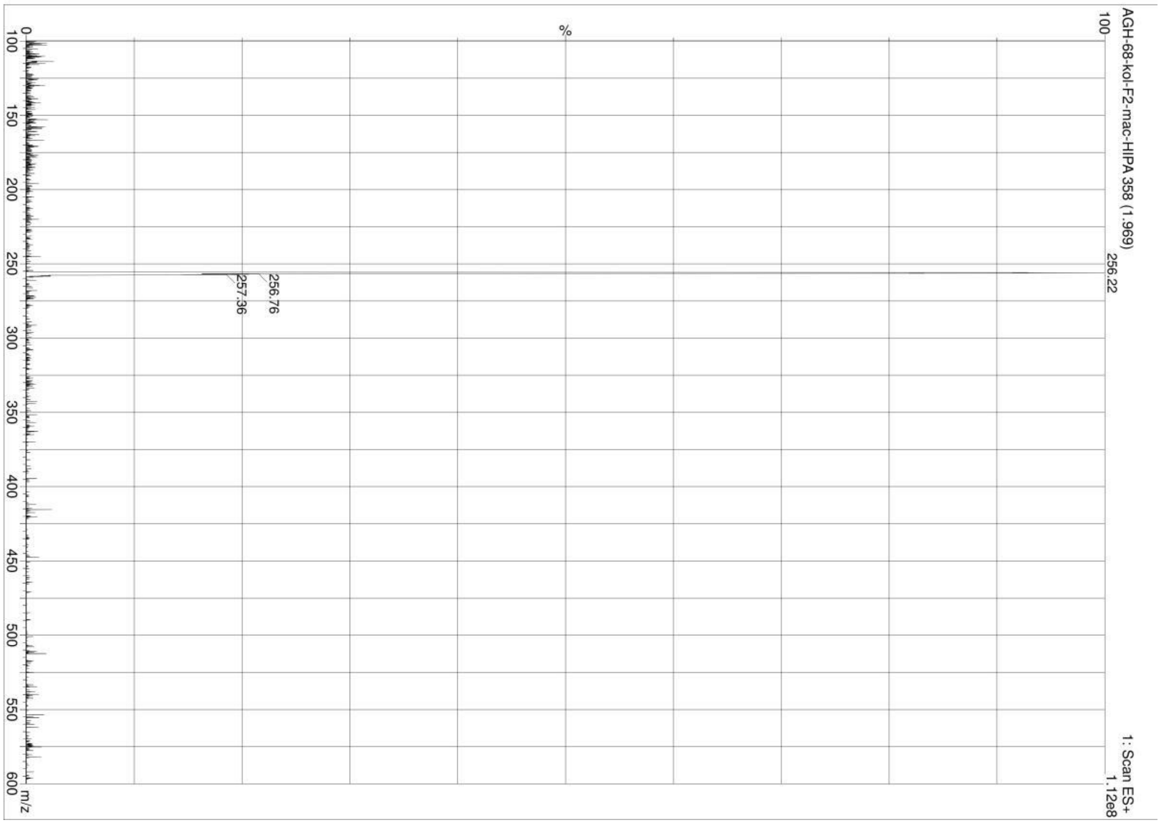
AGH-68-kol-F2-mac-HIPA 358 (1.969)
1: Scan ES+
1.12e8
256.22
256.76
257.36
m/z

1e: 3-(1-ethyl-1*H*-imidazol-5-yl)-5-methoxy-1*H*-indole (AGH-44)

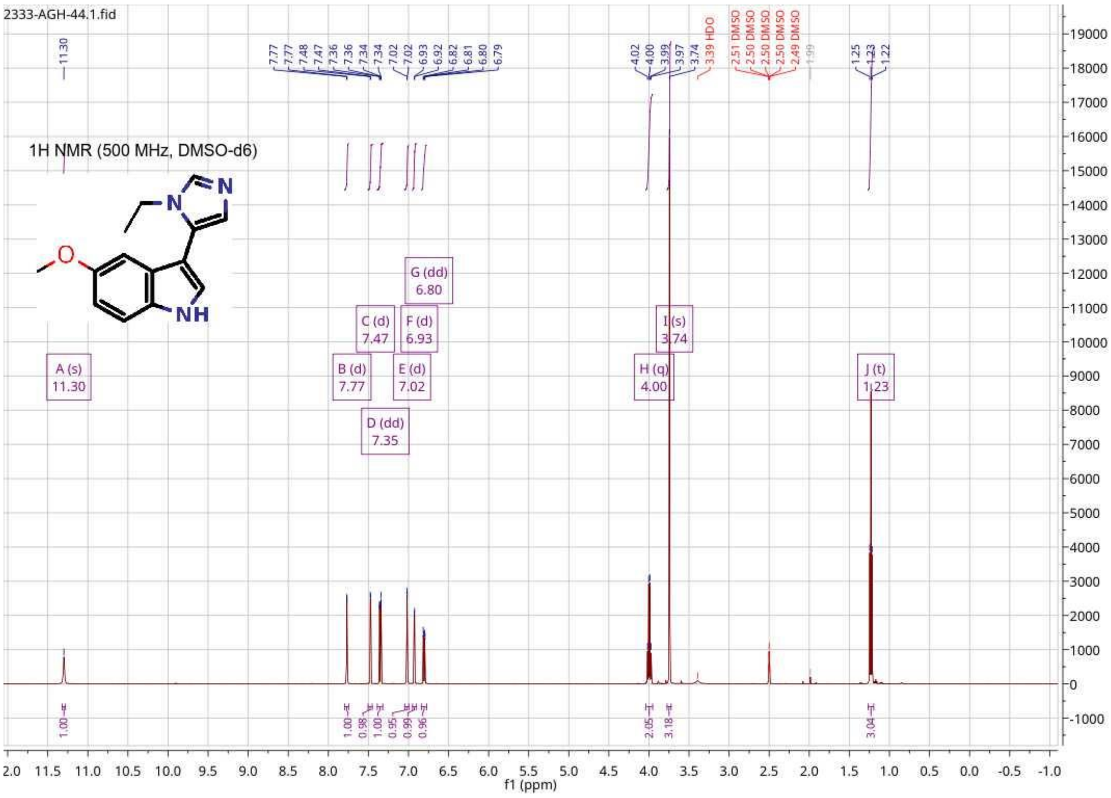

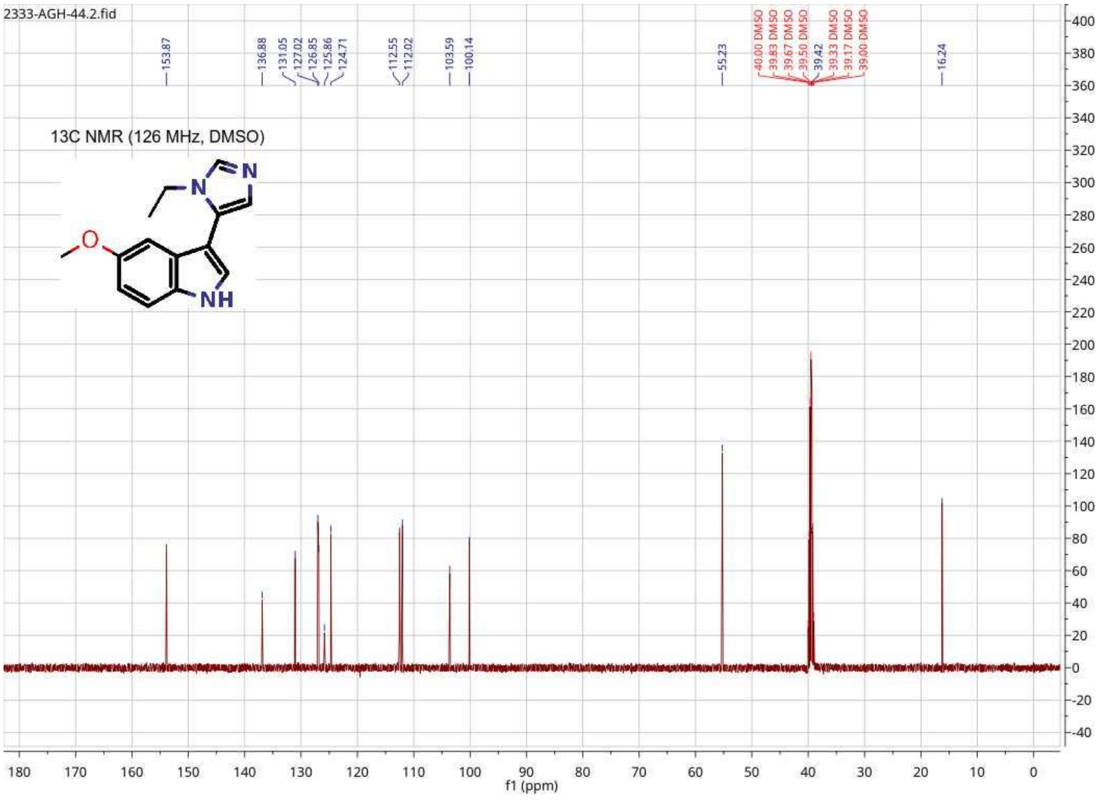

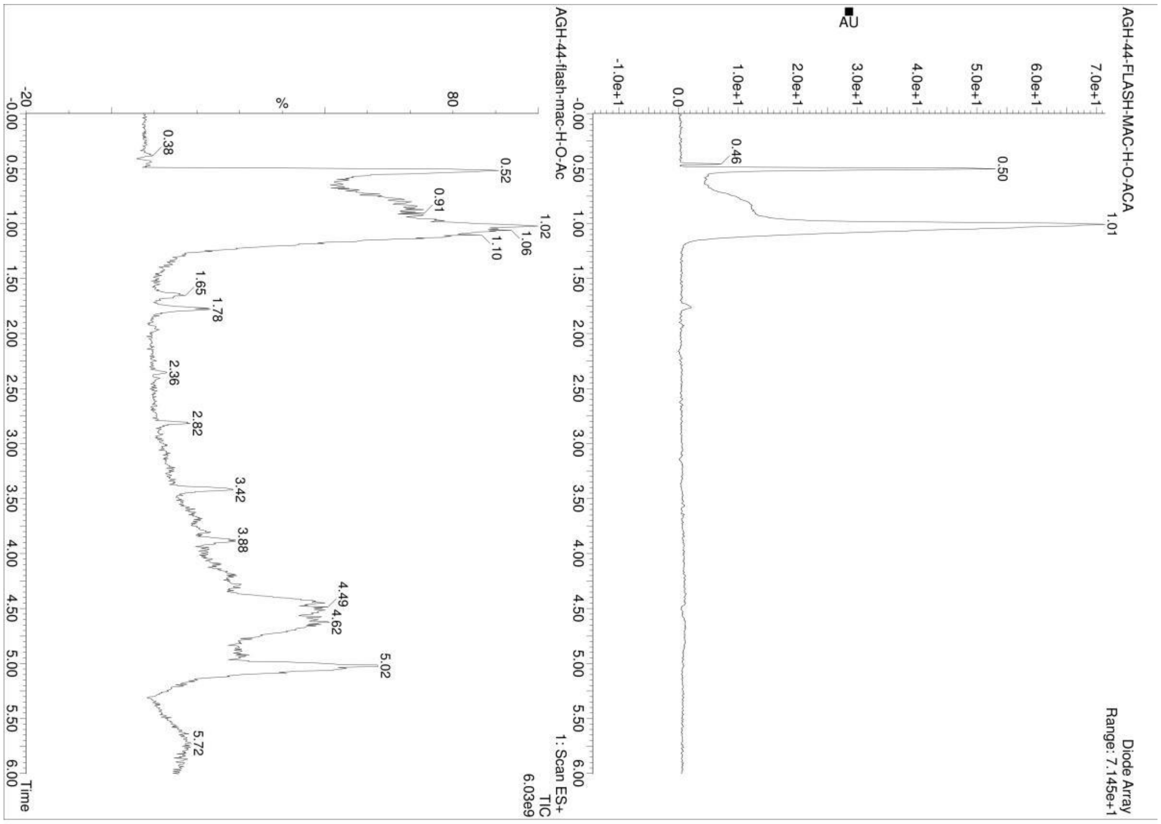
AGH-44-FLASH-MAC-H-O-ACA
Diode Array
Range: 7.145e+1
AU
0.46
0.50
1.01
AGH-44-flash-mac-H-O-Ac
1: Scan ES+
TIC
6.03e9
0.38
0.52
0.91
1.02
1.06
1.10
1.65
1.78
2.36
2.82
3.42
3.88
4.49
4.62
5.02
5.72
Time

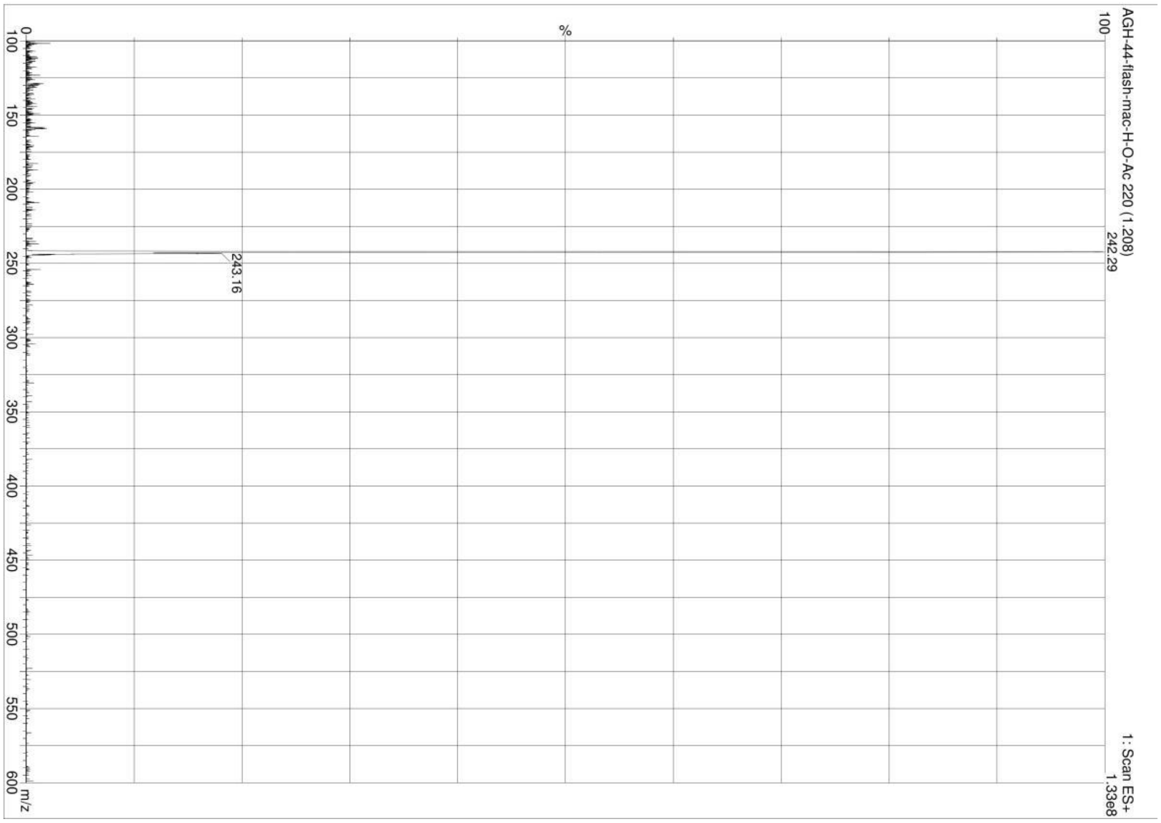
AGH-44-flash-mac-H-O-Ac 220 (1.208)
1: Scan ES+
1.33e8
242.29
243.16
m/z

1f: 3-(1-propyl-1*H*-imidazol-5-yl)-5-methoxy-1*H*-indole (AGH-87)

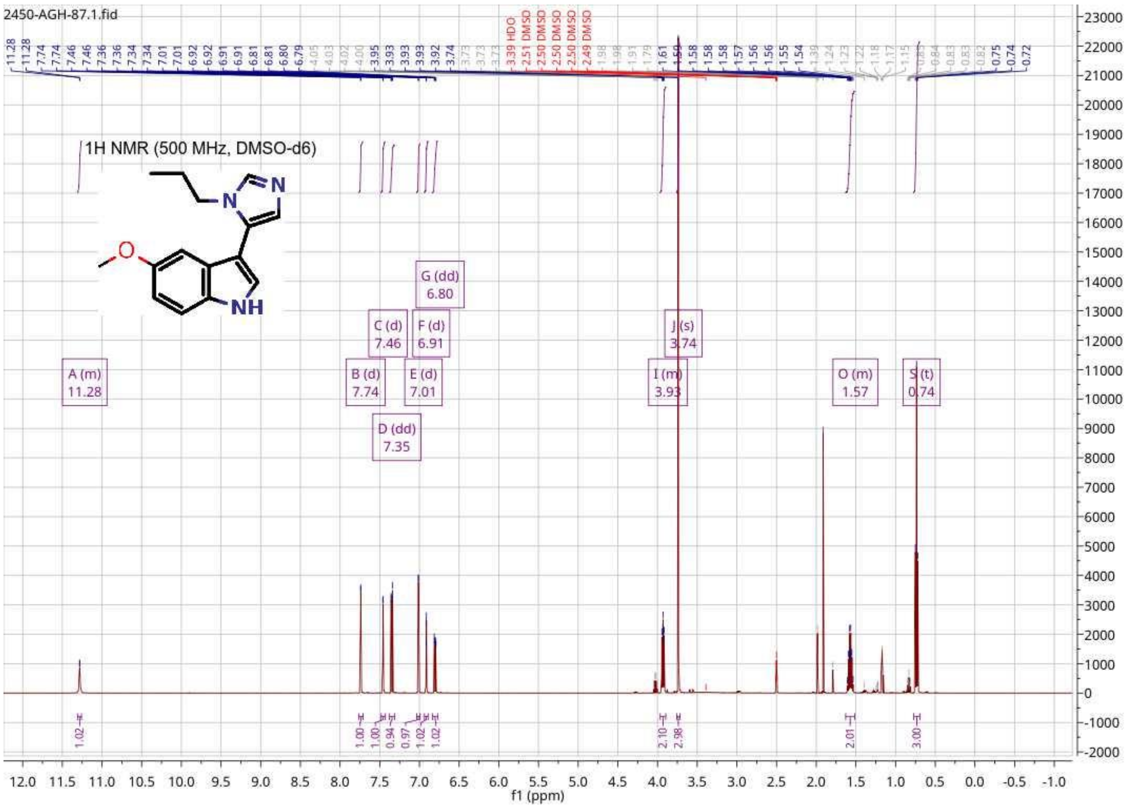

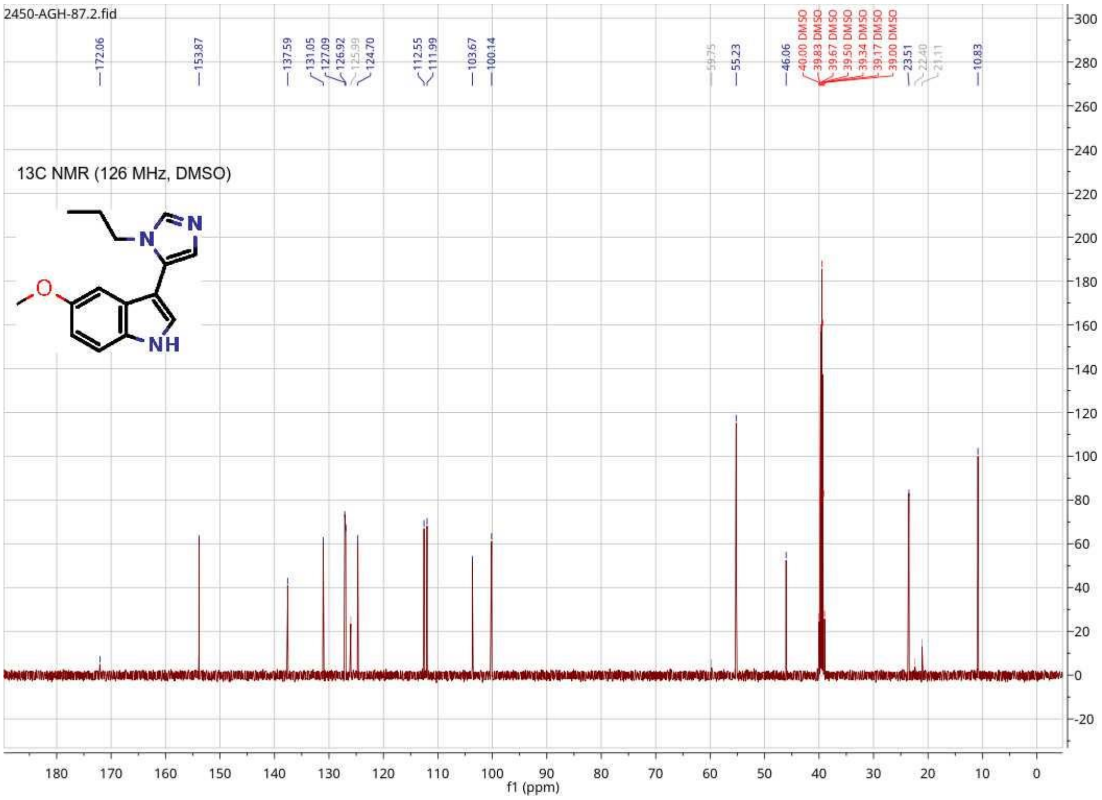

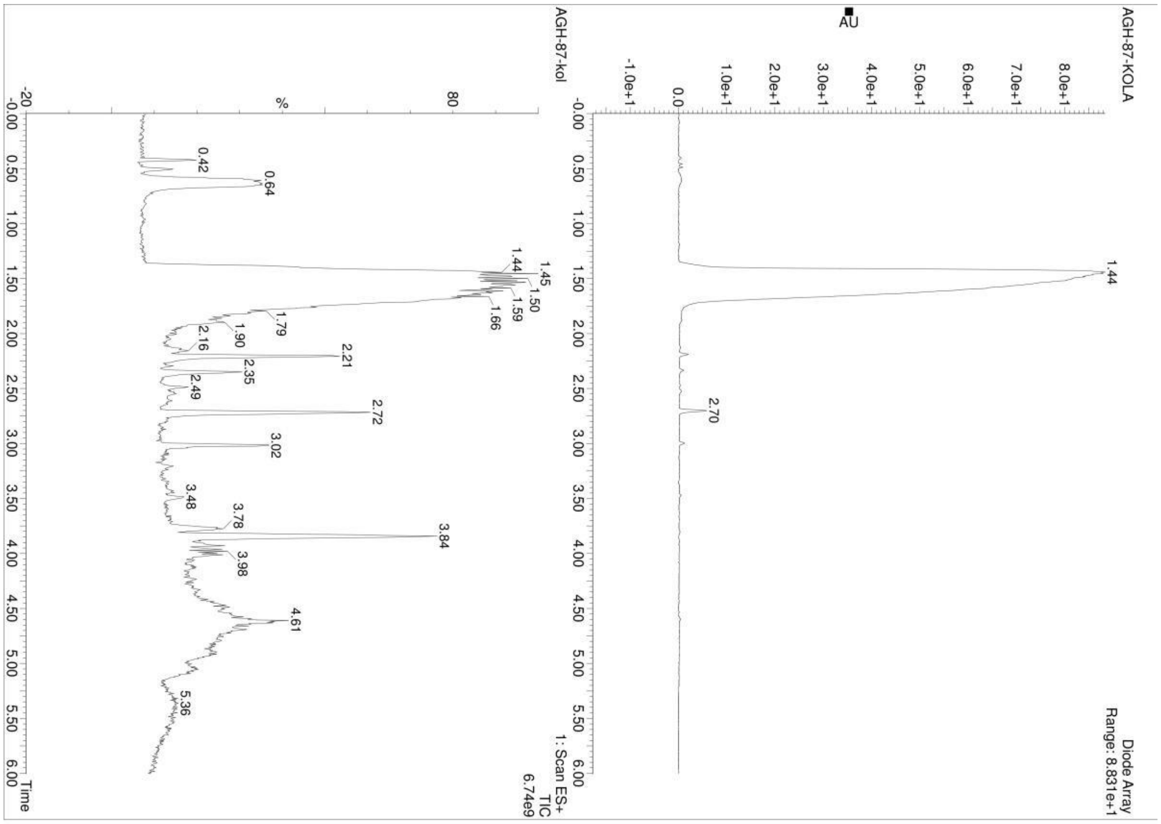

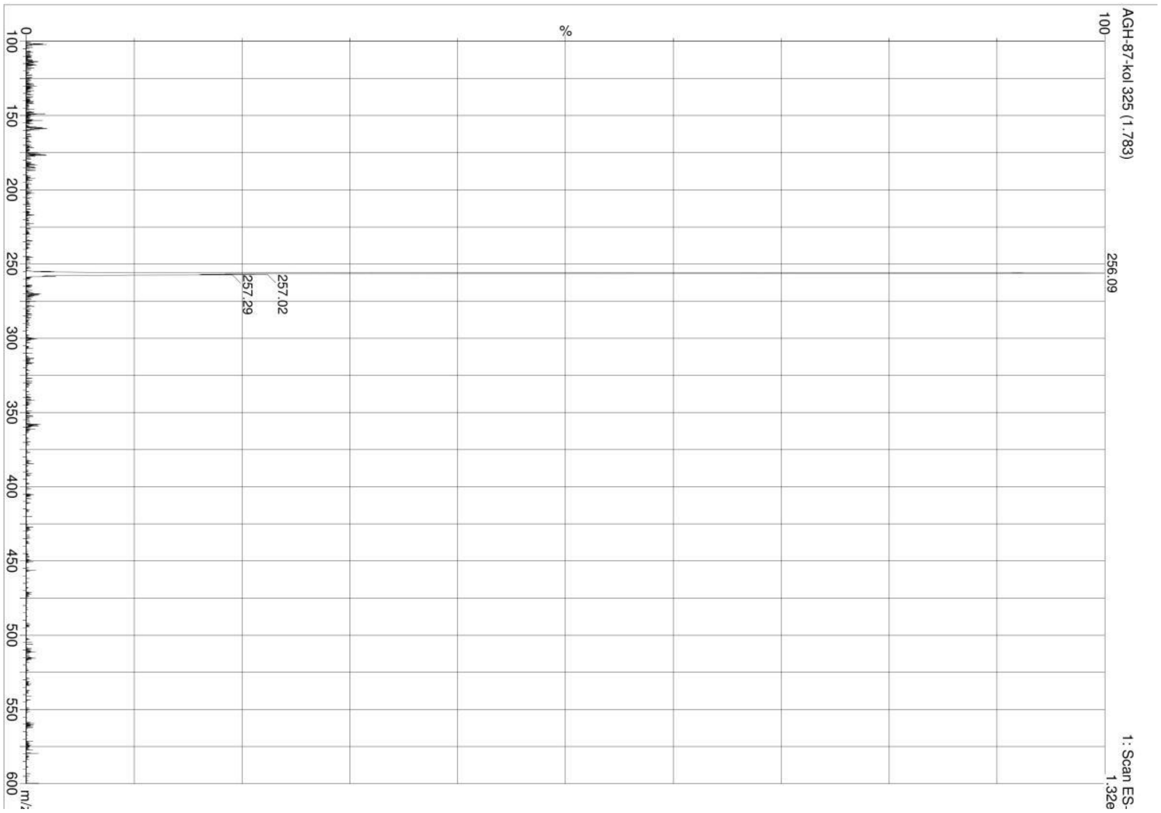

1g: 3-(1-butyl-1*H*-imidazol-5-yl)-5-methoxy-1*H*-indole (AGH-45)

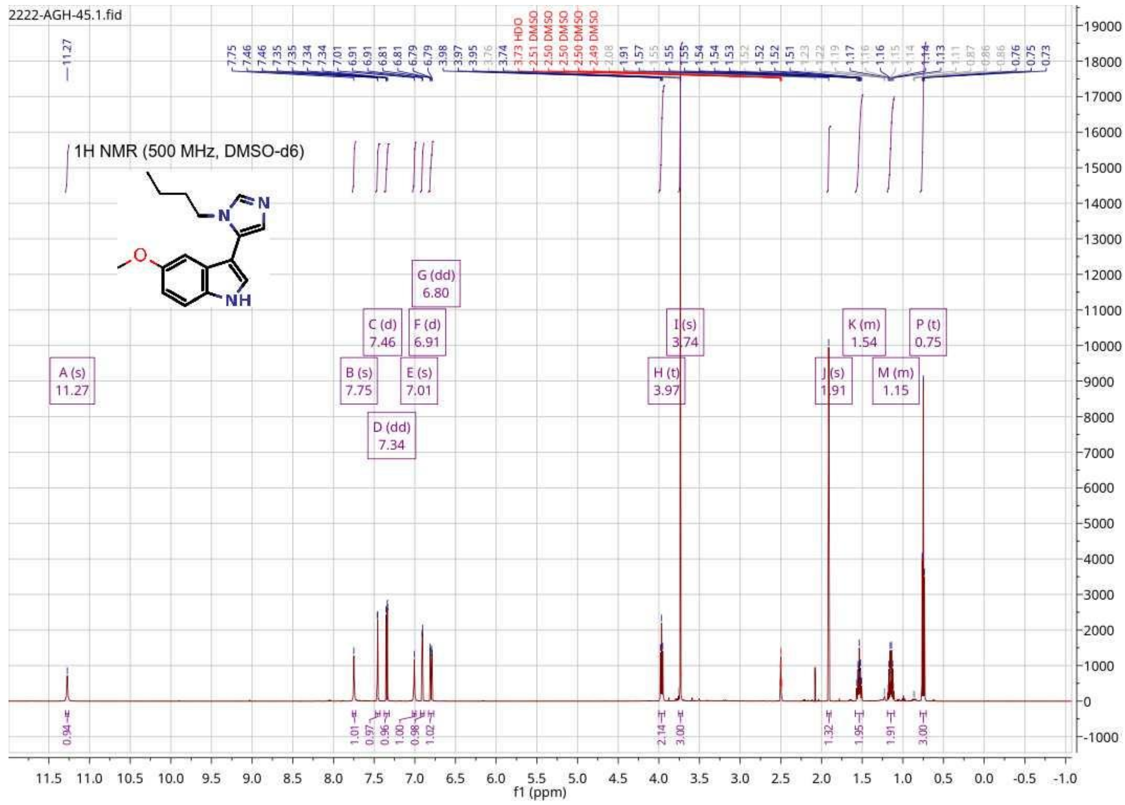
2222-AGH-45.1.fid
1H NMR (500 MHz, DMSO-d6)
A (s) 11.27
B (s) 7.75
C (d) 7.46
D (dd) 7.34
E (s) 7.01
F (d) 6.91
G (dd) 6.80
H (t) 3.97
I (s) 3.74
J (s) 1.91
K (m) 1.54
M (m) 1.15
P (t) 0.75
f1 (ppm)

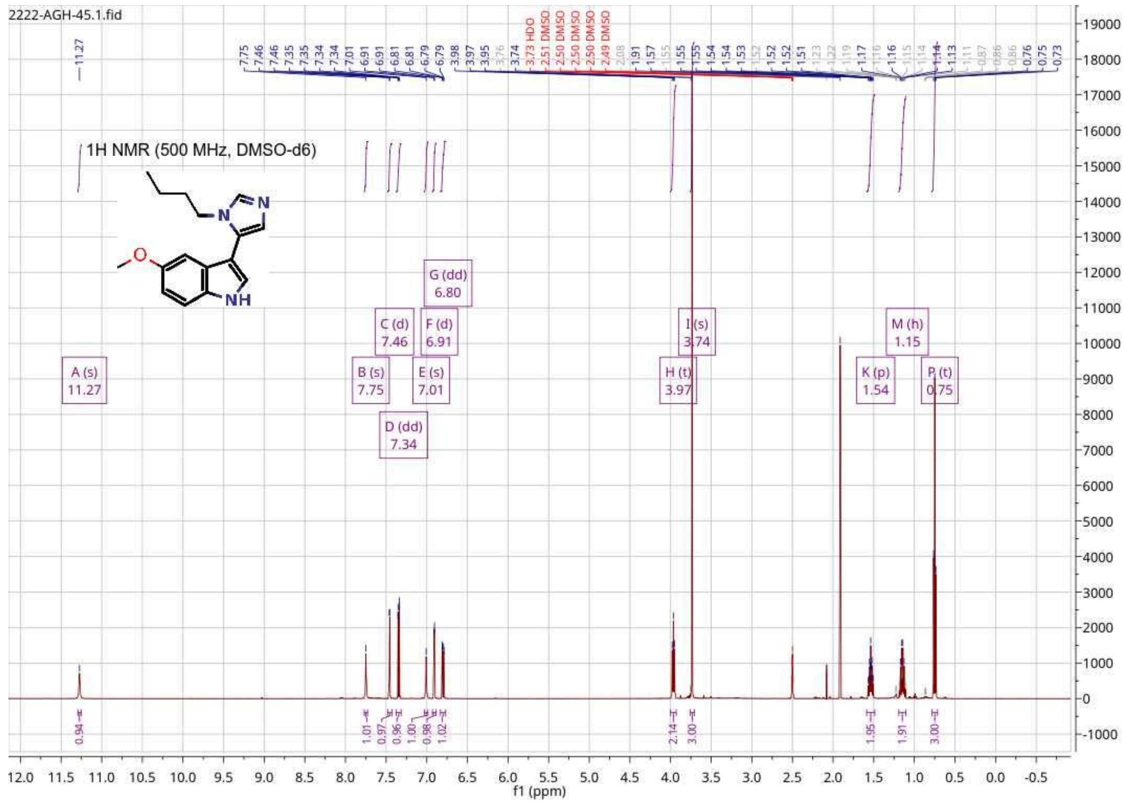
2222-AGH-45.1.fid
1H NMR (500 MHz, DMSO-d6)
A (s) 11.27
B (s) 7.75
C (d) 7.46
D (dd) 7.34
E (s) 7.01
F (d) 6.91
G (dd) 6.80
H (t) 3.97
I (s) 3.74
K (p) 1.54
M (h) 1.15
P (t) 0.75
f1 (ppm)

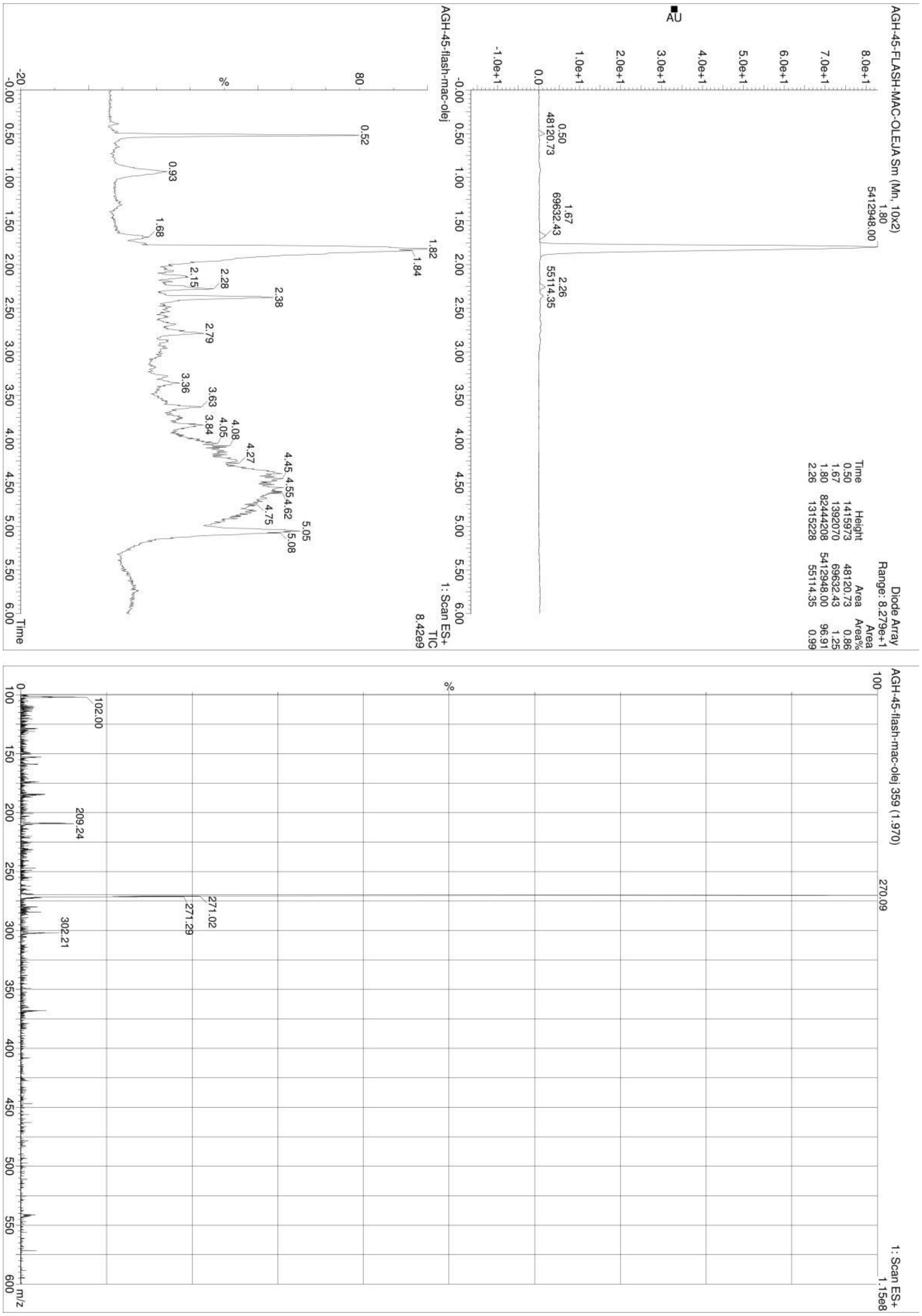

1h: 3-(1-cyclopropyl-1*H*-imidazol-5-yl)-5-methoxy-1*H*-indole (AH-427)

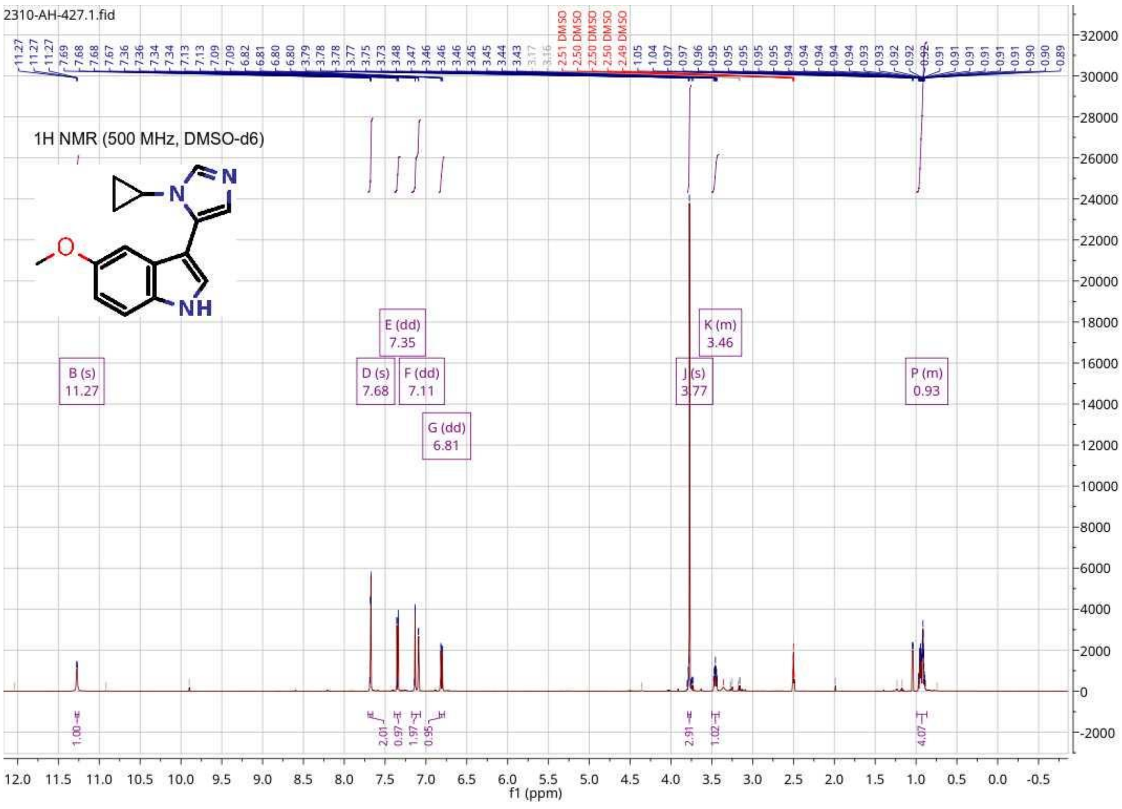

2310-AH-427.1.fid
1H NMR (500 MHz, DMSO-d6)
B (s) 11.27
D (s) 7.68
E (dd) 7.35
F (dd) 7.11
G (dd) 6.81
J (s) 3.77
K (m) 3.46
P (m) 0.93
f1 (ppm)

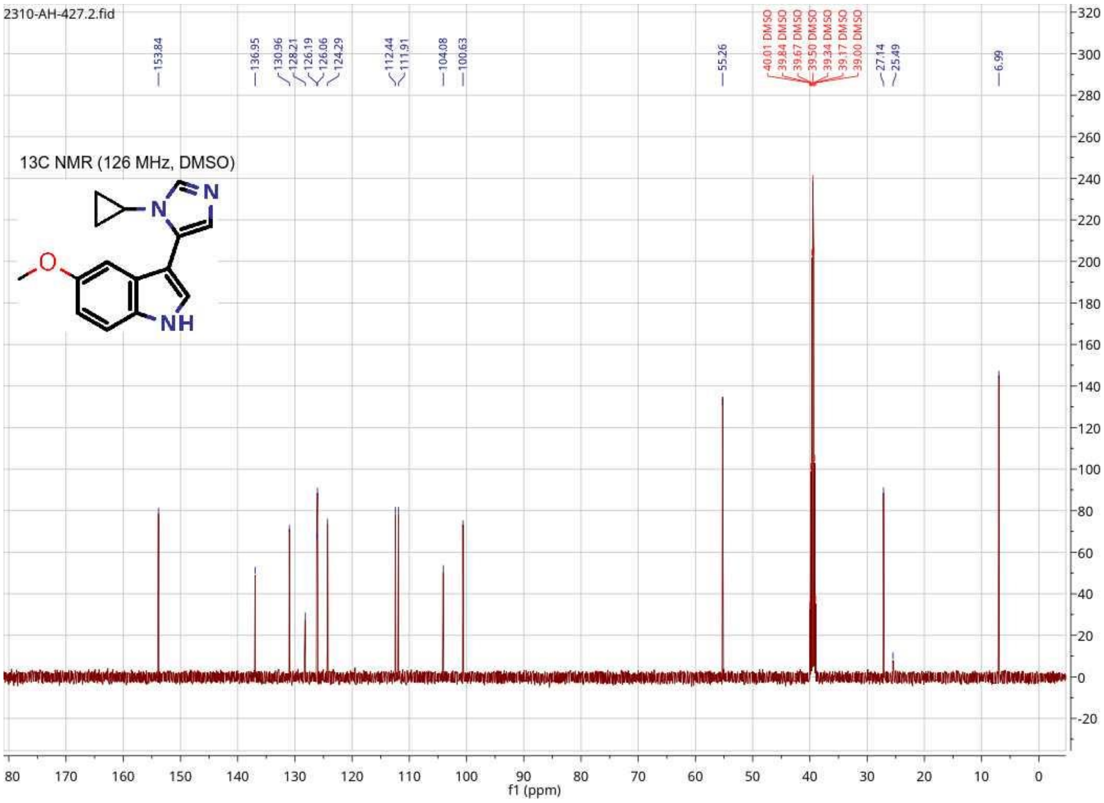

2310-AH-427.2.fid
13C NMR (126 MHz, DMSO)
153.84
136.95
130.96
128.21
126.19
126.06
124.29
112.44
111.91
104.08
100.63
55.26
27.14
25.49
6.99
f1 (ppm)

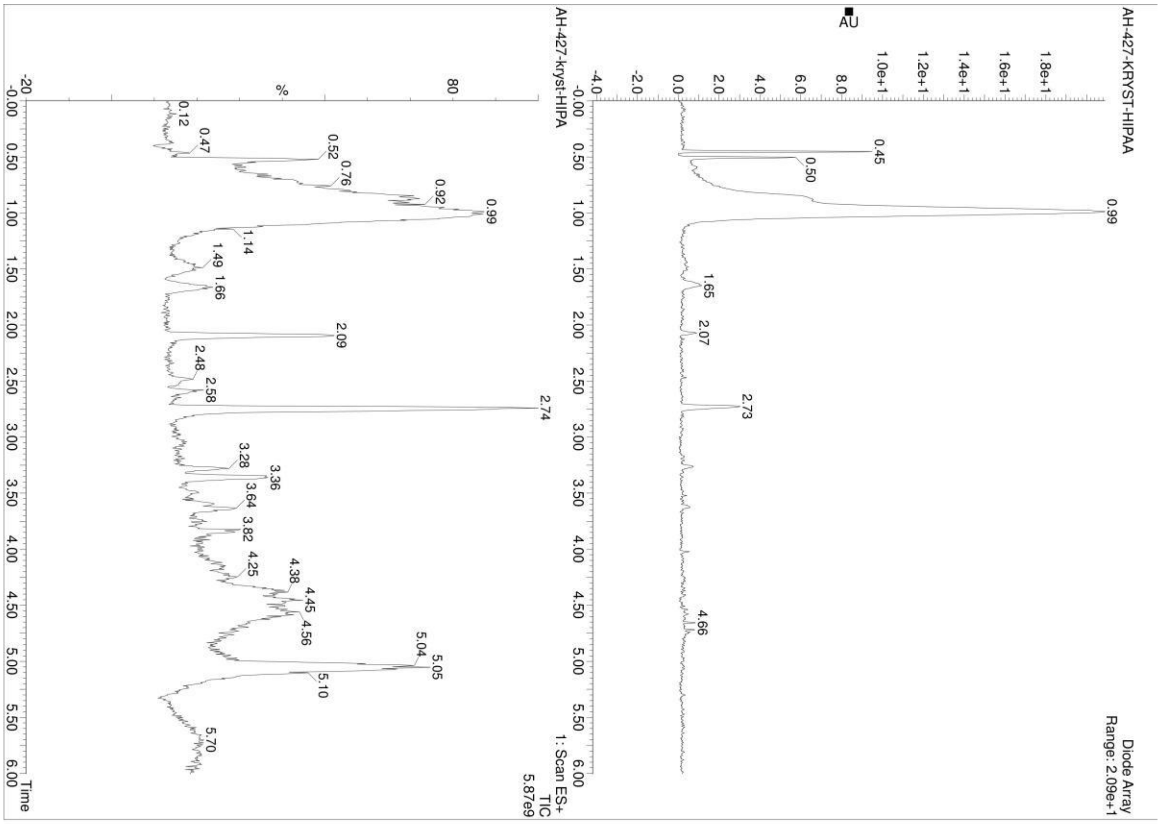

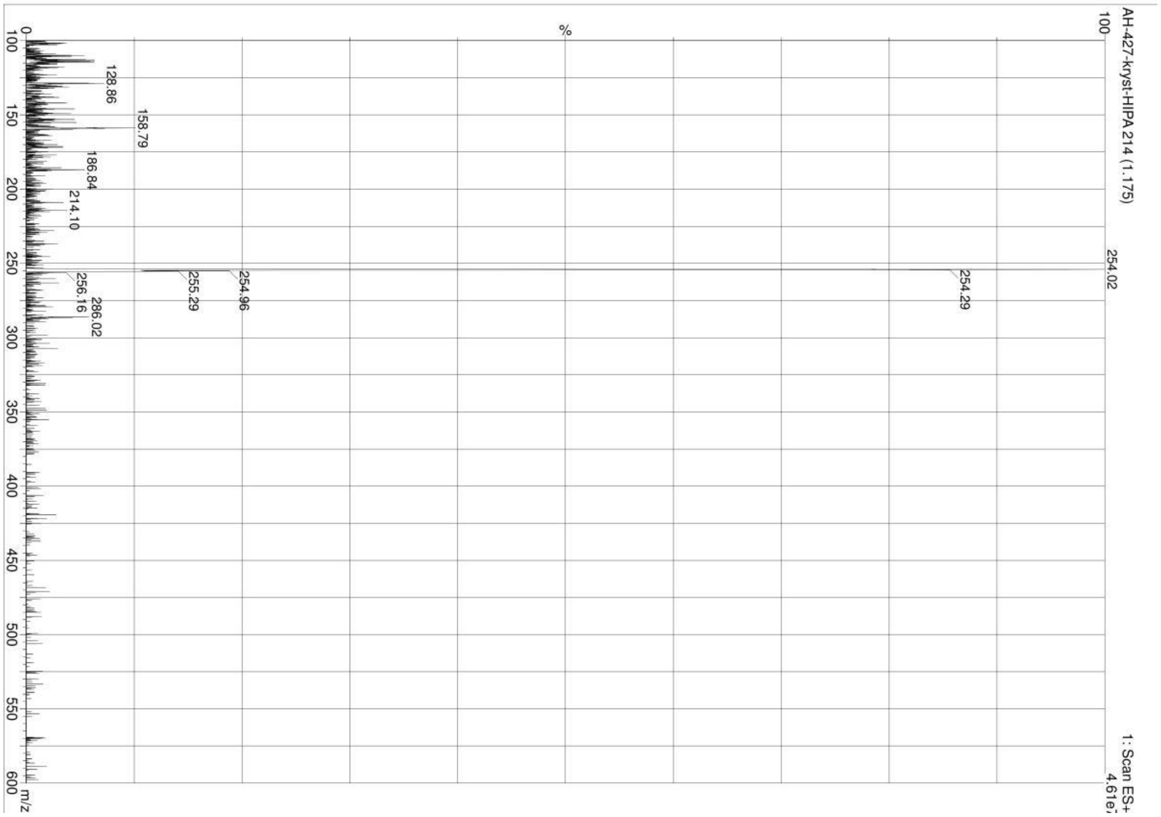

1i: 5-methoxy-3-[1-(prop-2-en-1-yl)-1*H*-imidazol-5-yl]-1*H*-indole (AGH-56)

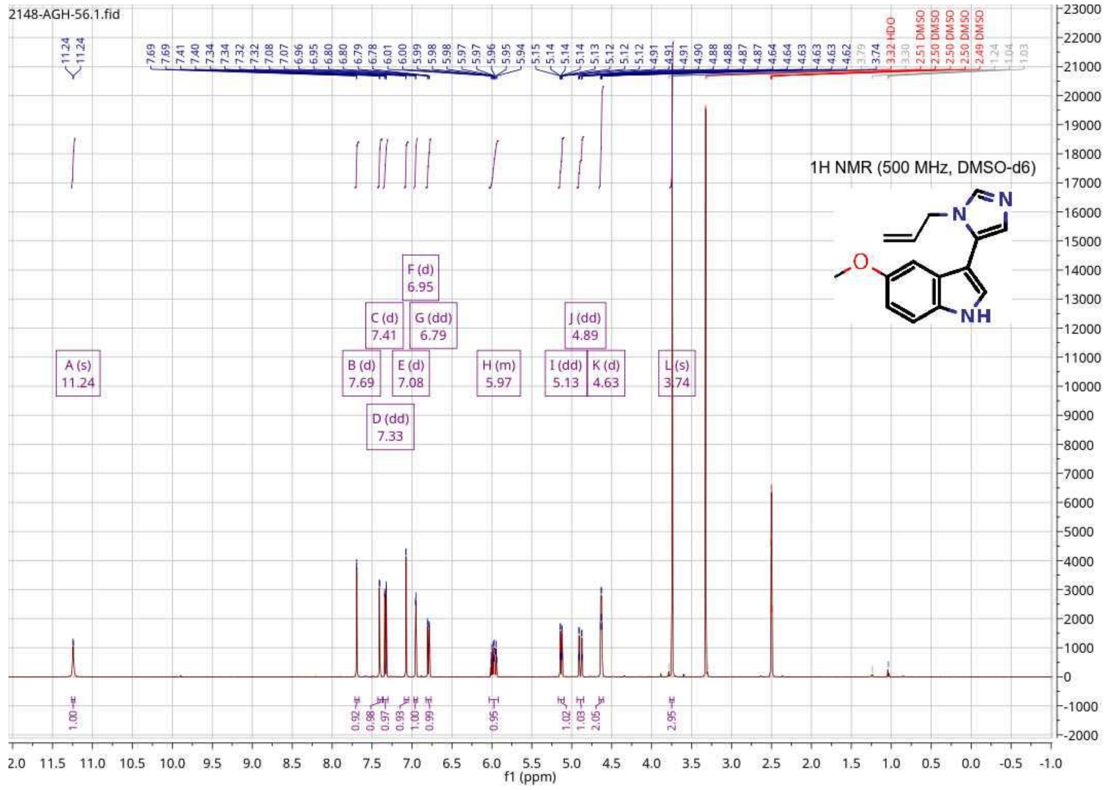
2148-AGH-56.1.fid
1H NMR (500 MHz, DMSO-d6)
A (s) 11.24
B (d) 7.69
C (d) 7.41
D (dd) 7.33
E (d) 7.08
F (d) 6.95
G (dd) 6.79
H (m) 5.97
I (dd) 5.13
J (dd) 4.89
K (d) 4.63
L (s) 3.74
f1 (ppm)

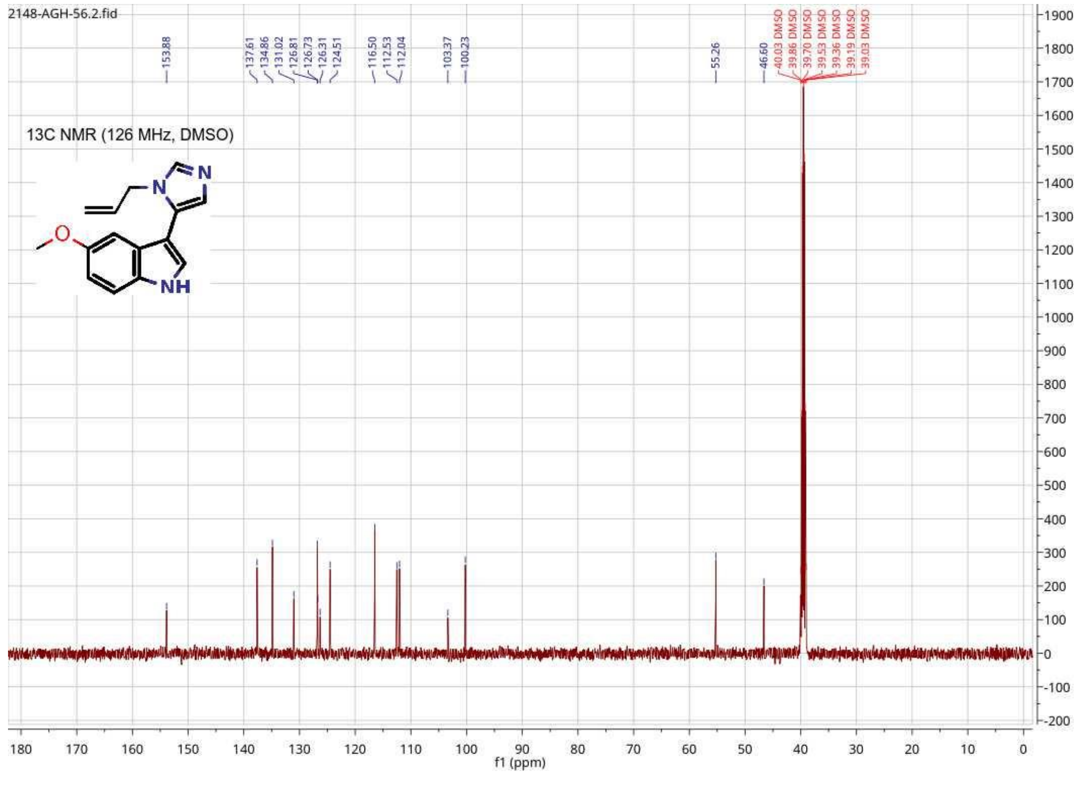
2148-AGH-56.2.fid
13C NMR (126 MHz, DMSO)
153.88
137.61
134.86
131.02
126.81
126.73
126.31
124.51
116.50
112.53
112.04
103.37
100.23
55.26
46.60
f1 (ppm)

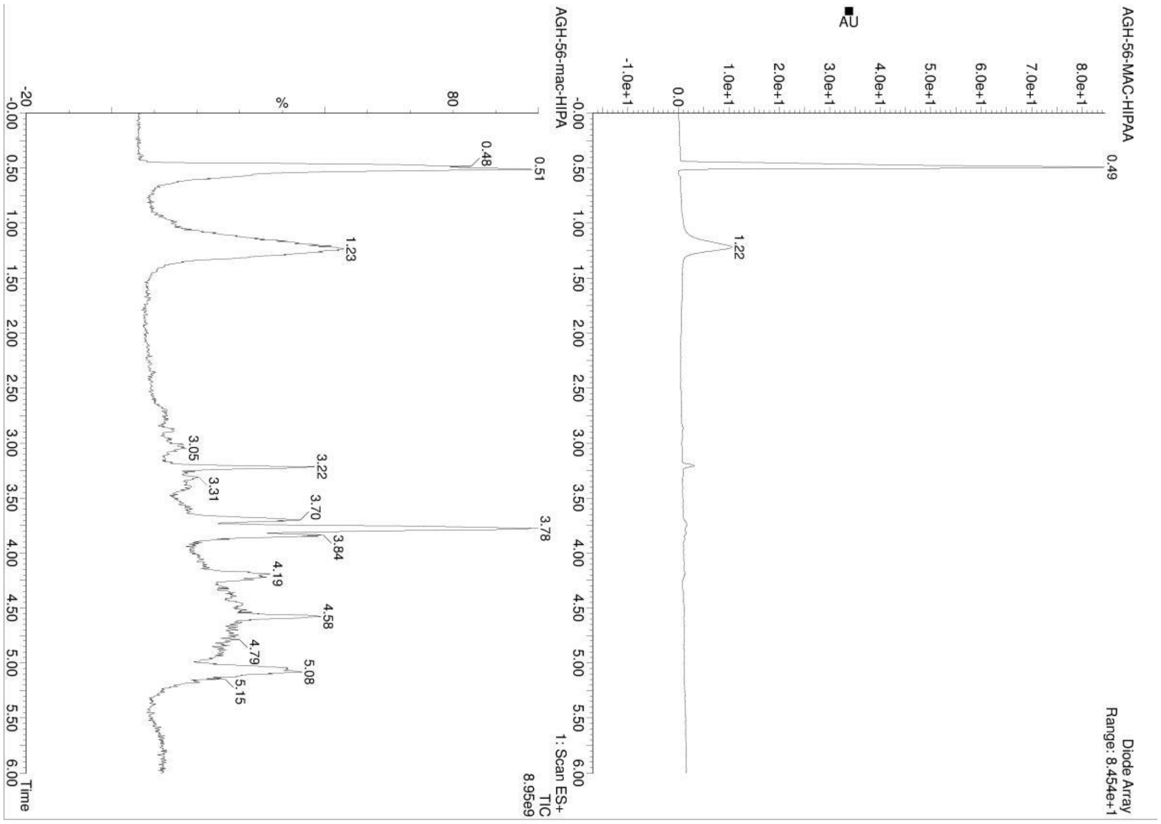

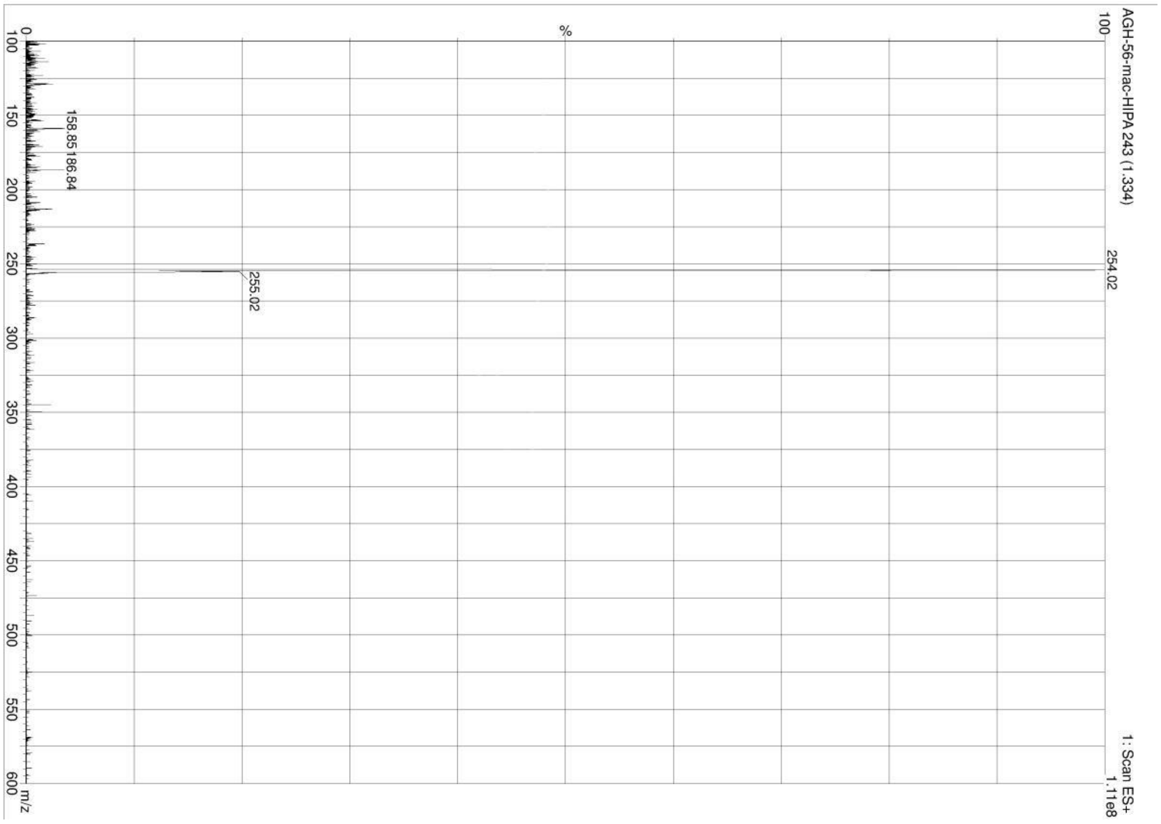

1j: 6-bromo-3-(1-ethyl-1*H*-imidazol-5-yl)-1*H*-indole (AGH-76)

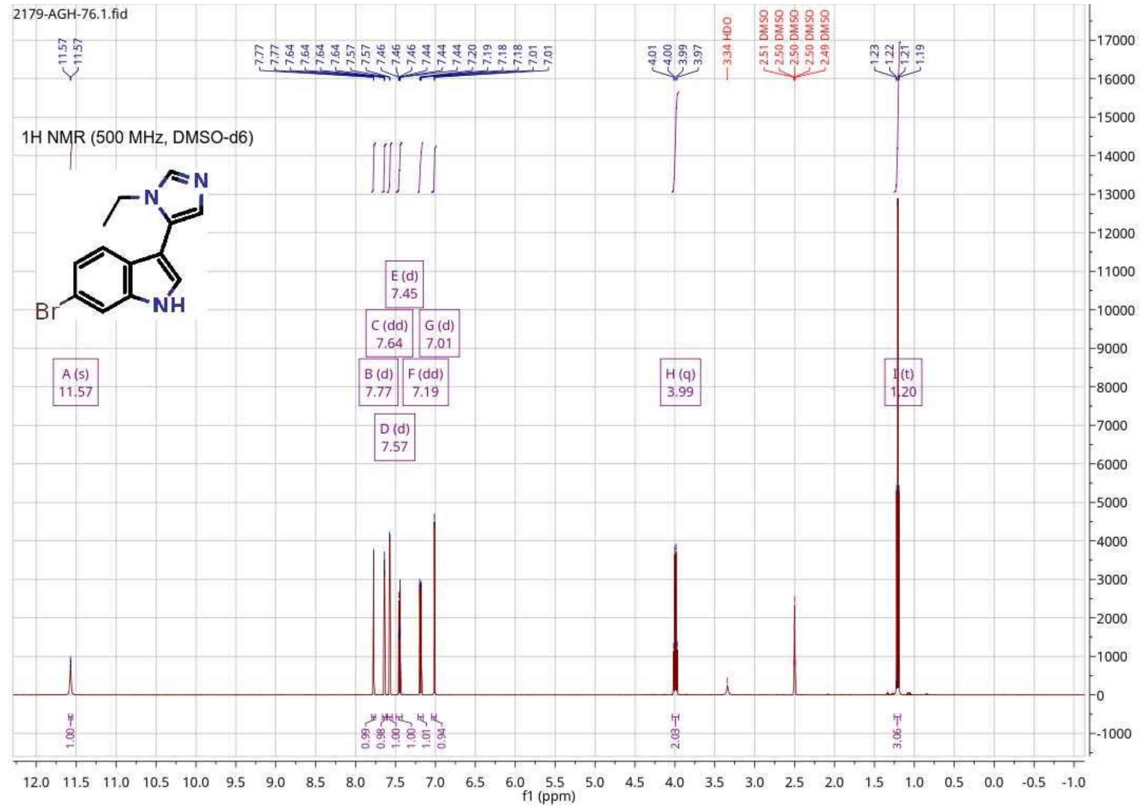
2179-AGH-76.1.fid
1H NMR (500 MHz, DMSO-d6)
11.57
11.57
7.77
7.77
7.64
7.64
7.64
7.64
7.57
7.57
7.46
7.46
7.44
7.44
7.44
7.20
7.19
7.18
7.01
7.01
4.01
4.00
3.99
3.97
3.34 HDO
2.51 DMSO
2.50 DMSO
2.50 DMSO
2.50 DMSO
2.49 DMSO
1.23
1.22
1.21
1.19
A (s) 11.57
B (d) 7.77
C (dd) 7.64
D (d) 7.57
E (d) 7.45
F (dd) 7.19
G (d) 7.01
H (q) 3.99
I (t) 1.20
1.00
0.99
0.98
1.00
1.00
1.01
0.94
2.03
3.06
Br
NH
N
N
f1 (ppm)

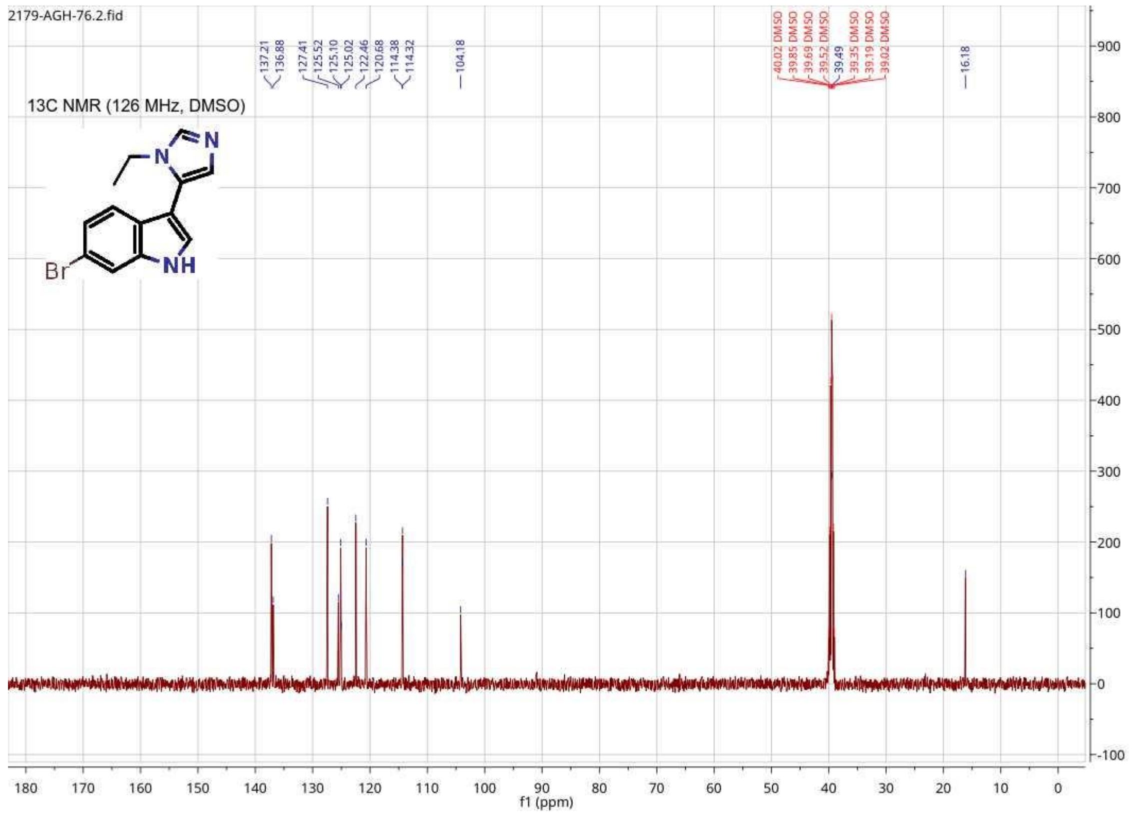
2179-AGH-76.2.fid
13C NMR (126 MHz, DMSO)
137.21
136.88
127.41
125.52
125.10
125.02
122.46
120.68
114.38
114.32
104.18
40.02 DMSO
39.85 DMSO
39.69 DMSO
39.52 DMSO
39.49
39.35 DMSO
39.19 DMSO
39.02 DMSO
16.18
Br
NH
N
N
f1 (ppm)

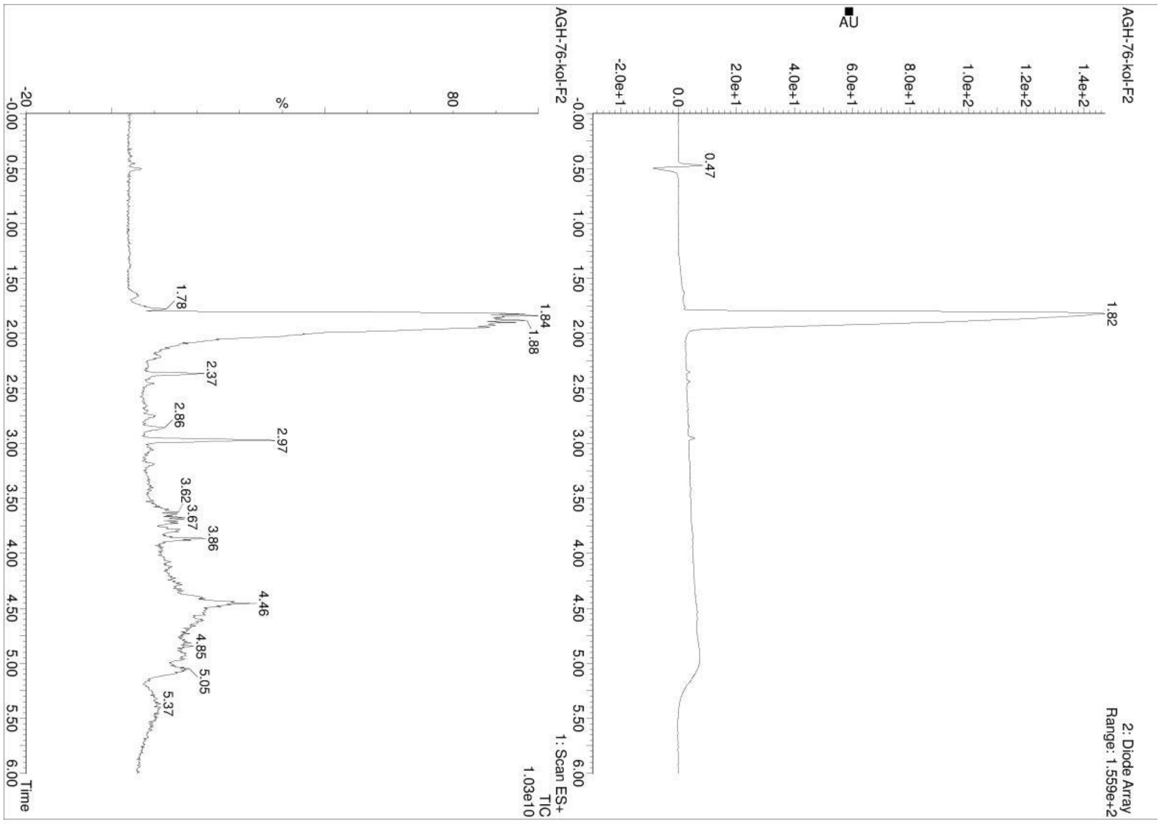

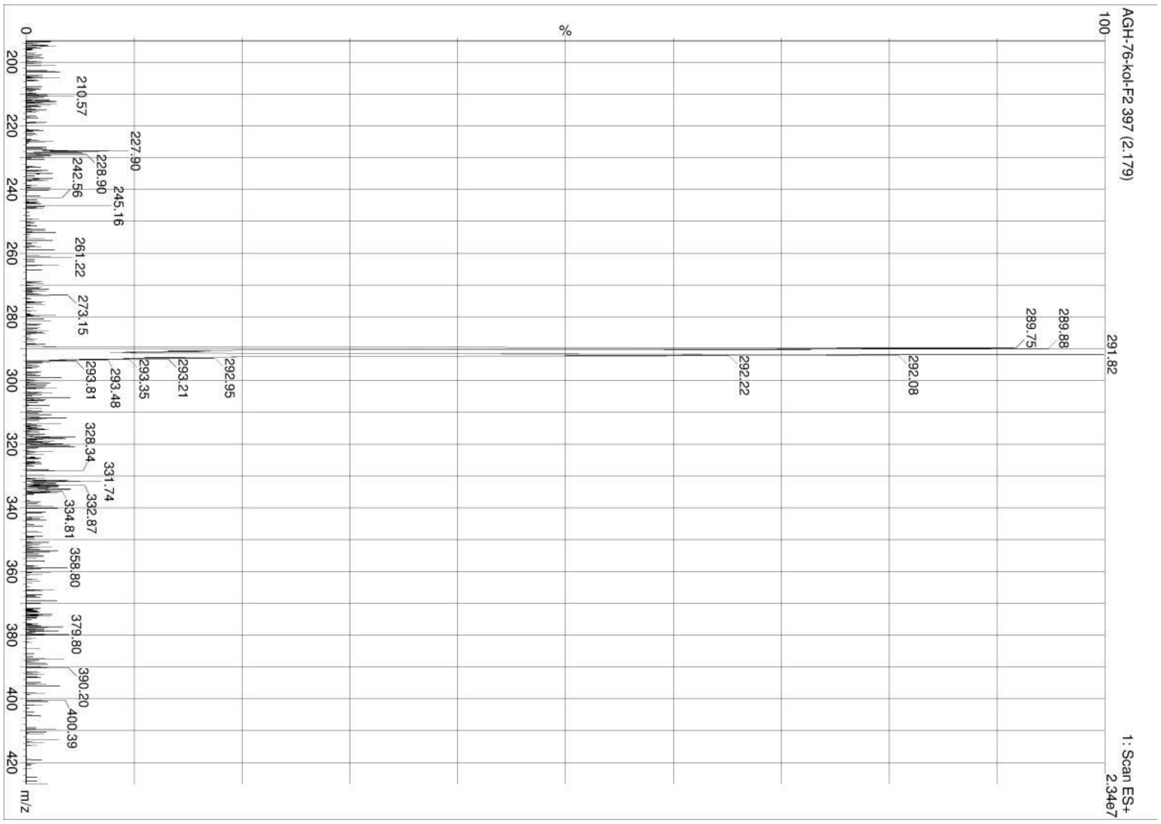

1k: 4-bromo-3-(1-ethyl-1*H*-imidazol-5-yl)-1*H*-indole (AGH-84)

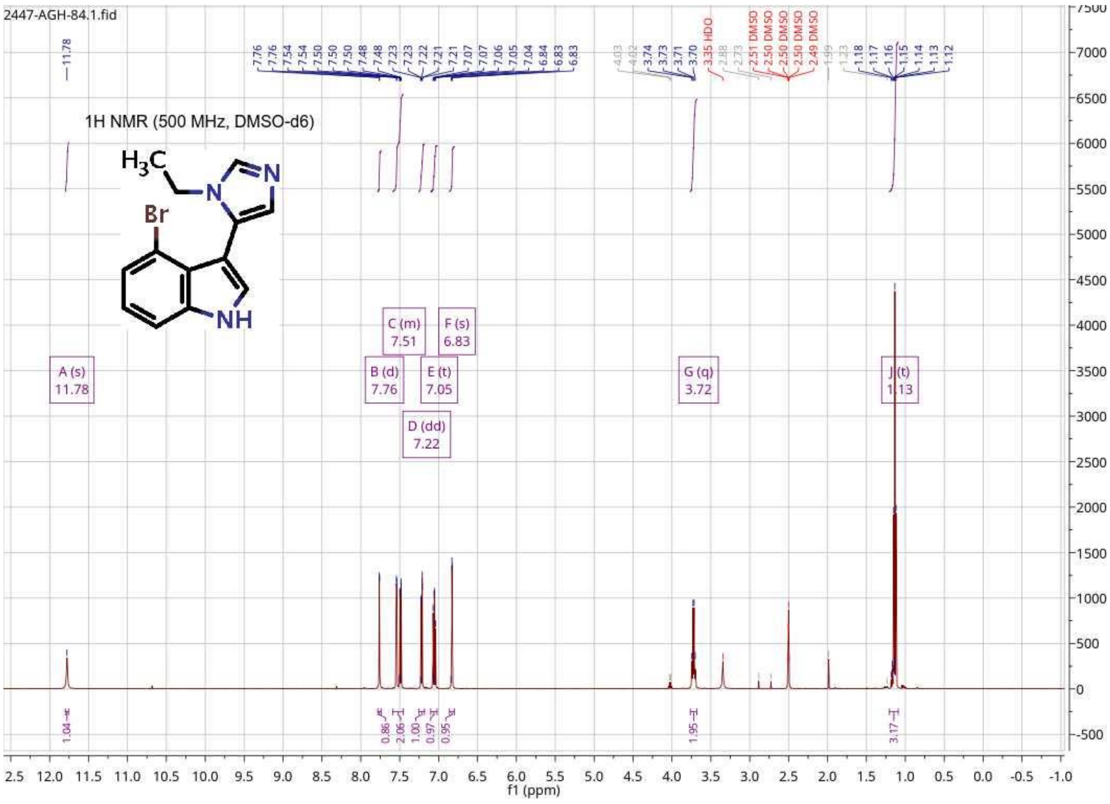
2447-AGH-84.1.fid
1H NMR (500 MHz, DMSO-d6)
A (s) 11.78
B (d) 7.76
C (m) 7.51
D (dd) 7.22
E (t) 7.05
F (s) 6.83
G (q) 3.72
J (t) 1.13
3.35 HDO
2.50 DMSO
f1 (ppm)

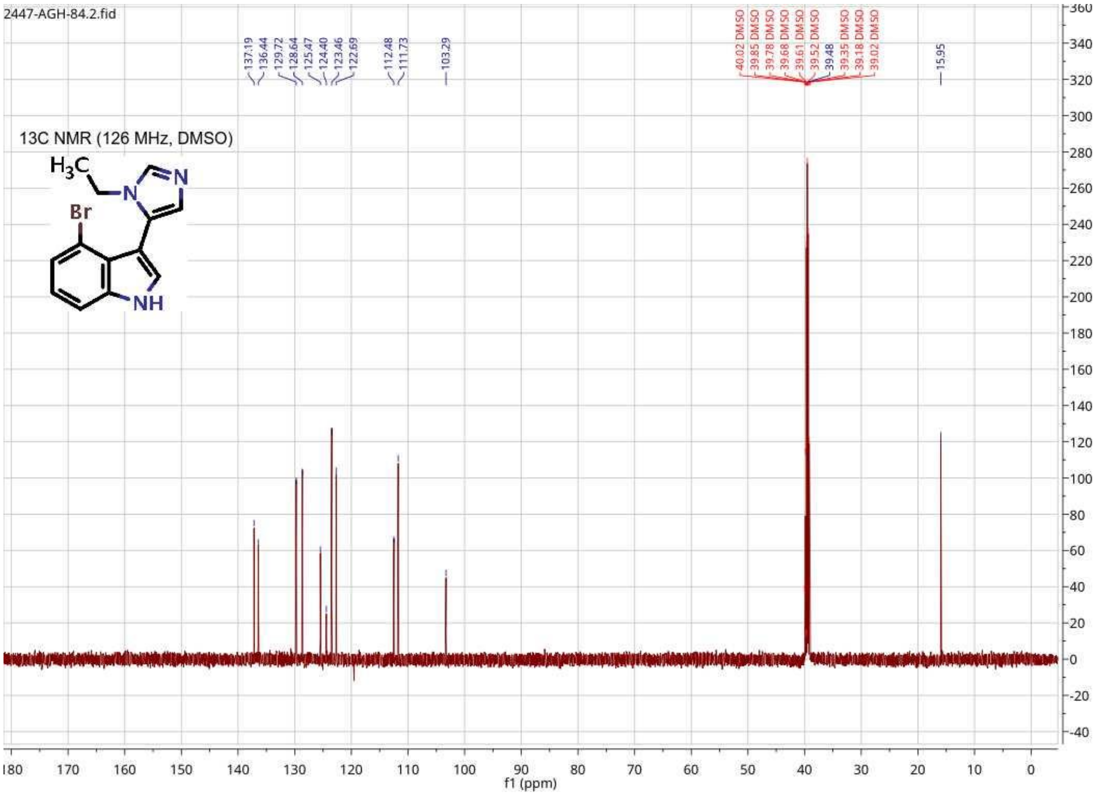
2447-AGH-84.2.fid
13C NMR (126 MHz, DMSO)
137.19
136.44
129.72
128.64
125.47
124.40
123.46
122.69
112.48
111.73
103.29
15.95
f1 (ppm)

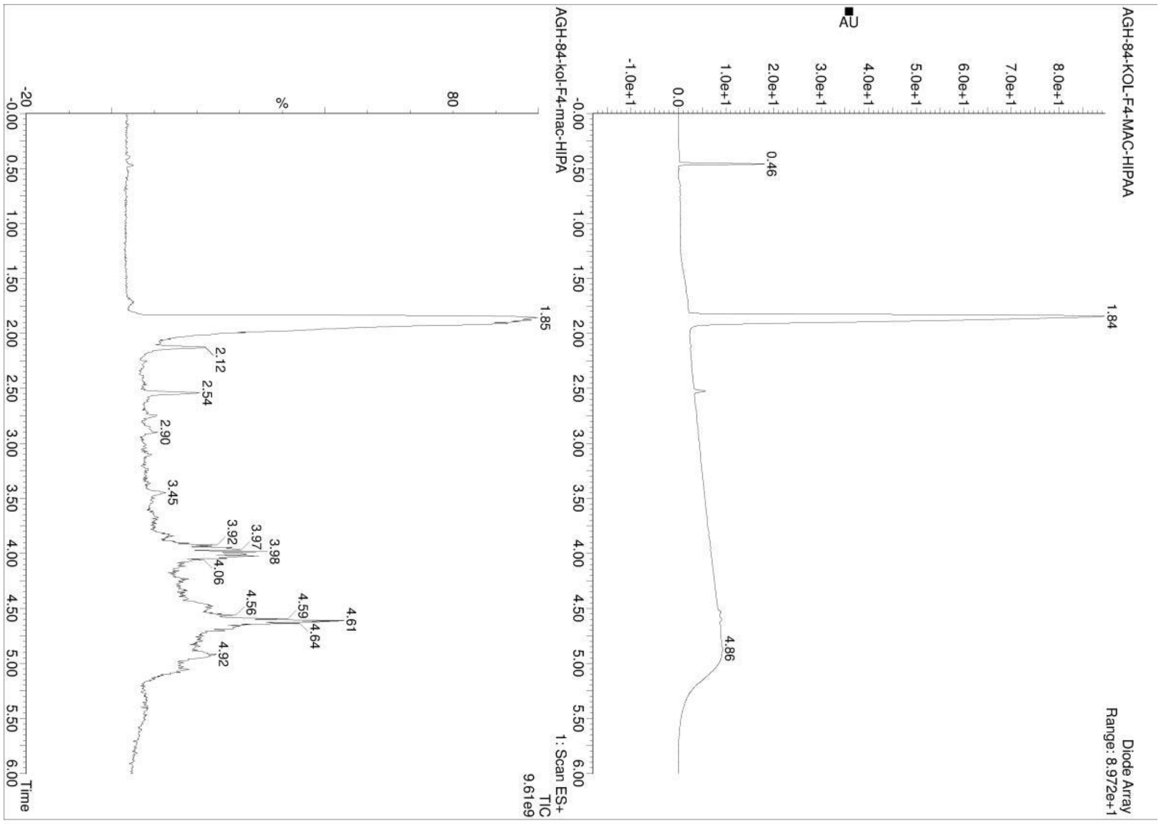

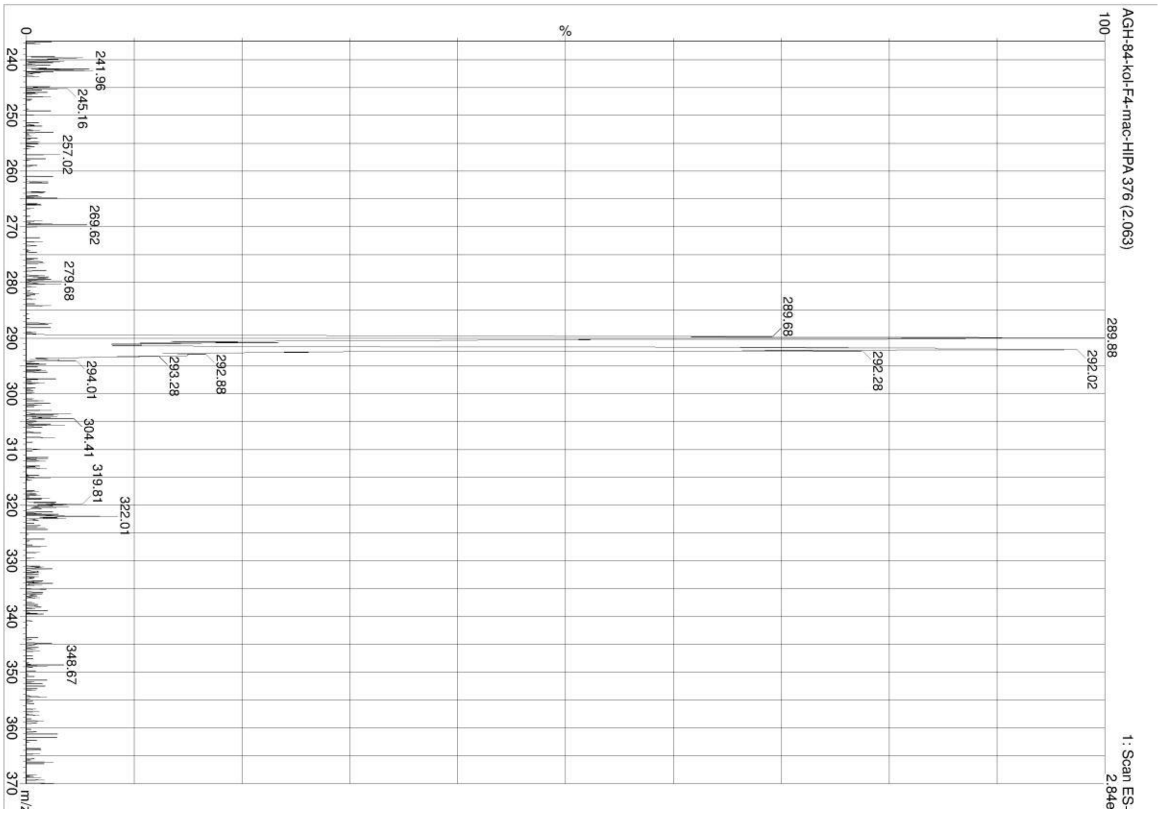

1l: 3-(1-ethyl-1*H*-imidazol-5-yl)-5-fluoro-1*H*-indole (AGH-80)

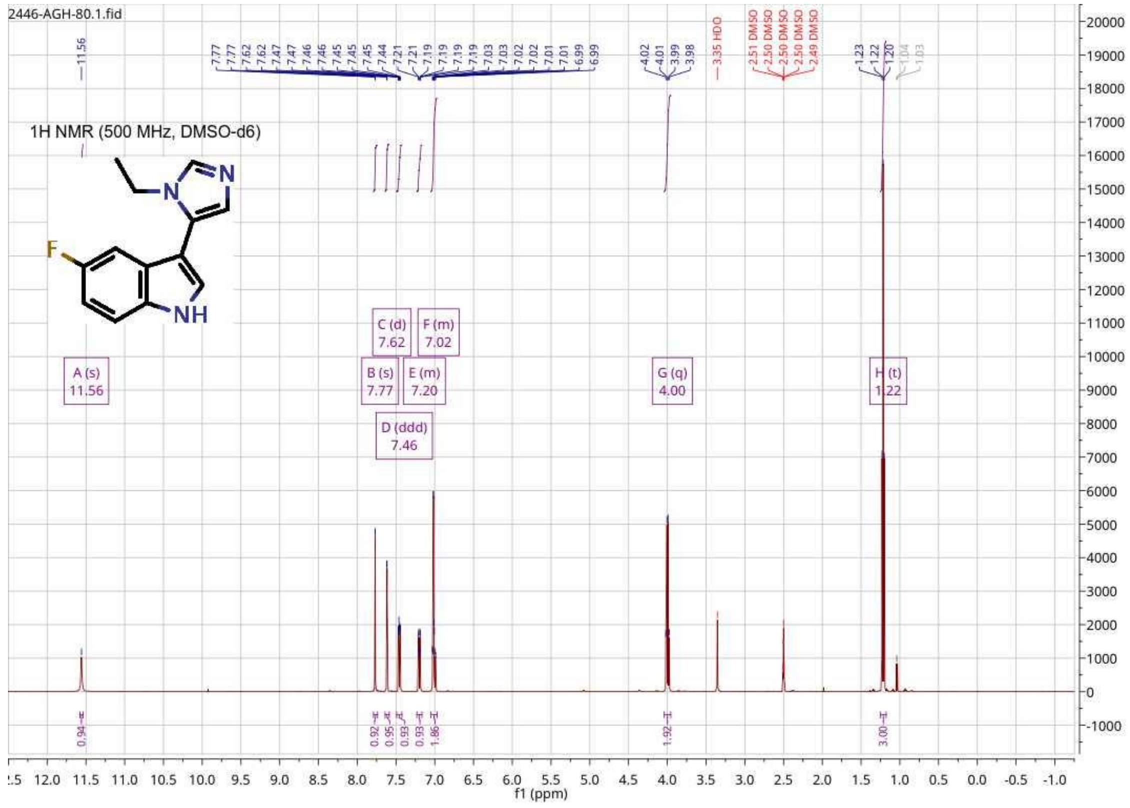
2446-AGH-80.1.fid
1H NMR (500 MHz, DMSO-d6)
A (s) 11.56
B (s) 7.77
C (d) 7.62
D (ddd) 7.46
E (m) 7.20
F (m) 7.02
G (q) 4.00
H (t) 1.22
3.35 HDO
2.50 DMSO
NH
N
F
f1 (ppm)

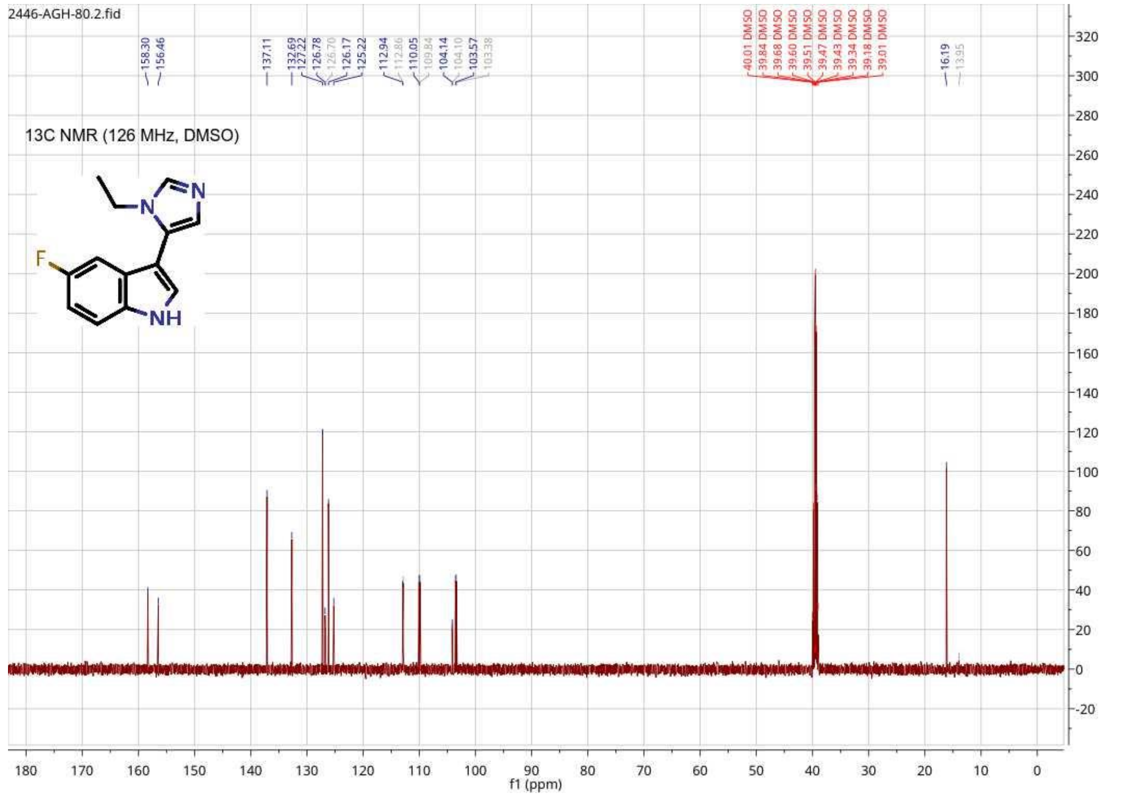
2446-AGH-80.2.fid
13C NMR (126 MHz, DMSO)
158.30
156.46
137.11
132.69
127.22
126.78
126.17
125.22
112.94
110.05
104.14
103.57
16.19
NH
N
F
f1 (ppm)

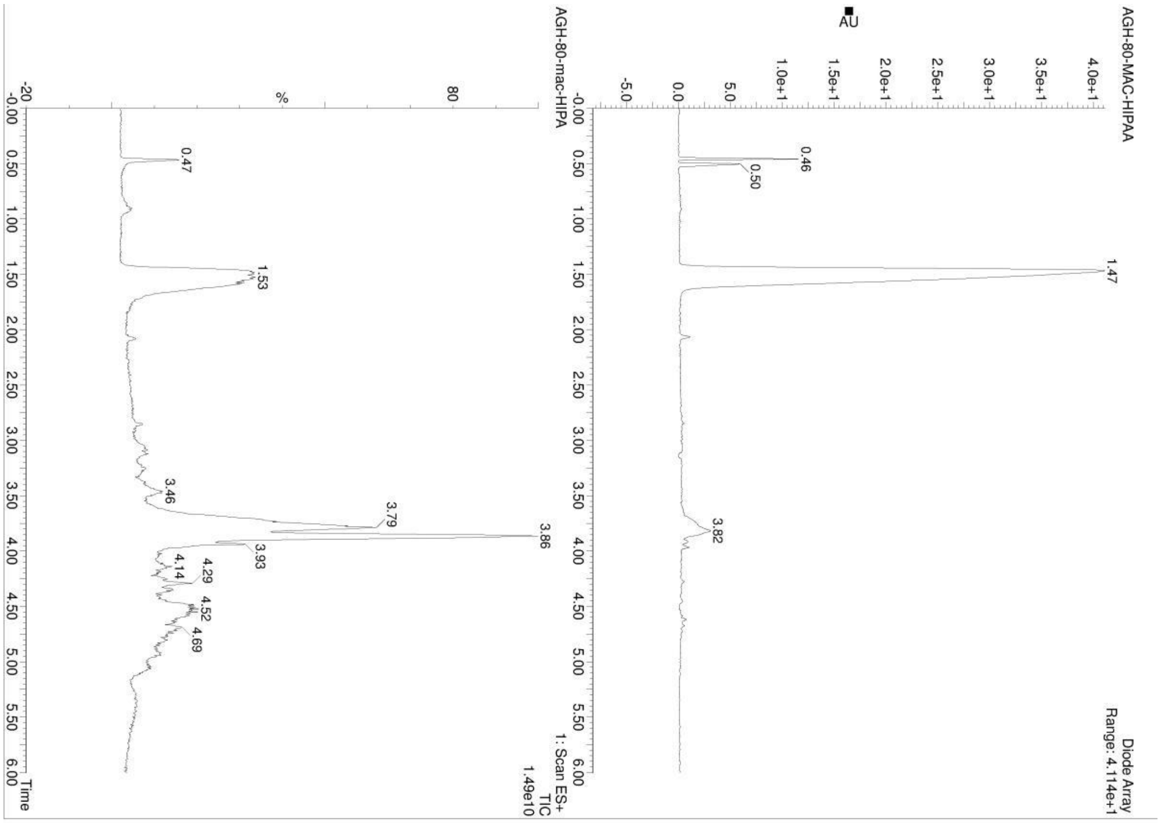

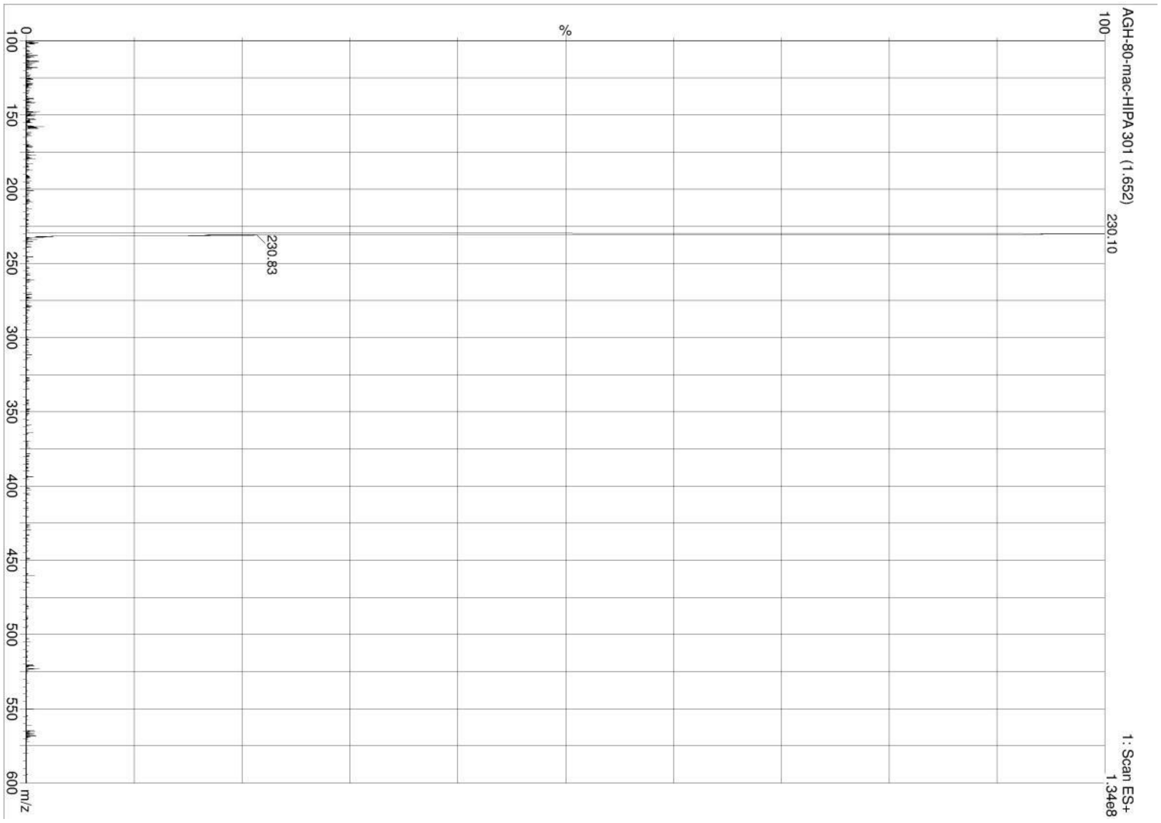

1m: 5-chloro-3-(1-ethyl-1H-imidazol-5-yl)-1H-indole (AGH-79)

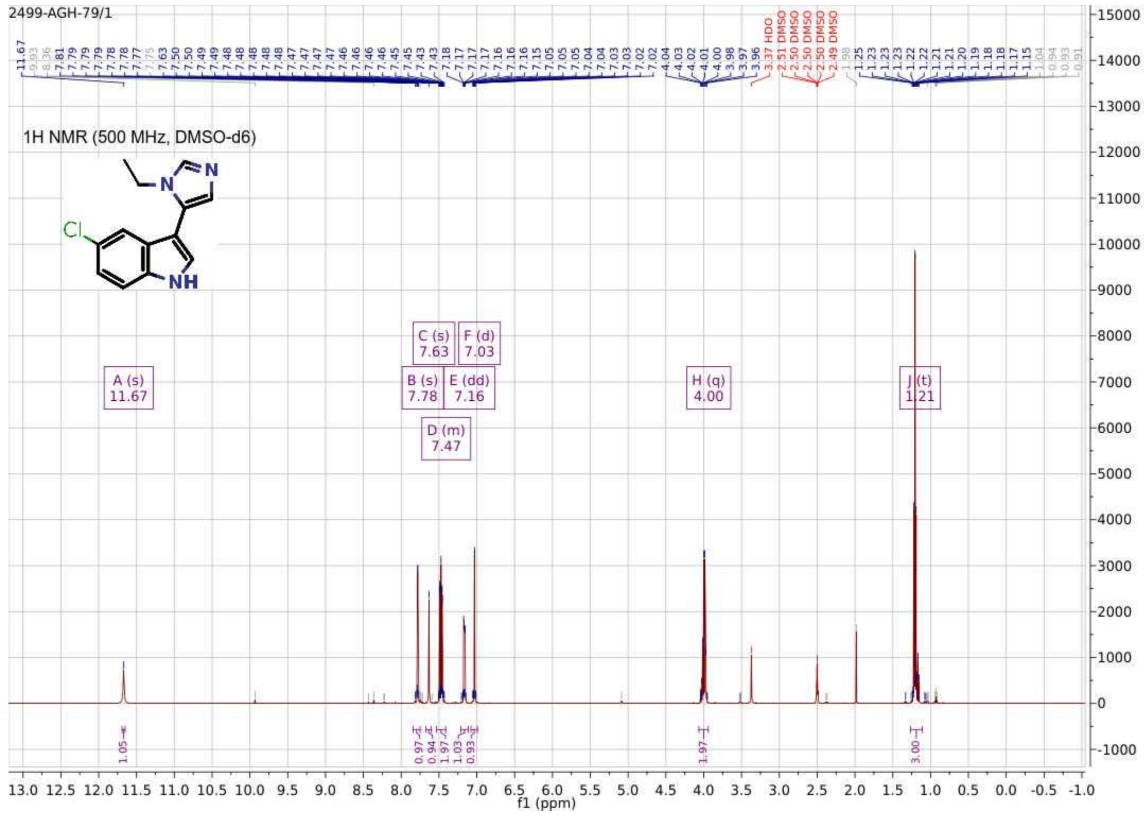
2499-AGH-79/1
1H NMR (500 MHz, DMSO-d6)
Cl
N
N
NH
A (s)
11.67
B (s)
7.78
C (s)
7.63
D (m)
7.47
E (dd)
7.16
F (d)
7.03
H (q)
4.00
J (t)
1.21
f1 (ppm)

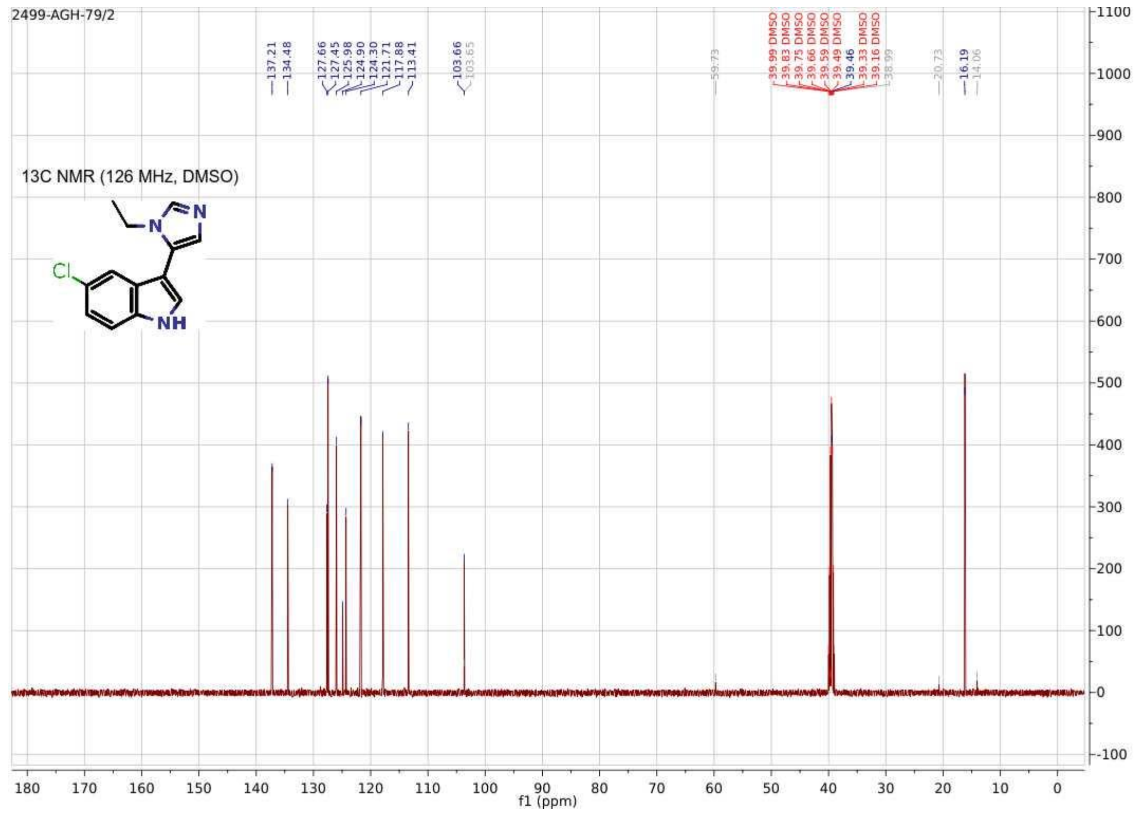
2499-AGH-79/2
13C NMR (126 MHz, DMSO)
Cl
N
N
NH
137.21
134.48
127.66
127.45
125.98
124.90
124.30
121.71
117.88
113.41
103.66
16.19
f1 (ppm)

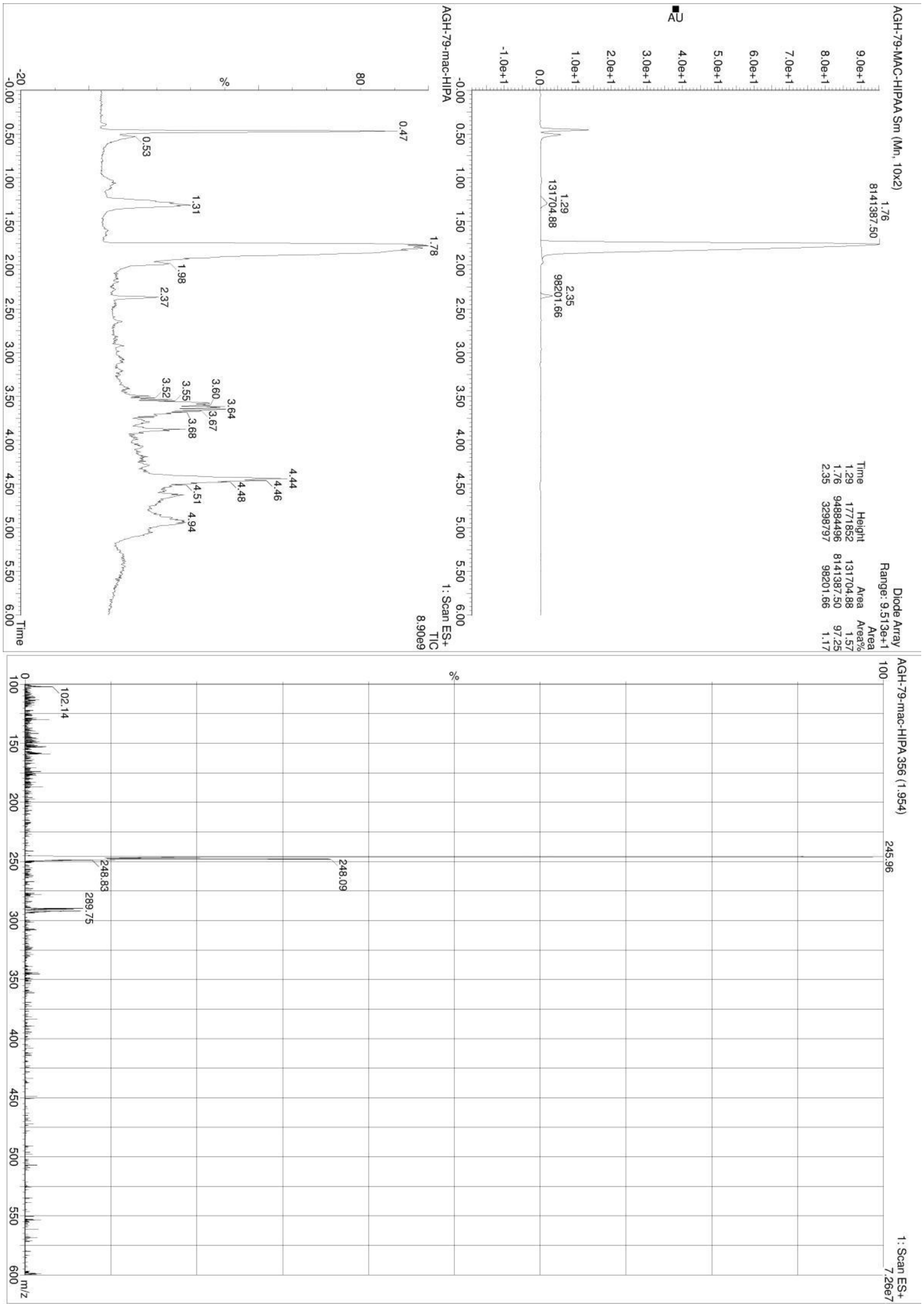
AGH-79-MAC-HIPAA Sm (Mn, 10x2)
Diode Array
Range: 9.513e+1
AU
Time Height Area Area%
1.29 1771852 131704.88 1.57
1.76 94884496 8141387.50 97.25
2.35 3298797 98201.66 1.17
1.76
8141387.50
1.29
131704.88
2.35
98201.66
AGH-79-mac-HIPA
1: Scan ES+
TIC
8.90e9
0.47
0.53
1.31
1.78
1.98
2.37
3.52
3.55
3.60
3.64
3.67
3.68
4.44
4.46
4.48
4.51
4.94
Time
AGH-79-mac-HIPA 356 (1.954)
1: Scan ES+
7.26e7
245.96
248.09
248.83
289.75
102.14
m/z

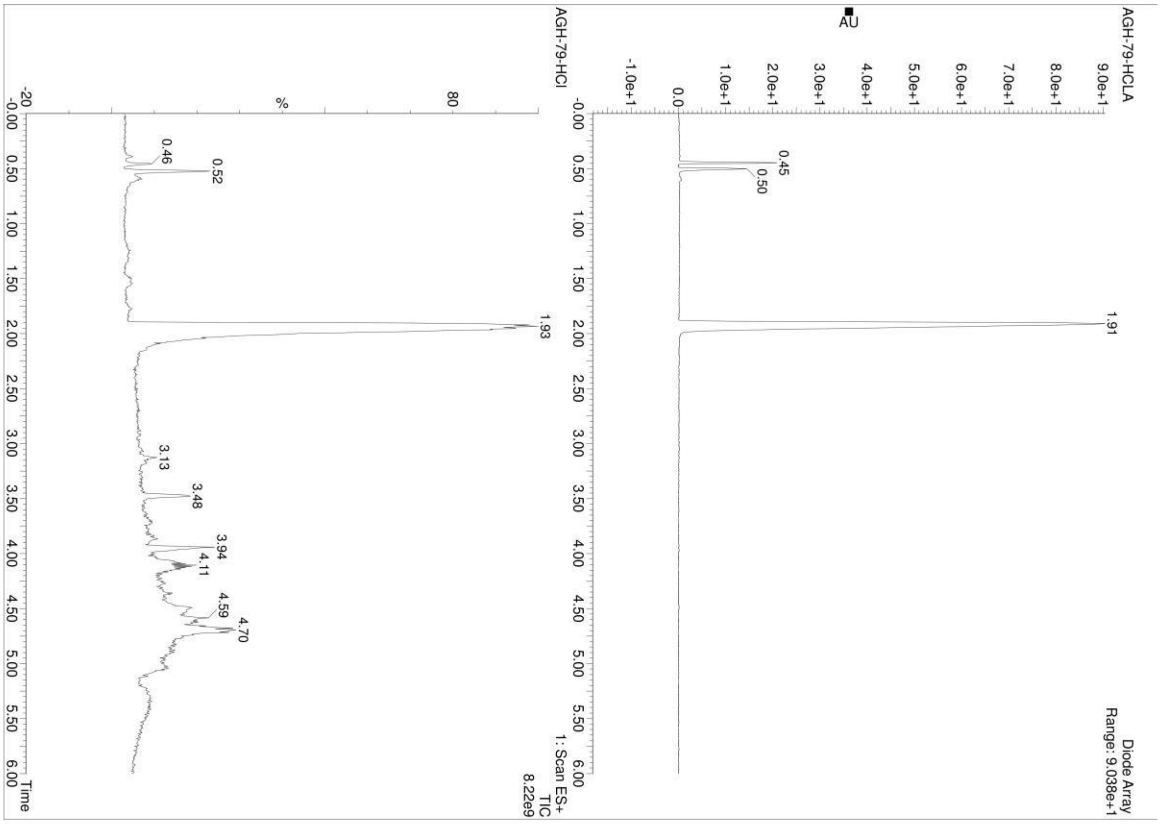

AGH-79-HCLA
Diode Array
Range: 9.038e+1
AU
1.91
0.45
0.50
AGH-79-HCl
1: Scan ES+
TIC
8.22e9
1.93
0.46
0.52
3.13
3.48
3.94
4.11
4.59
4.70
Time

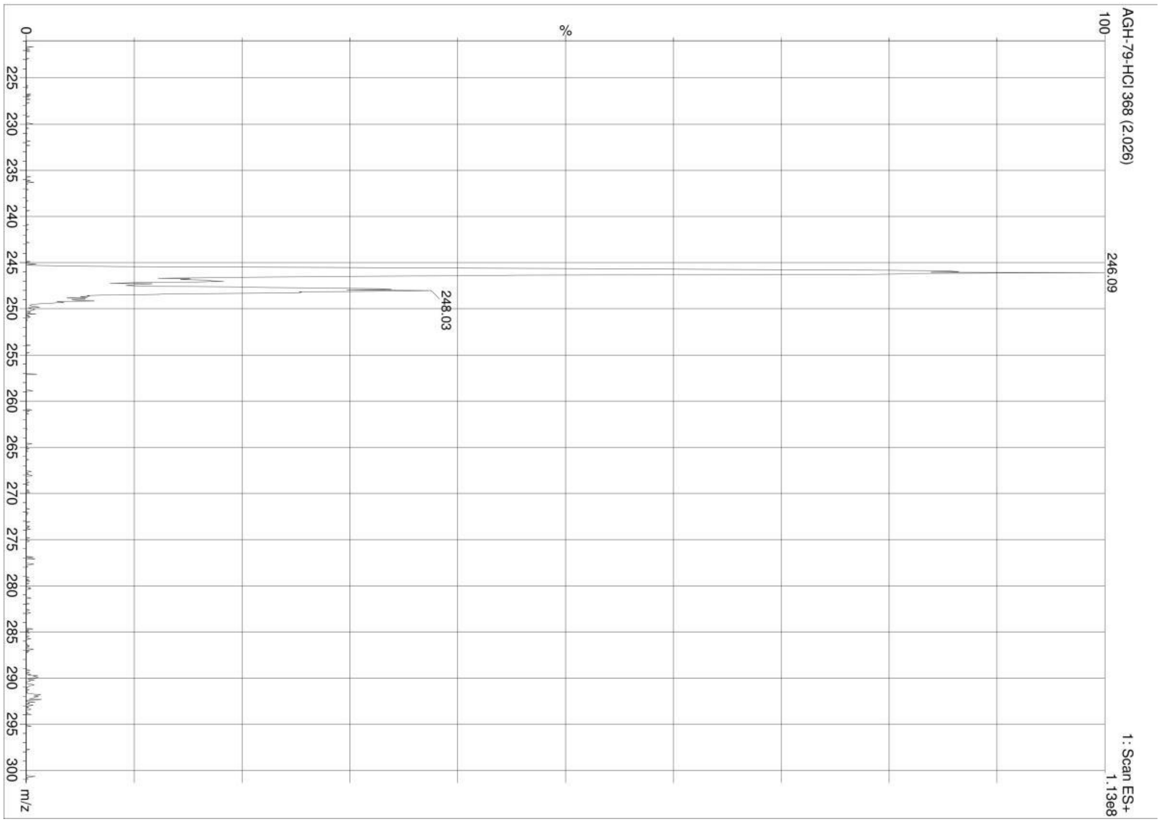

AGH-79-HCl 368 (2.026)
1: Scan ES+
1.13e8
246.09
248.03
m/z

1n: 5-bromo-3-(1-ethyl-1*H*-imidazol-5-yl)-1*H*-indole (AGH-75)

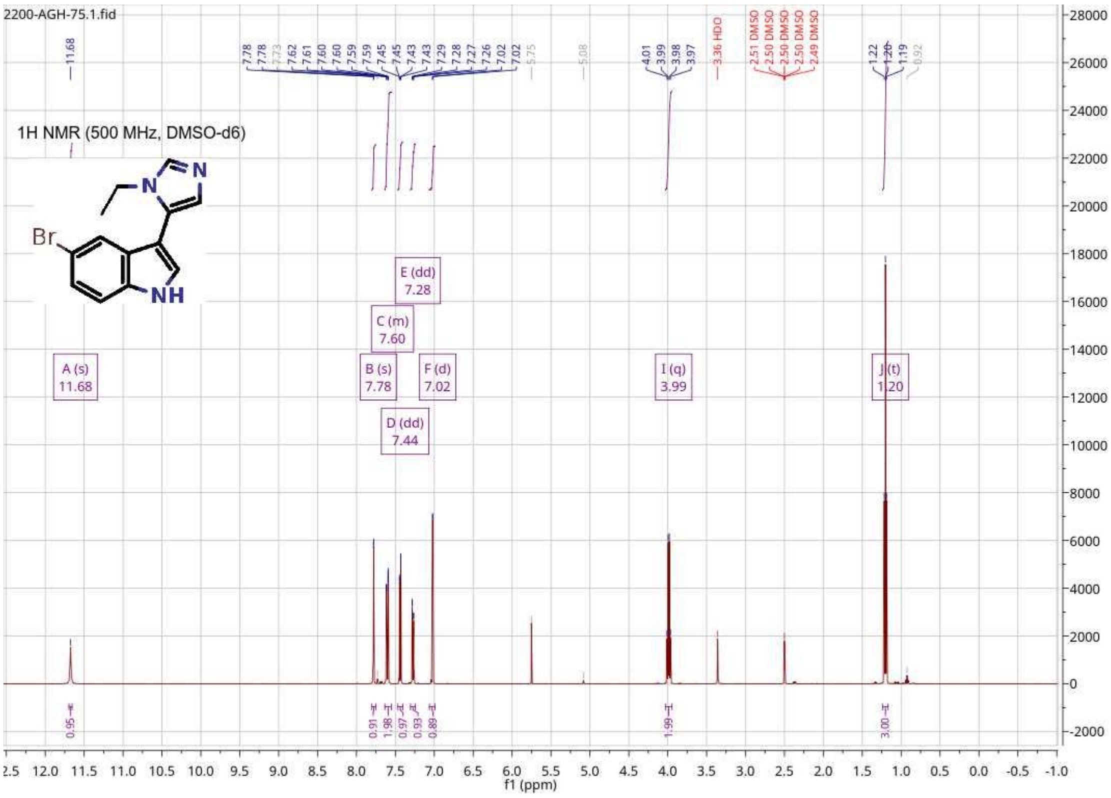

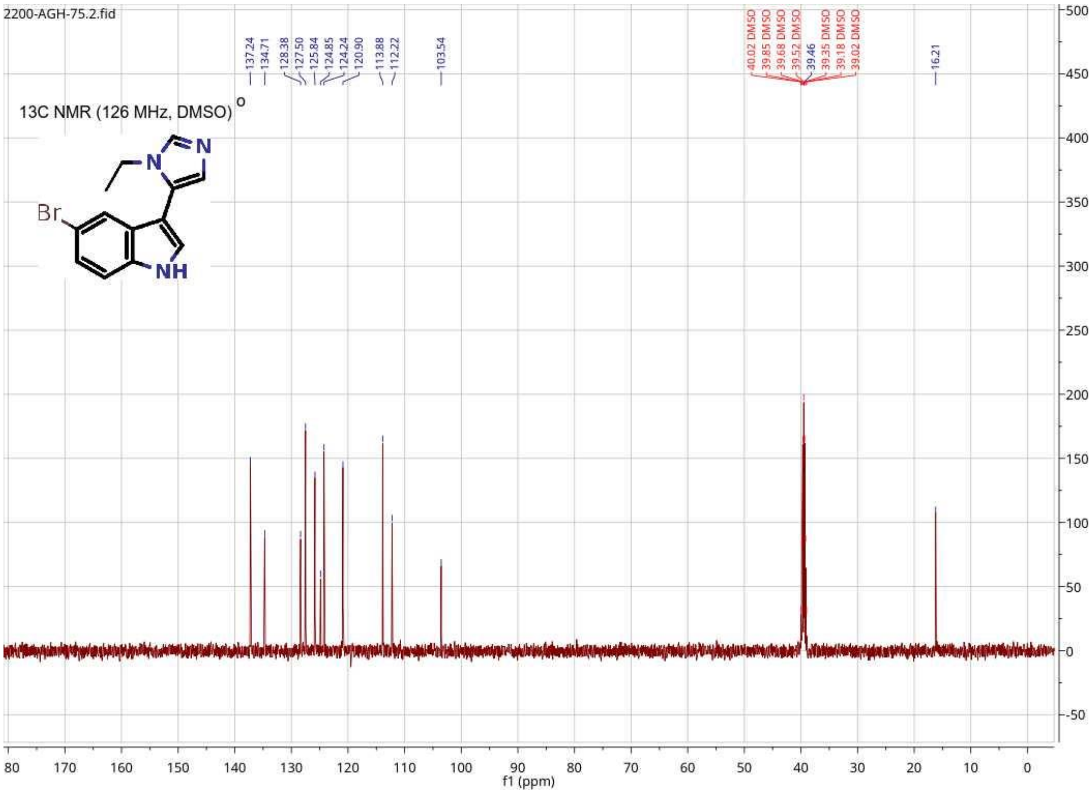

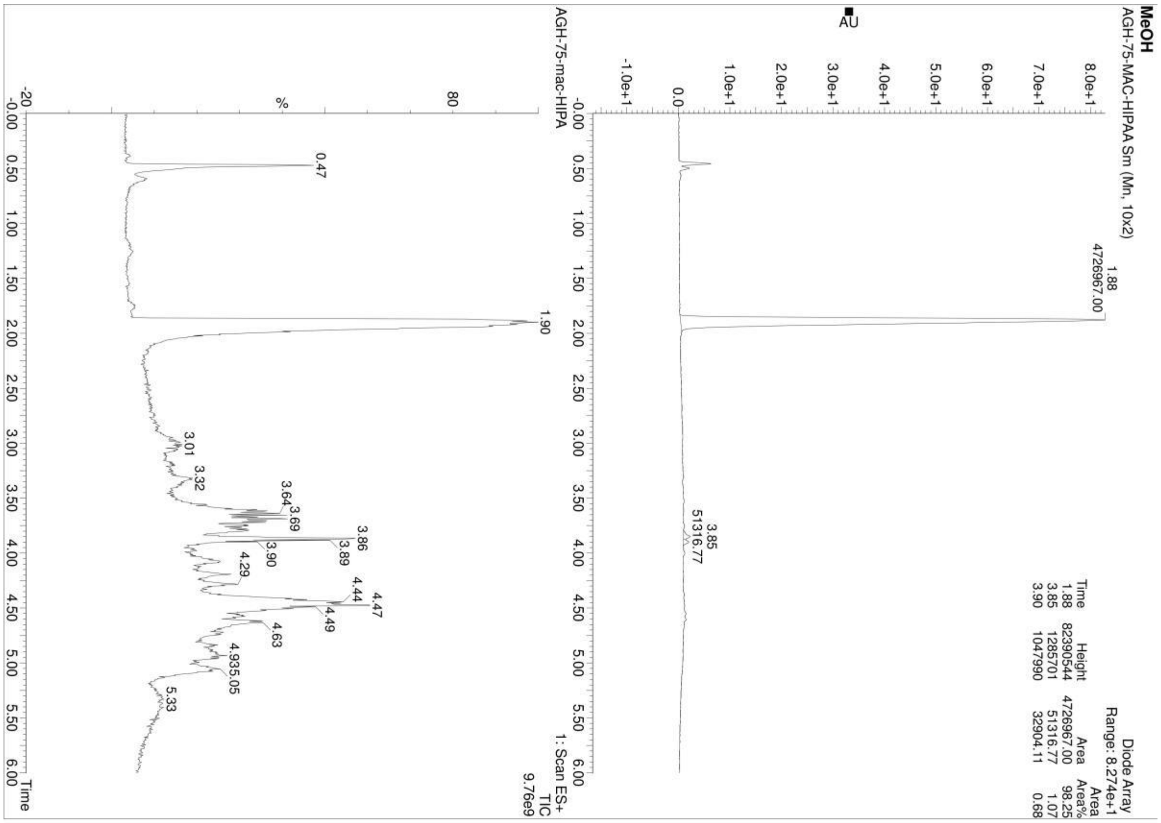
MeOH
AGH-75-MAC-HIPAA Sm (Mn, 10x2)
AU
Diode Array
Range: 8.274e+1
Time Height Area Area%
1.88 82390544 4726967.00 98.25
3.85 1285701 51316.77 1.07
3.90 1047990 32904.11 0.68
1.88
4726967.00
3.85
5316.77
AGH-75-mac-HIPA
1: Scan ES+
TIC
9.76e9
0.47
1.90
3.01
3.32
3.64
3.69
3.86
3.89
3.90
4.29
4.44
4.47
4.49
4.63
4.93
5.05
5.33
Time

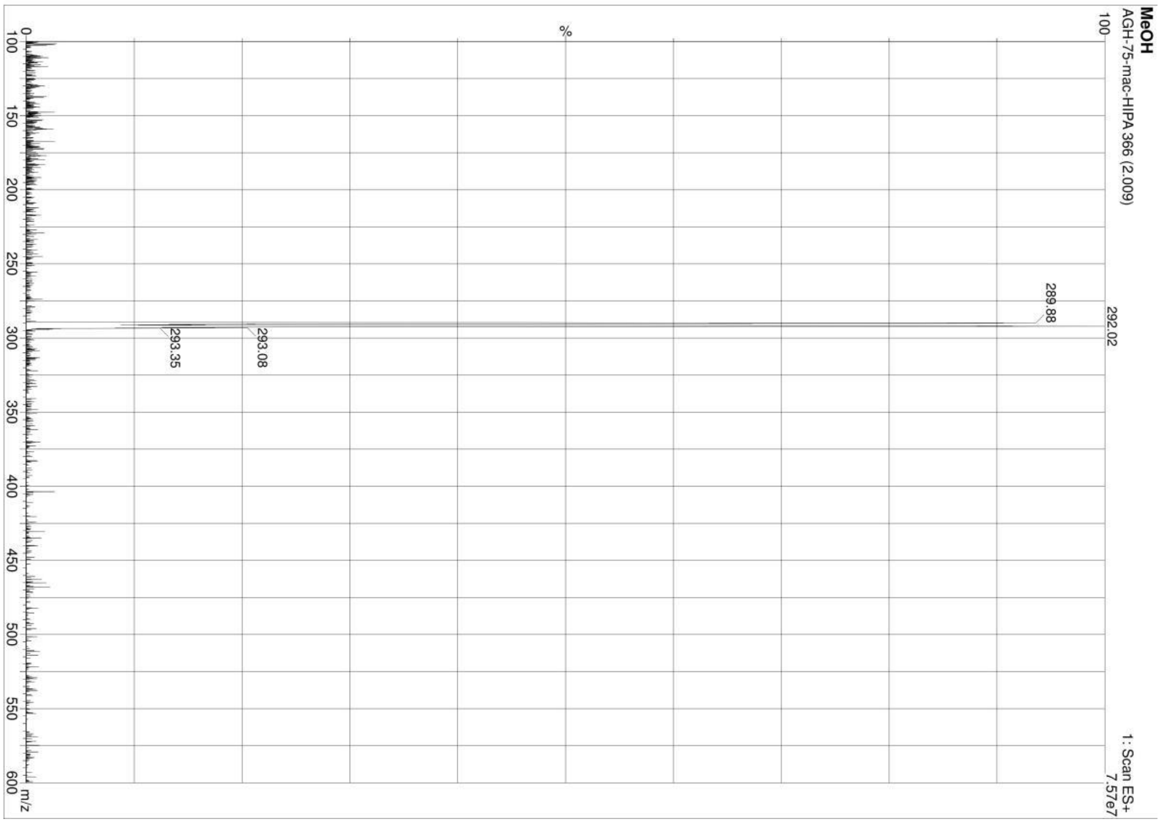
MeOH
AGH-75-mac-HIPA 366 (2.009)
1: Scan ES+
7.57e7
289.88
292.02
293.08
293.35
m/z

1o: 5-iodo-3-(1-ethyl-1*H*-imidazol-5-yl)-1*H*-indole (AGH-107)

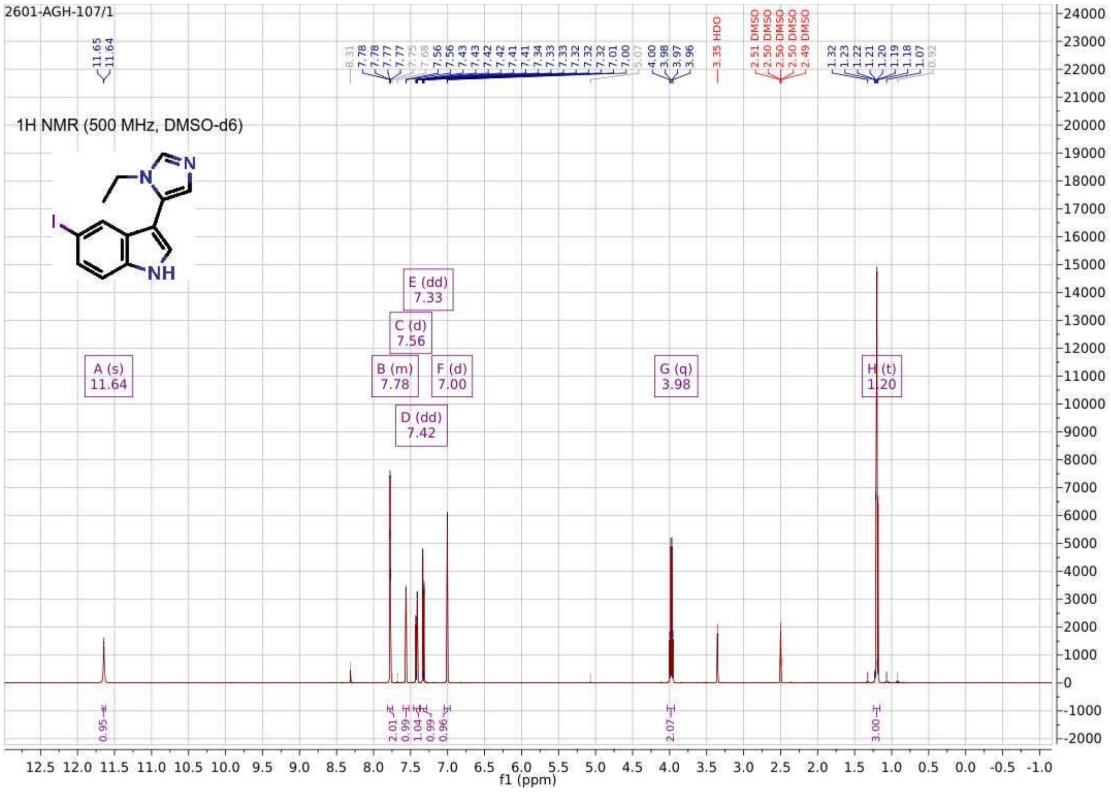

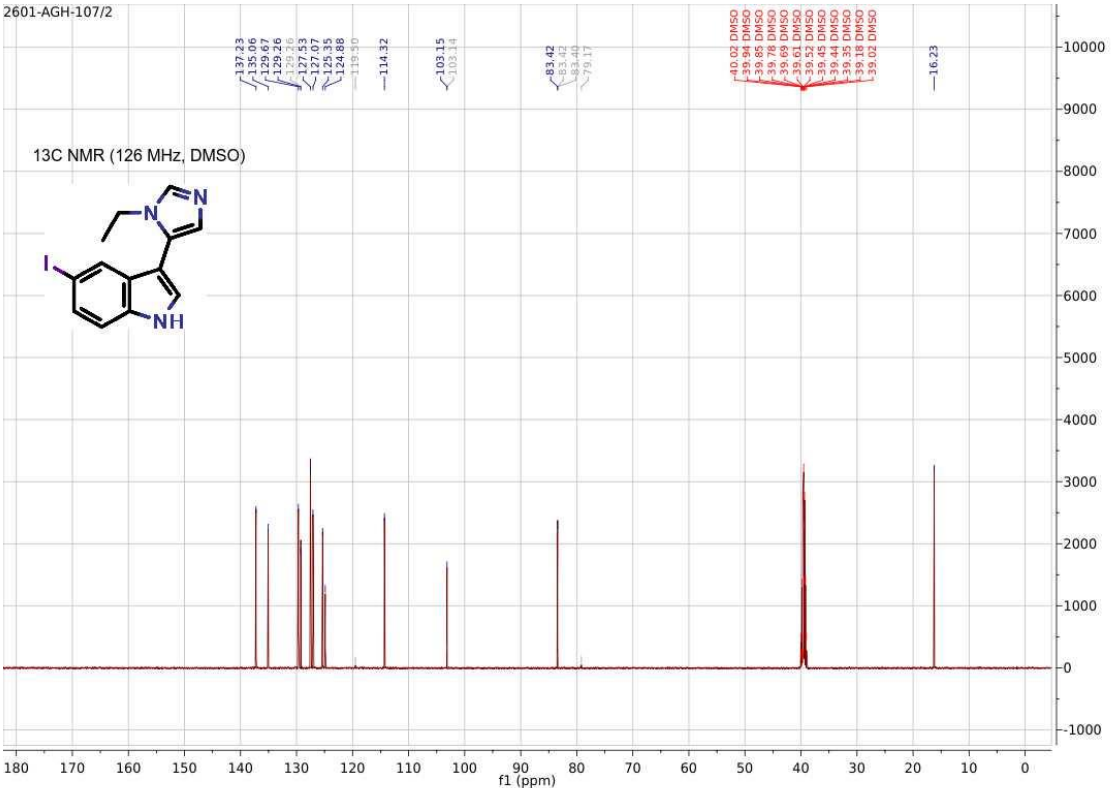

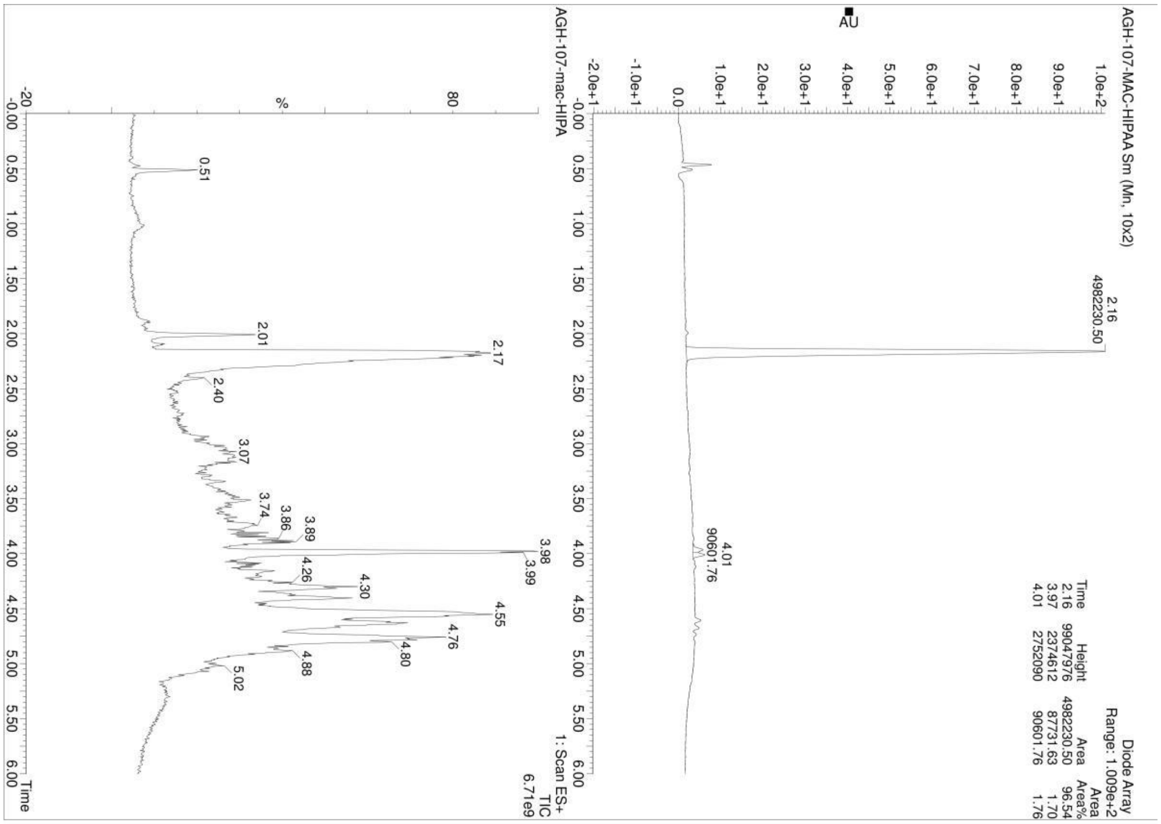
AGH-107-MAC-HIPAA Sm (Mn, 10x2)
Diode Array
Range: 1.009e+2
Time Height Area Area%
2.16 99047976 4982230.50 96.54
3.97 2374612 87731.63 1.70
4.01 2752090 90601.76 1.76
AU
AGH-107-mac-HIPA
1: Scan ES+
TIC
6.71e9
Time

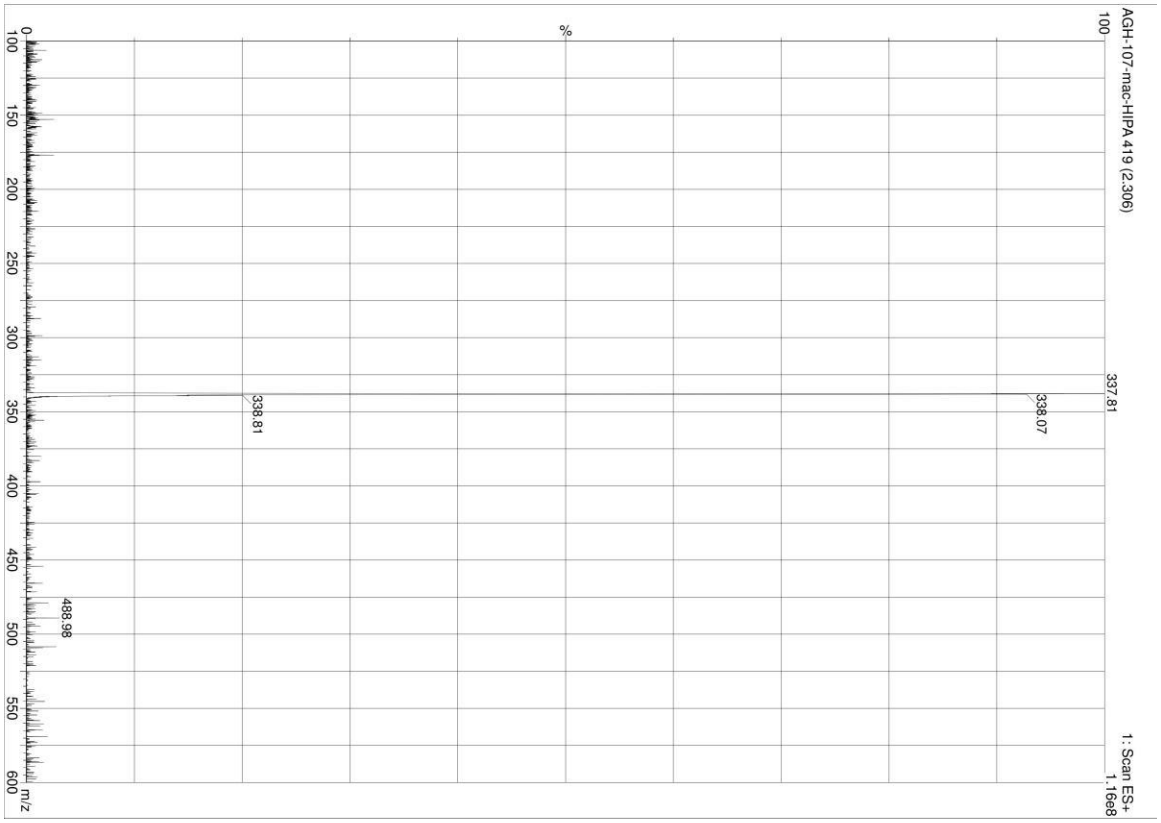
AGH-107-mac-HIPA 419 (2.306)
1: Scan ES+
1.16e8
337.81
338.07
338.81
488.98
m/z

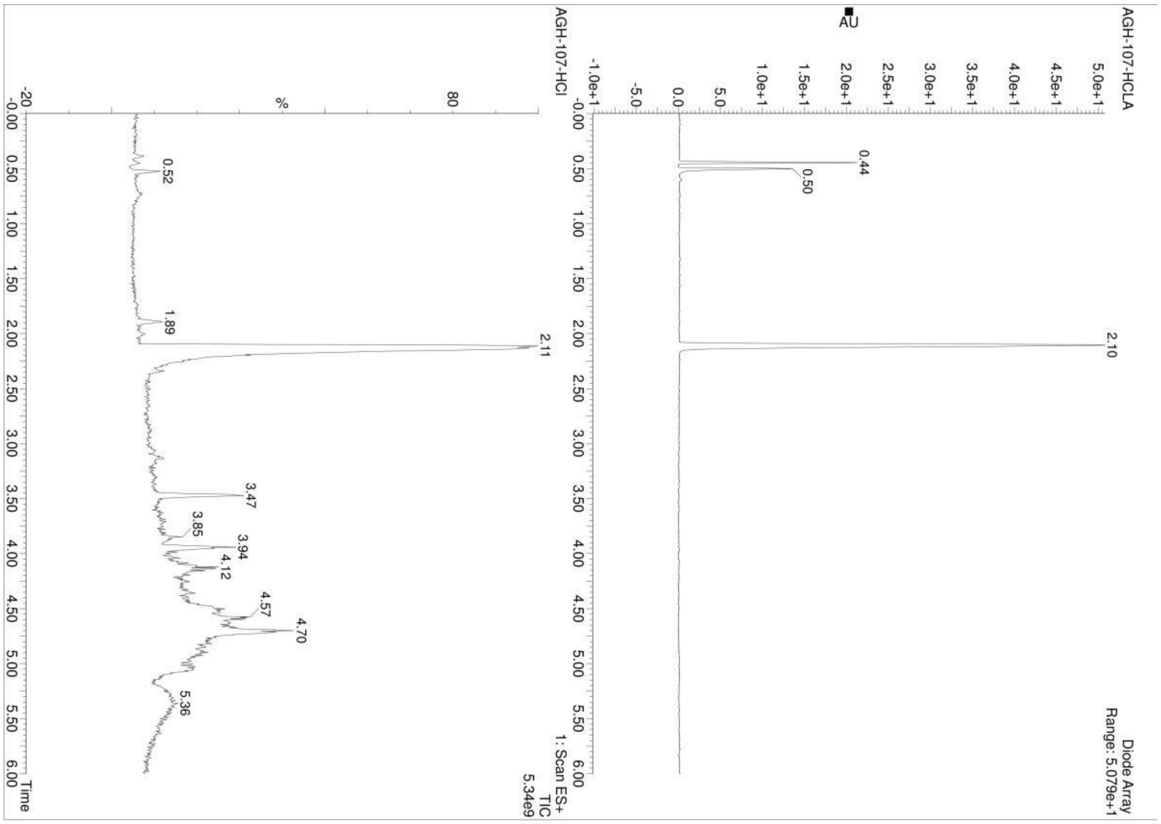

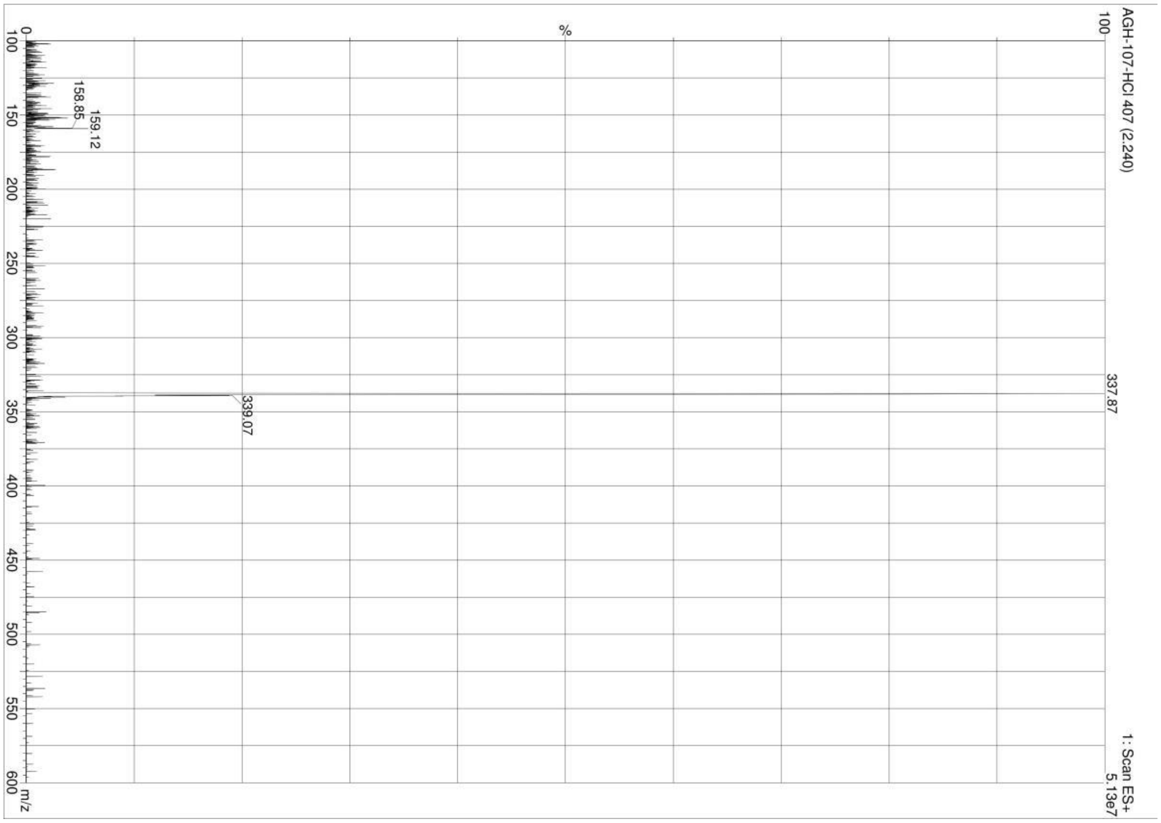

1p: 3-(1-ethyl-1*H*-imidazol-5-yl)-1*H*-indole-5-carbonitrile (AGH-85)

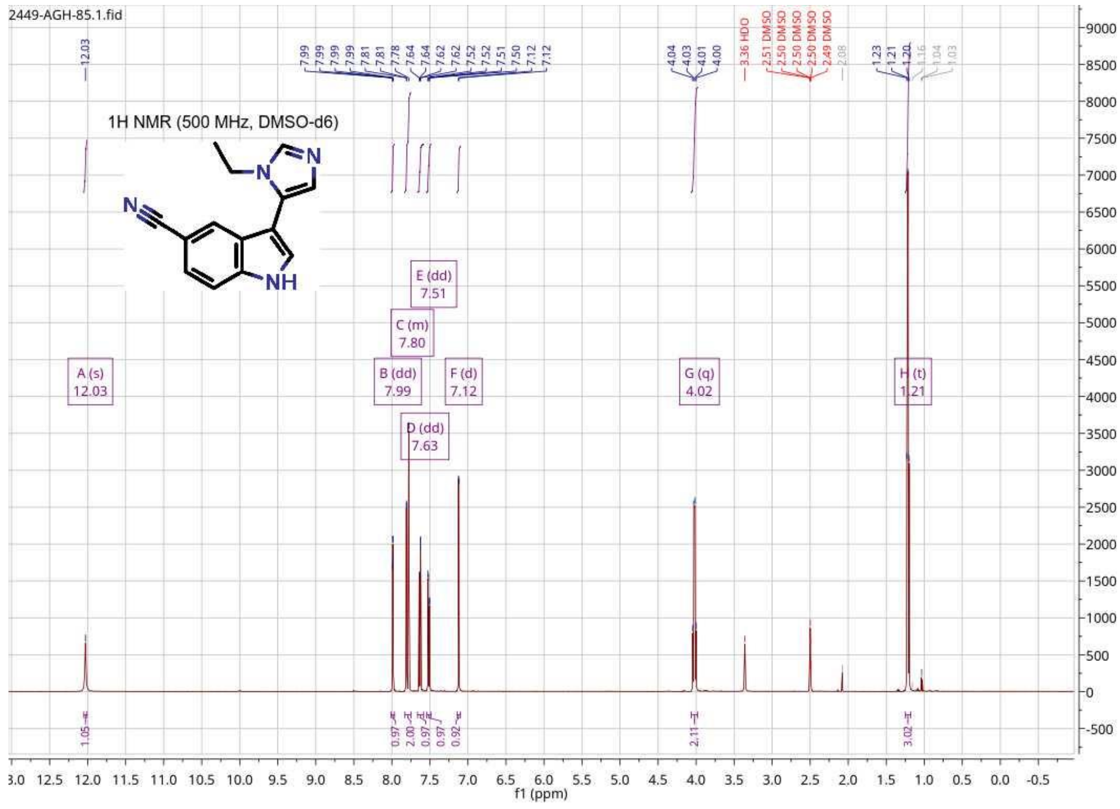
2449-AGH-85.1.fid
1H NMR (500 MHz, DMSO-d6)
A (s) 12.03
B (dd) 7.99
C (m) 7.80
D (dd) 7.63
E (dd) 7.51
F (d) 7.12
G (q) 4.02
H (t) 1.21
f1 (ppm)

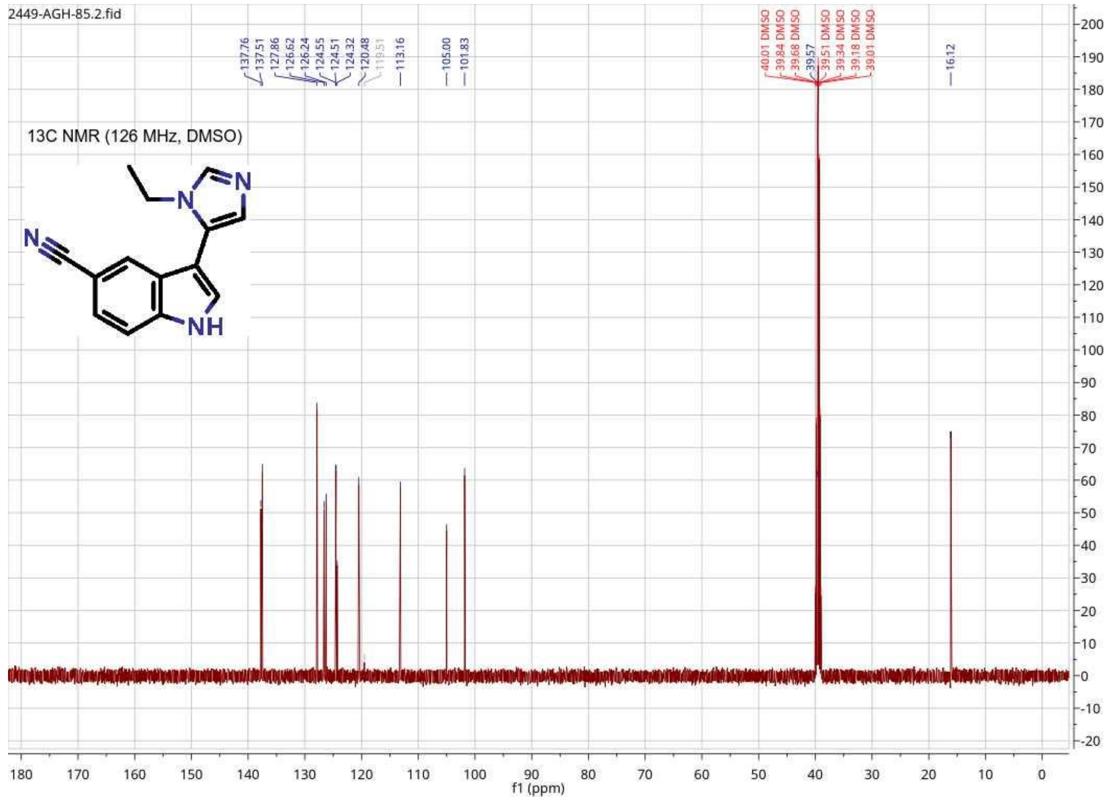
2449-AGH-85.2.fid
13C NMR (126 MHz, DMSO)
137.76
137.51
127.86
126.62
126.24
124.55
124.51
124.32
120.48
113.16
105.00
101.83
16.12
f1 (ppm)

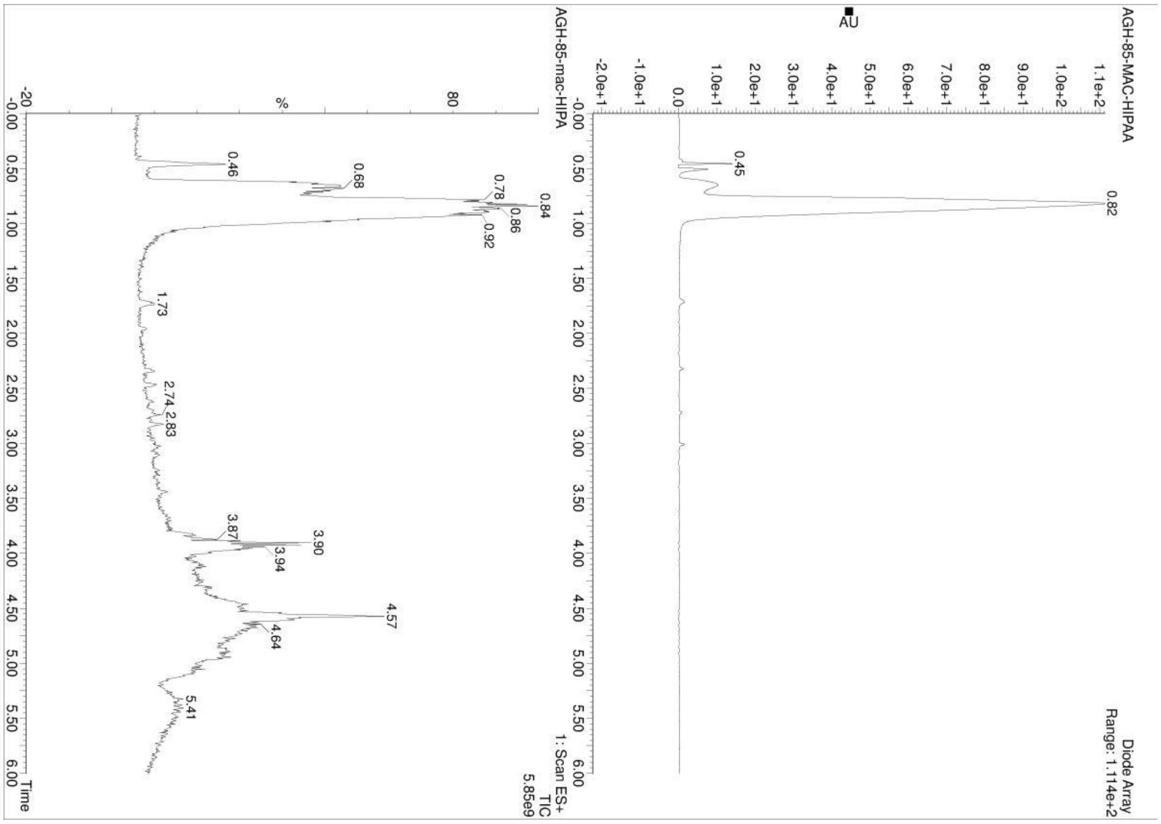

AGH-85-MAC-HIPAA
Diode Array
Range: 1.114e+2
AU
AGH-85-mac-HIPA
1: Scan ES+
TIC
5.85e9
Time

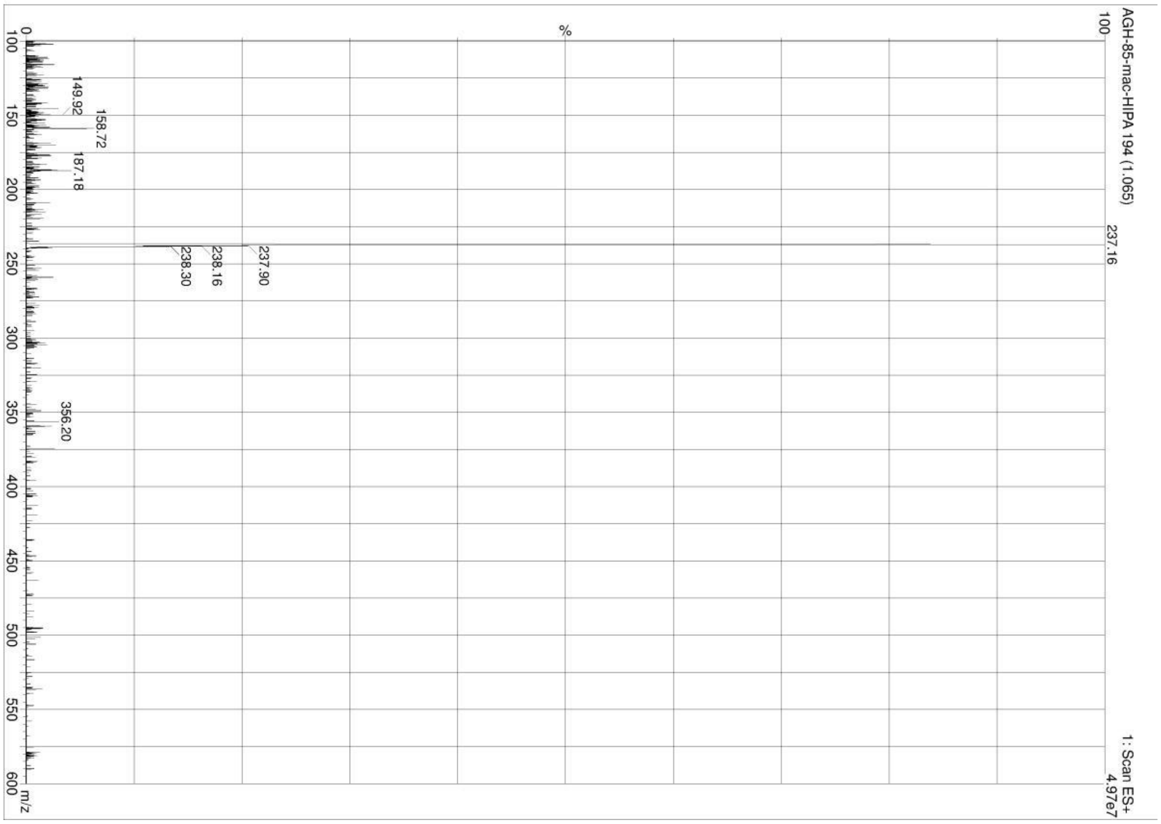

AGH-85-mac-HIPA 194 (1.065)
1: Scan ES+
4.97e7
237.16
m/z

1q: 3-(1-ethyl-1*H*-imidazol-5-yl)-5-methyl-1*H*-indole (AGH-108)

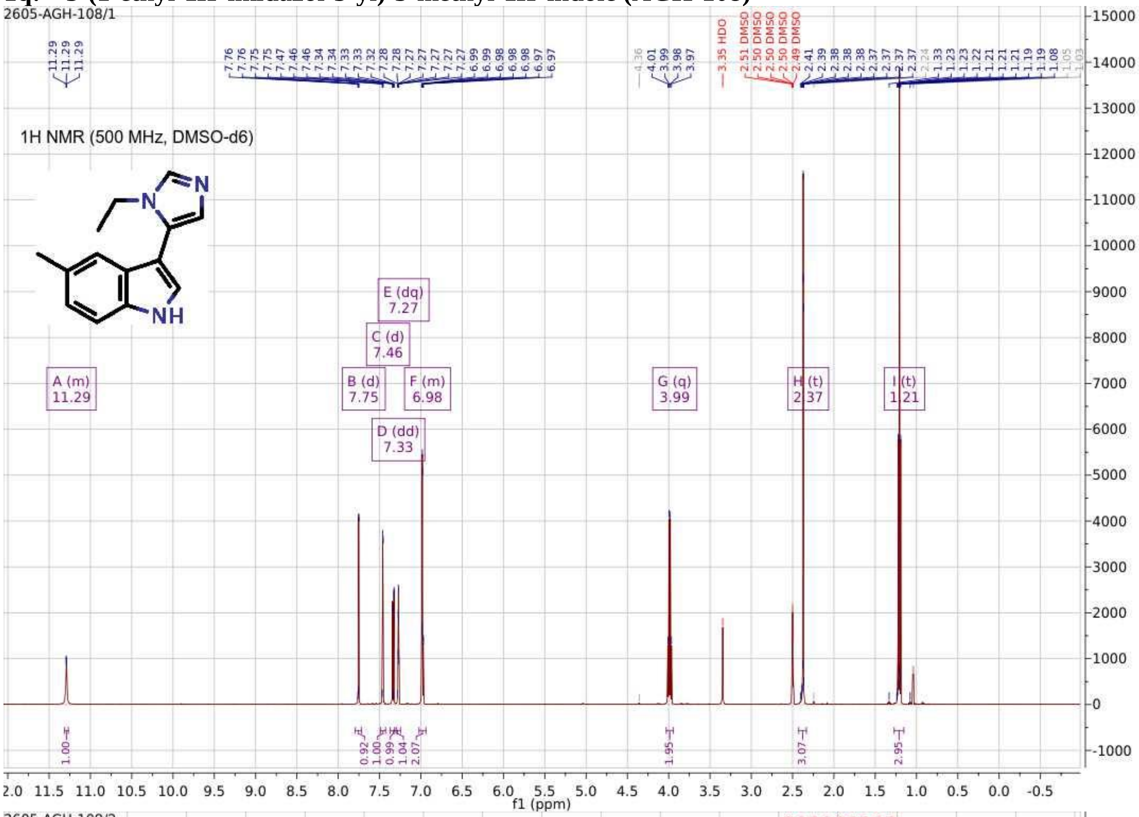

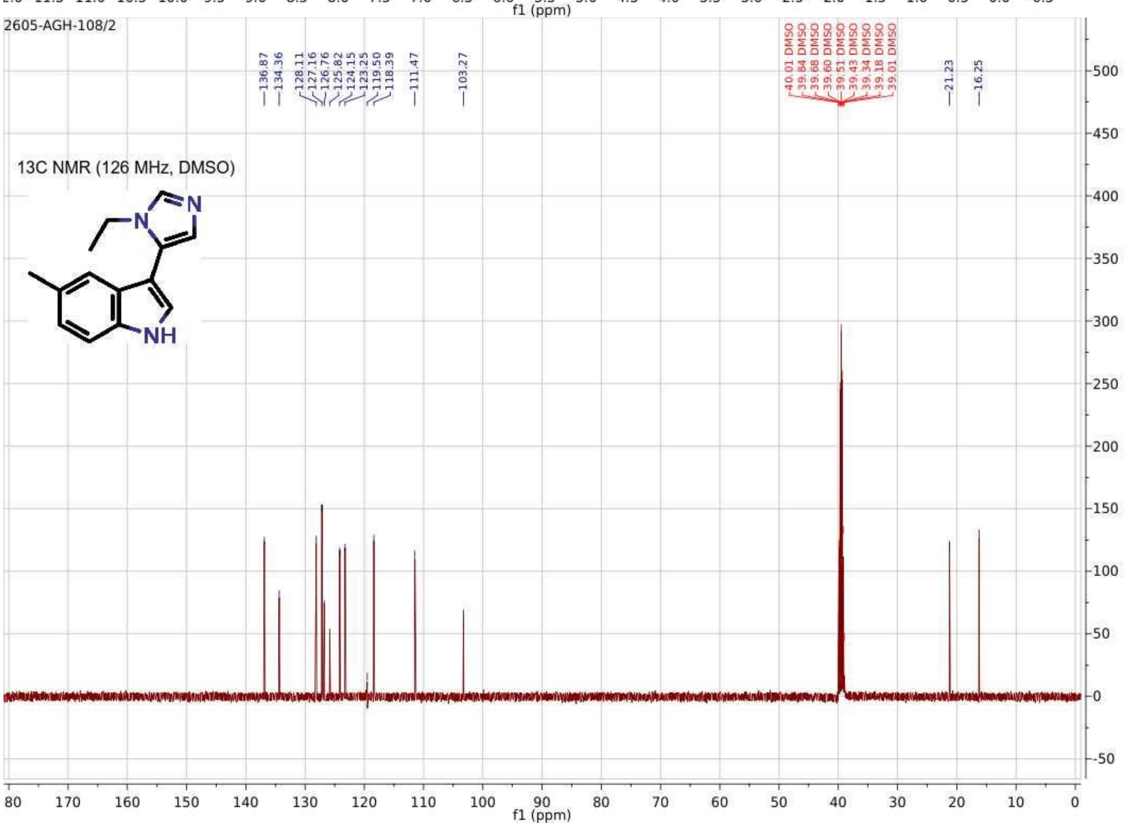

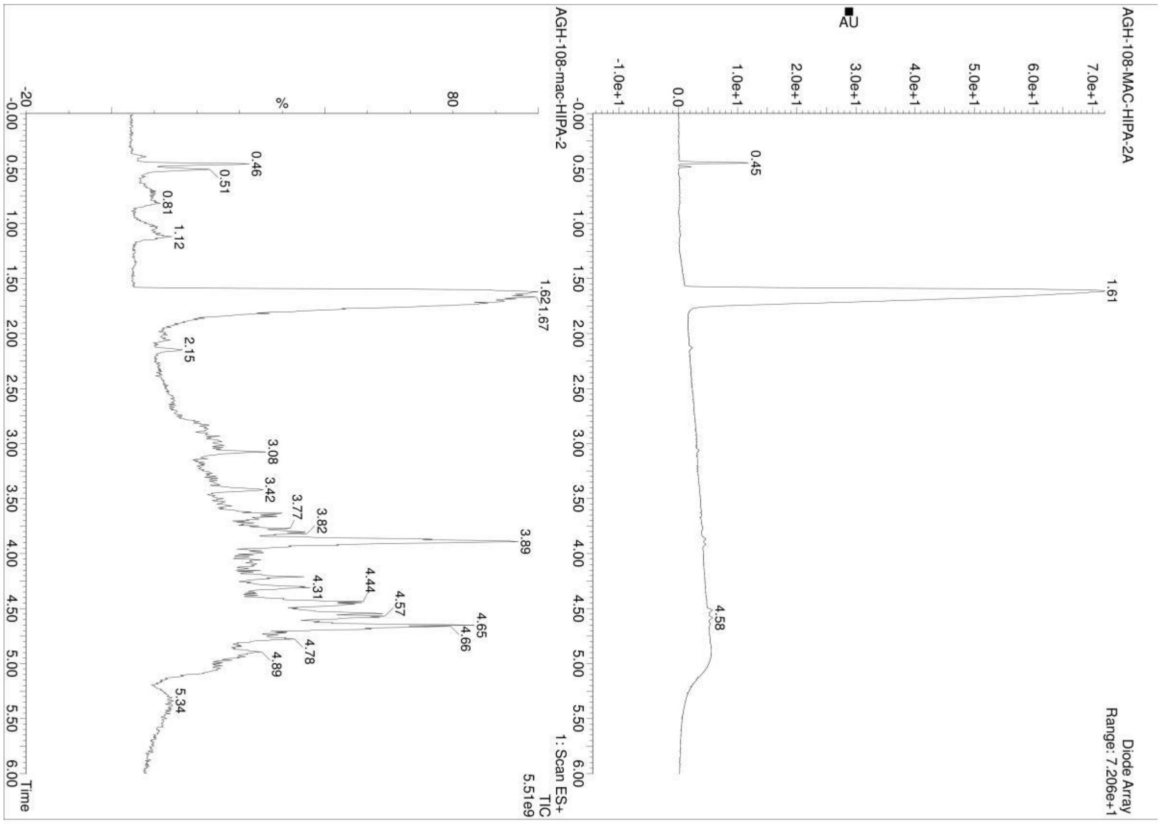
AGH-108-MAC-HIPA-2A
Diode Array
Range: 7.206e+1
AU
AGH-108-mac-HIPA-2
1: Scan ES+
TIC
5.51e9
Time

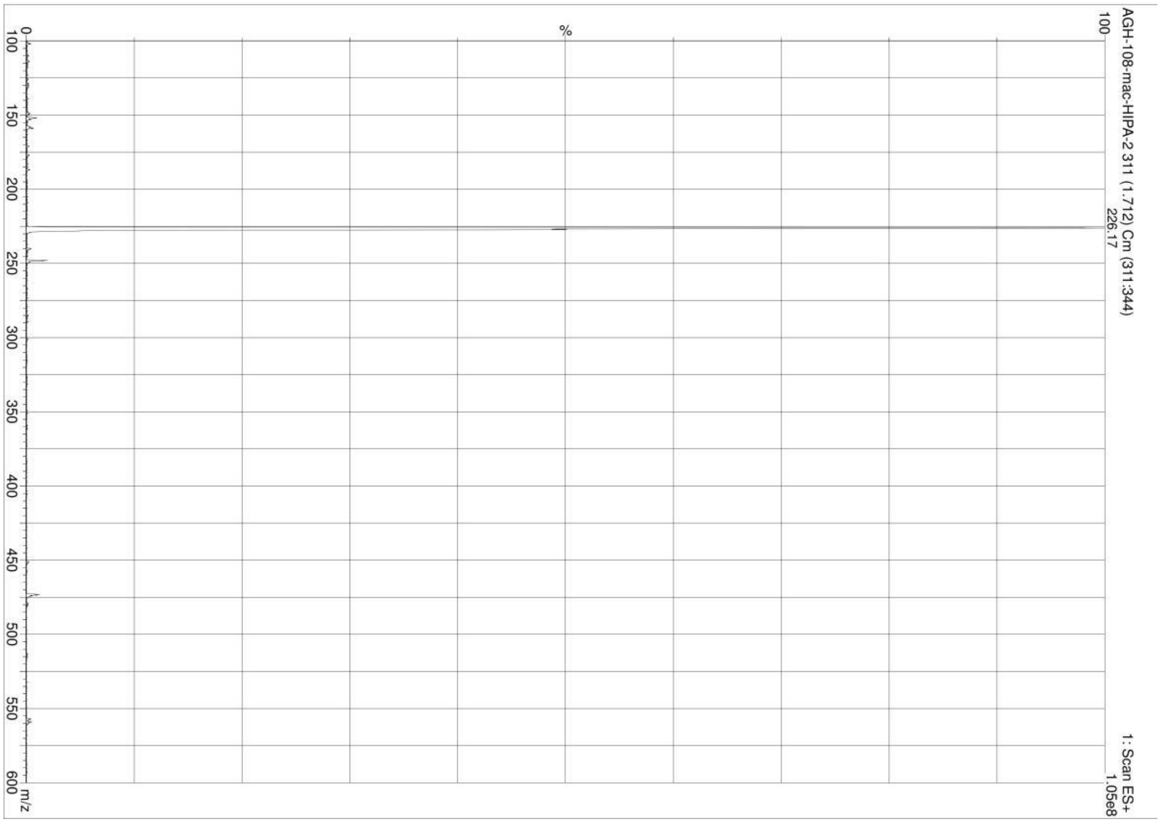
AGH-108-mac-HIPA-2 311 (1.712) Cm (311:344)
226.17
1: Scan ES+
1.05e8
m/z

1r: 3-(1-ethyl-1*H*-imidazol-5-yl)-1*H*-indol-5-ol (AGH-110)

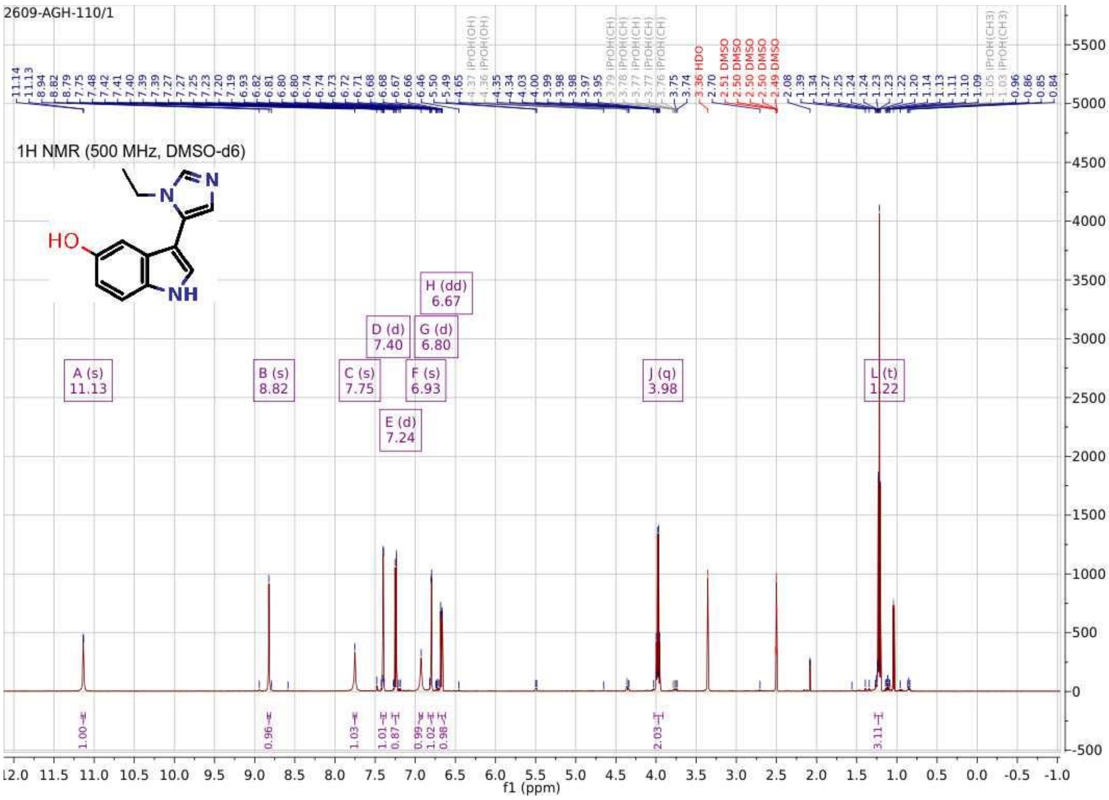

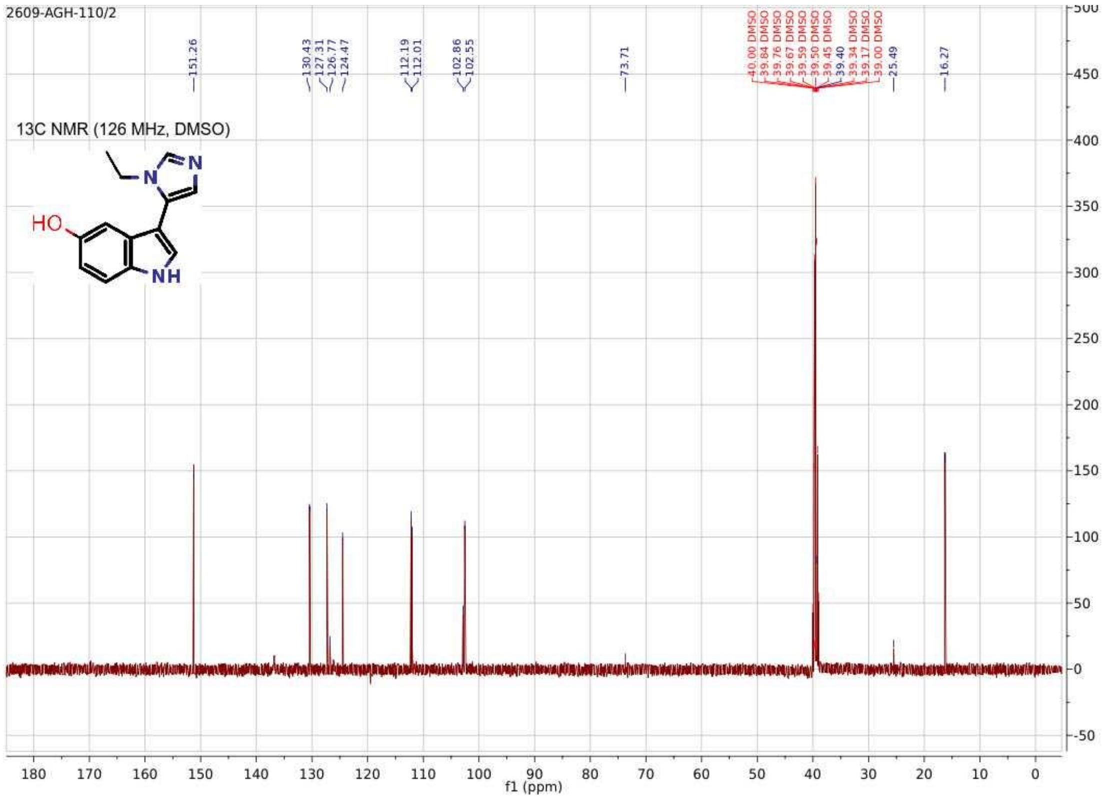

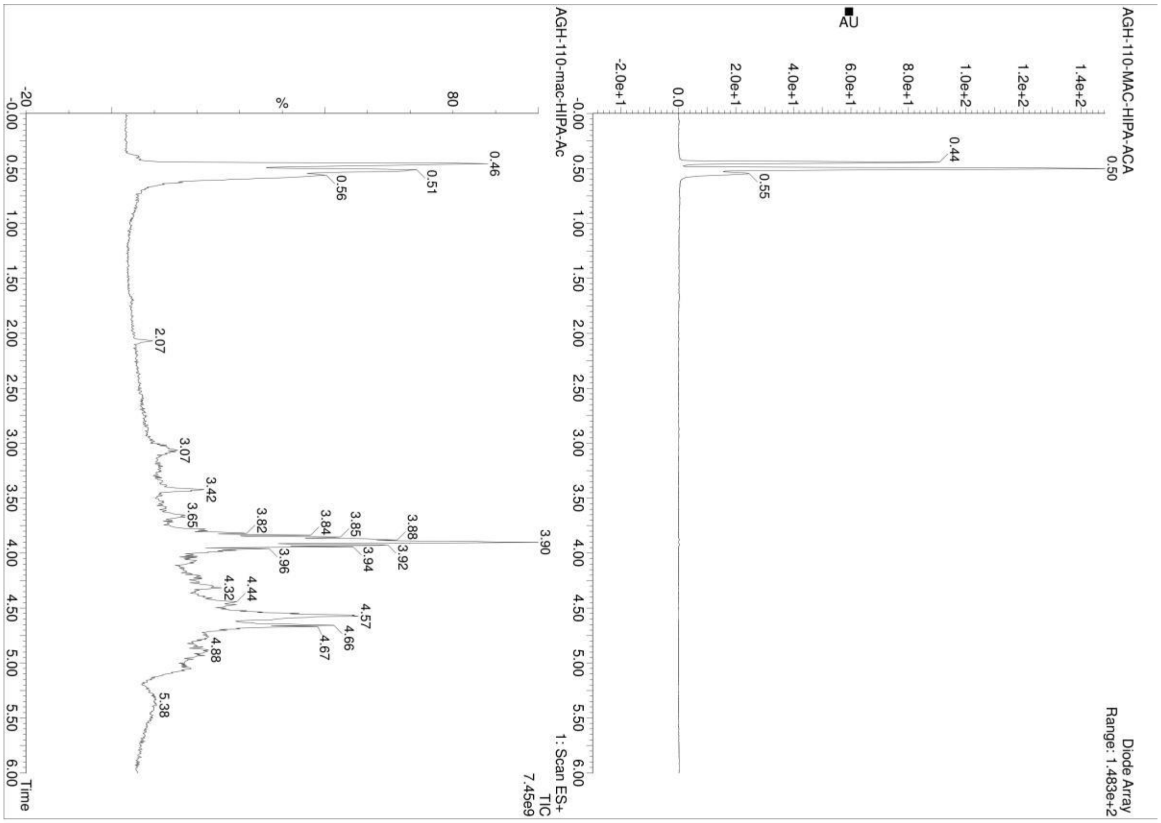
AGH-110-MAC-HIPA-ACA
Diode Array
Range: 1.483e+2
AU
AGH-110-mac-HIPA-Ac
1: Scan ES+
TIC
7.45e9
Time

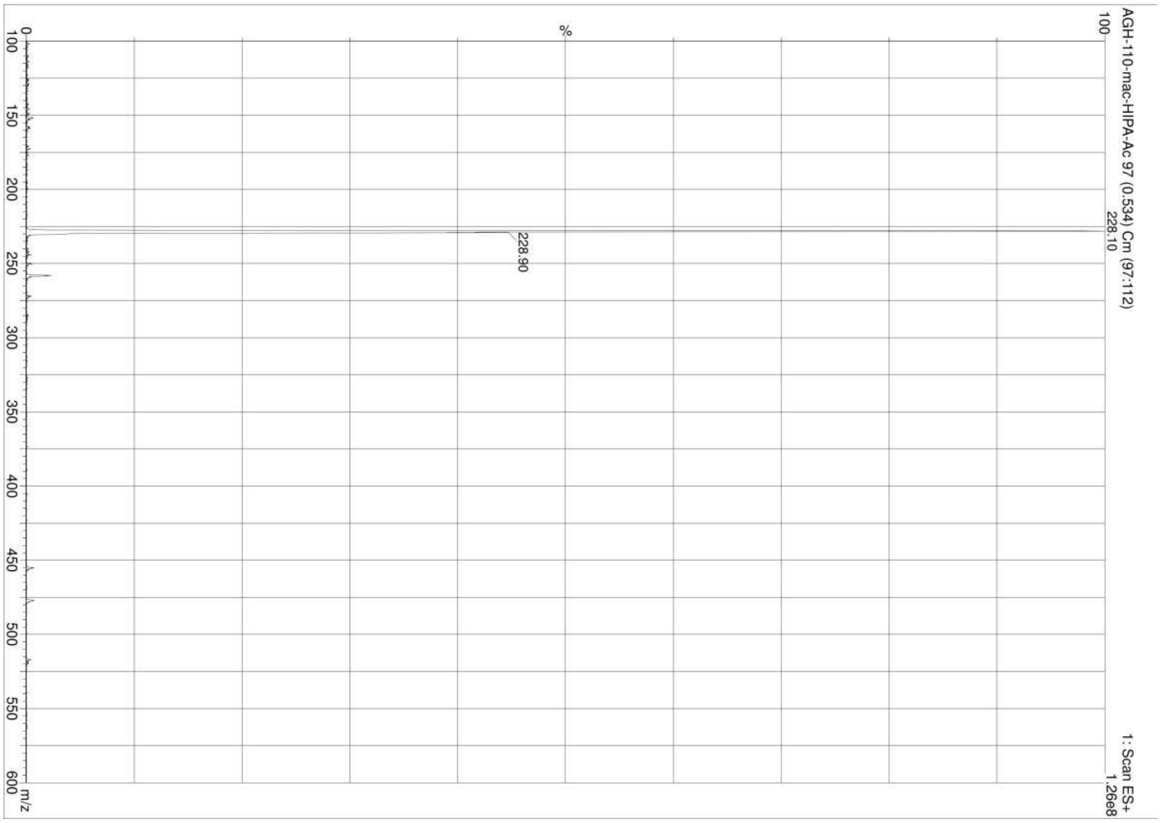
AGH-110-mac-HIPA-Ac 97 (0.534) Cm (97:112)
228.10
228.90
1: Scan ES+
1.26e8
m/z

1s: 3-(1-benzyl-1*H*-imidazol-5-yl)-5-methoxy-1*H*-indole (AGH-58)

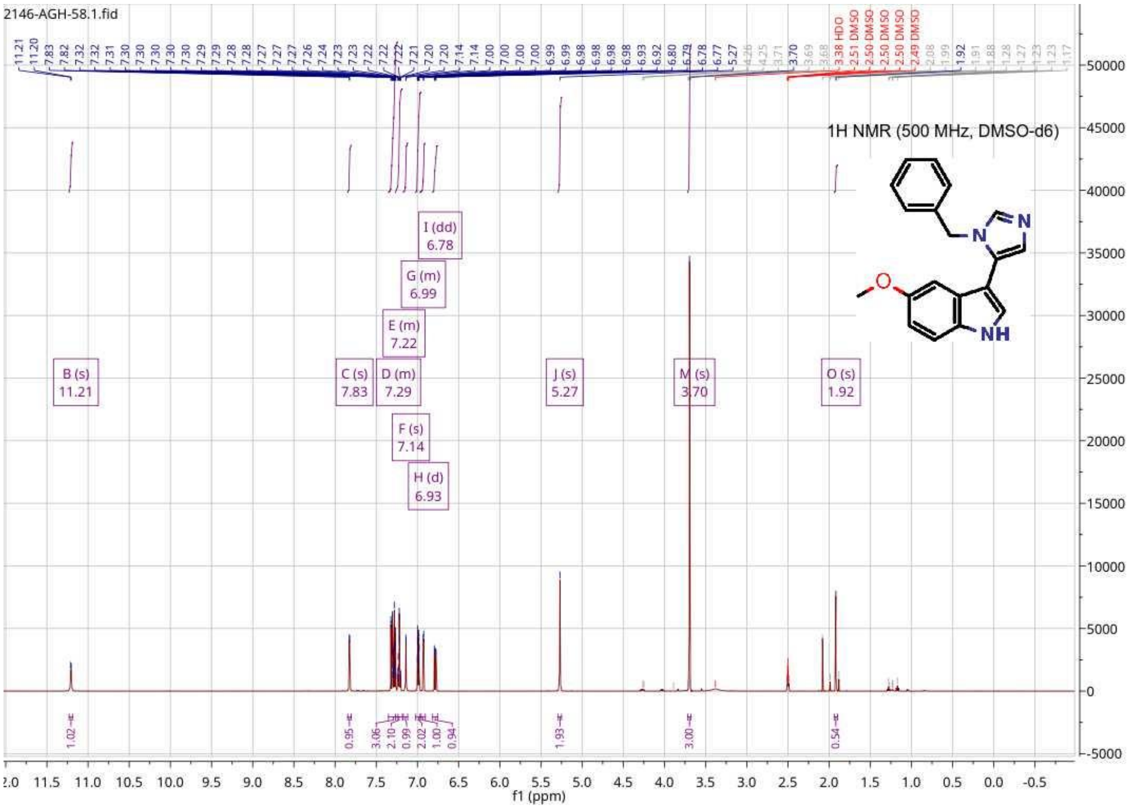

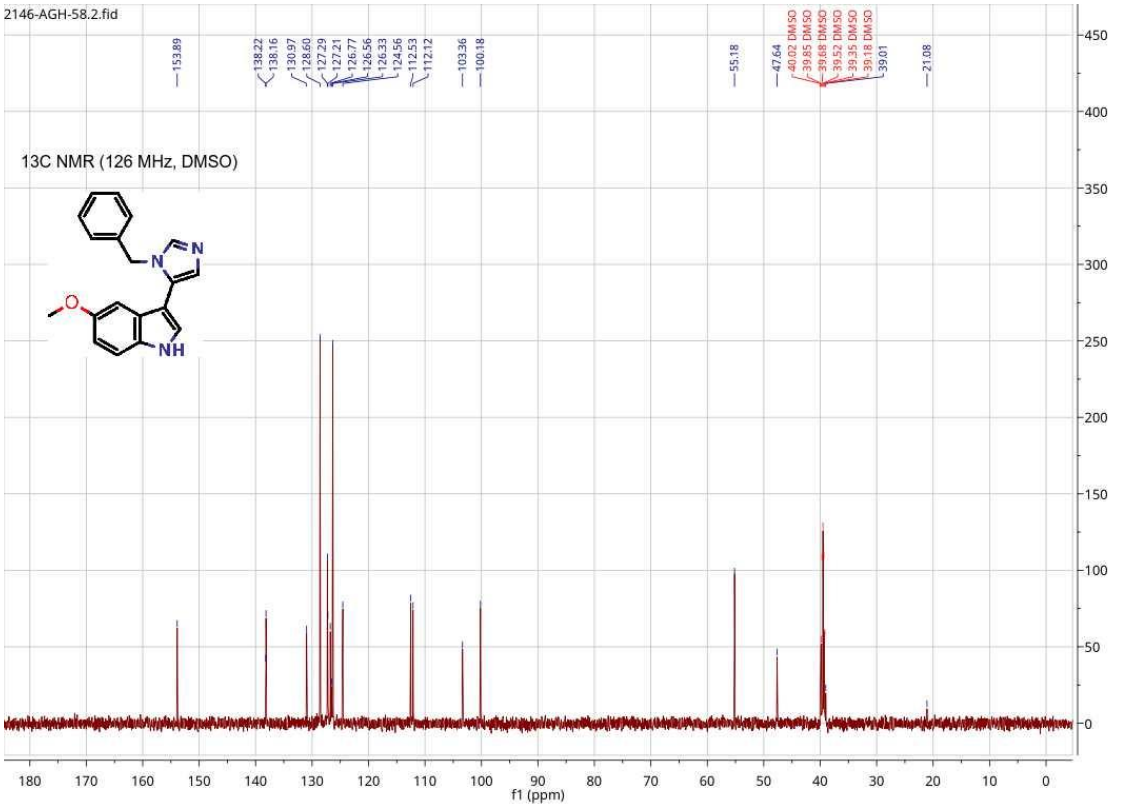

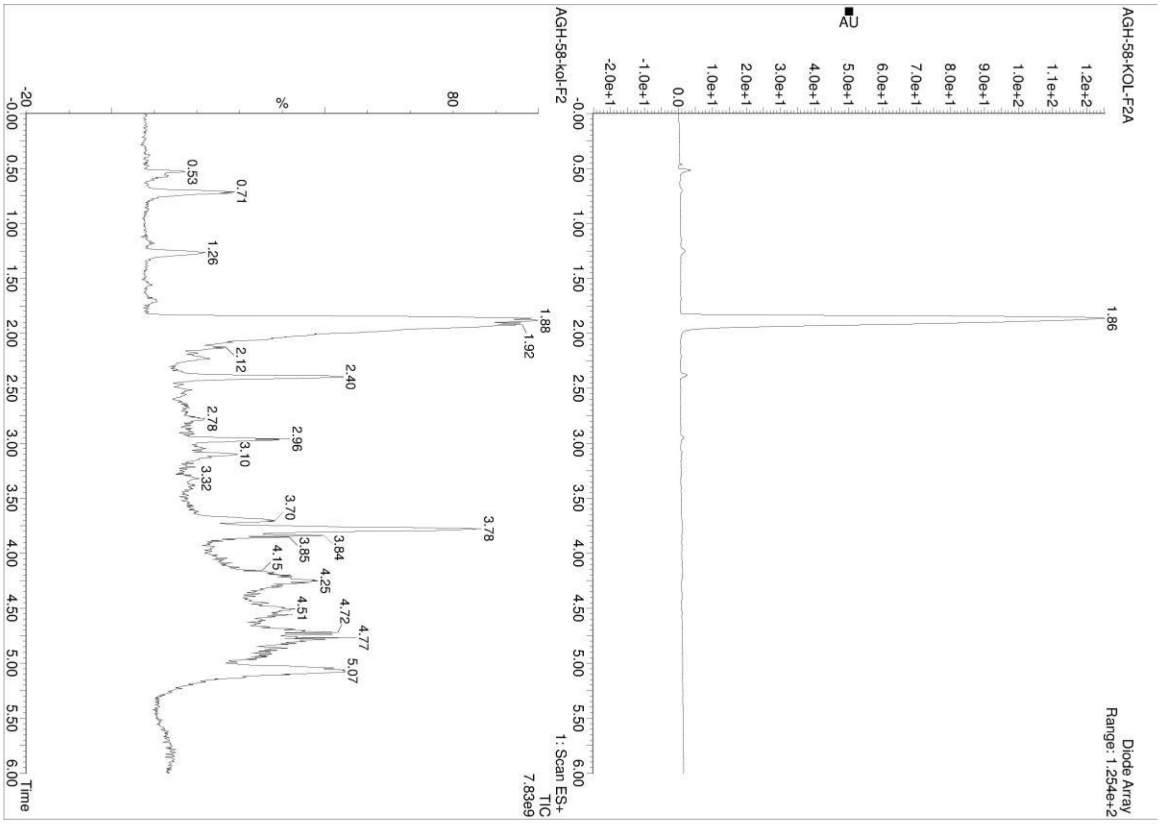
AGH-58-KOL-F2A
Diode Array
Range: 1.254e+2
AU
1.86
AGH-58-kol-F2
1: Scan ES+
TIC
7.83e9
1.88
1.92
3.78
Time

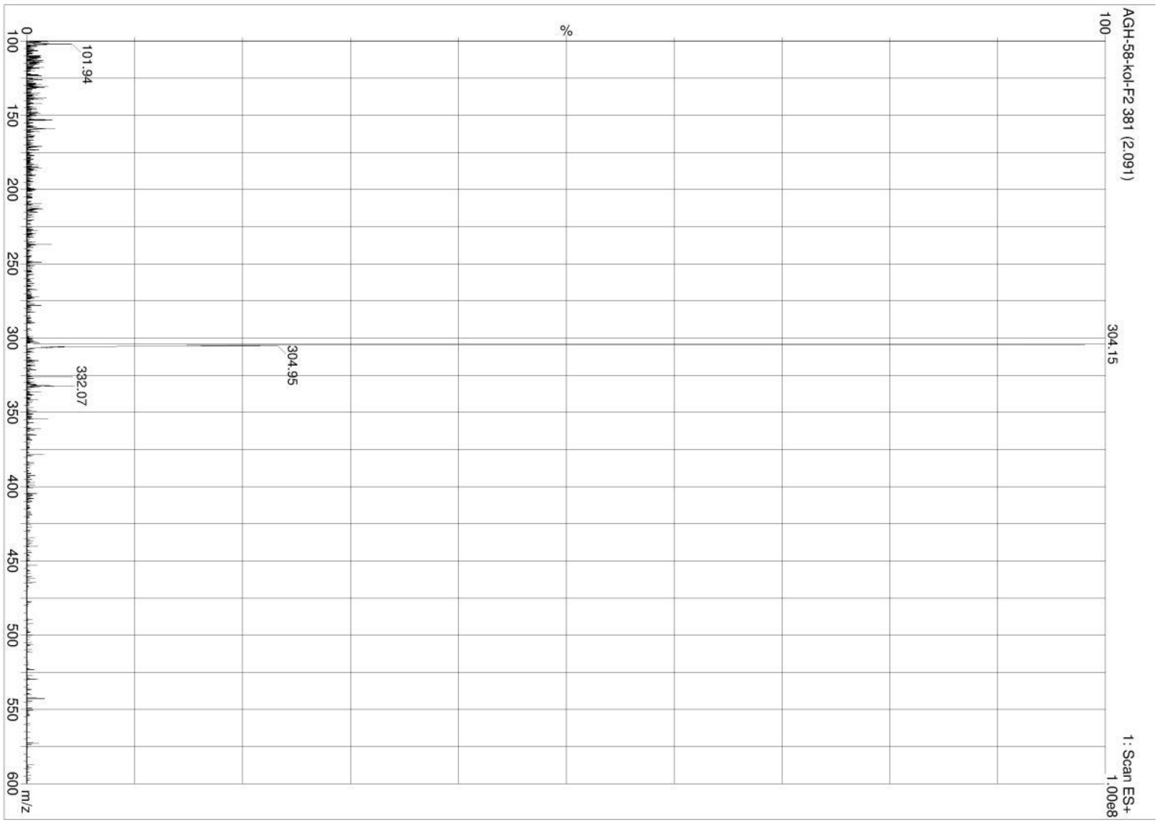
AGH-58-kol-F2 381 (2.091)
1: Scan ES+
1.00e8
304.15
304.95
332.07
101.94
m/z

1t: 3-(1-ethyl-1*H*-imidazol-5-yl)-4-methoxy-1*H*-indole (AGH-102)

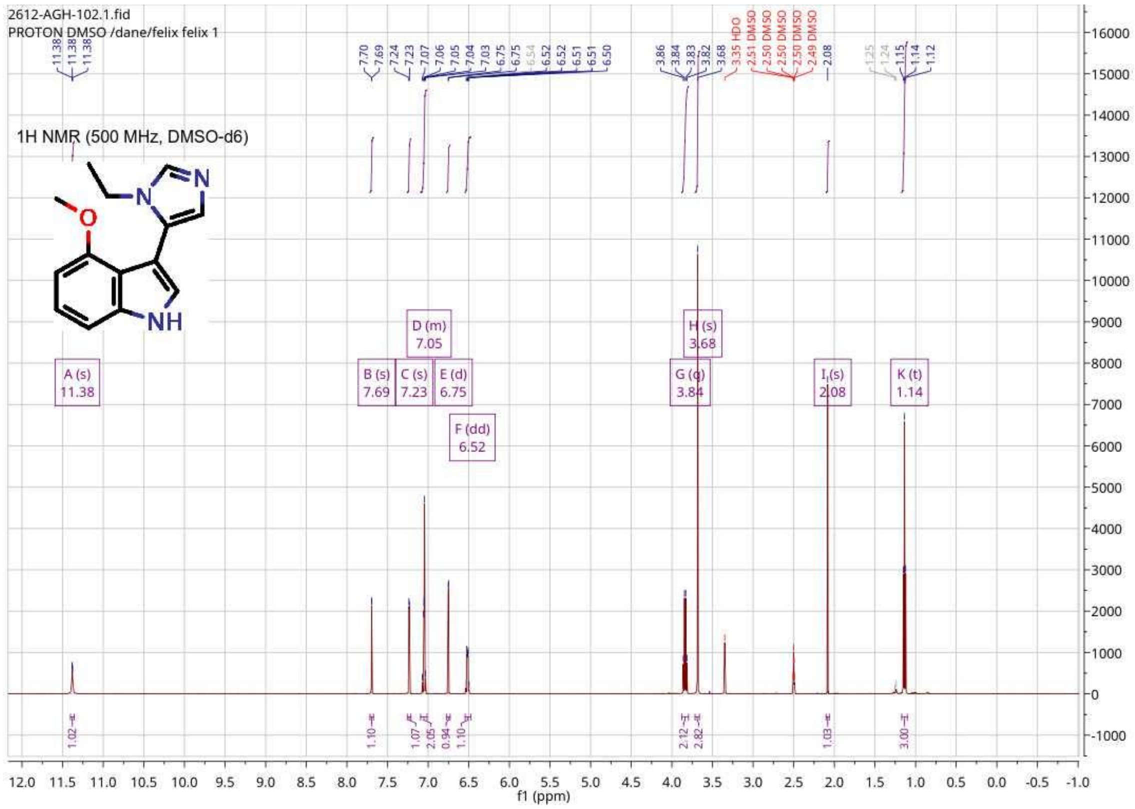

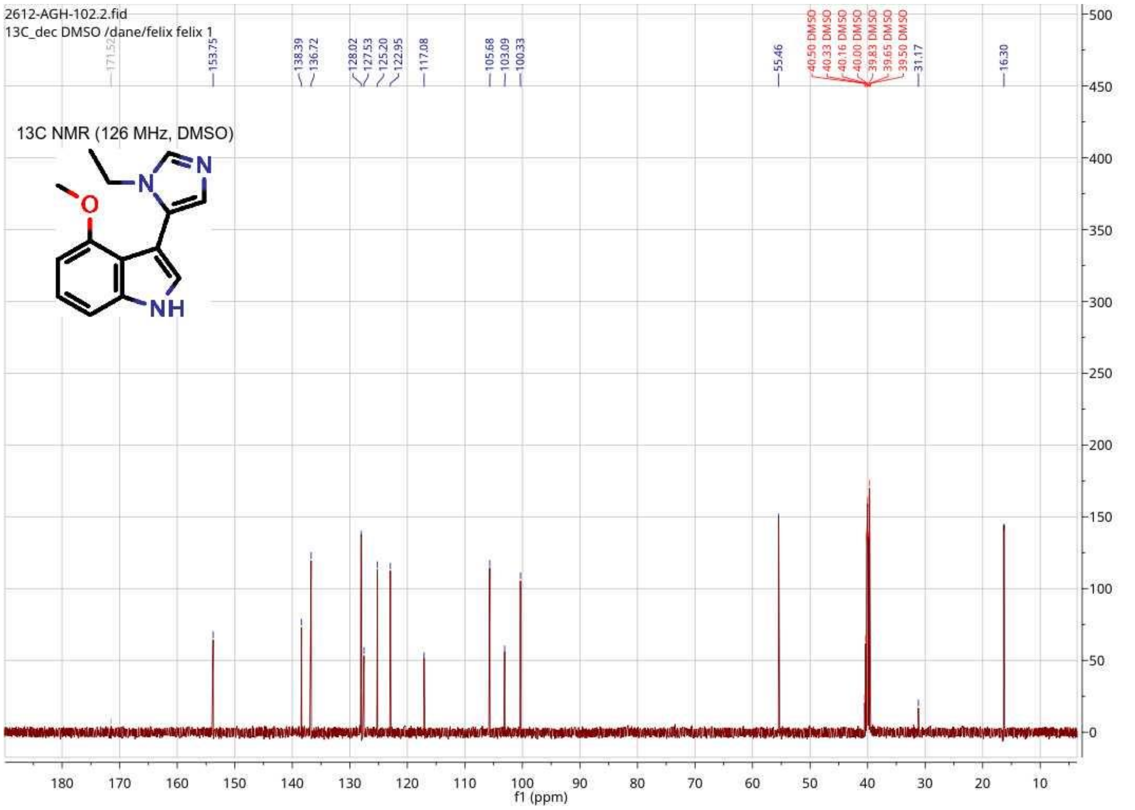

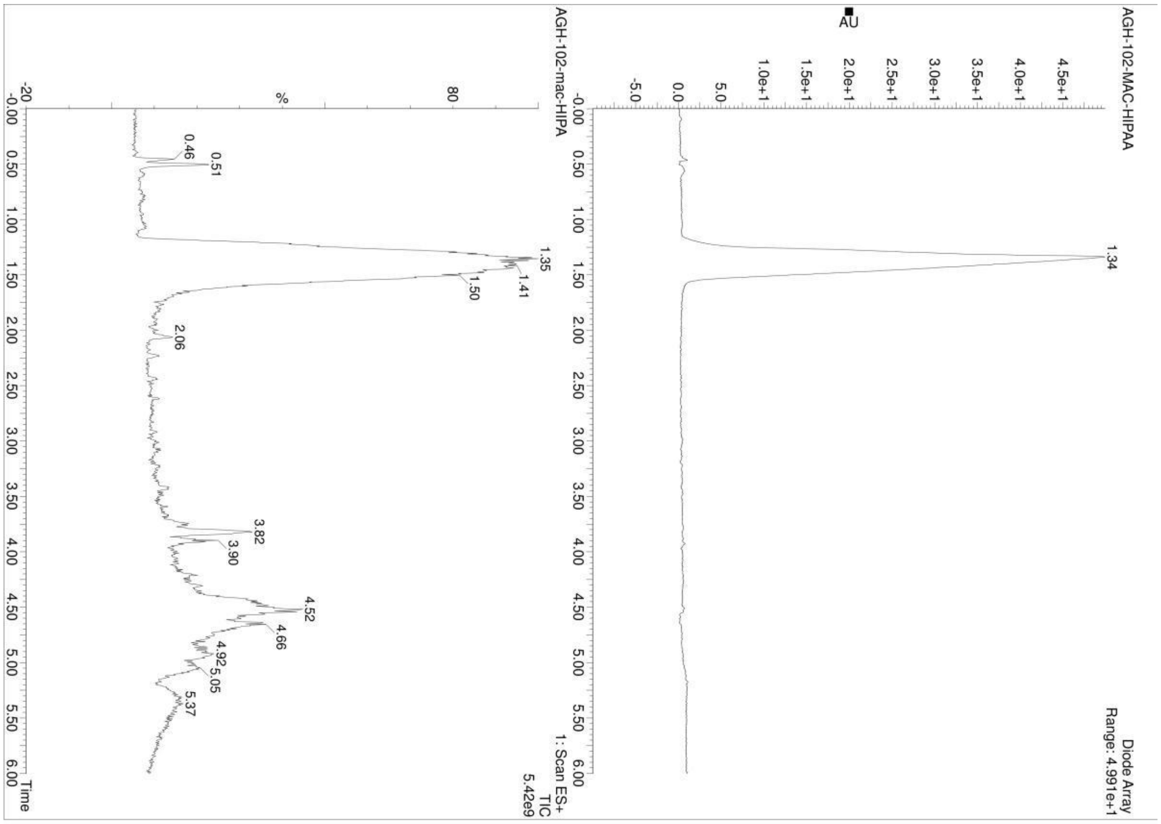
AGH-102-MAC-HIPAA
Diode Array
Range: 4.991e+1
AU
1.34
AGH-102-mac-HIPA
1: Scan ES+
TIC
5.42e9
%
Time
1.35
1.41
1.50
0.46
0.51
2.06
3.82
3.90
4.52
4.66
4.92
5.05
5.37

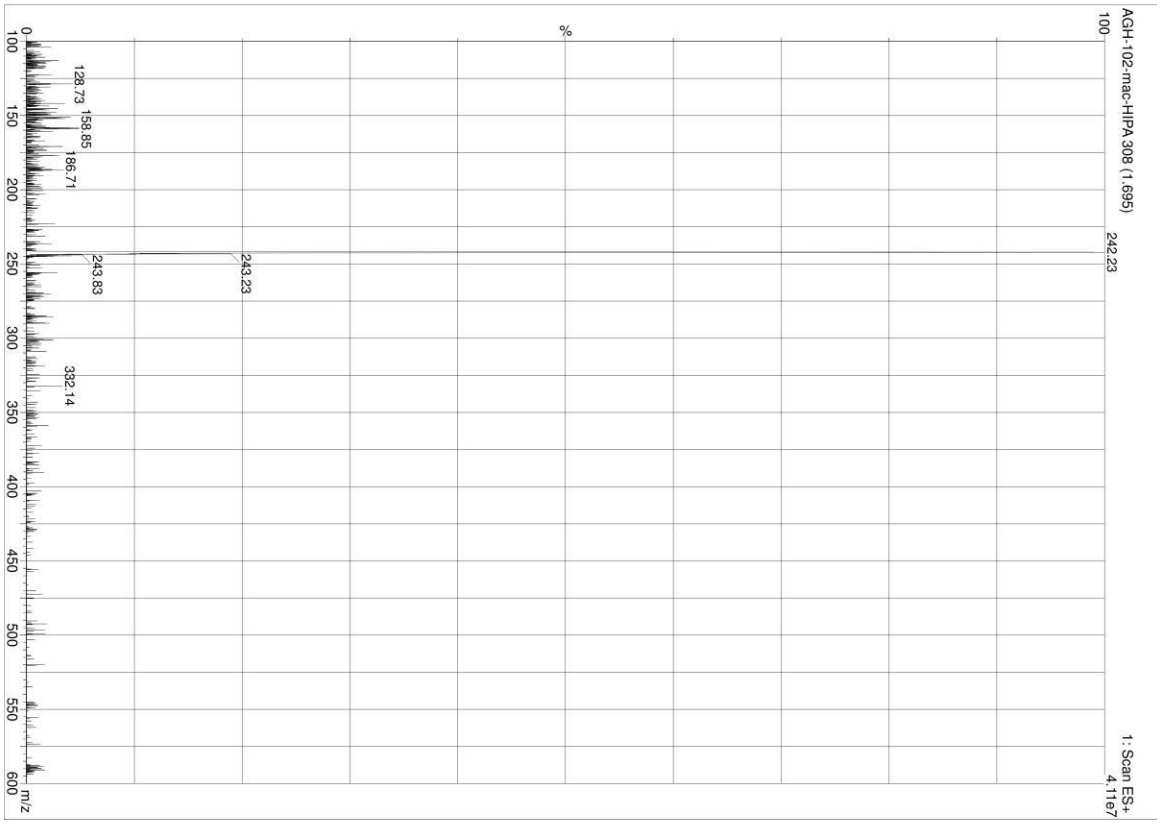
AGH-102-mac-HIPA 308 (1.695)
1: Scan ES+
4.11e7
%
m/z
242.23
243.23
243.83
128.73
158.85
186.71
332.14

1u: 3-(1-ethyl-1*H*-imidazol-5-yl)-7-methyl-1*H*-indole (AGH-104)

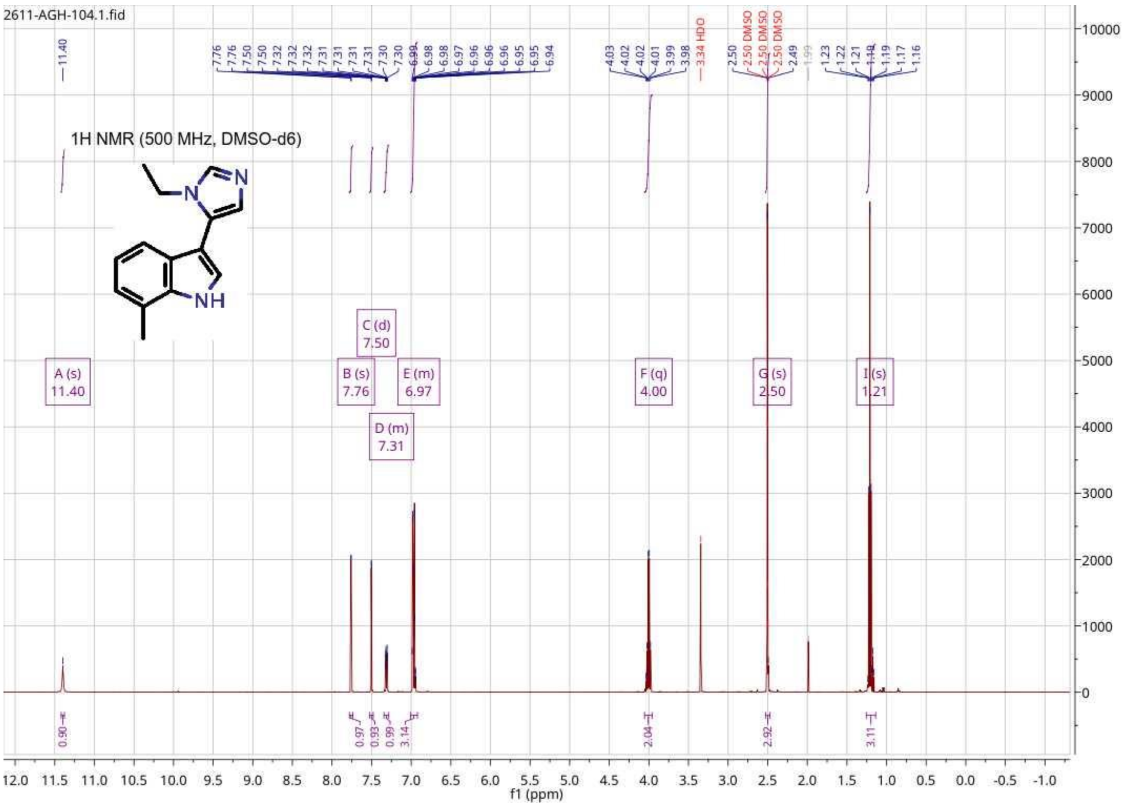

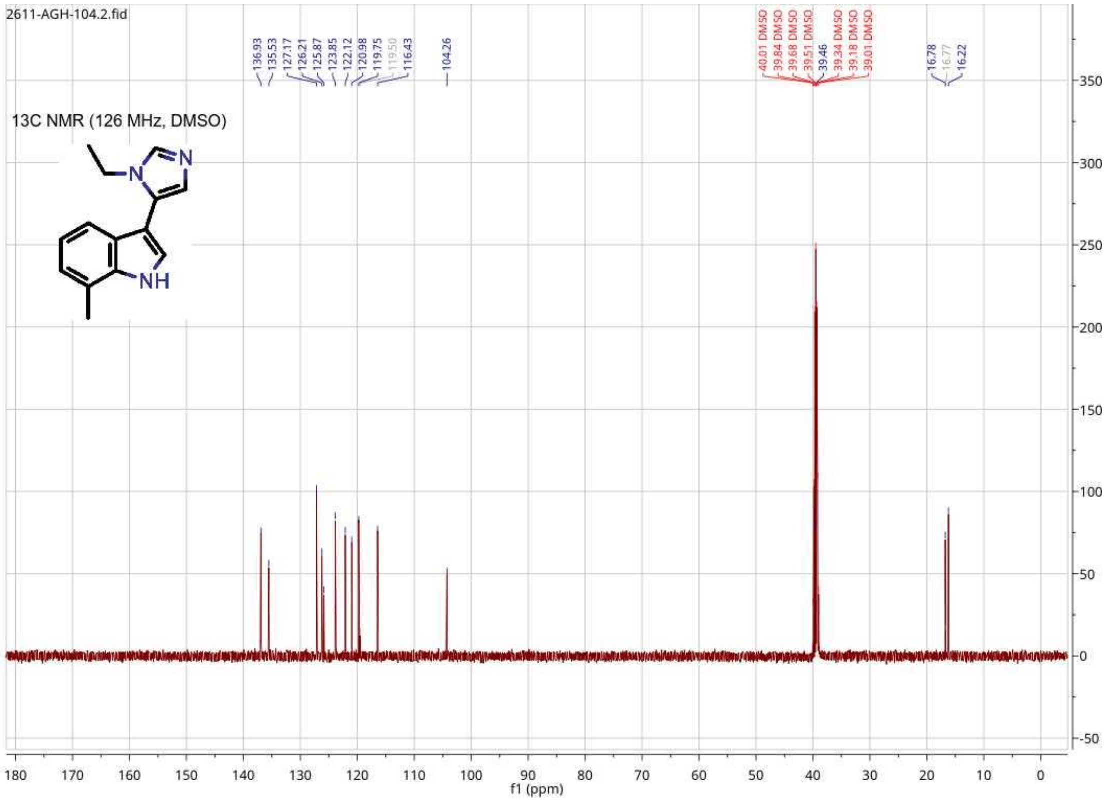

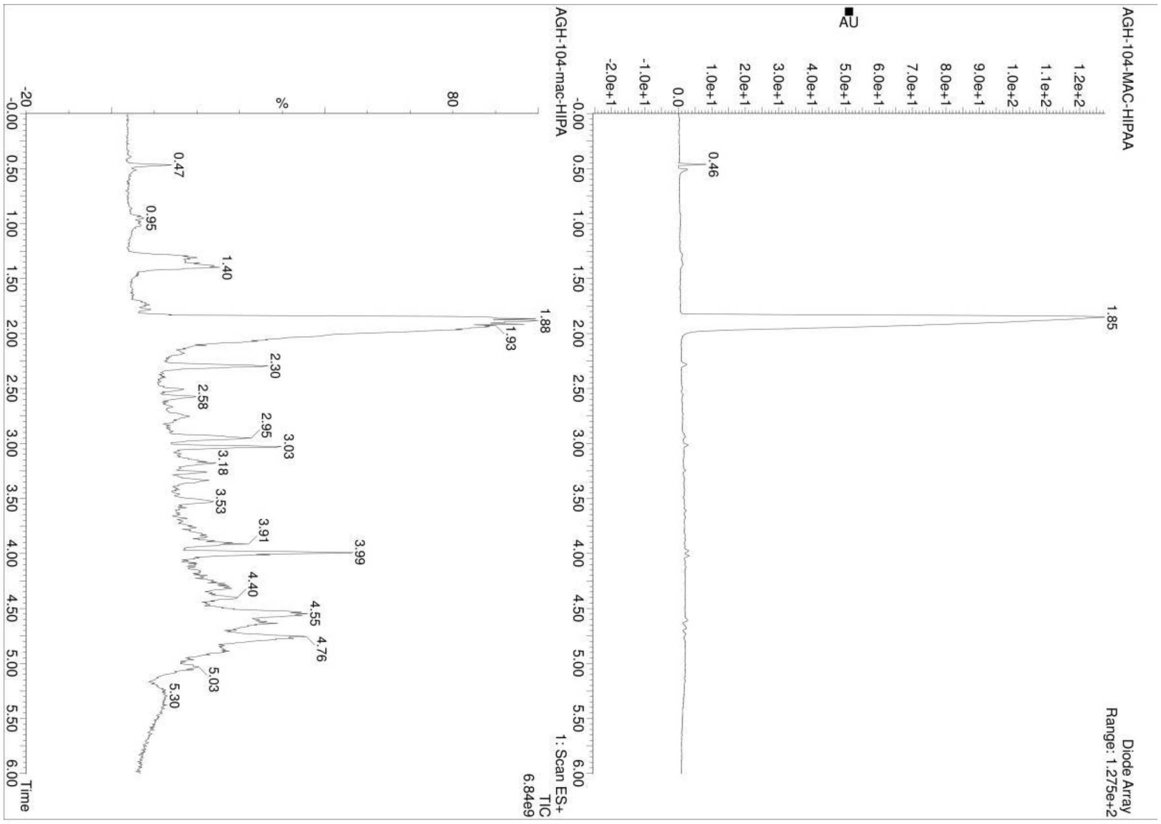
AGH-104-MAC-HIPAA
Diode Array
Range: 1.275e+2
AU
1.85
0.46
AGH-104-mac-HIPA
1: Scan ES+
TIC
6.84e9
1.88
1.93
0.47
0.95
1.40
2.30
2.58
2.95
3.03
3.18
3.53
3.91
3.99
4.40
4.55
4.76
5.03
5.30
Time

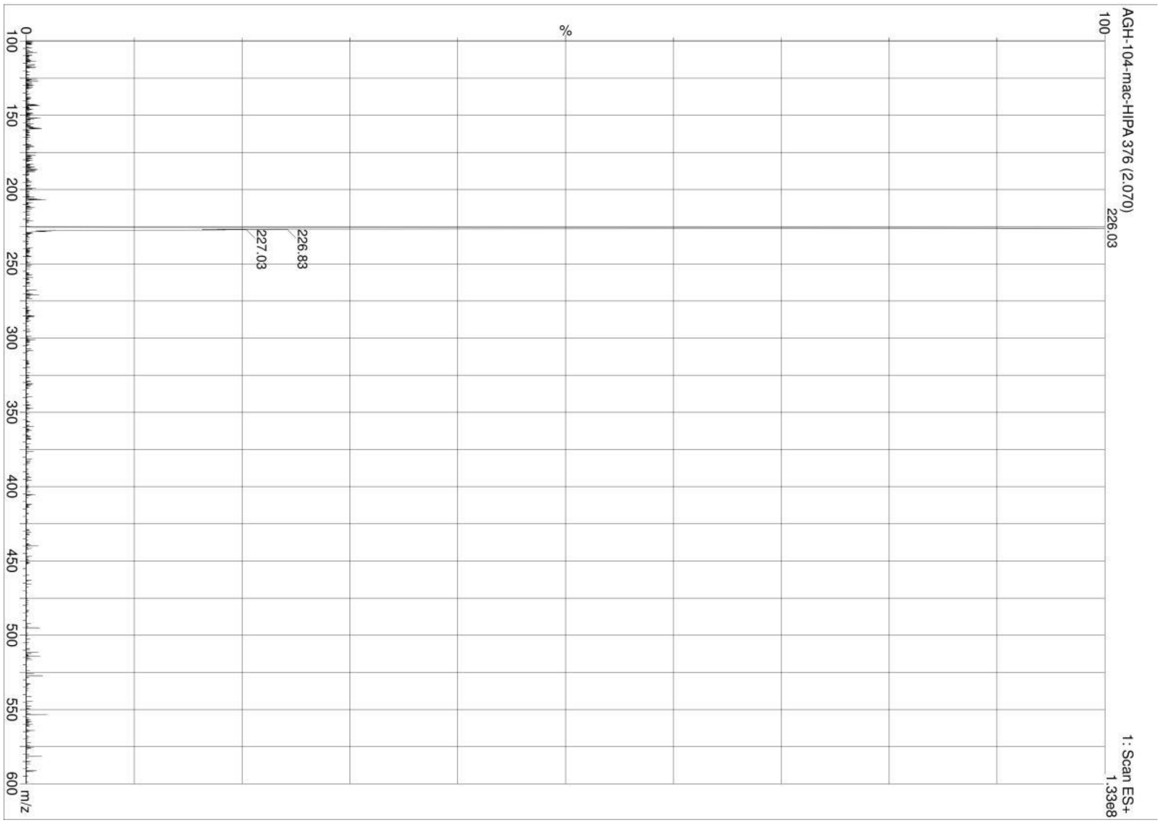
AGH-104-mac-HIPA 376 (2.070)
1: Scan ES+
1.33e8
226.03
226.83
227.03
m/z

1v: 3-(1-ethyl-1*H*-imidazol-5-yl)-5-methoxy-2-methyl-1*H*-indole (AGH-89)

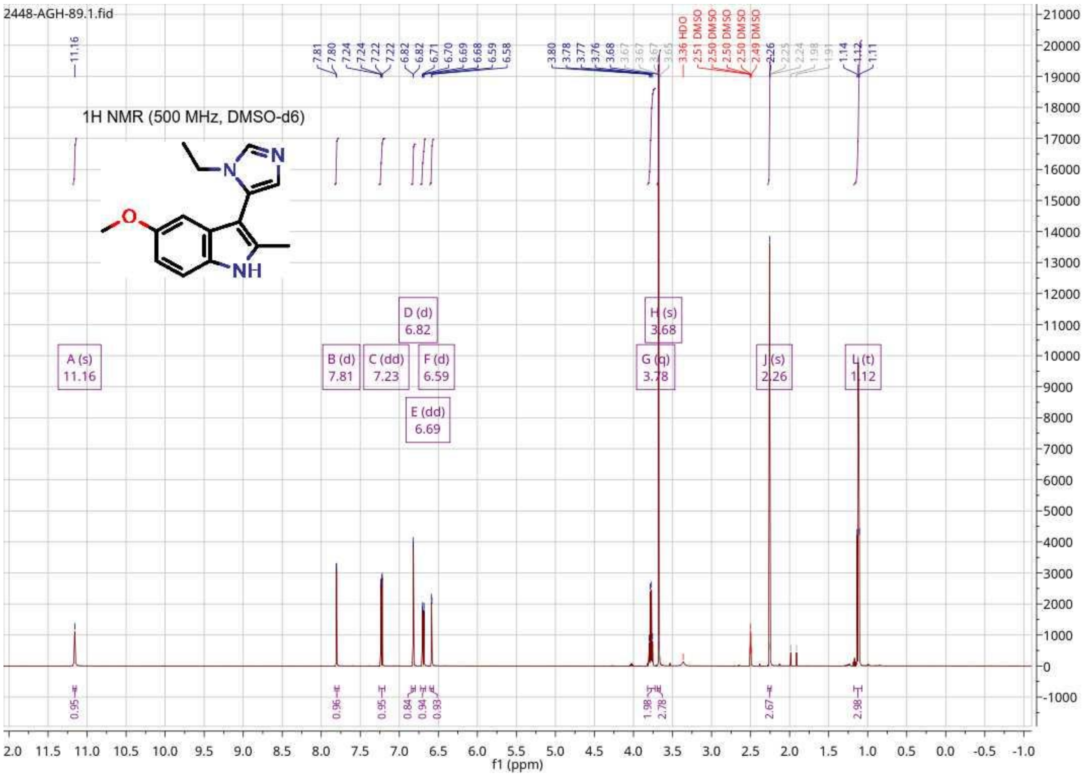

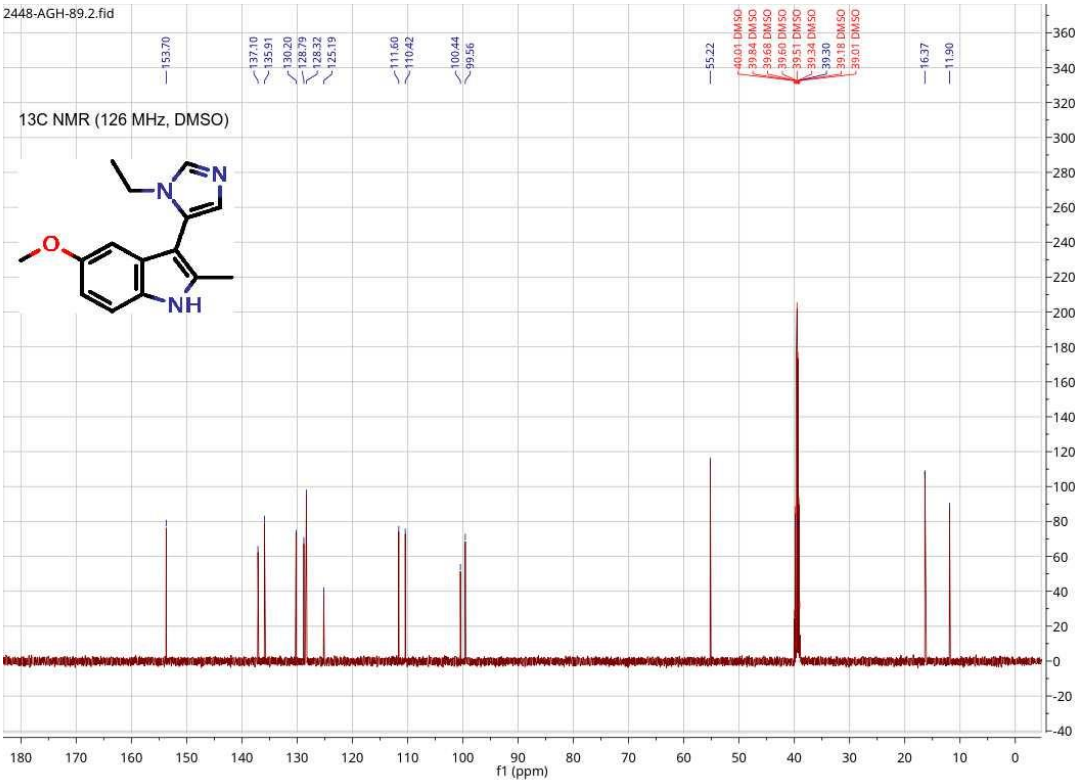

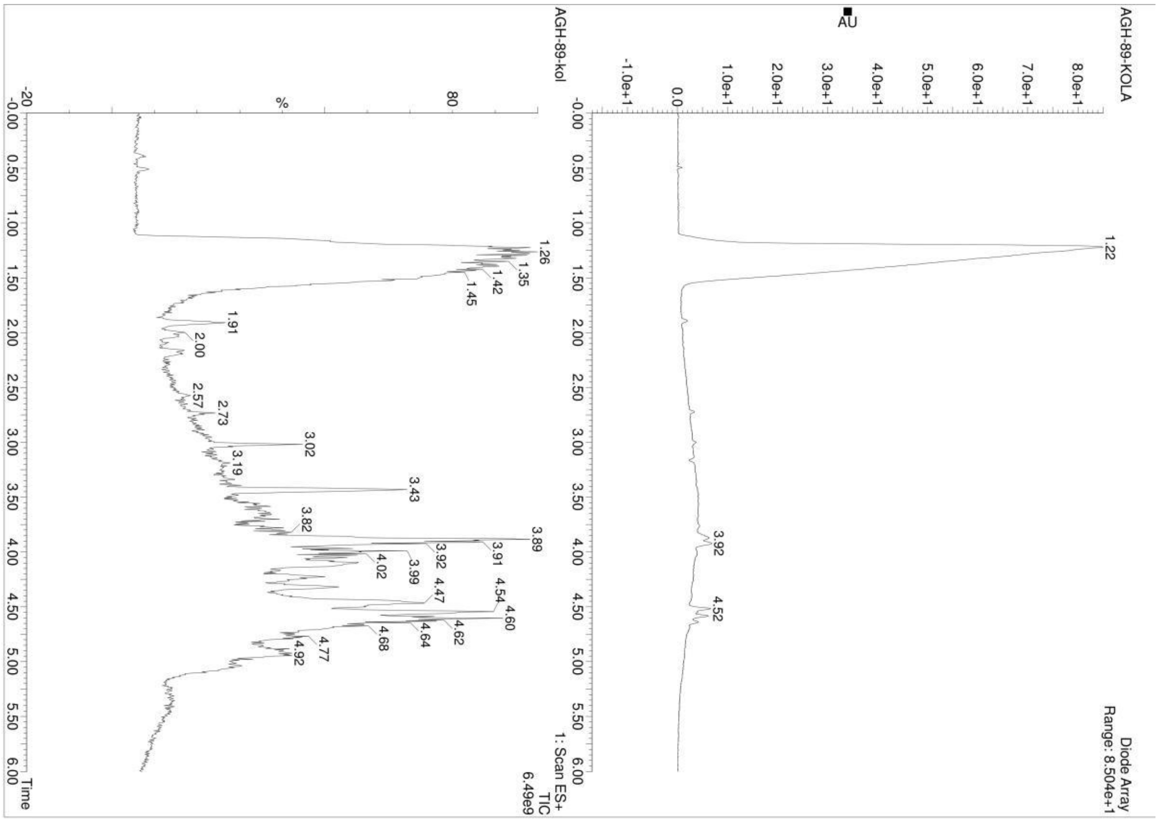
AGH-89-KOLA
Diode Array
Range: 8.504e+1
AU
1.22
3.92
4.52
AGH-89-kol
1: Scan ES+
TIC
6.49e9
Time

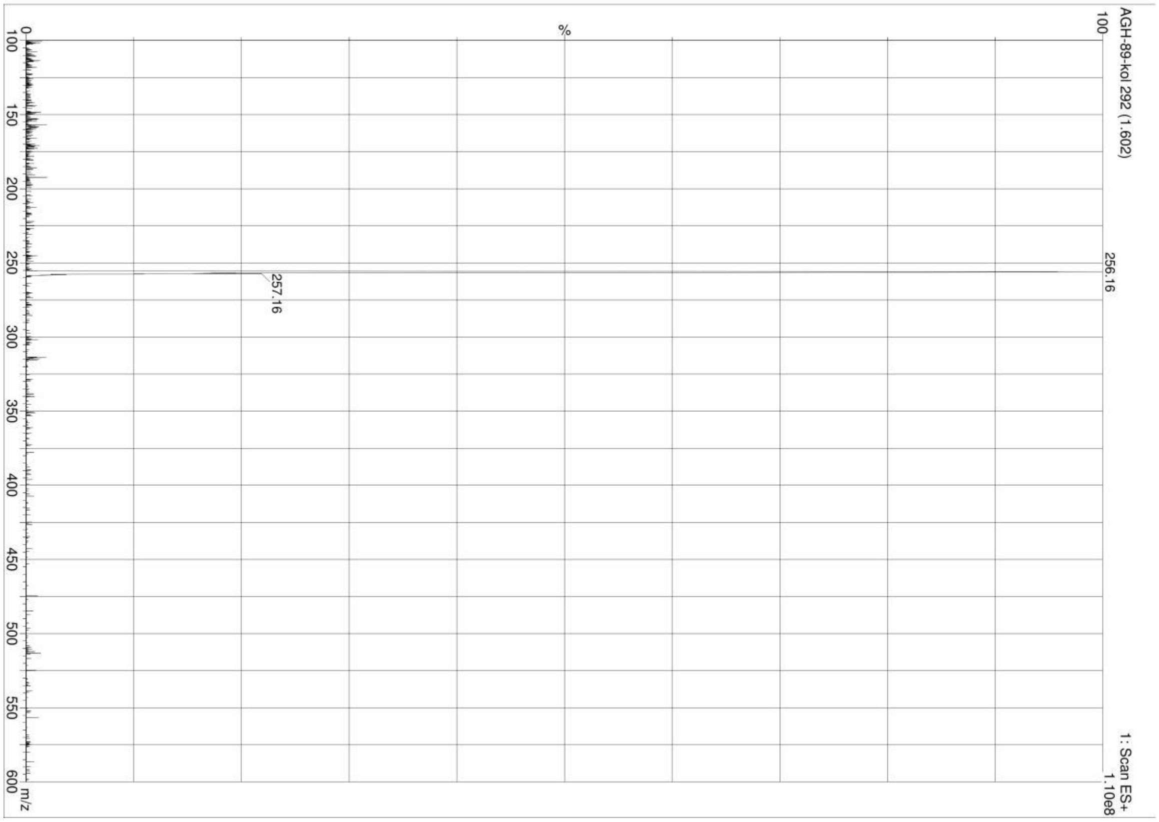
AGH-89-kol 292 (1.602)
1: Scan ES+
1.10e8
256.16
257.16
m/z

1w: 5-(1-ethyl-1*H*-imidazol-5-yl)-1*H*-indole (AGH-96)

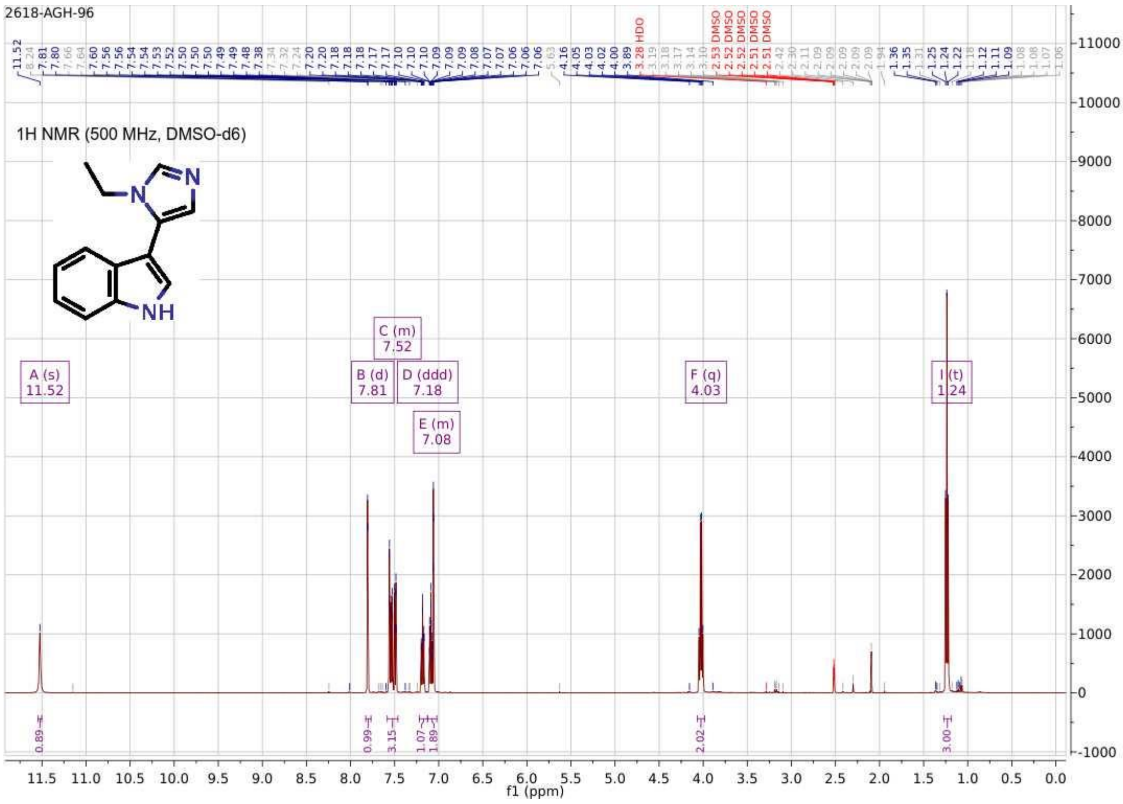

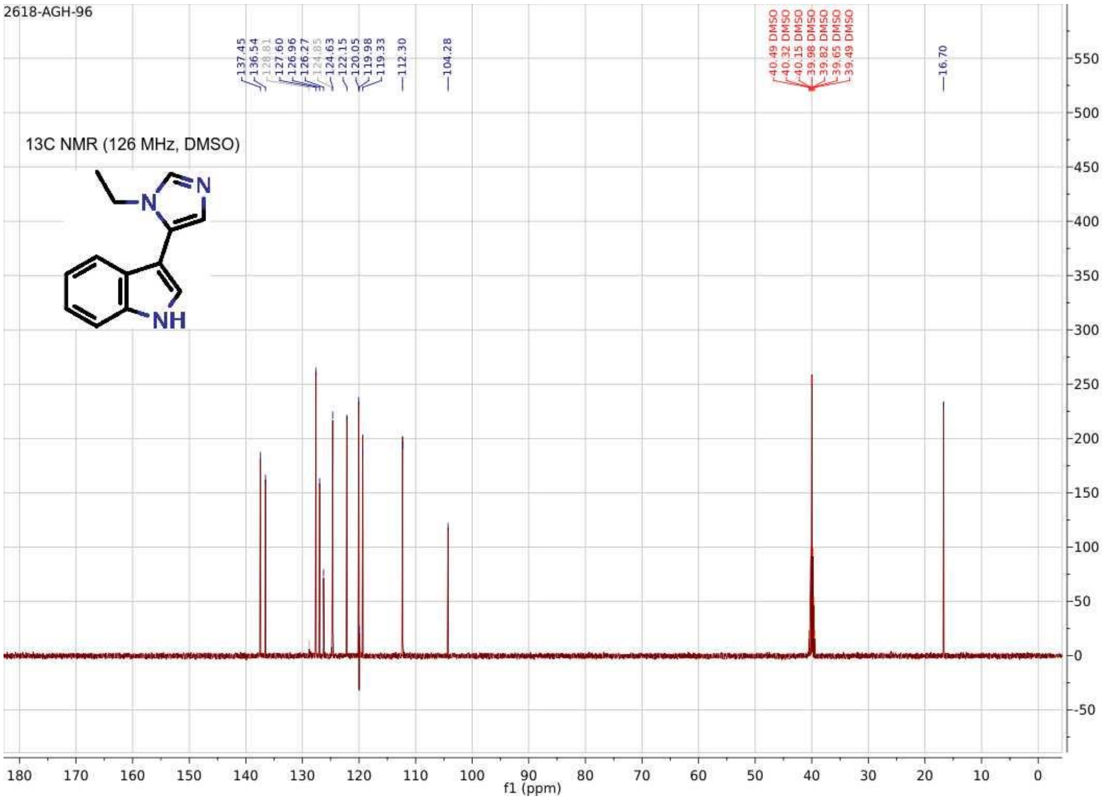

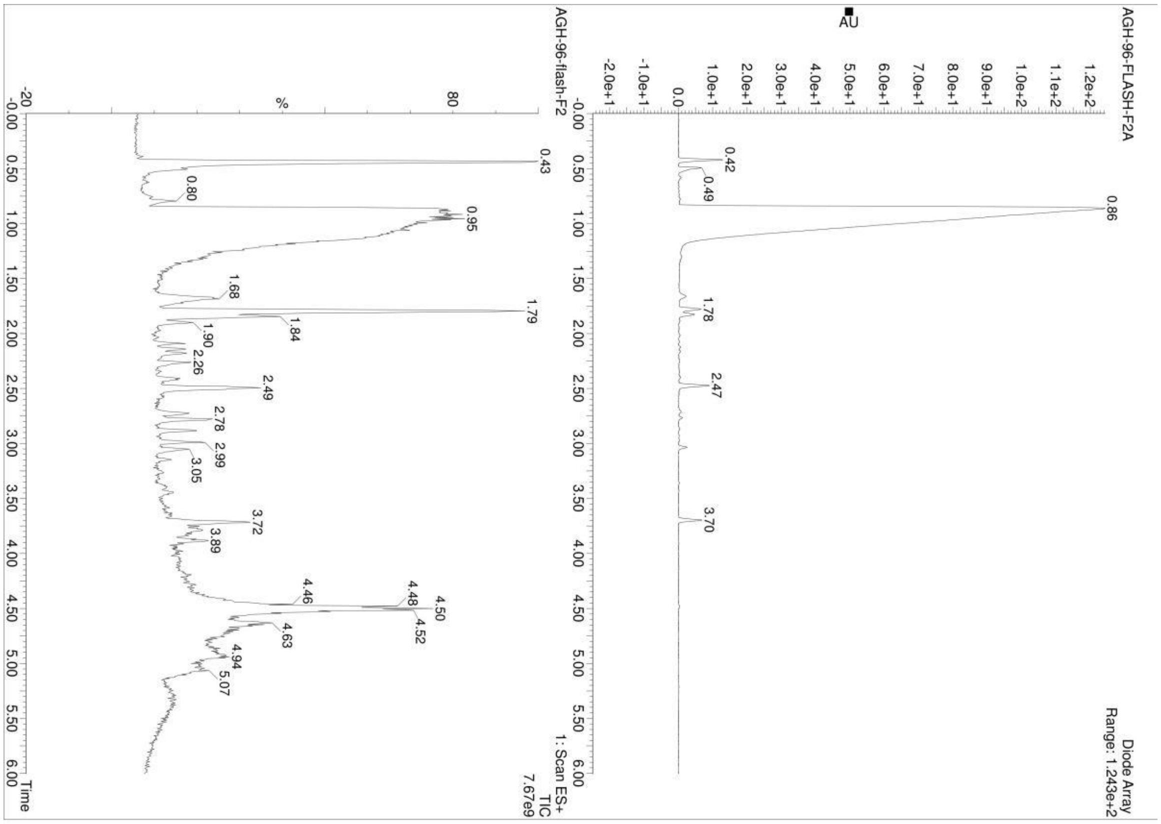
AGH-96-FLASH-F2A
Diode Array
Range: 1.243e+2
AU
AGH-96-flash-F2
1: Scan ES+
TIC
7.67e9
Time

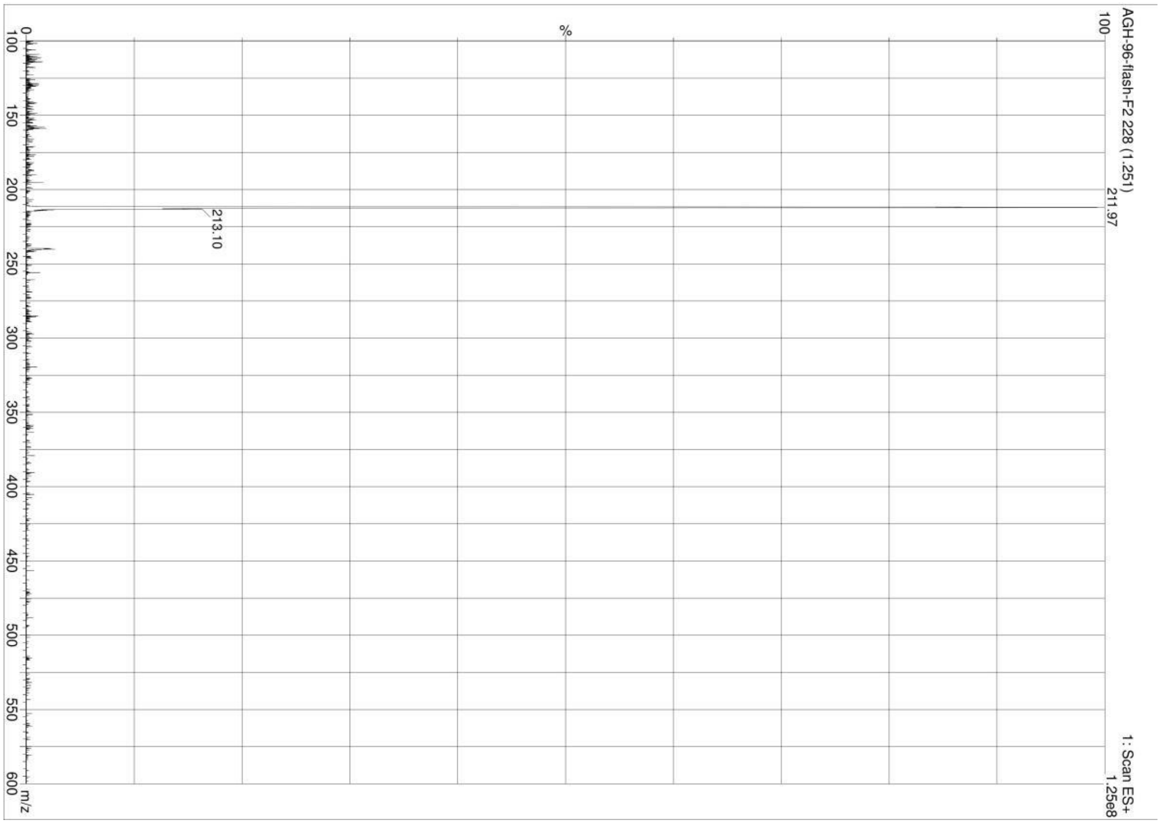
AGH-96-flash-F2 228 (1.251)
1: Scan ES+
1.25e8
211.97
213.10
m/z

1x: 3-(1*H*-imidazol-5-yl)-5-methoxy-1*H*-indole (AGH-117)

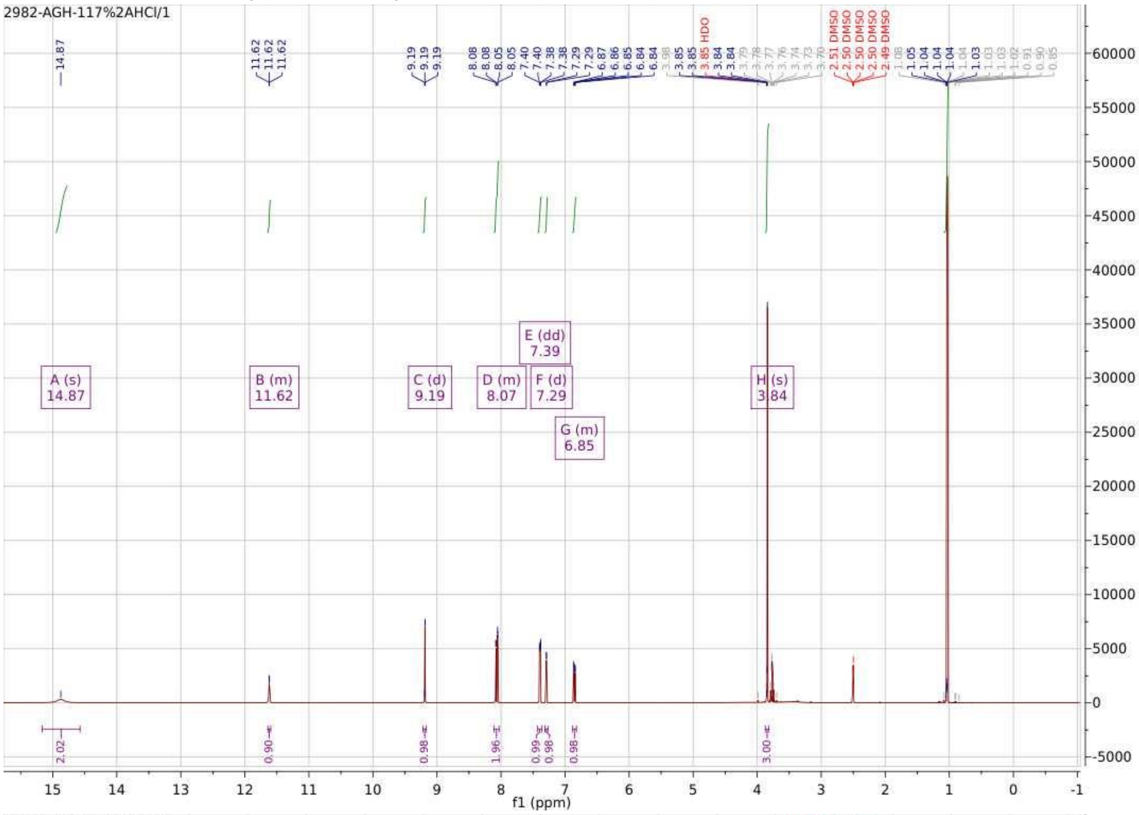

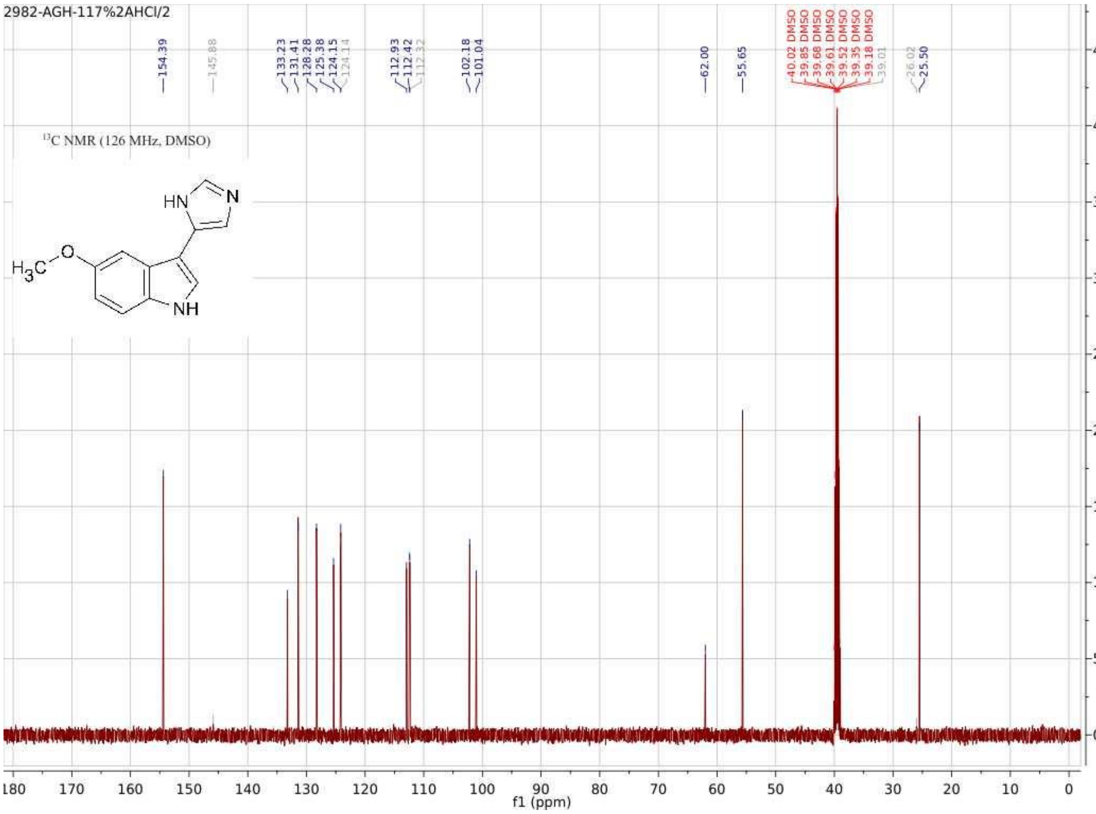

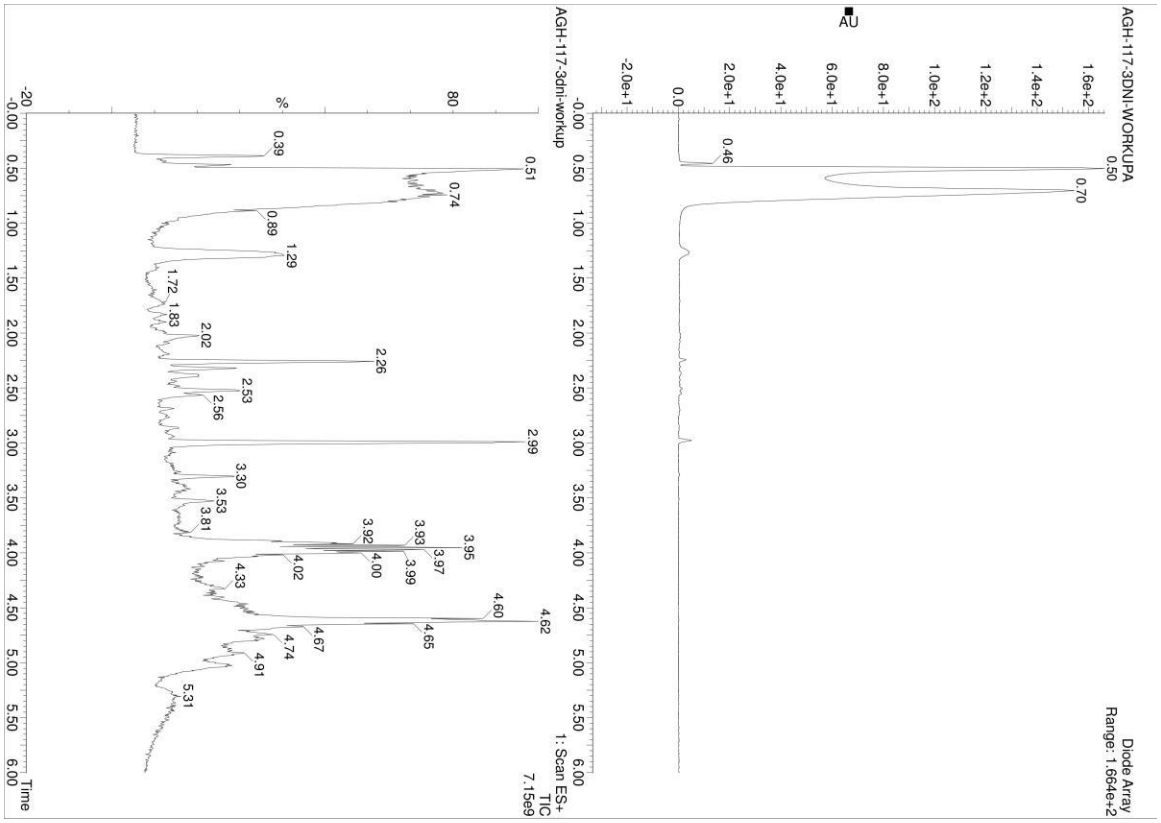
AGH-117-3DNI-WORKUPA
Diode Array
Range: 1.664e+2
AU
AGH-117-3dni-workup
1: Scan ES+
TIC
7.15e9
Time

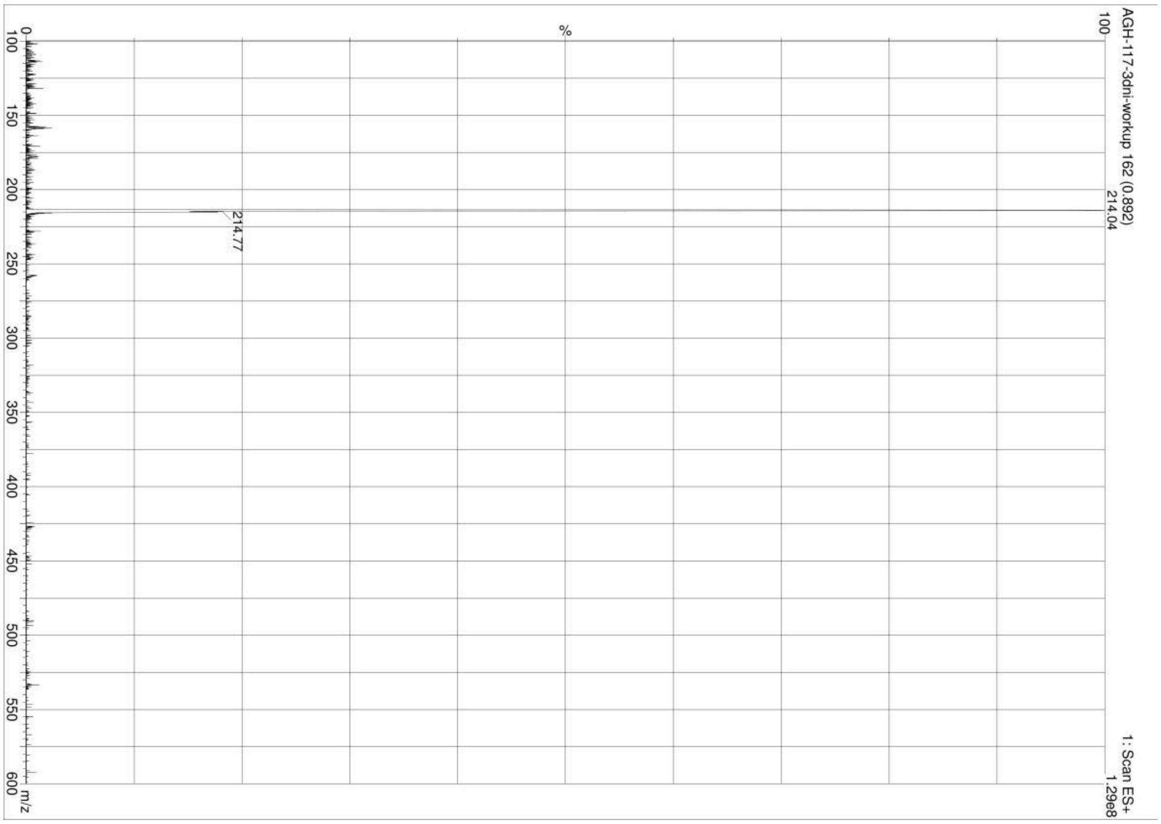
AGH-117-3dni-workup 162 (0.892)
1: Scan ES+
1.29e8
214.04
214.77
%
m/z

1y: 5-(benzyloxy)-3-(1-ethyl-1*H*-imidazol-5-yl)-1*H*-indole (AGH-101)

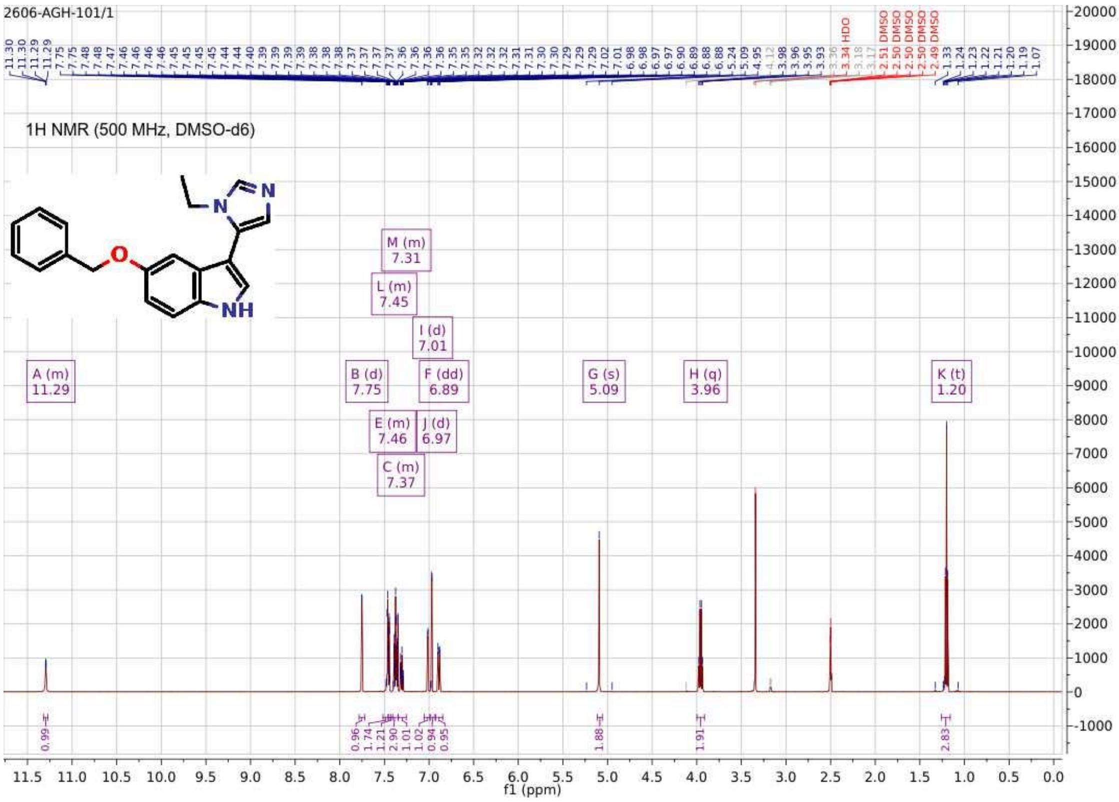

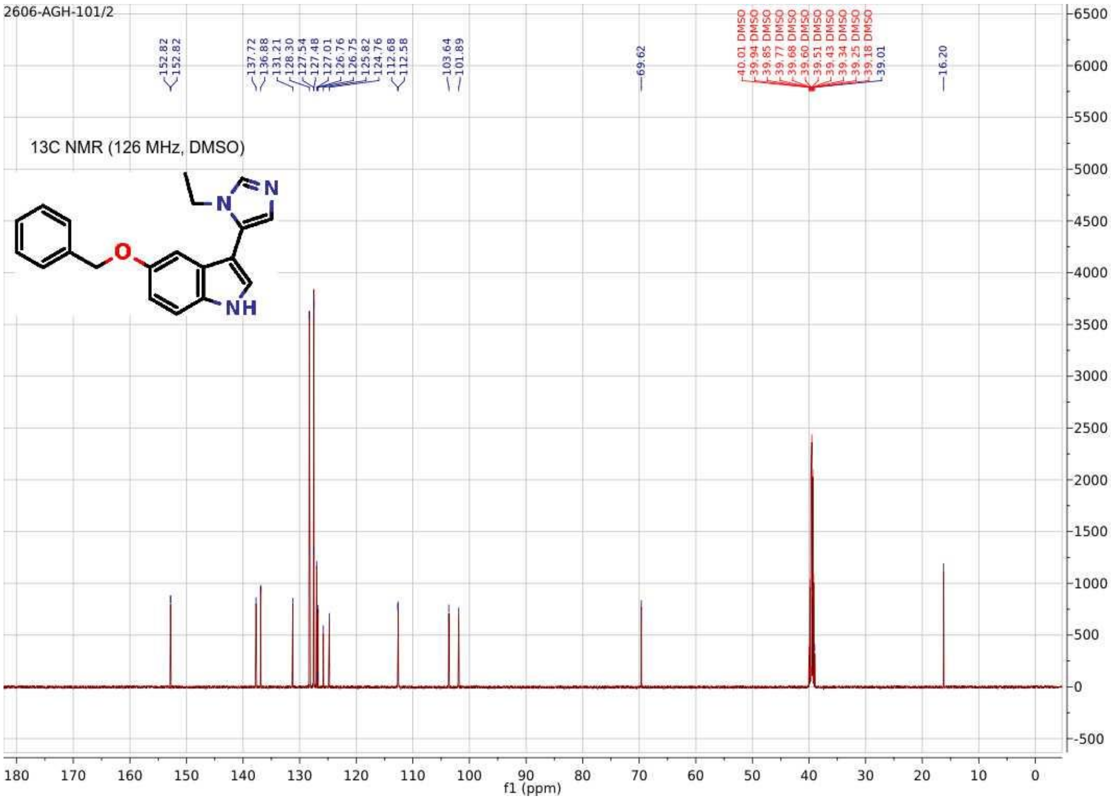

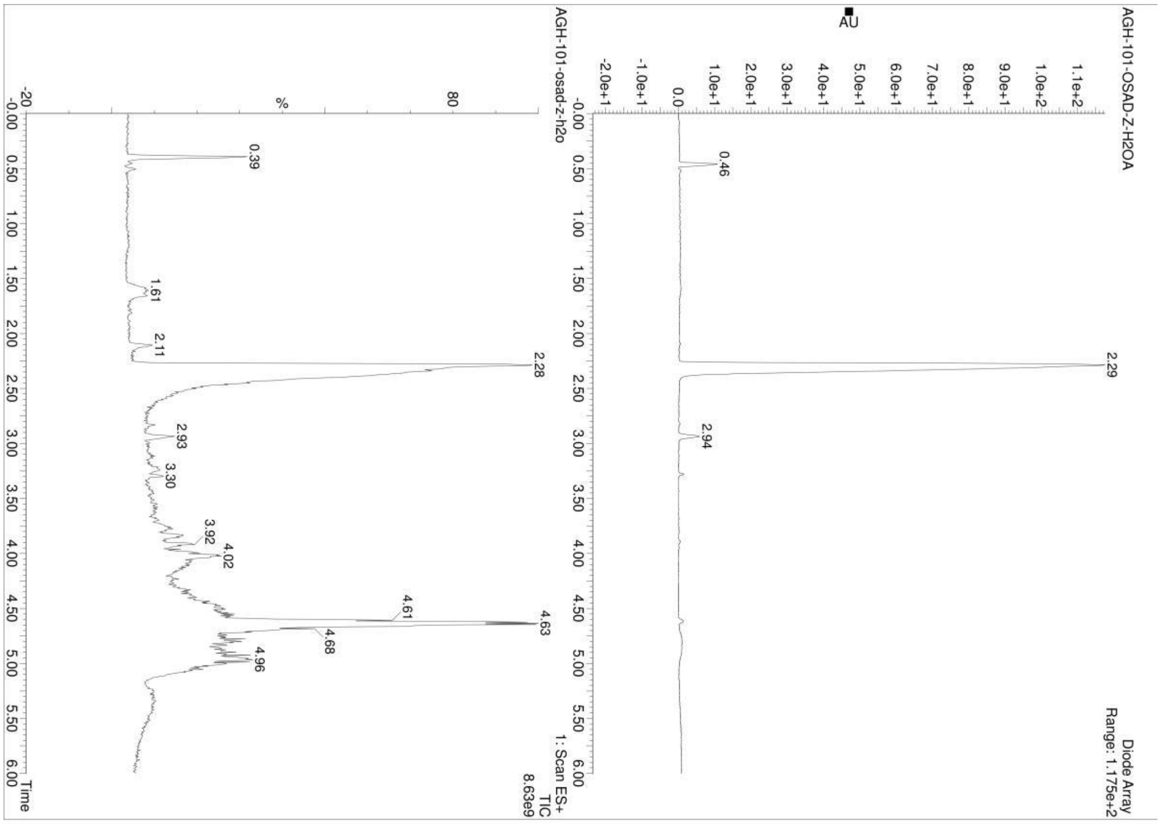

AGH-101-OSAD-Z-H2OA
Diode Array
Range: 1.175e+2
AU
2.29
0.46
2.94
AGH-101-osad-z-h2o
1: Scan ES+
TIC
8.63e9
%
2.28
0.39
1.61
2.11
2.93
3.30
3.92
4.02
4.61
4.63
4.68
4.96
Time

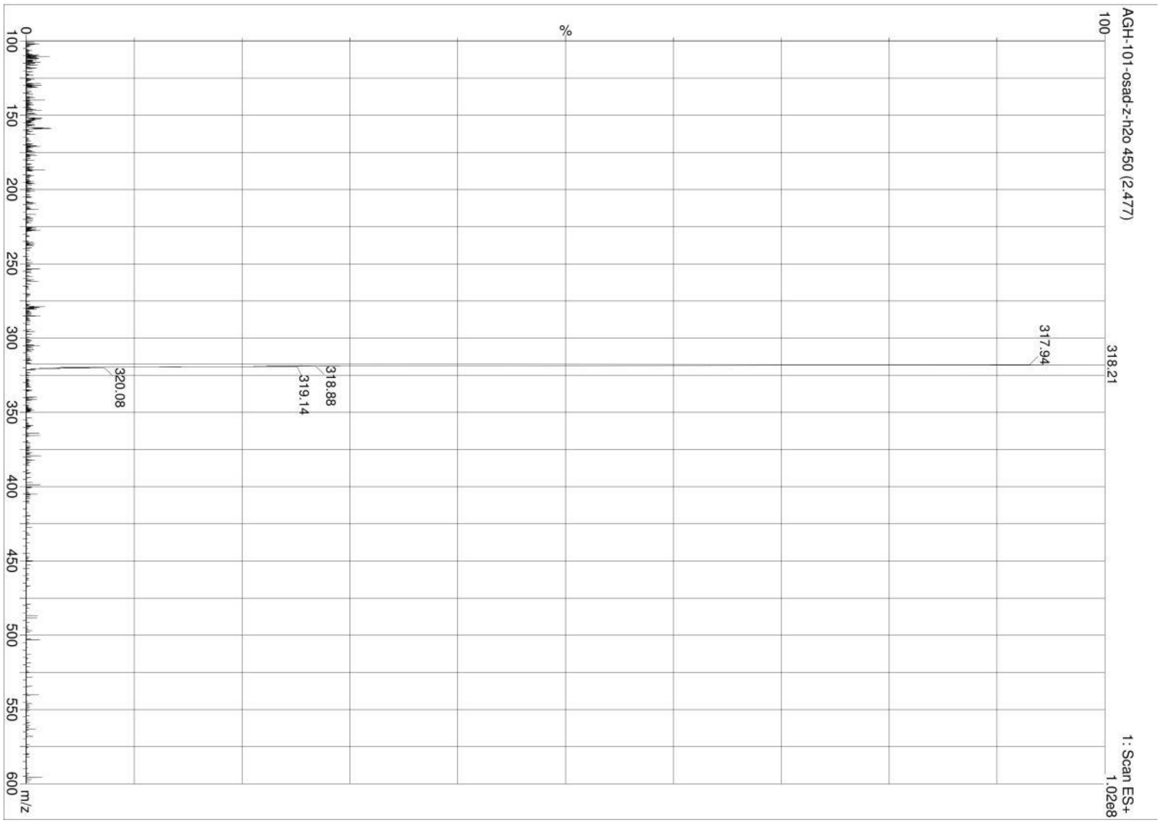

AGH-101-osad-z-h2o 450 (2.477)
1: Scan ES+
1.02e8
%
318.21
317.94
318.88
319.14
320.08
m/z

1z: 3-(1-ethyl-1*H*-imidazol-5-yl)-7-fluoro-5-iodo-1*H*-indole (AGH-116)

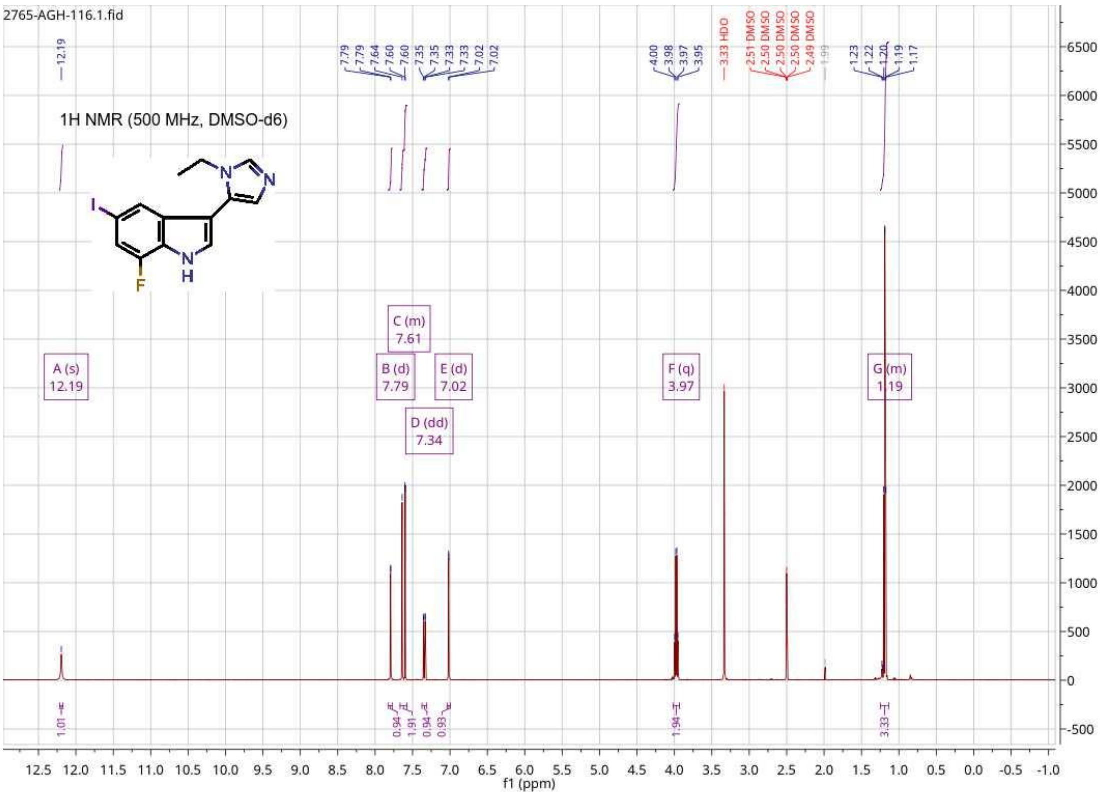

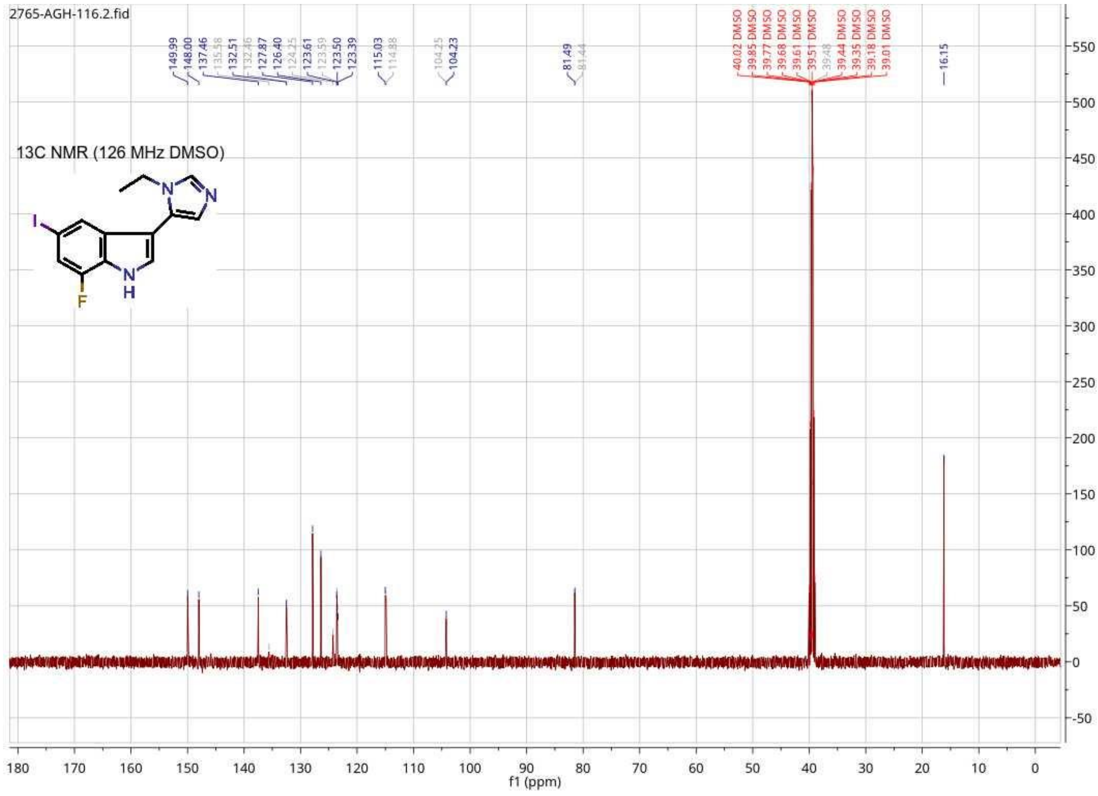

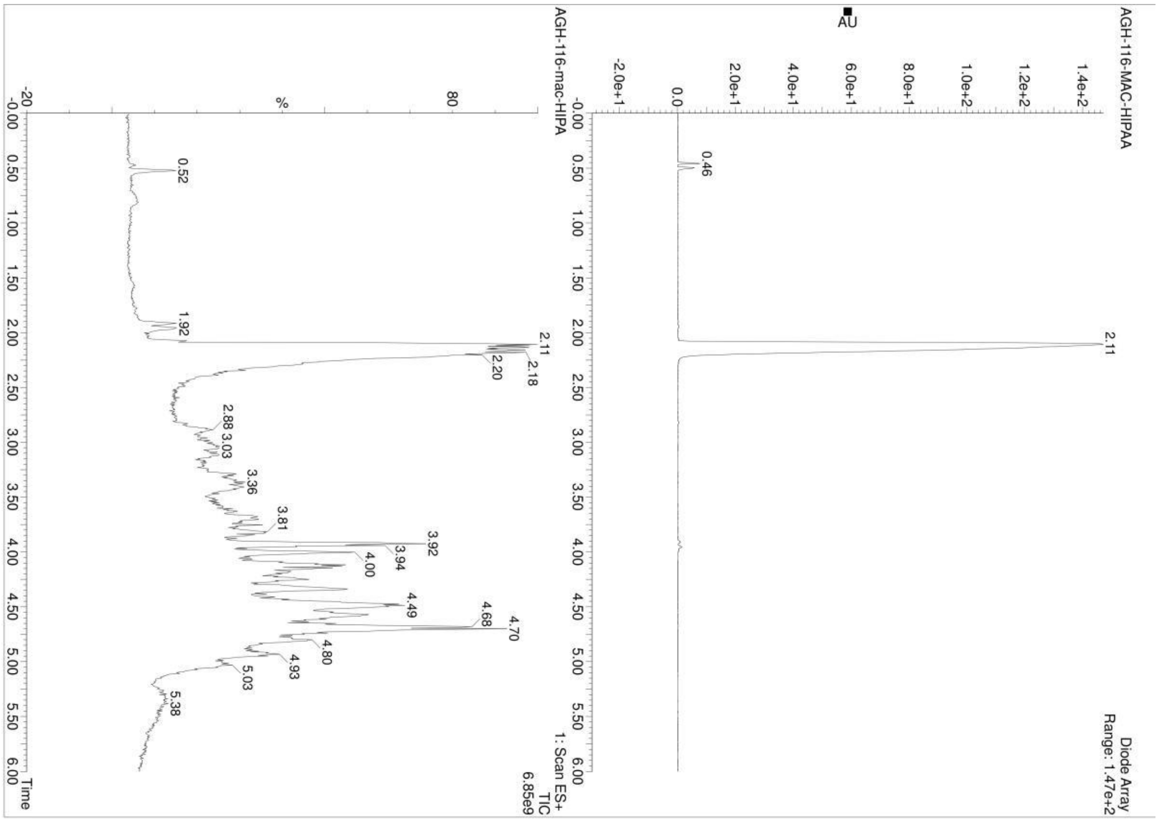
AGH-116-MAC-HIPAA
Diode Array
Range: 1.47e+2
AU
AGH-116-mac-HIPA
1: Scan ES+
TIC
6.85e9
Time

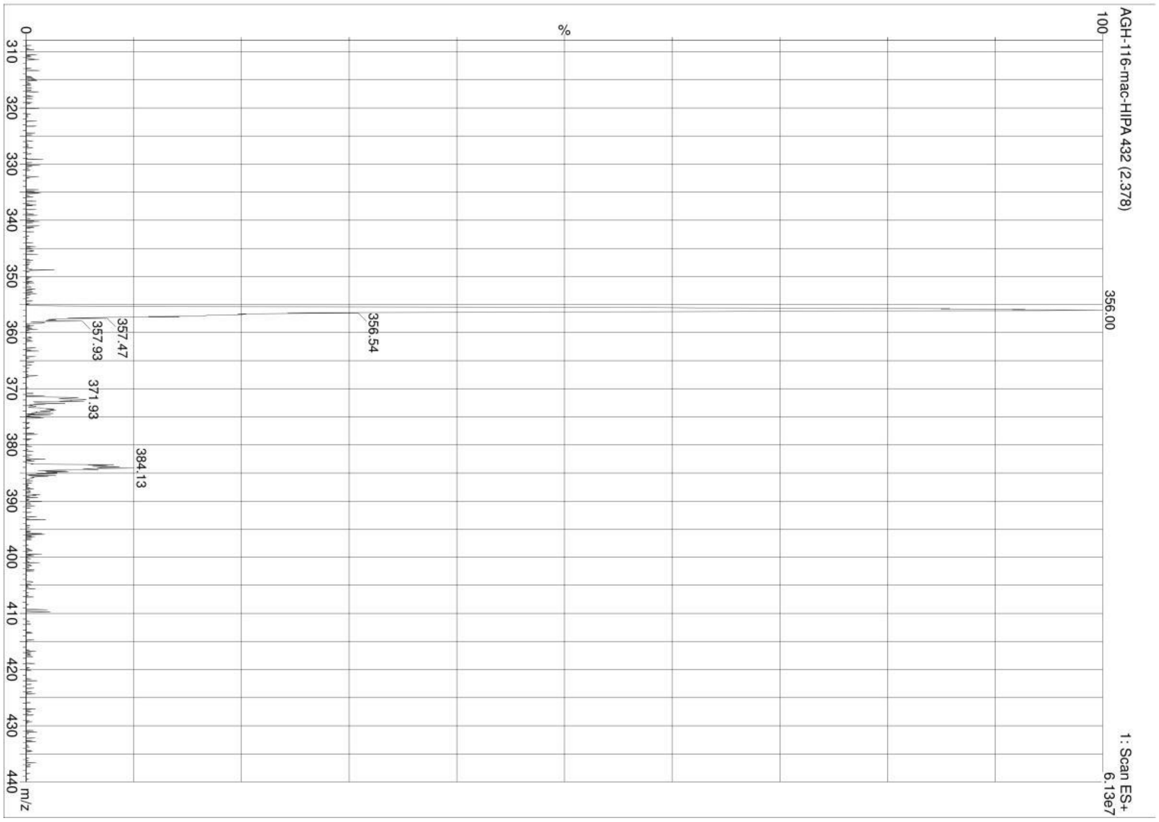
AGH-116-mac-HIPA 432 (2.378)
1: Scan ES+
6.13e7
356.00
356.54
357.47
357.93
371.93
384.13
m/z

1za: 3-(1-ethyl-1*H*-imidazol-5-yl)-1*H*-indole-5-carboxamide (AH-494)

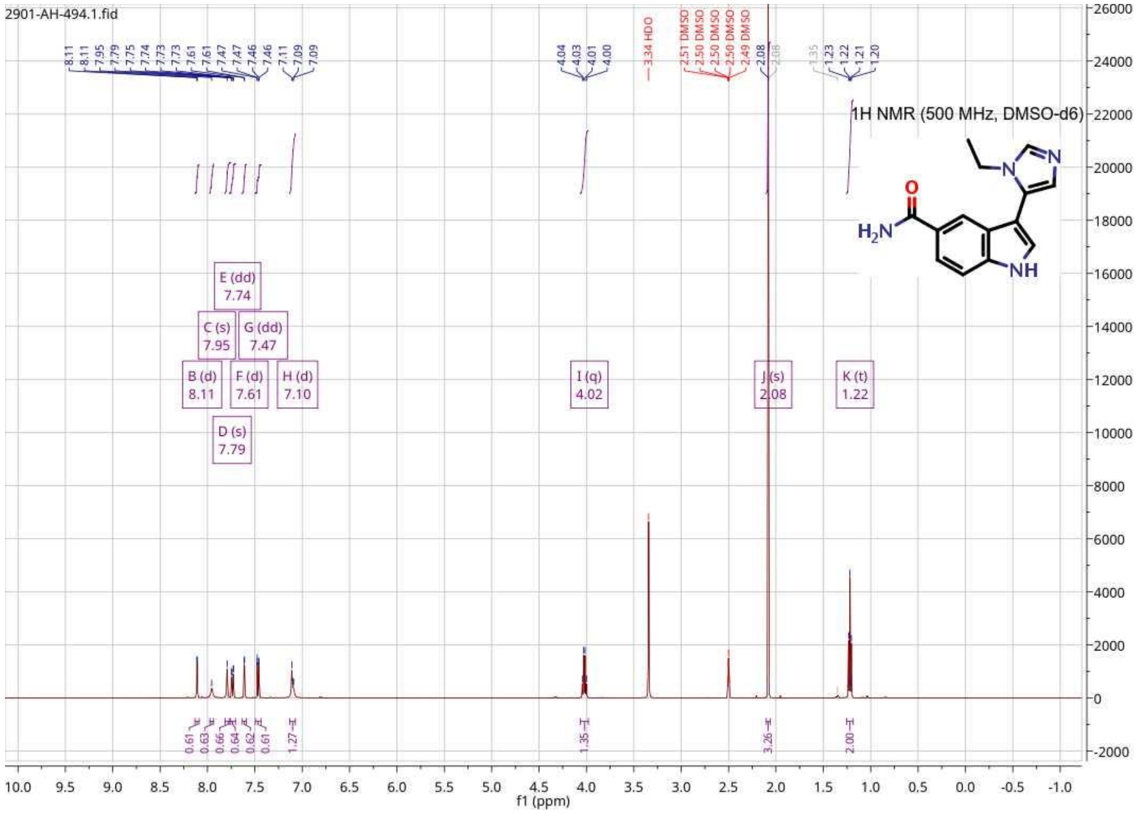

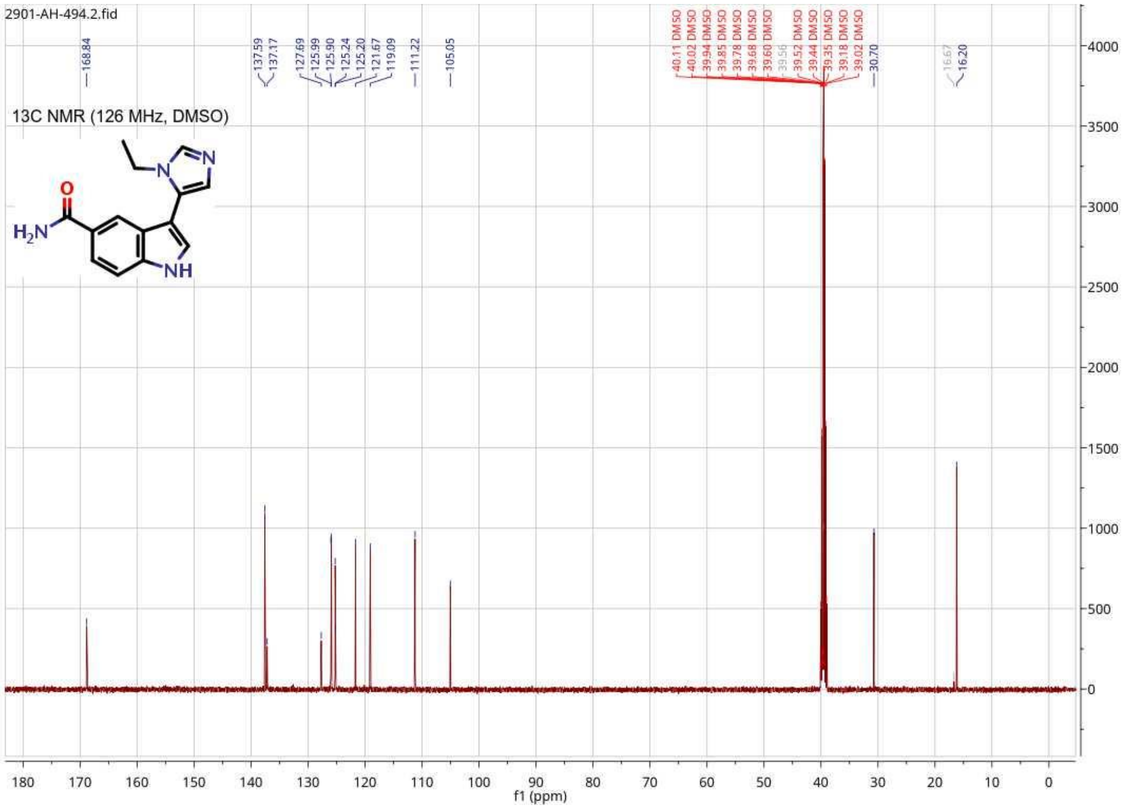

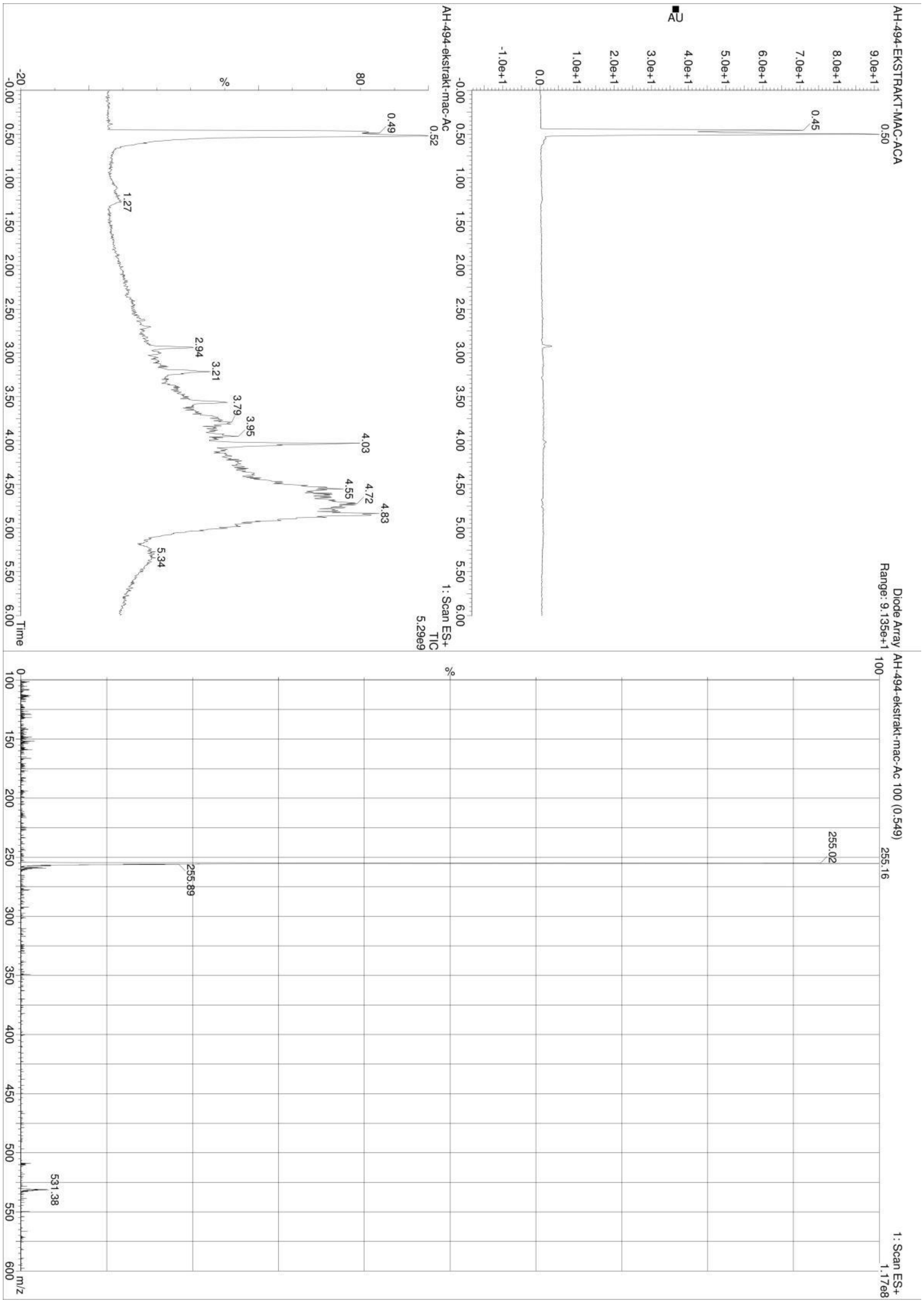
AH-494-EKSTRAKT-MAC-ACA
AU
Diode Array
Range: 9.135e+1
0.45
0.50
AH-494-ekstrakt-mac-Ac
1: Scan ES+
TIC
5.29e9
0.49
0.52
1.27
2.94
3.21
3.79
3.95
4.03
4.55
4.72
4.83
5.34
Time
AH-494-ekstrakt-mac-Ac 100 (0.549)
1: Scan ES+
1.17e8
255.02
255.16
255.89
531.38
m/z

2a: 5-(4-methoxyphenyl)-1-methyl-4-nitro-1*H*-imidazole (AGH-54)

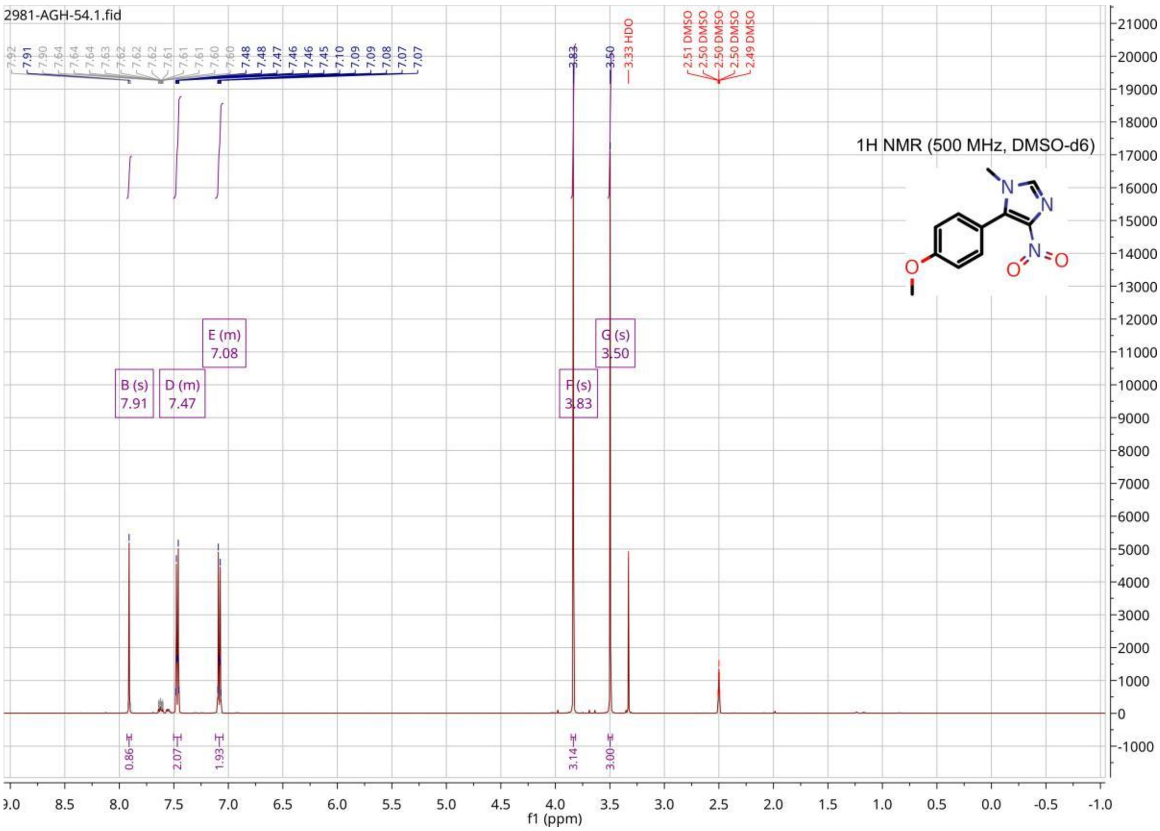

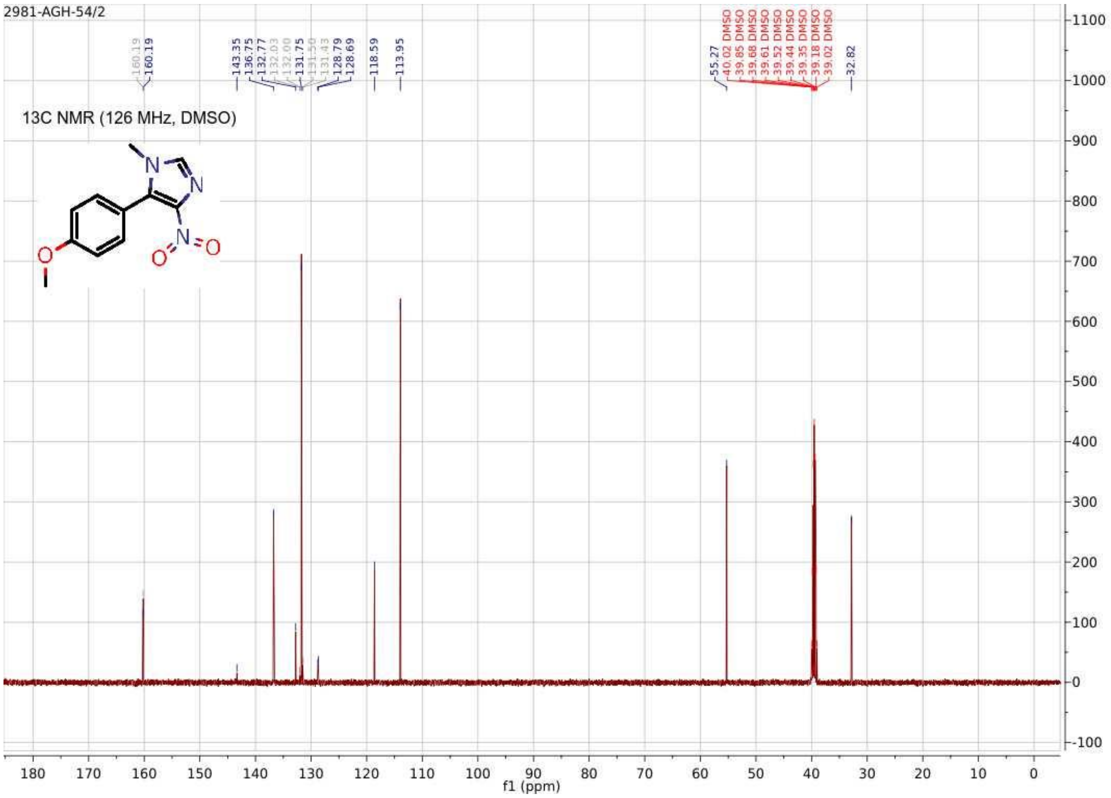

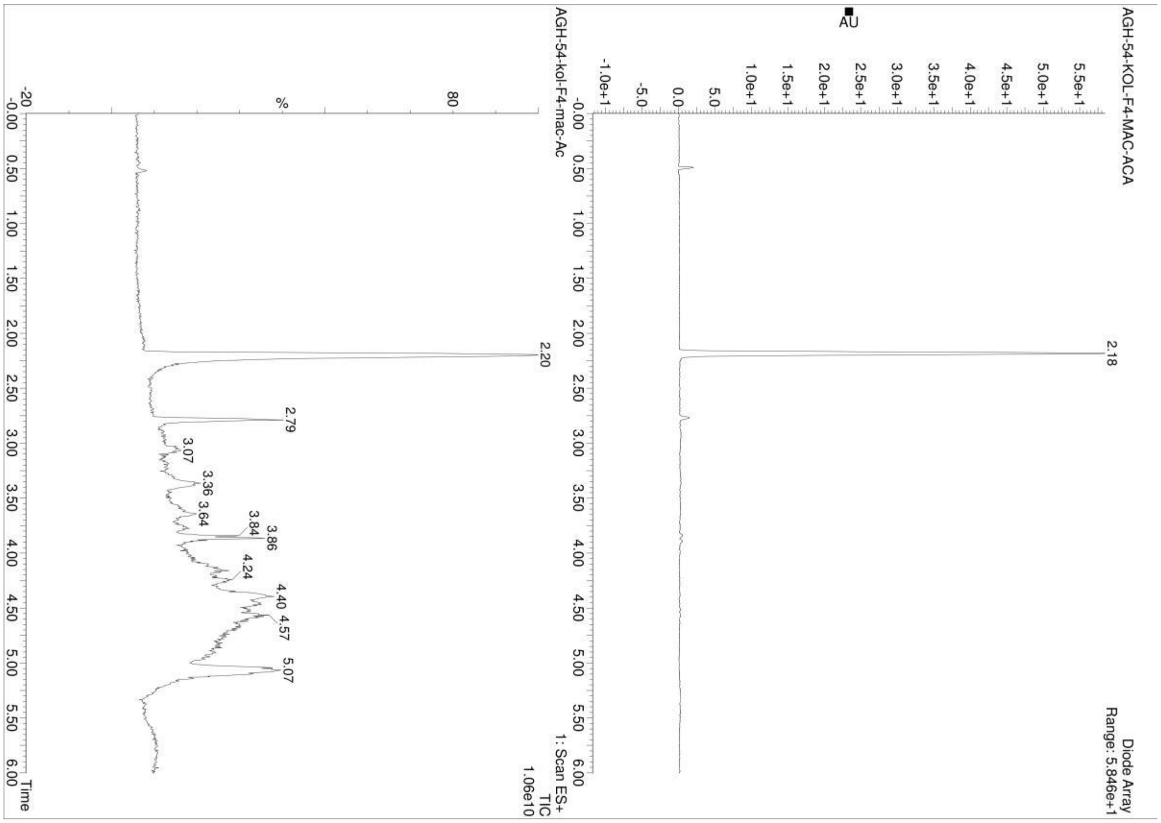
AGH-54-KOL-F4-MAC-ACA
AU
Diode Array
Range: 5.846e+1
2.18
AGH-54-kol-F4-mac-Ac
1: Scan ES+
TIC
1.06e10
%
2.20
2.79
3.07
3.36
3.64
3.84
3.86
4.24
4.40
4.57
5.07
Time

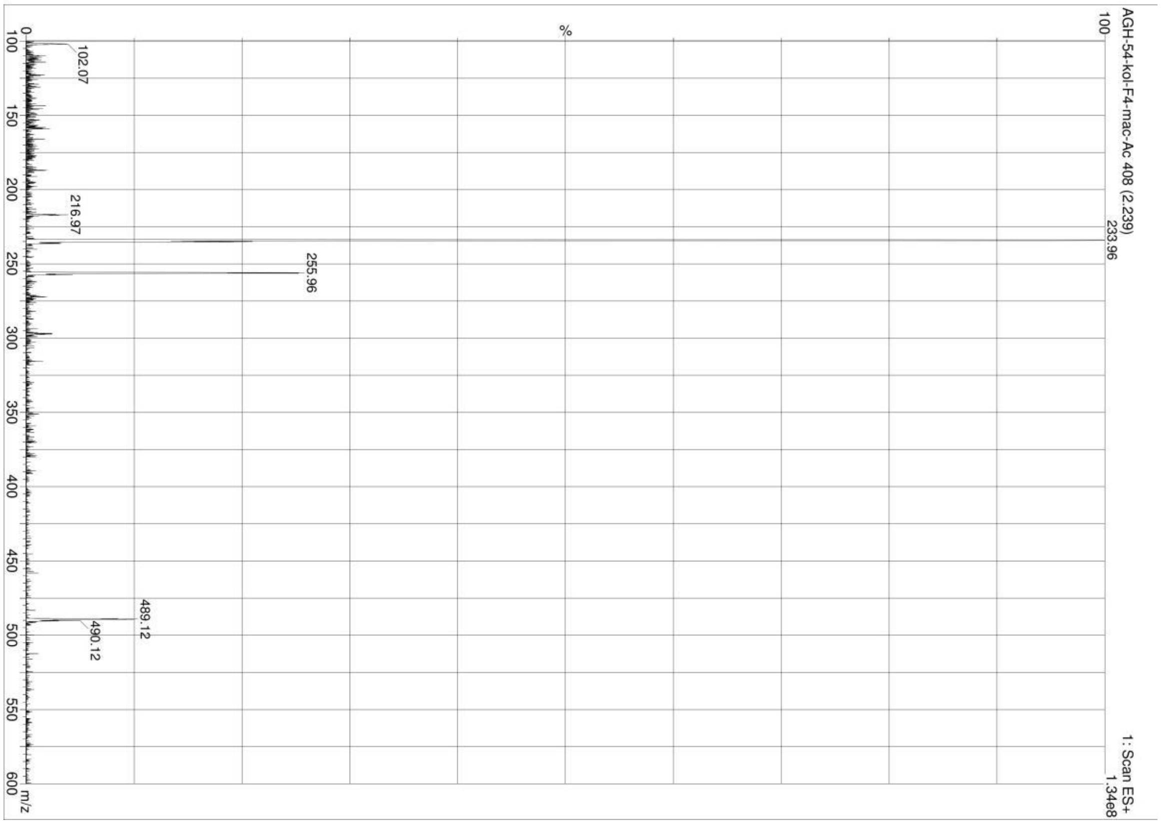
AGH-54-kol-F4-mac-Ac 408 (2.239)
1: Scan ES+
1.34e8
%
233.96
255.96
216.97
102.07
489.12
490.12
m/z

2b: 5-(4-methoxyphenyl)-1-methyl-1*H*-imidazole (AH-445)

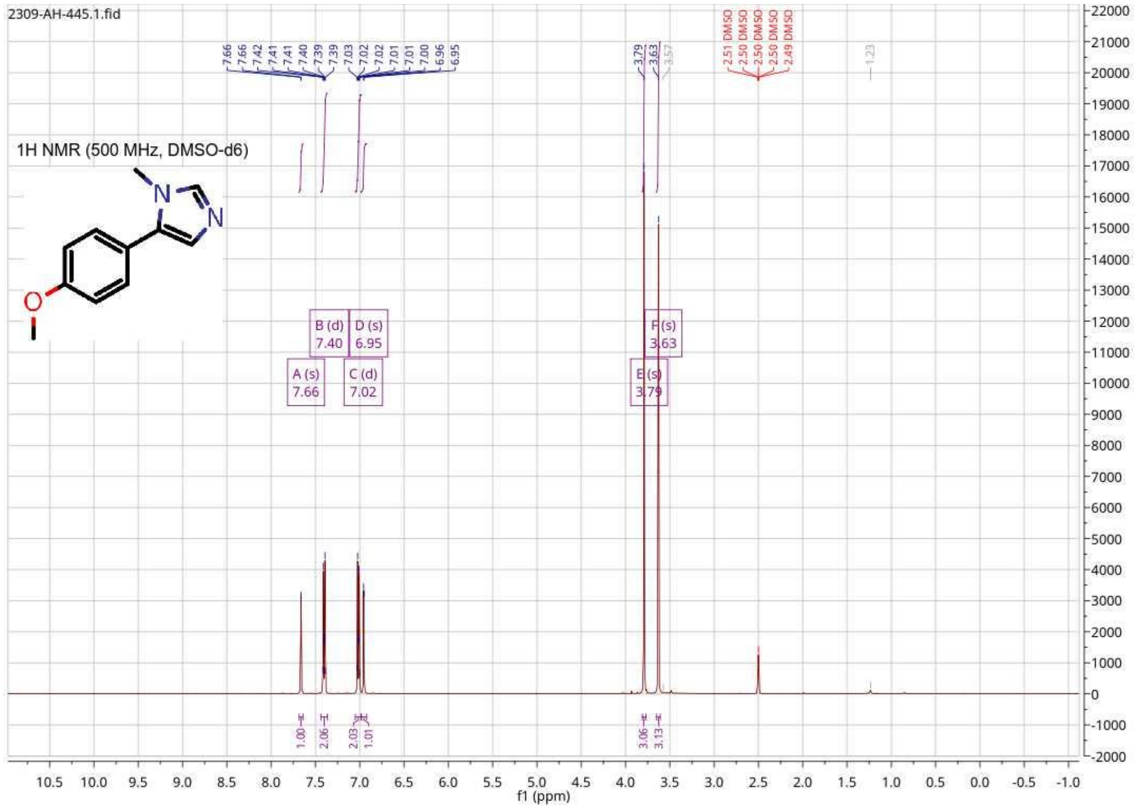

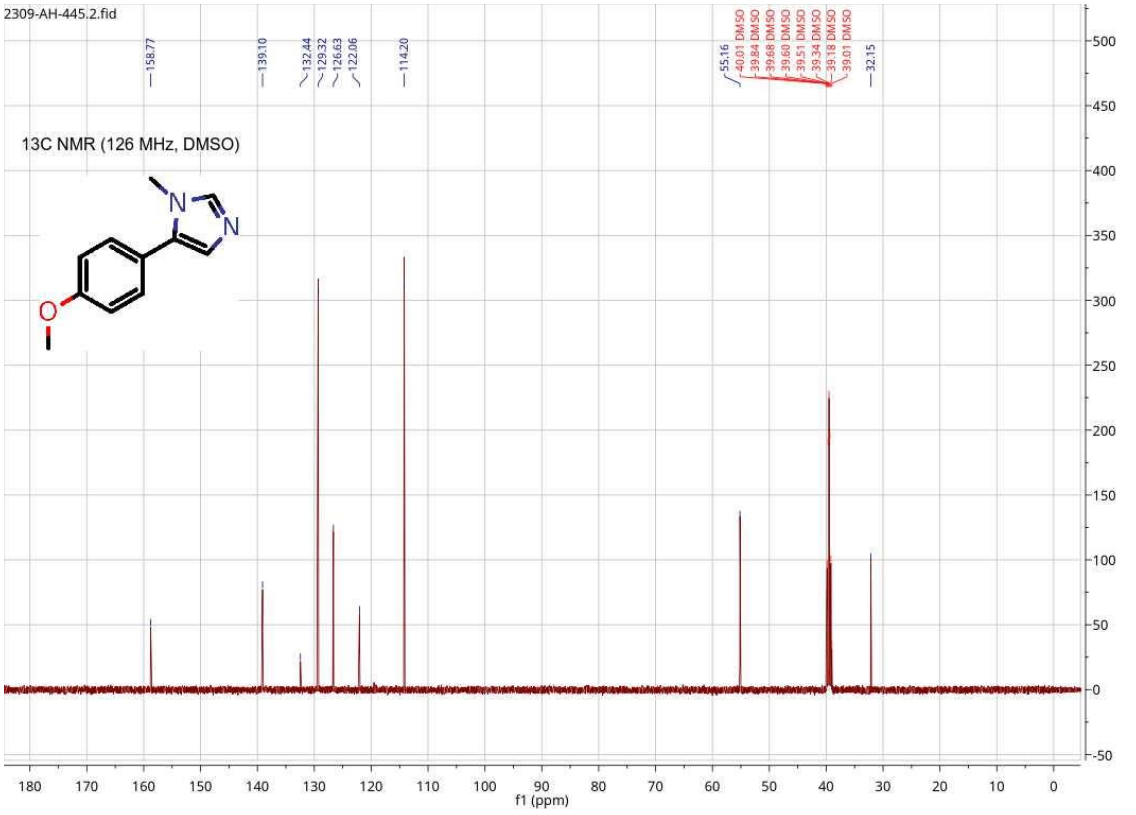

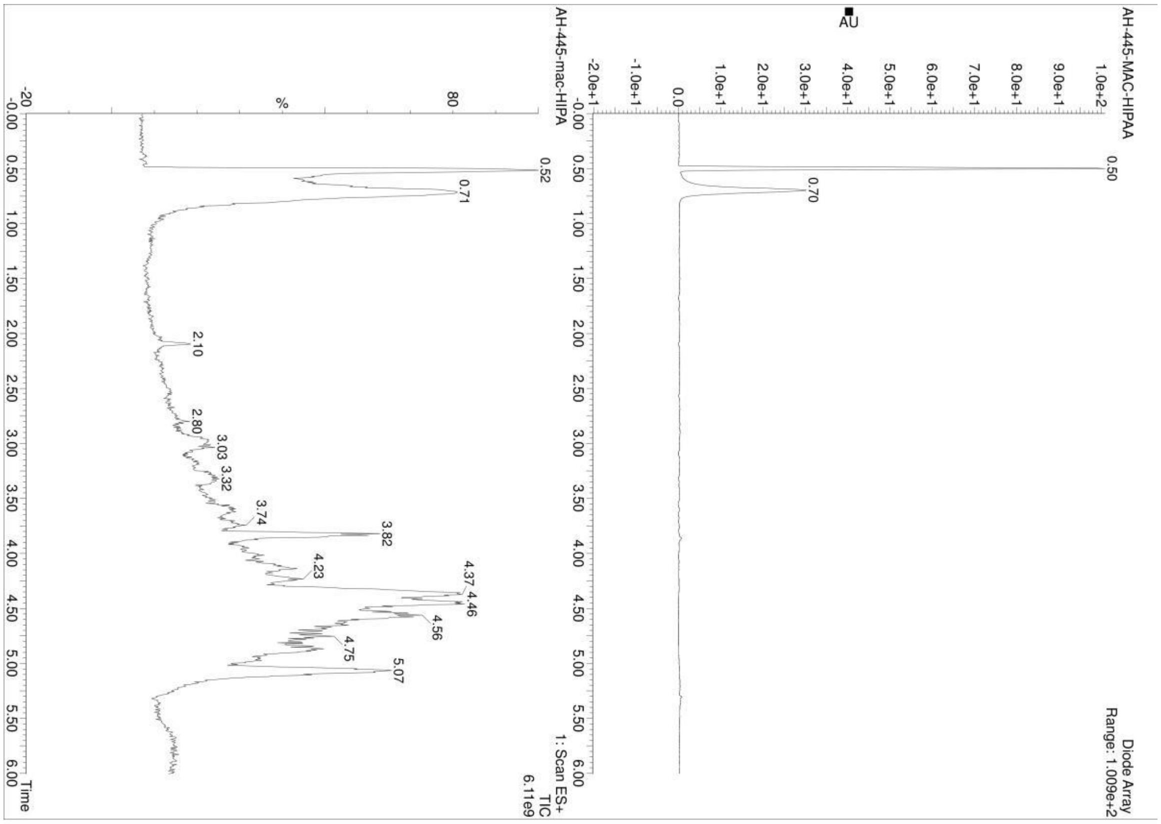

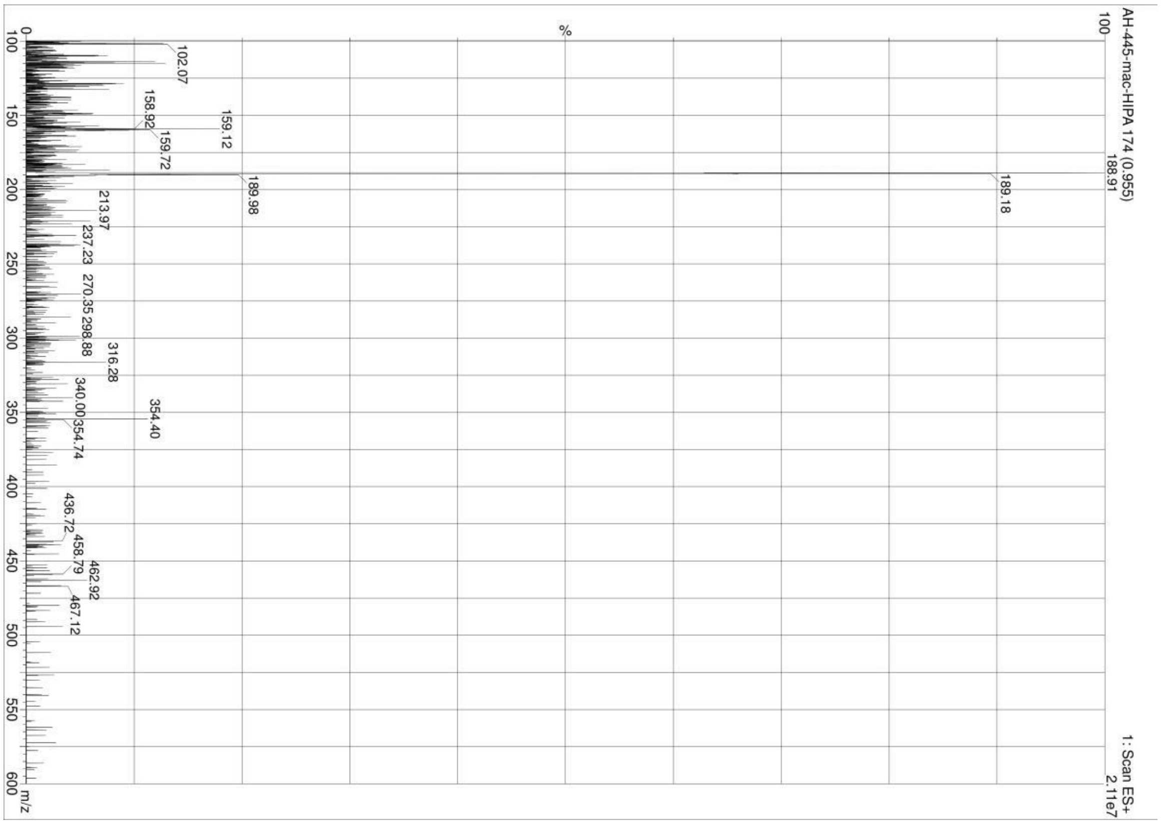

2c: 5-(3-methoxyphenyl)-1-methyl-1*H*-imidazole (AGH-47)

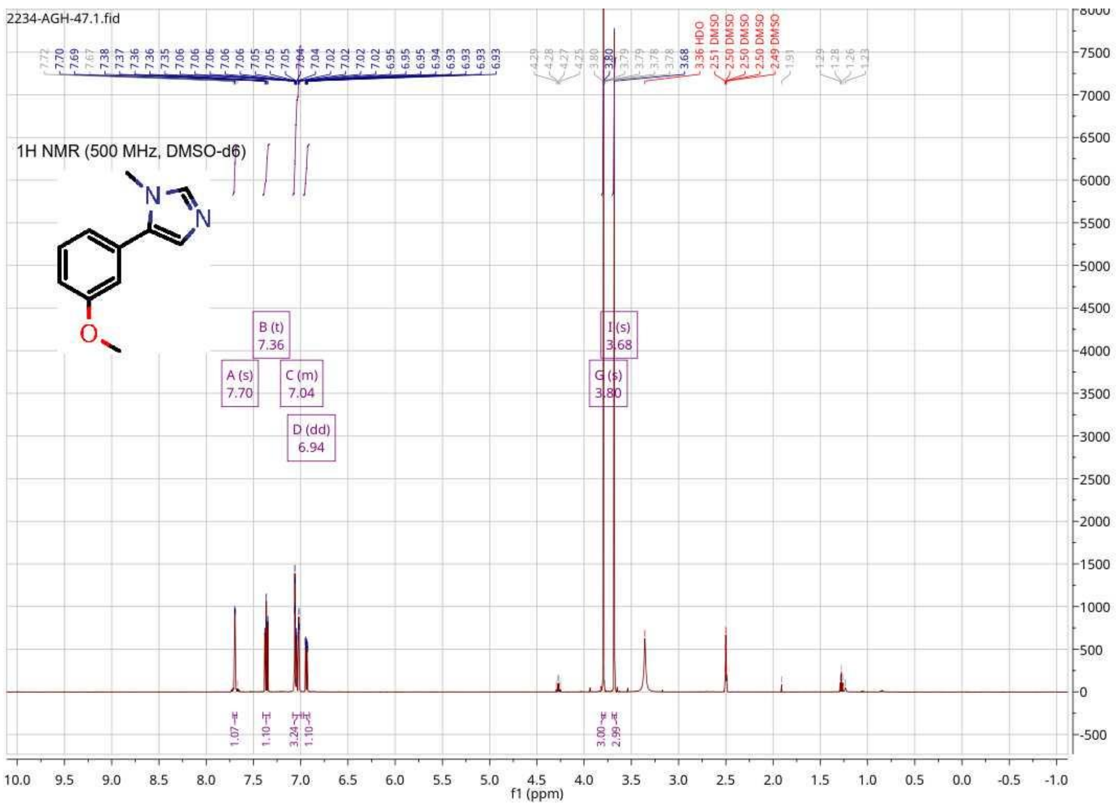
2234-AGH-47.1.fid
1H NMR (500 MHz, DMSO-d6)
A (s) 7.70
B (t) 7.36
C (m) 7.04
D (dd) 6.94
G (s) 3.80
I (s) 3.68
3.36 HDO
2.51 DMSO
2.50 DMSO
2.50 DMSO
2.50 DMSO
2.49 DMSO
1.07
1.10
3.24
1.10
3.00
2.99
f1 (ppm)

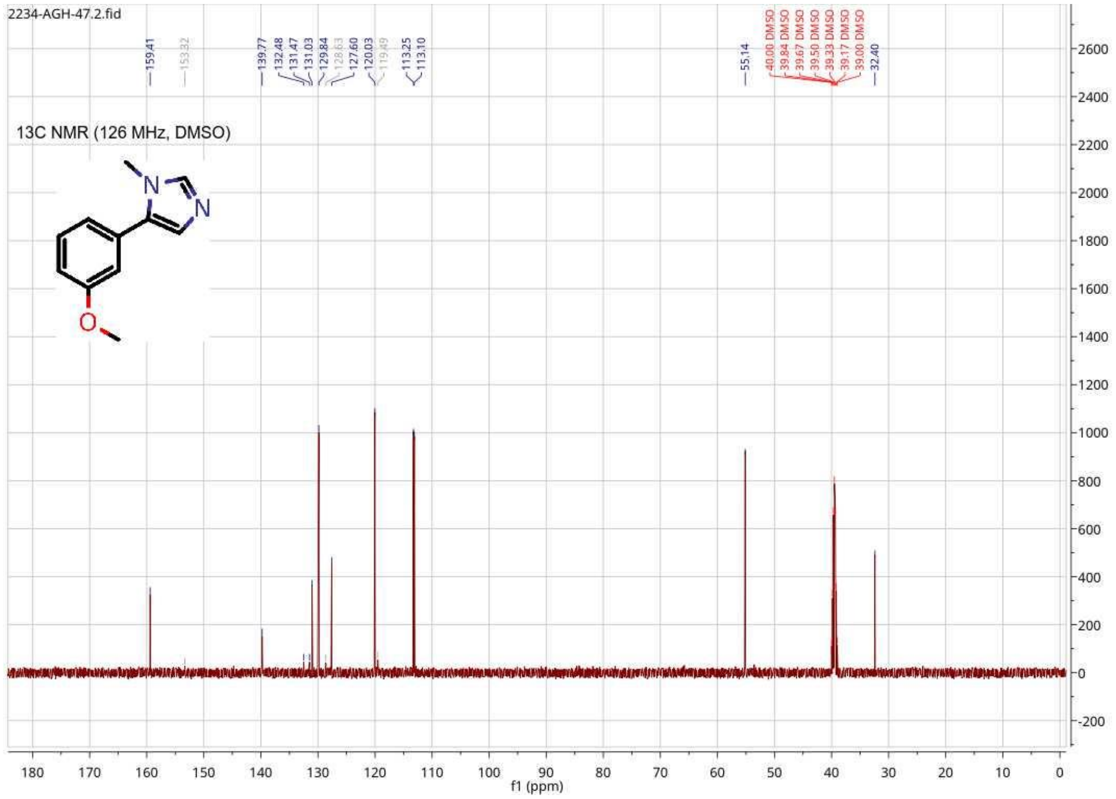
2234-AGH-47.2.fid
13C NMR (126 MHz, DMSO)
159.41
153.32
139.77
132.48
131.47
131.03
129.84
128.63
127.60
120.03
119.49
113.25
113.10
55.14
40.00 DMSO
39.84 DMSO
39.67 DMSO
39.50 DMSO
39.33 DMSO
39.17 DMSO
39.00 DMSO
32.40
f1 (ppm)

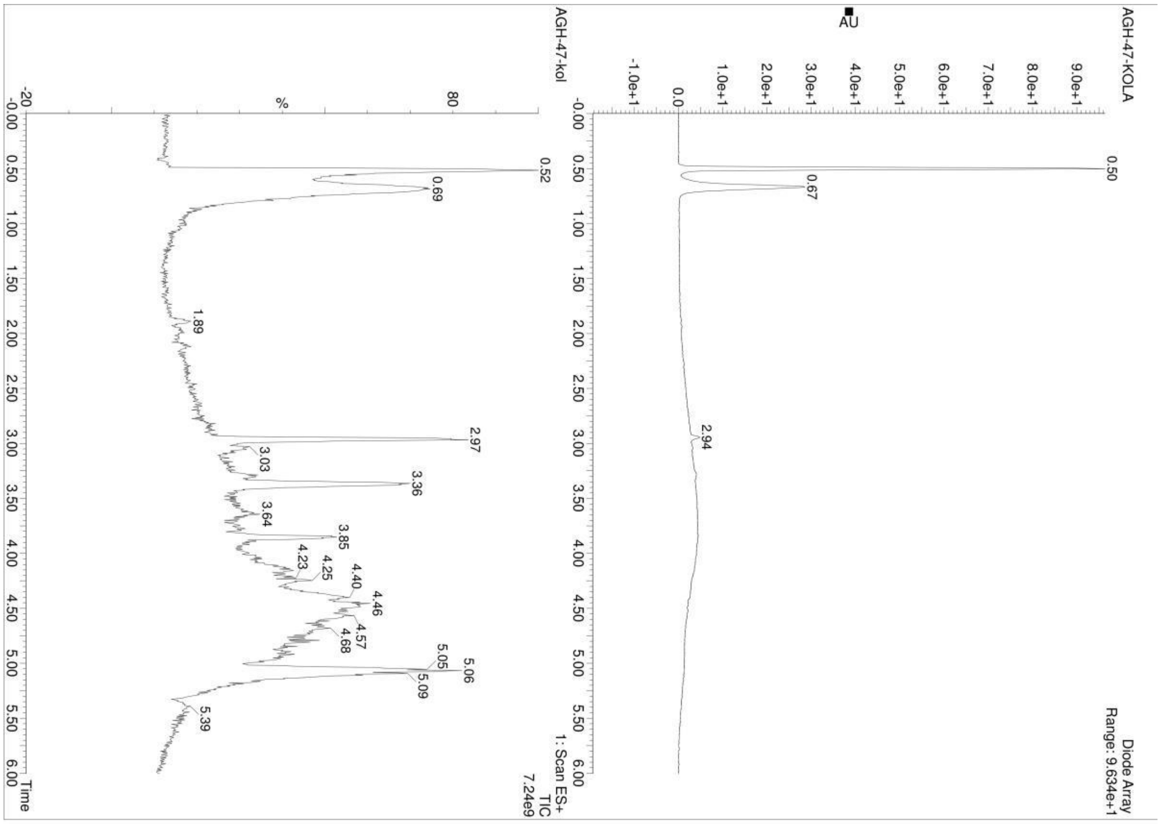

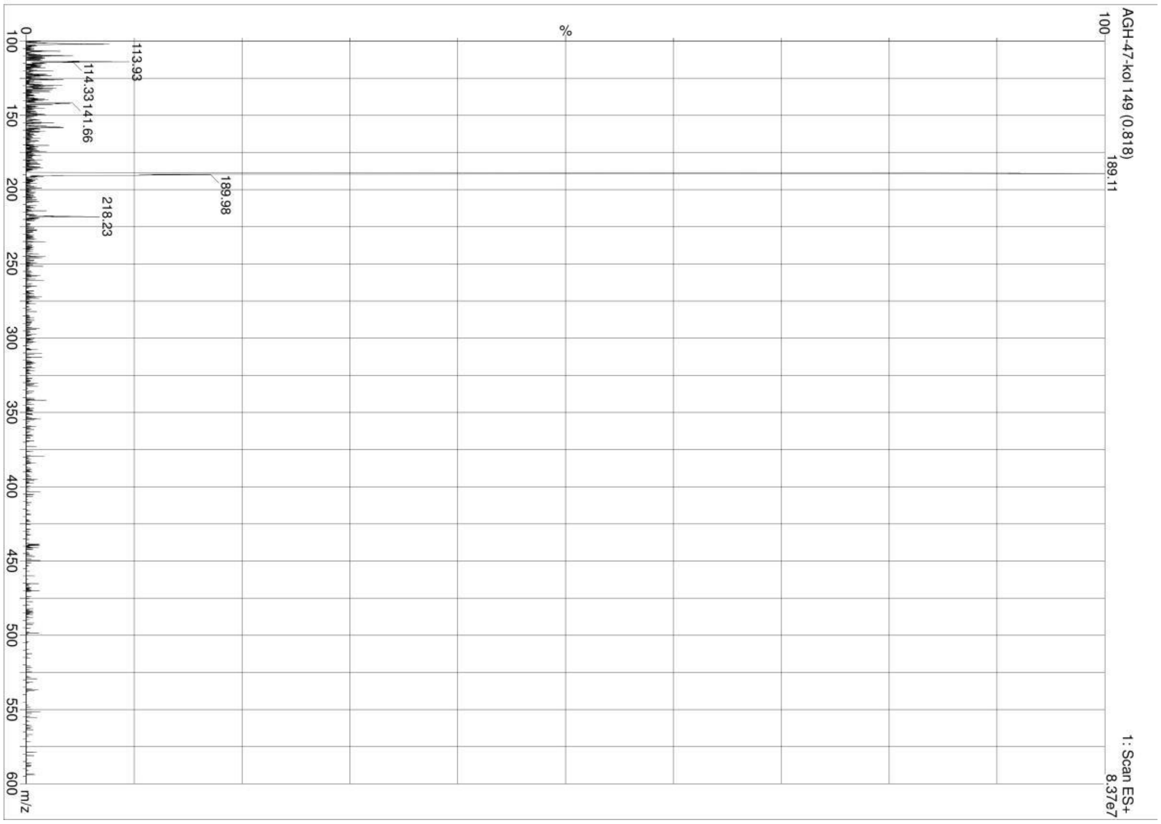

2d: 5-(2-methoxyphenyl)-1-methyl-1*H*-imidazole (AH-444)

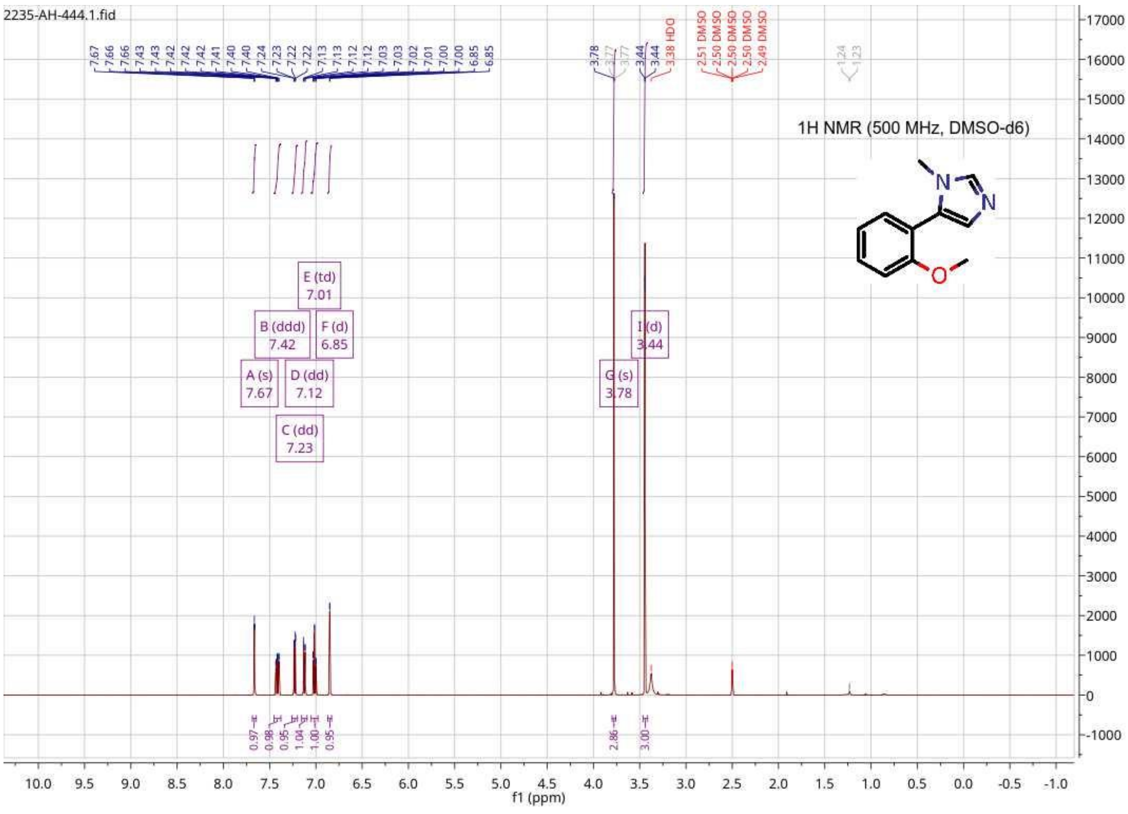
2235-AH-444.1.fid
1H NMR (500 MHz, DMSO-d6)
A (s) 7.67
B (ddd) 7.42
C (dd) 7.23
D (dd) 7.12
E (td) 7.01
F (d) 6.85
G (s) 3.78
I (d) 3.44
3.38 HDO
f1 (ppm)

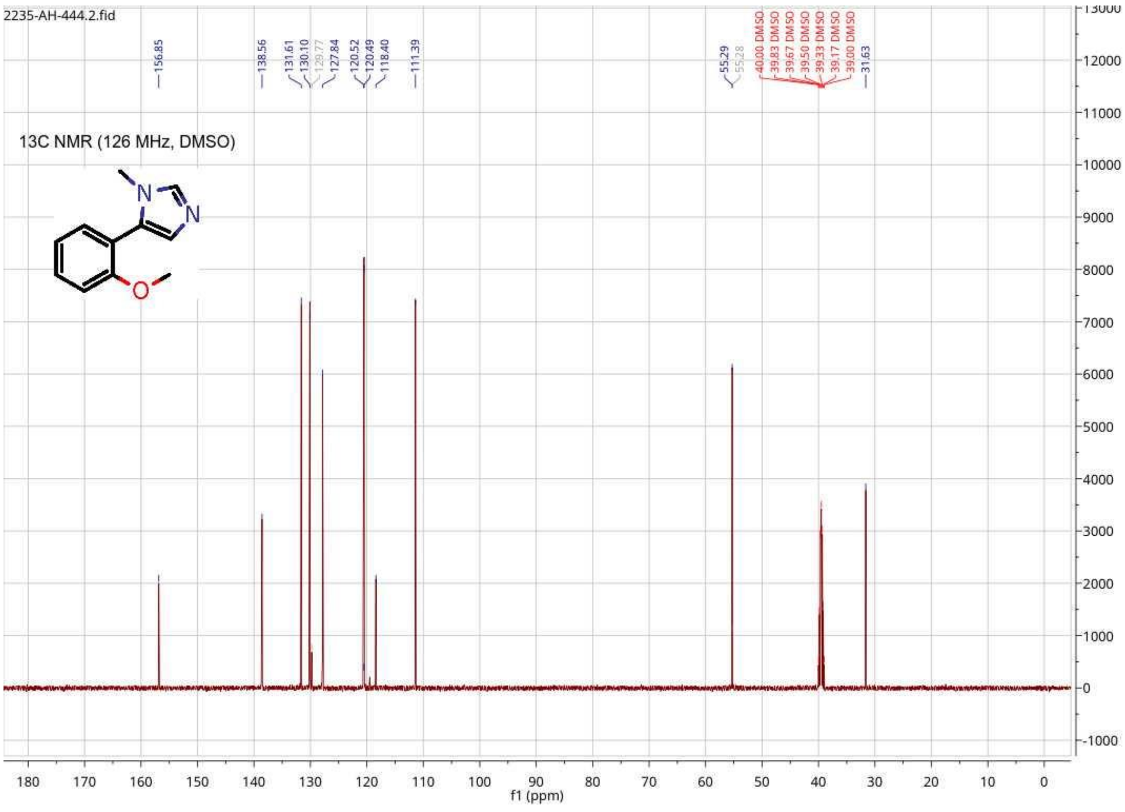
2235-AH-444.2.fid
13C NMR (126 MHz, DMSO)
156.85
138.56
131.61
130.10
127.84
120.52
120.49
118.40
111.39
55.29
31.63
f1 (ppm)

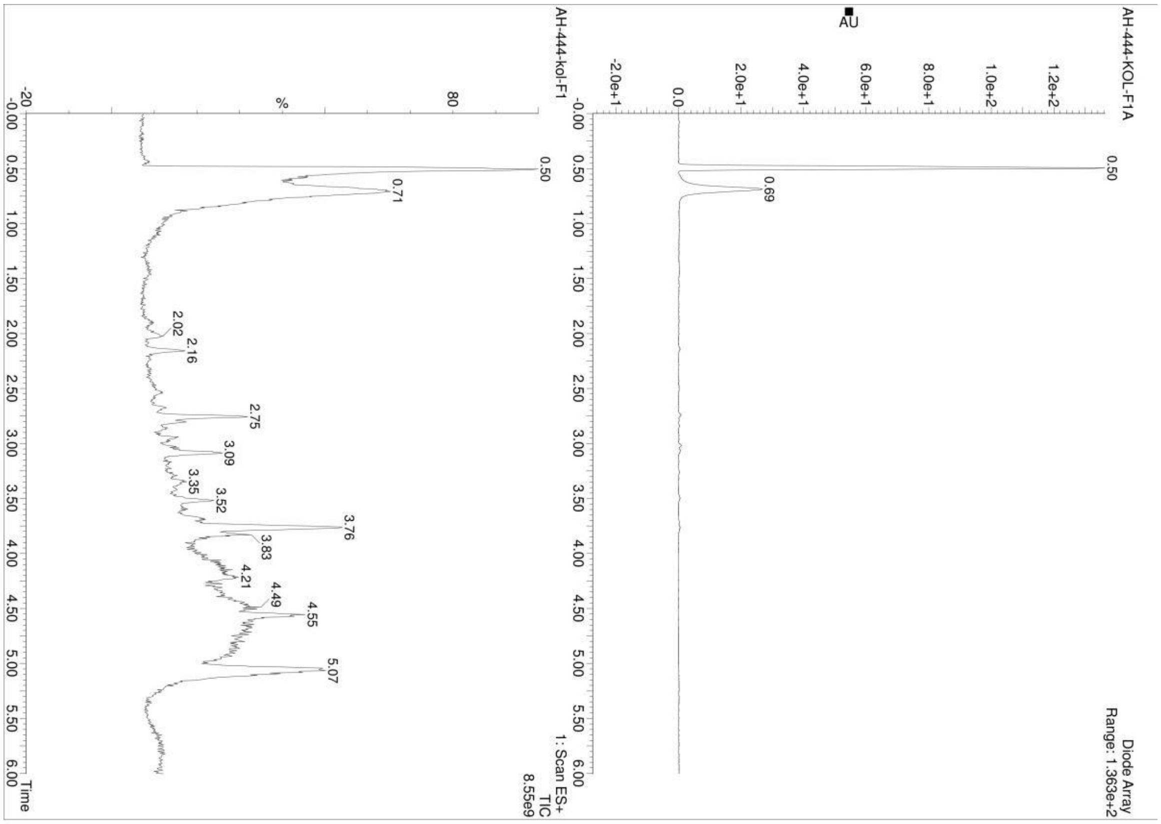

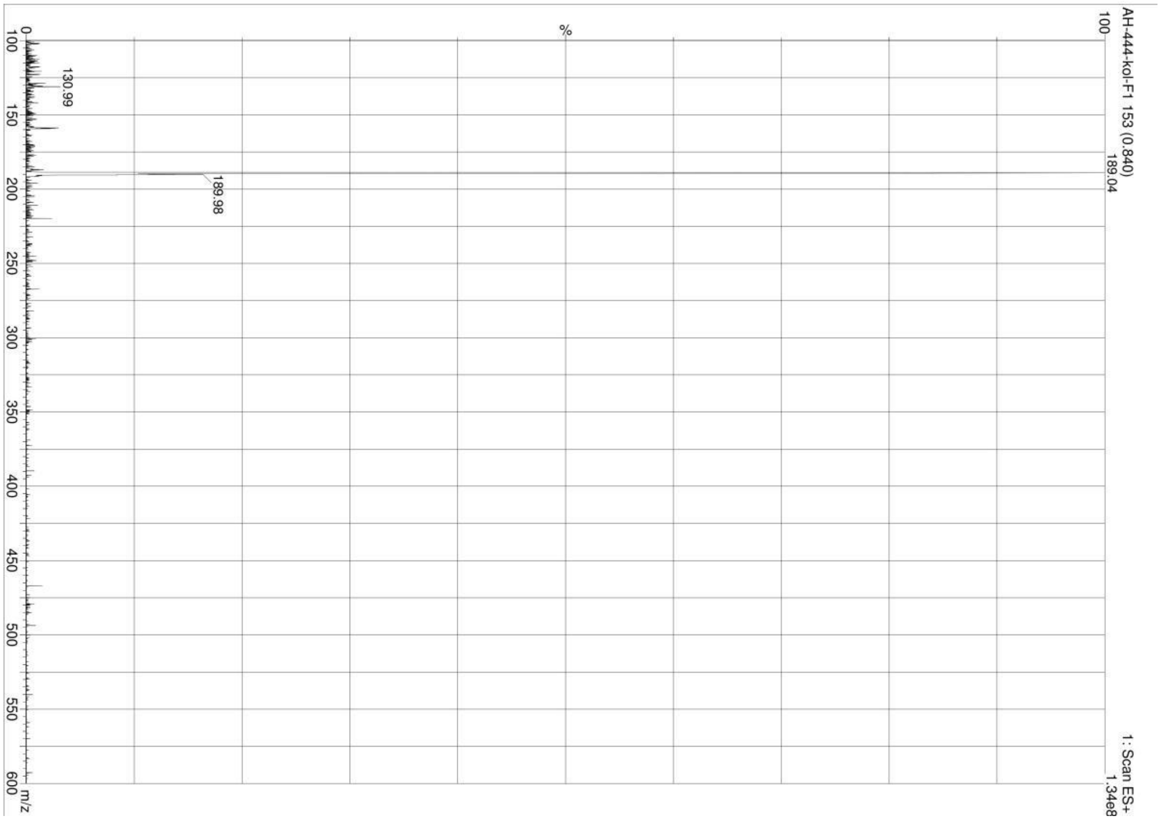

2e: 1-methyl-5-phenyl-1*H*-imidazole (AH-441)

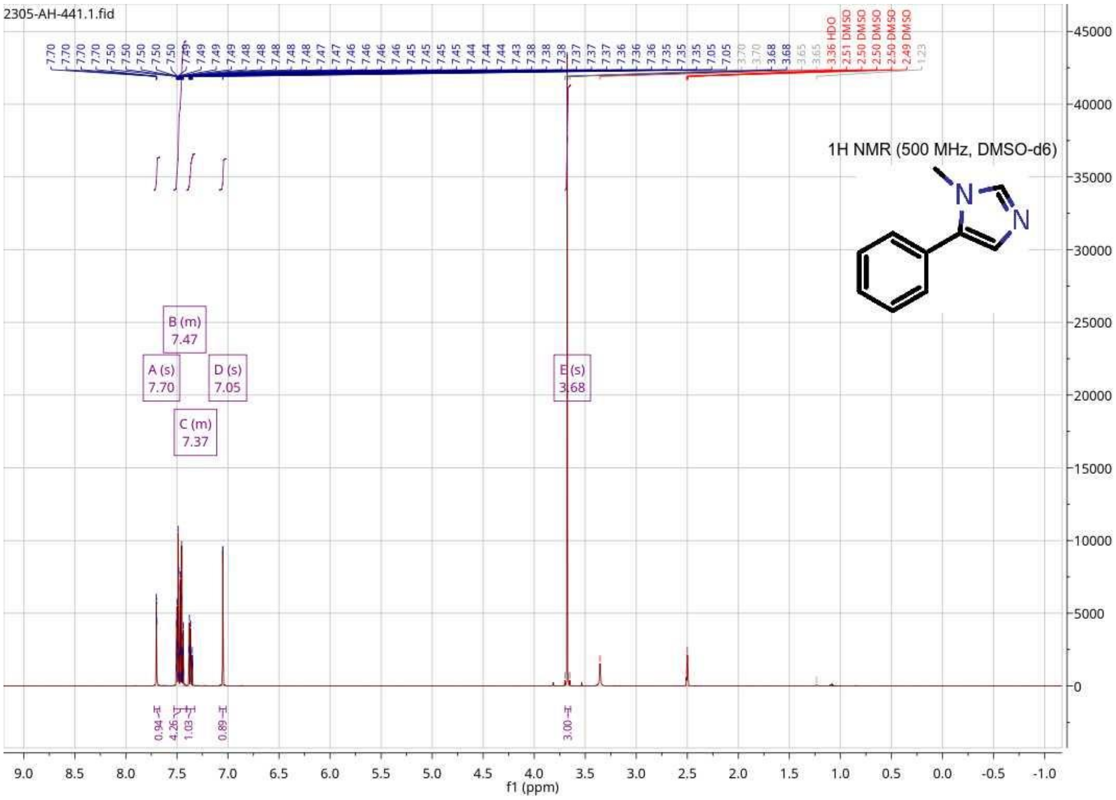
2305-AH-441.1.fid
1H NMR (500 MHz, DMSO-d6)
A (s) 7.70
B (m) 7.47
C (m) 7.37
D (s) 7.05
E (s) 3.68
3.36 HDO
2.51 DMSO
2.50 DMSO
2.50 DMSO
2.50 DMSO
2.49 DMSO
0.94
4.26
1.03
0.89
3.00
f1 (ppm)

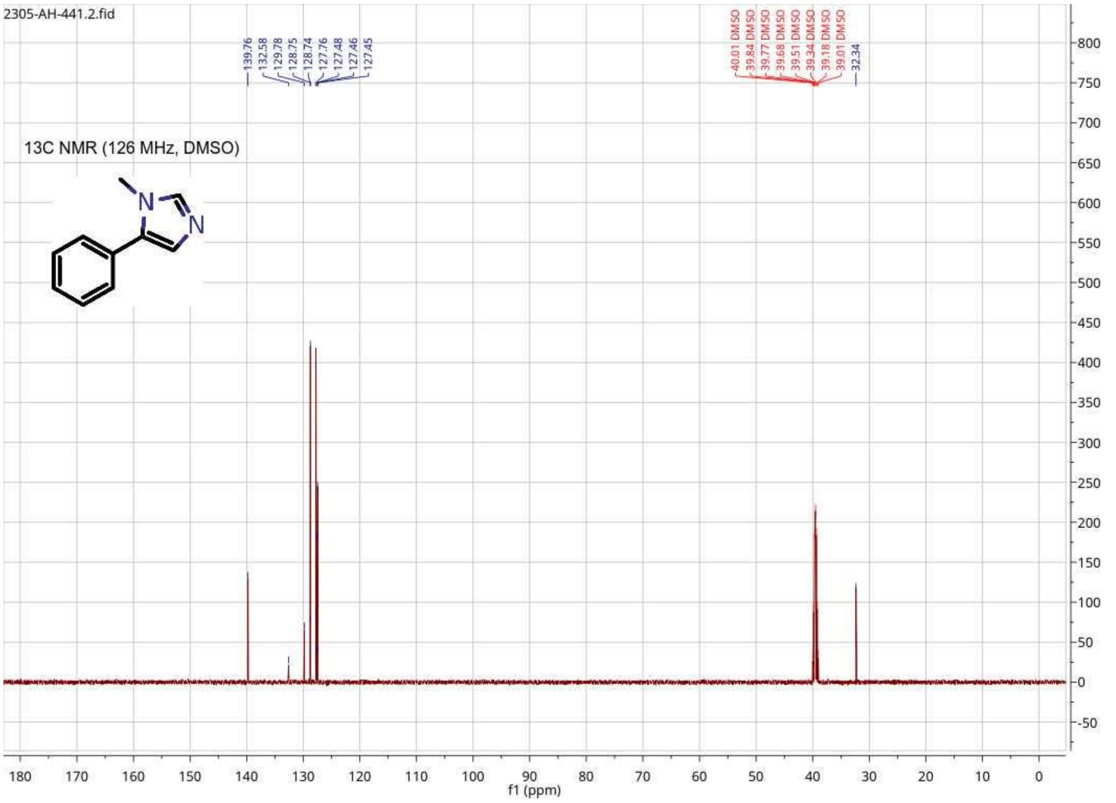
2305-AH-441.2.fid
13C NMR (126 MHz, DMSO)
139.76
132.58
129.78
128.75
128.74
127.76
127.48
127.46
127.45
40.01 DMSO
39.84 DMSO
39.77 DMSO
39.68 DMSO
39.51 DMSO
39.34 DMSO
39.18 DMSO
39.01 DMSO
32.34
f1 (ppm)

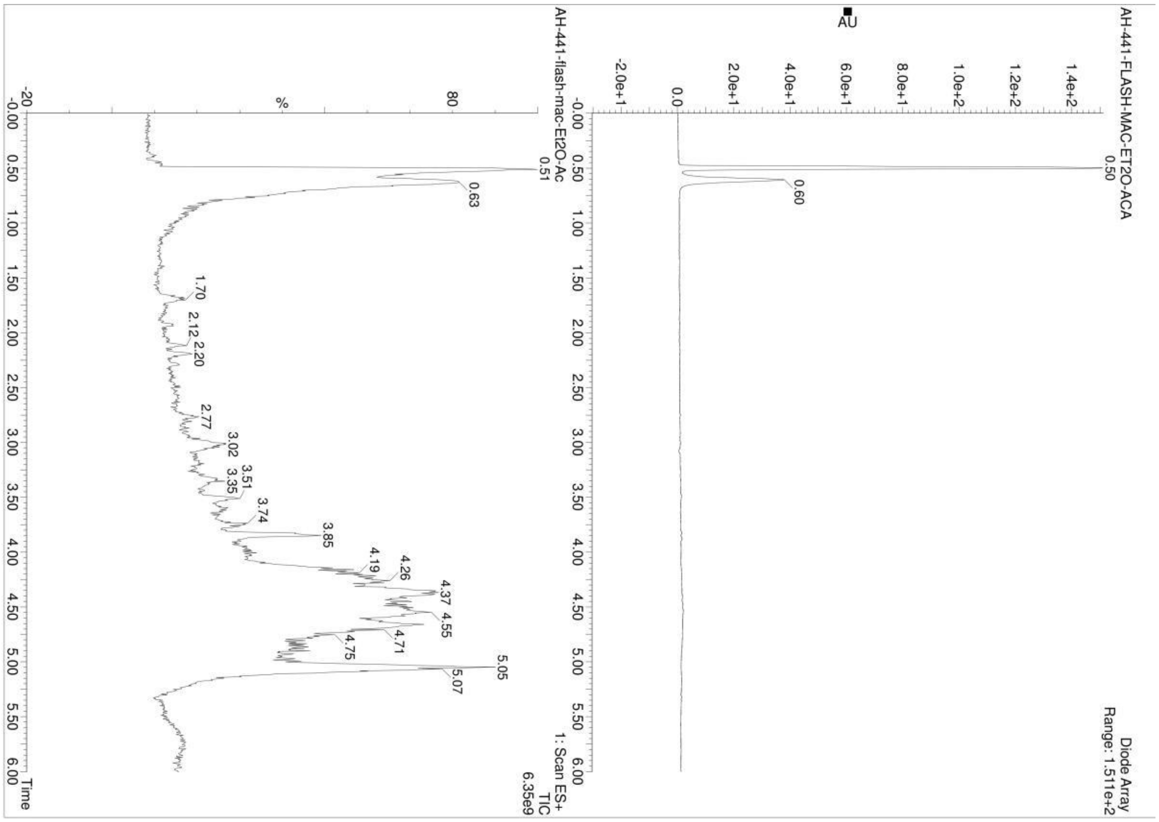
AH-441-FLASH-MAC-ET2O-ACA
Diode Array
Range: 1.511e+2
AU
0.50
0.60
AH-441-flash-mac-Et2O-Ac
1: Scan ES+
TIC
6.35e9
0.51
0.63
1.70
2.12
2.20
2.77
3.02
3.35
3.51
3.74
3.85
4.19
4.26
4.37
4.55
4.71
4.75
5.05
5.07
Time

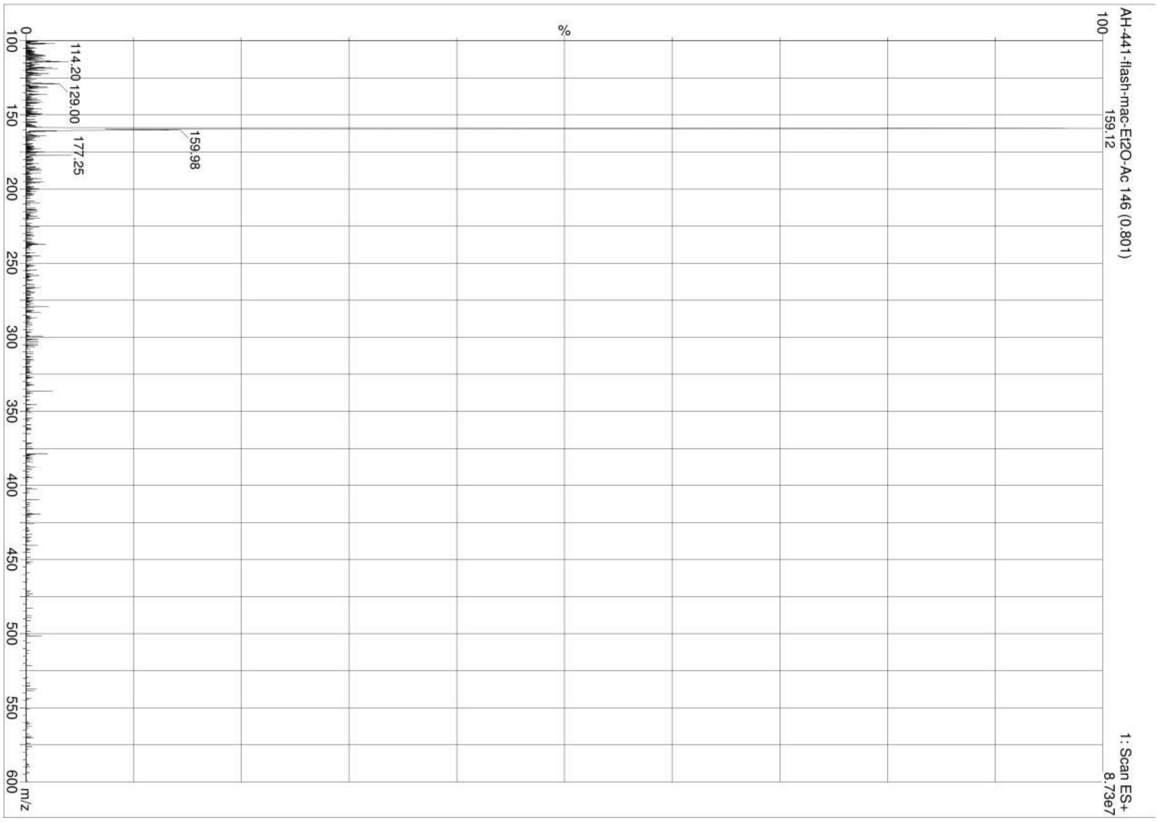
AH-441-flash-mac-Et2O-Ac 146 (0.801)
1: Scan ES+
8.73e7
159.12
114.20
129.00
159.98
177.25
m/z

2f: 5-(4-iodo-2,5-dimethoxyphenyl)-1-methyl-1*H*-imidazole (AH-438)

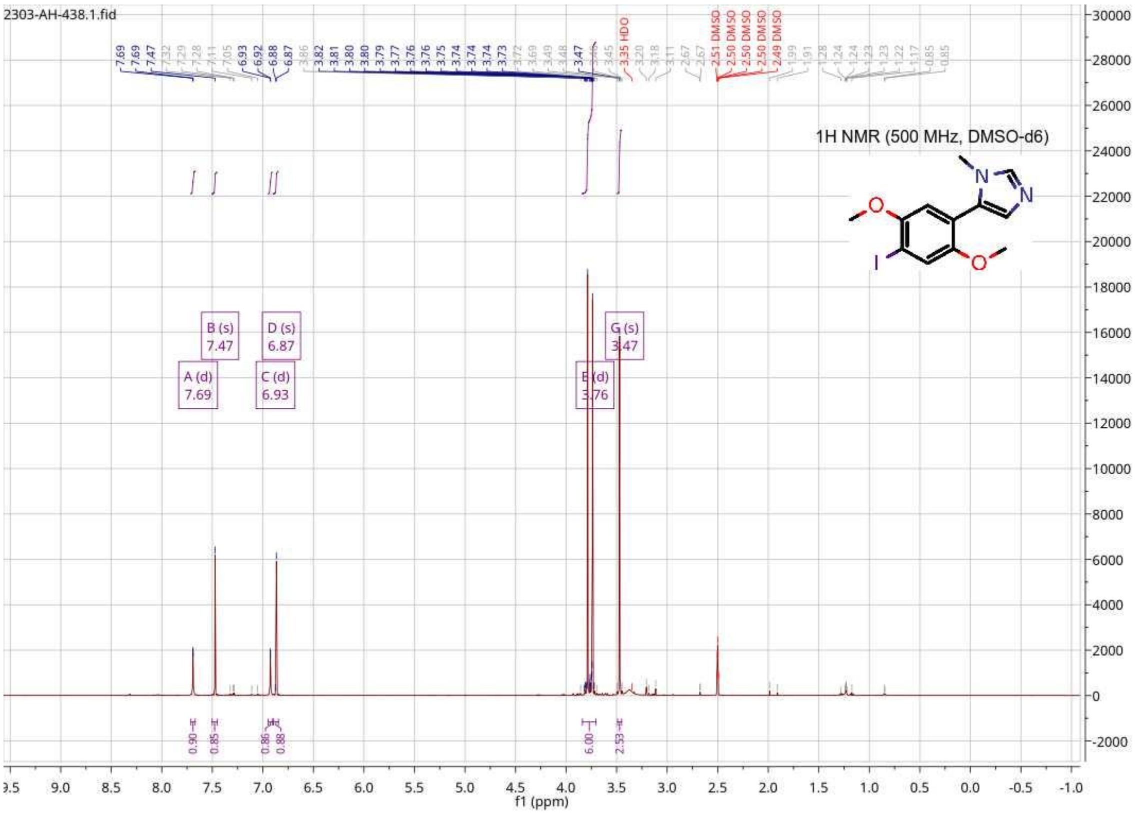

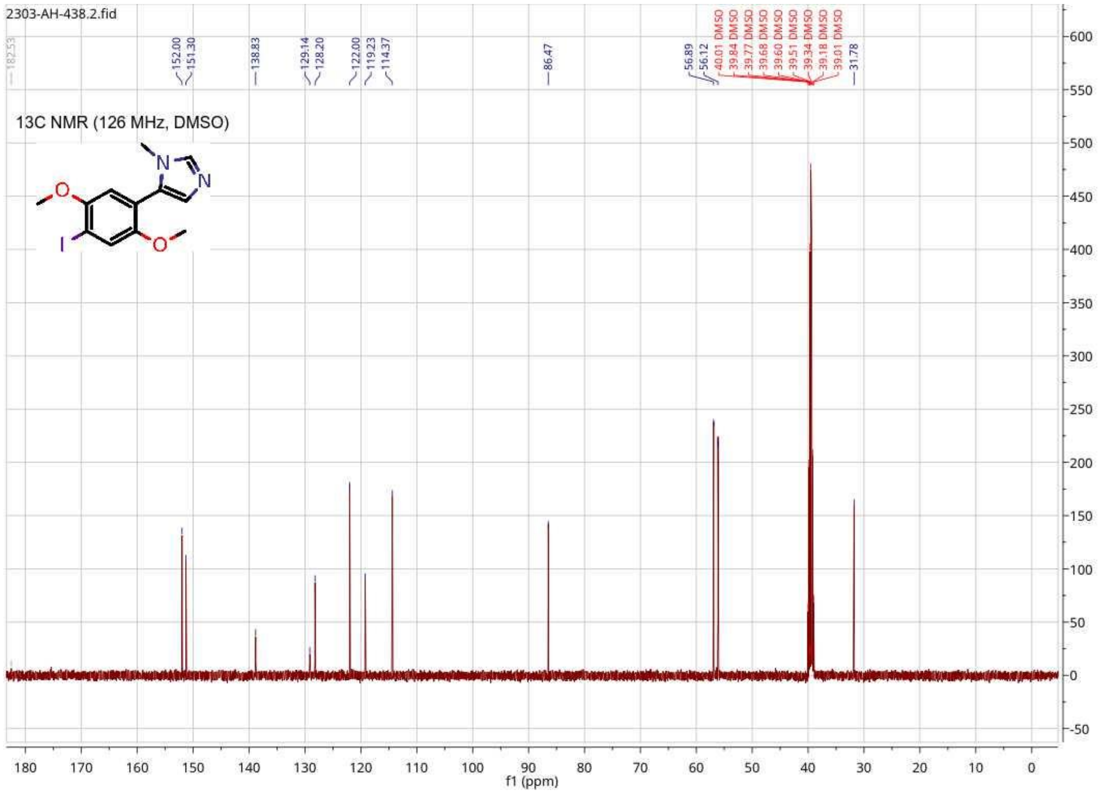

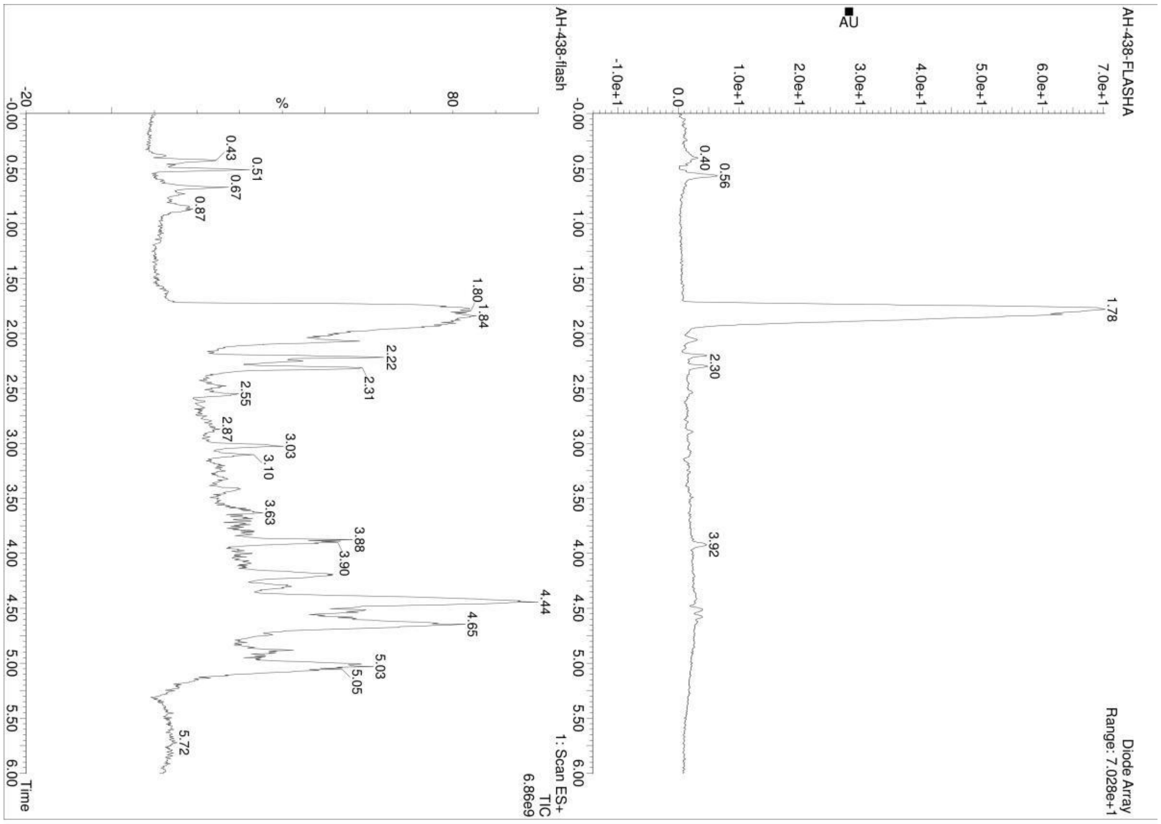
AH-438-FLASHA
Diode Array
Range: 7.028e+1
AU
AH-438-flash
1: Scan ES+
TIC
6.86e9
Time

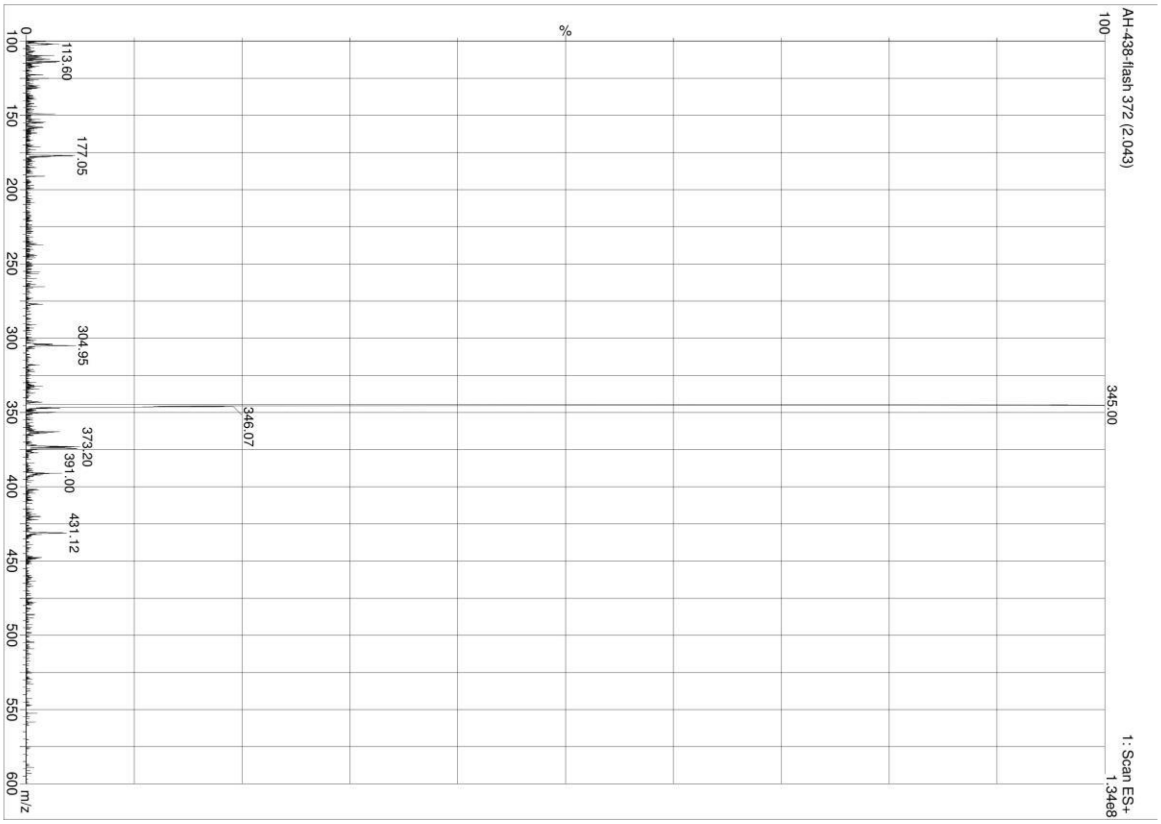
AH-438-flash 372 (2.043)
1: Scan ES+
1.34e8
345.00
346.07
m/z

3a: 1-methyl-5-(1*H*-pyrrol-2-yl)-1*H*-imidazole (AH-426)

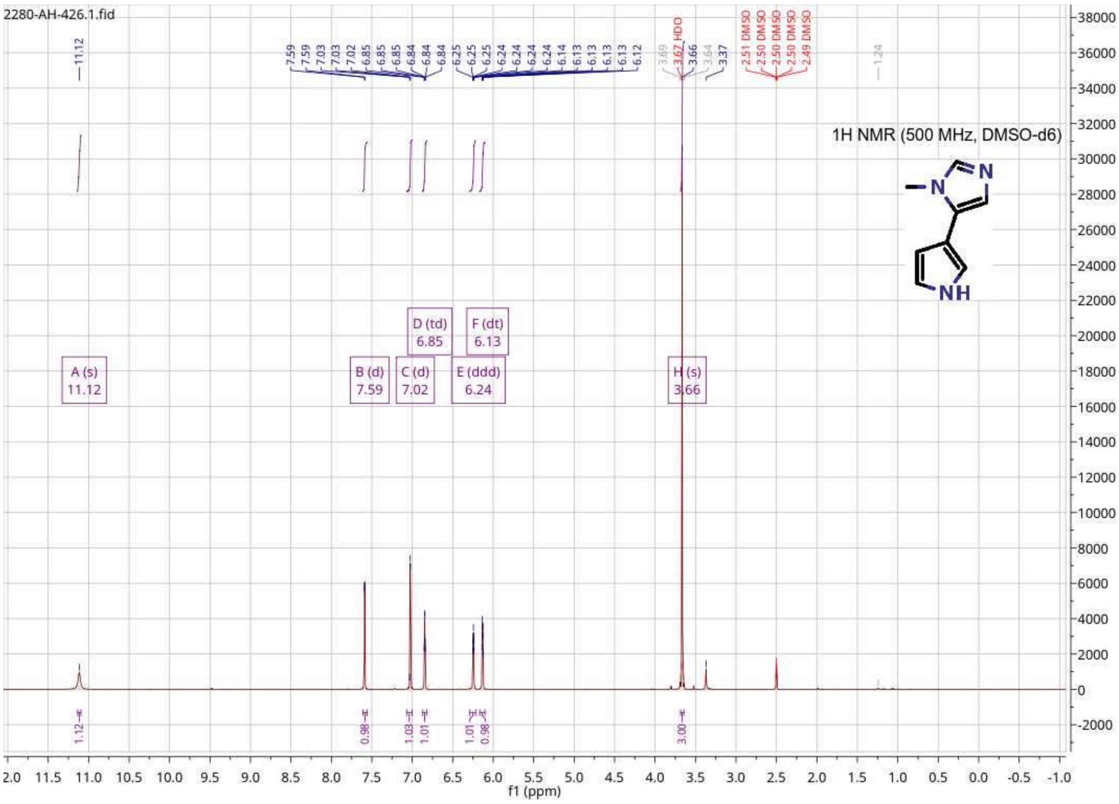

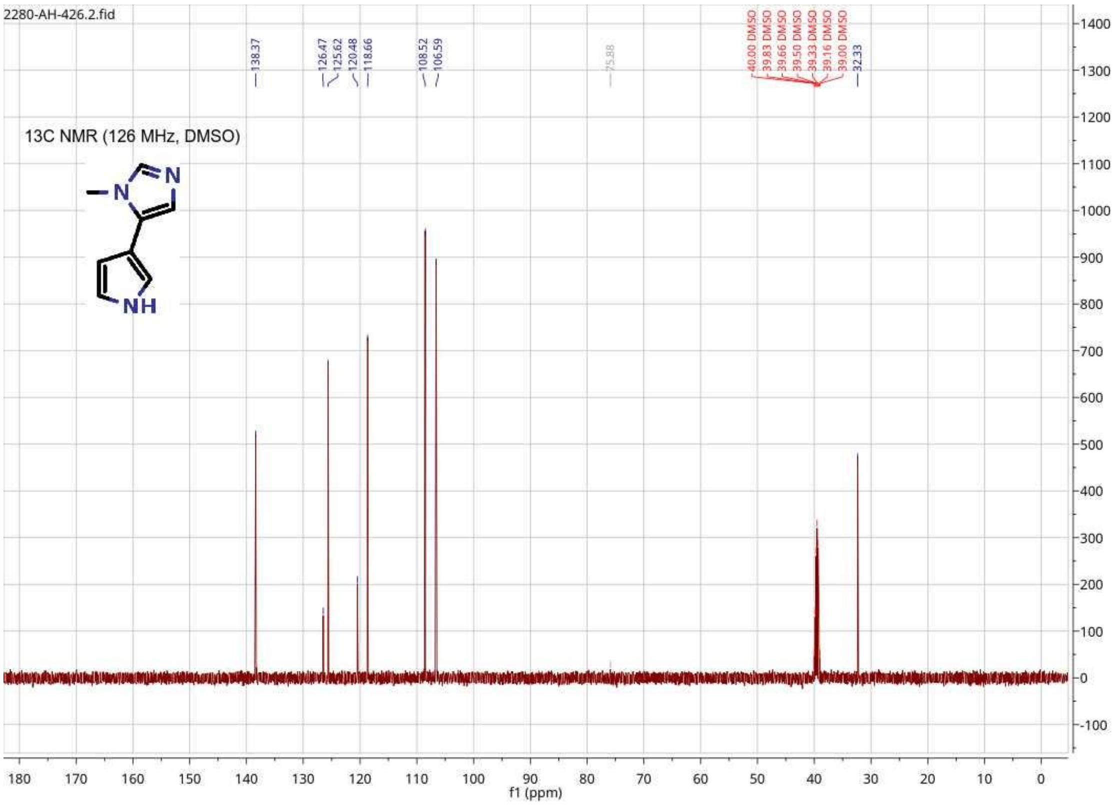

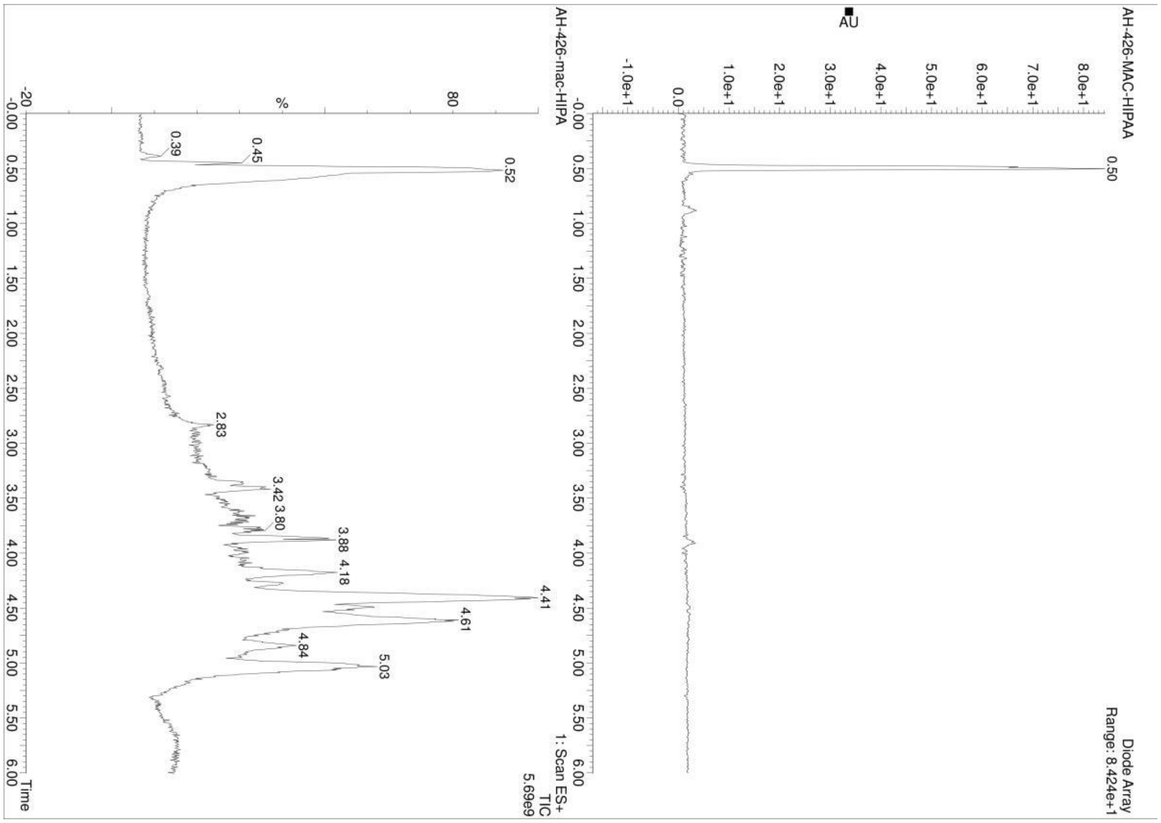

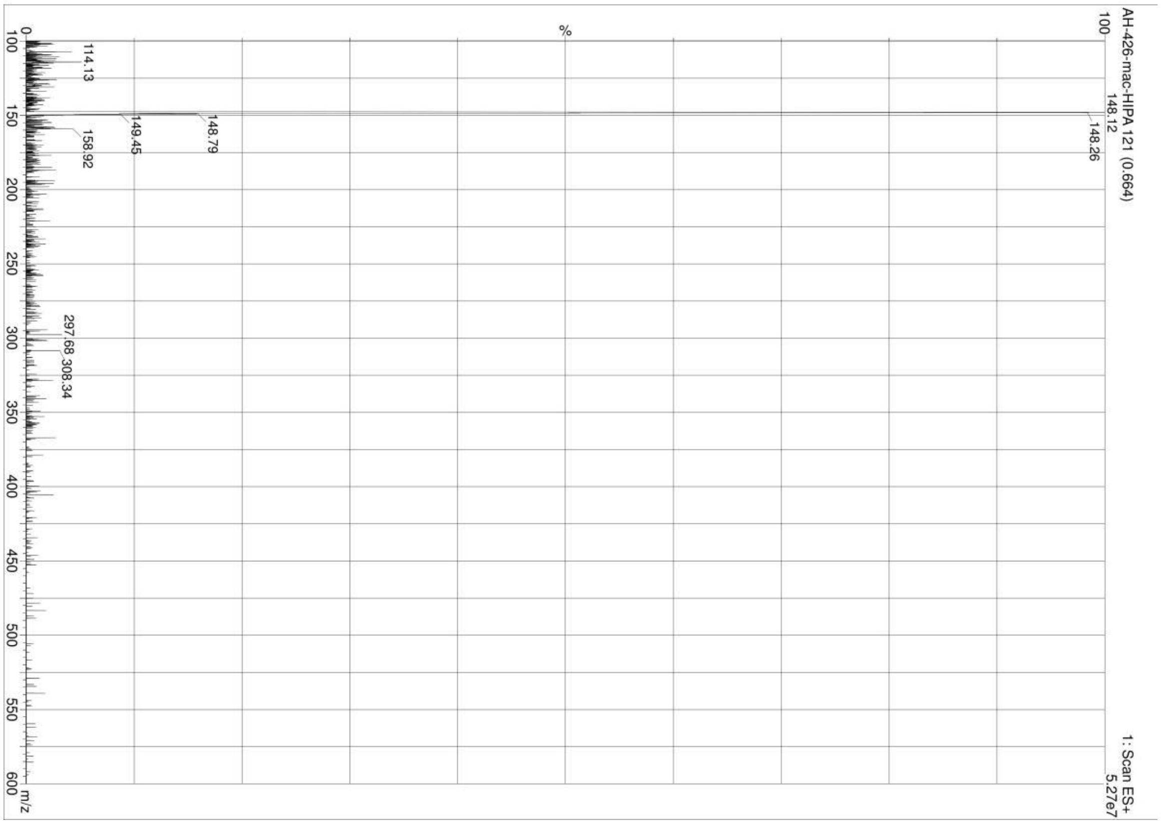

3b: 2-(1-methyl-1*H*-imidazol-4-yl)-1*H*-indole (AGH-51)

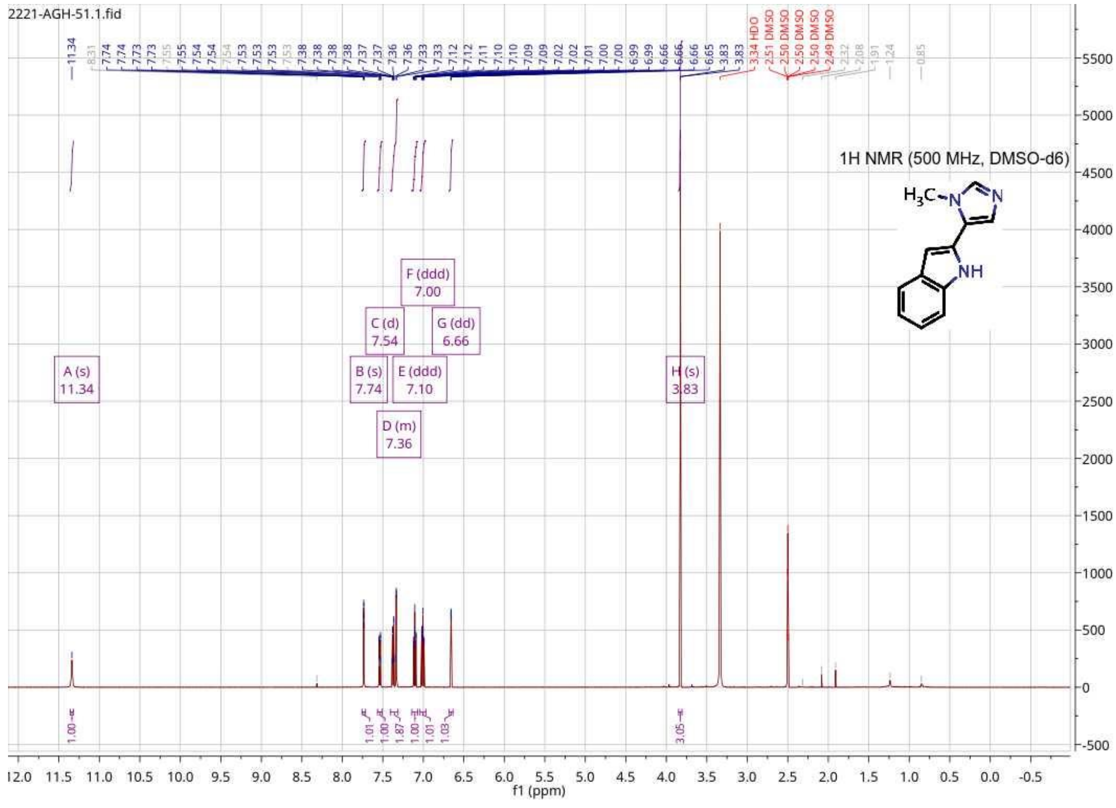

2221-AGH-51.1.fid
1H NMR (500 MHz, DMSO-d6)
A (s) 11.34
B (s) 7.74
C (d) 7.54
D (m) 7.36
E (ddd) 7.10
F (ddd) 7.00
G (dd) 6.66
H (s) 3.83
f1 (ppm)

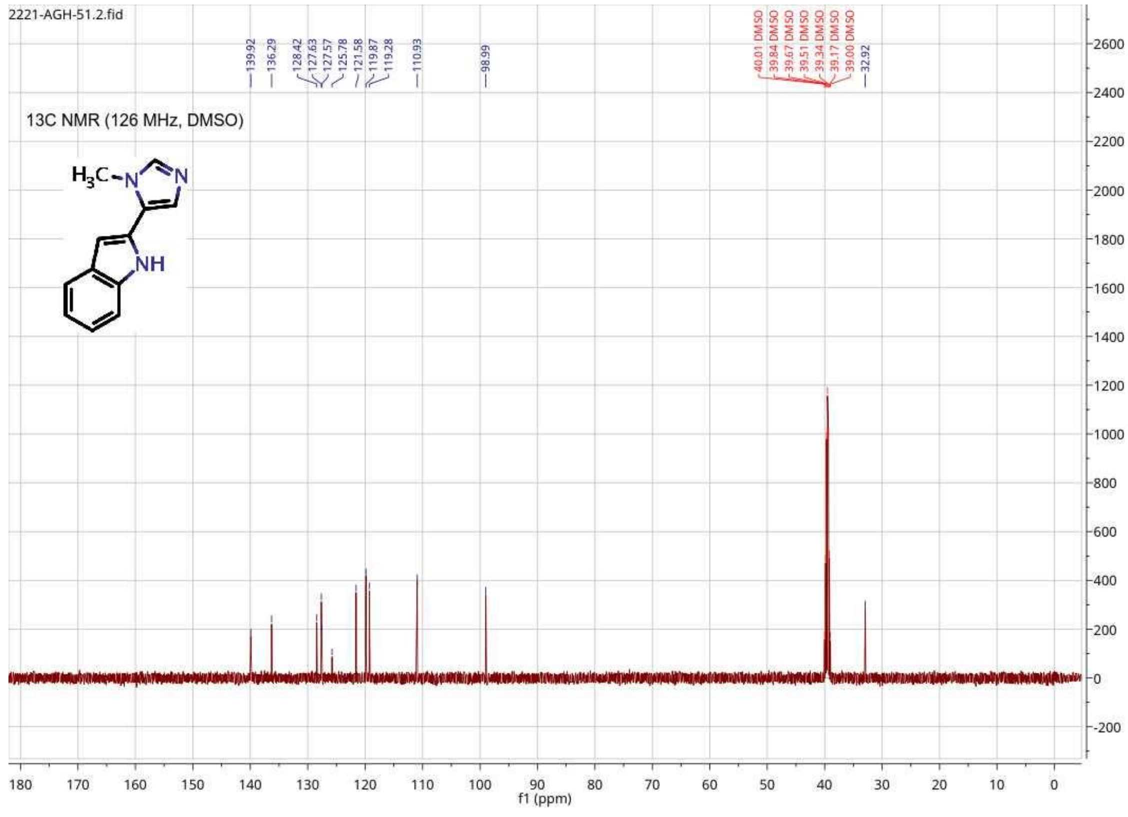

2221-AGH-51.2.fid
13C NMR (126 MHz, DMSO)
139.92
136.29
128.42
127.63
127.57
125.78
121.58
119.87
119.28
110.93
98.99
32.92
f1 (ppm)

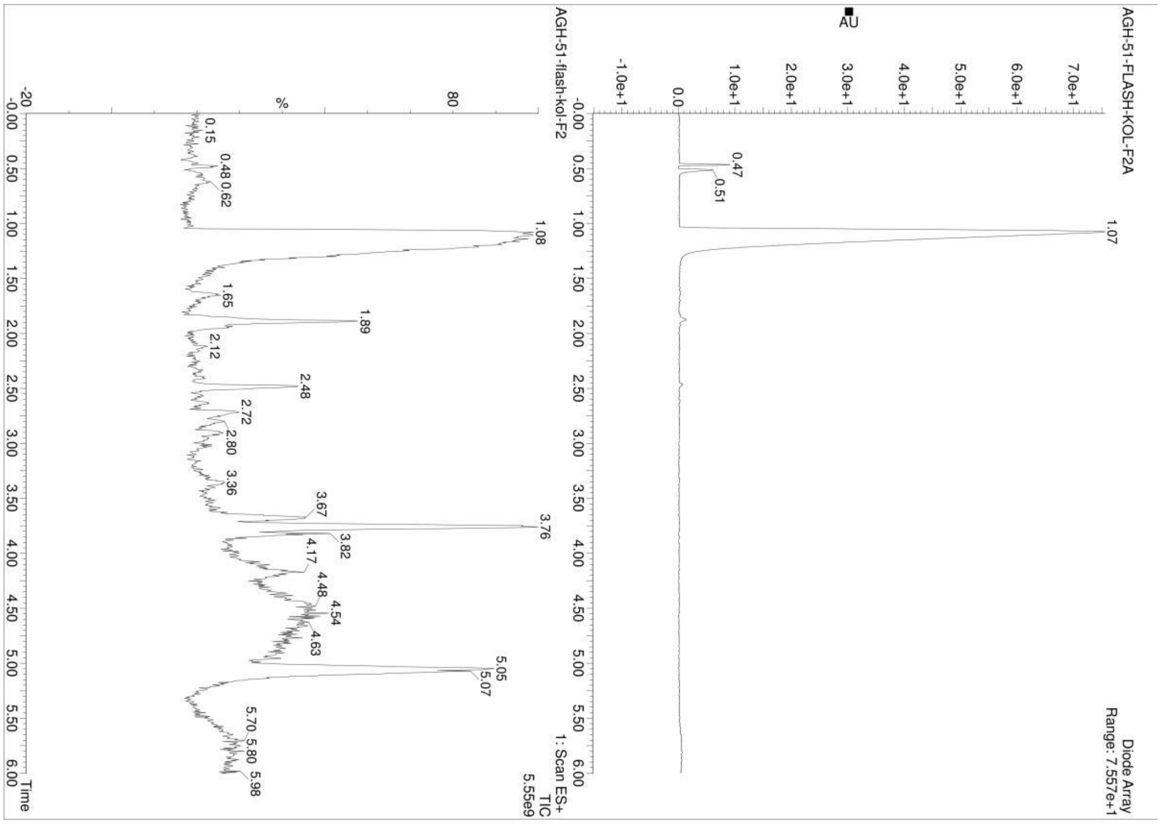
AGH-51-FLASH-KOL-F2A
Diode Array
Range: 7.557e+1
AU
1.07
0.47
0.51
AGH-51-flash-kol-F2
1: Scan ES+
TIC
5.55e9
1.08
1.89
2.48
3.76
5.05
5.07
Time

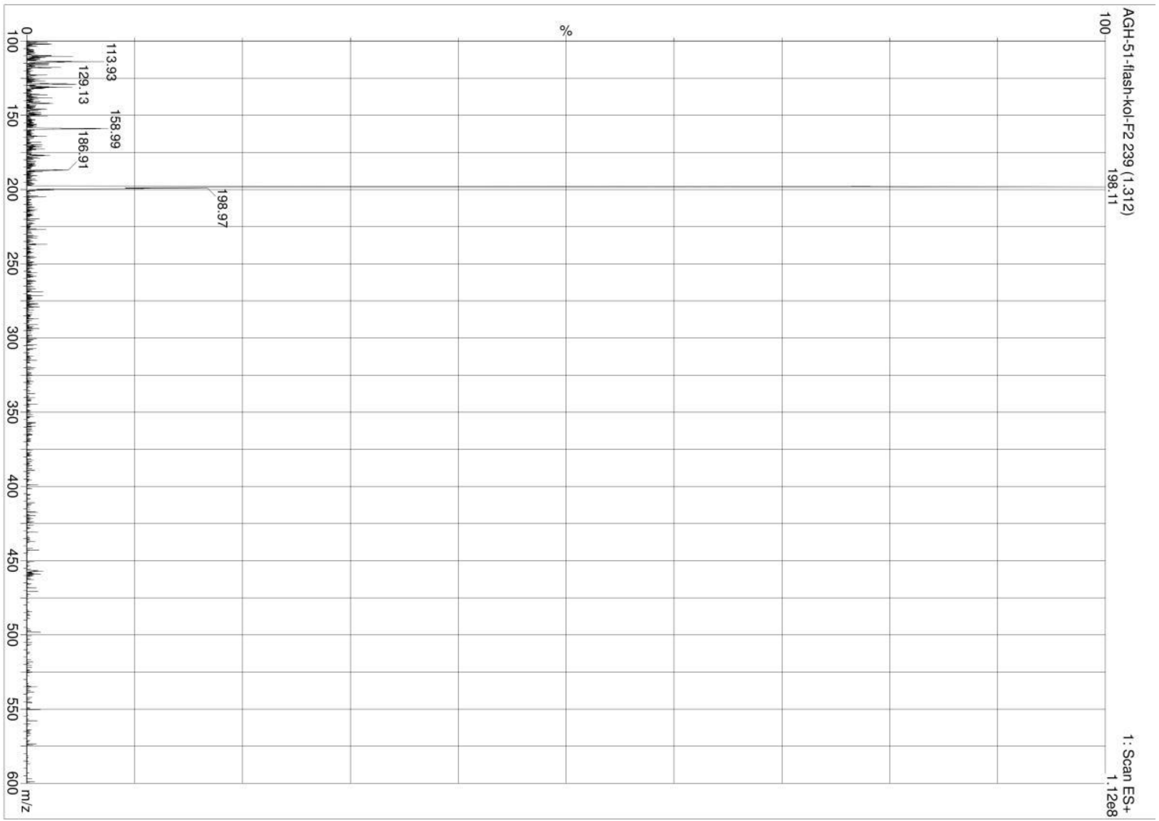
AGH-51-flash-kol-F2 239 (1.312)
1: Scan ES+
1.12e8
198.11
198.97
113.93
129.13
158.99
186.91
m/z

3c: 5-(1-ethyl-1*H*-imidazol-5-yl)-1*H*-indole (AGH-98)

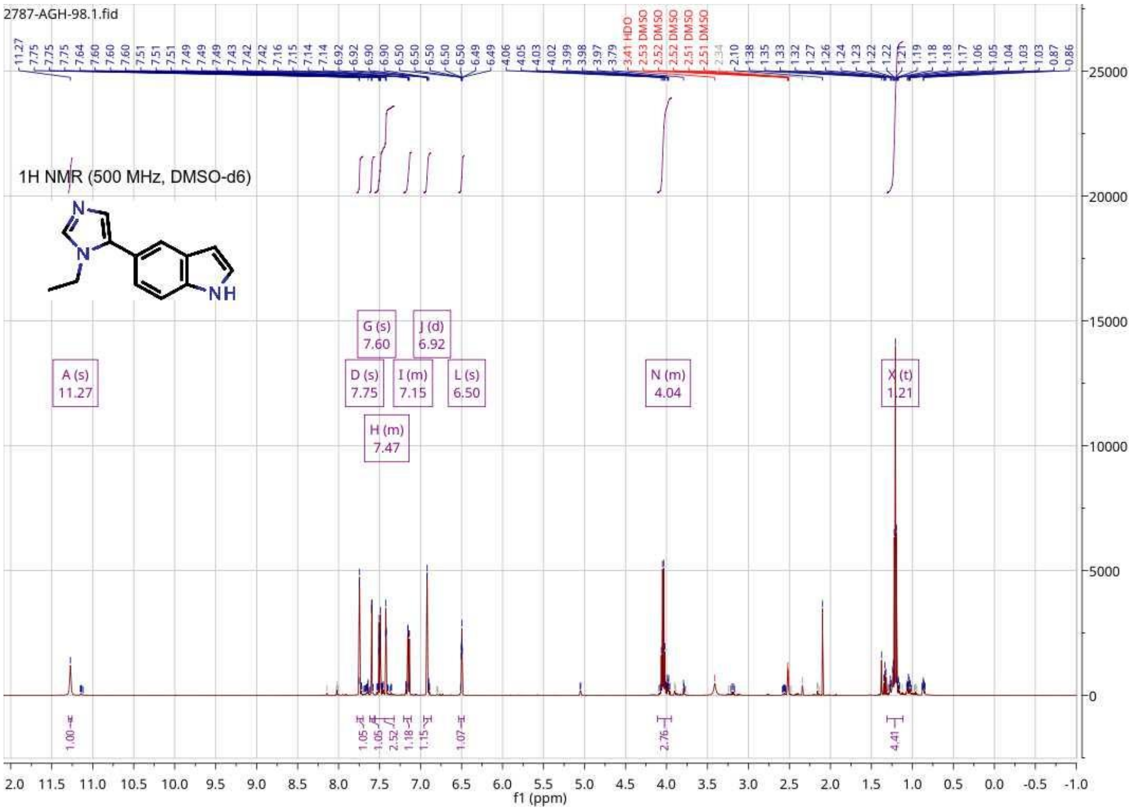

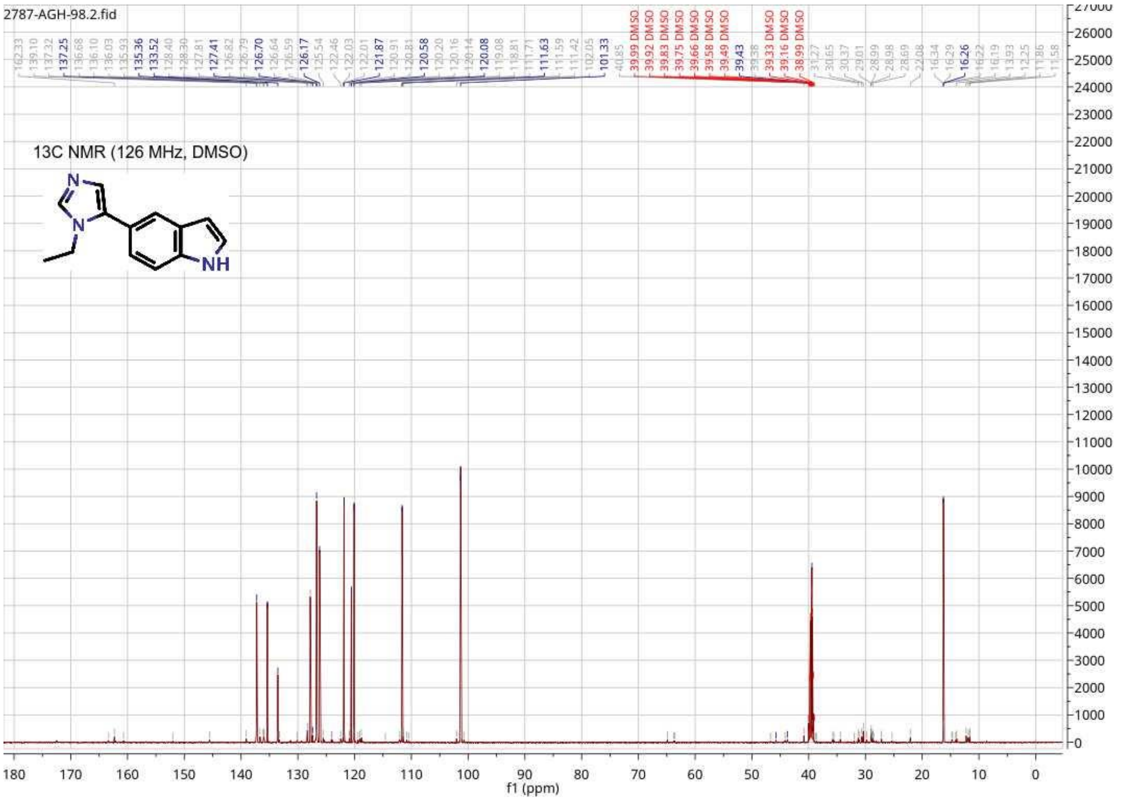

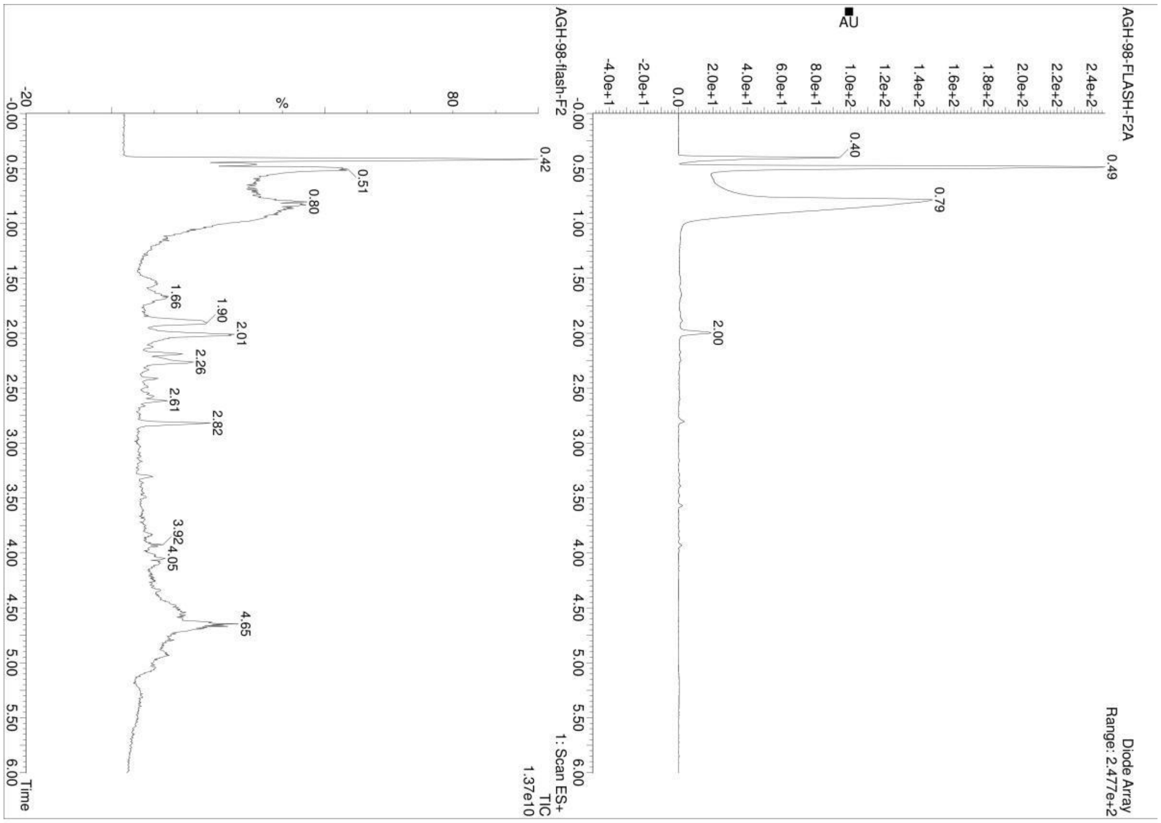
AGH-98-FLASH-F2A
Diode Array
Range: 2.477e+2
AU
0.40
0.49
0.79
2.00
AGH-98-flash-F2
1: Scan ES+
TIC
1.37e10
0.42
0.51
0.80
1.66
1.90
2.01
2.26
2.61
2.82
3.92
4.05
4.65
%
Time

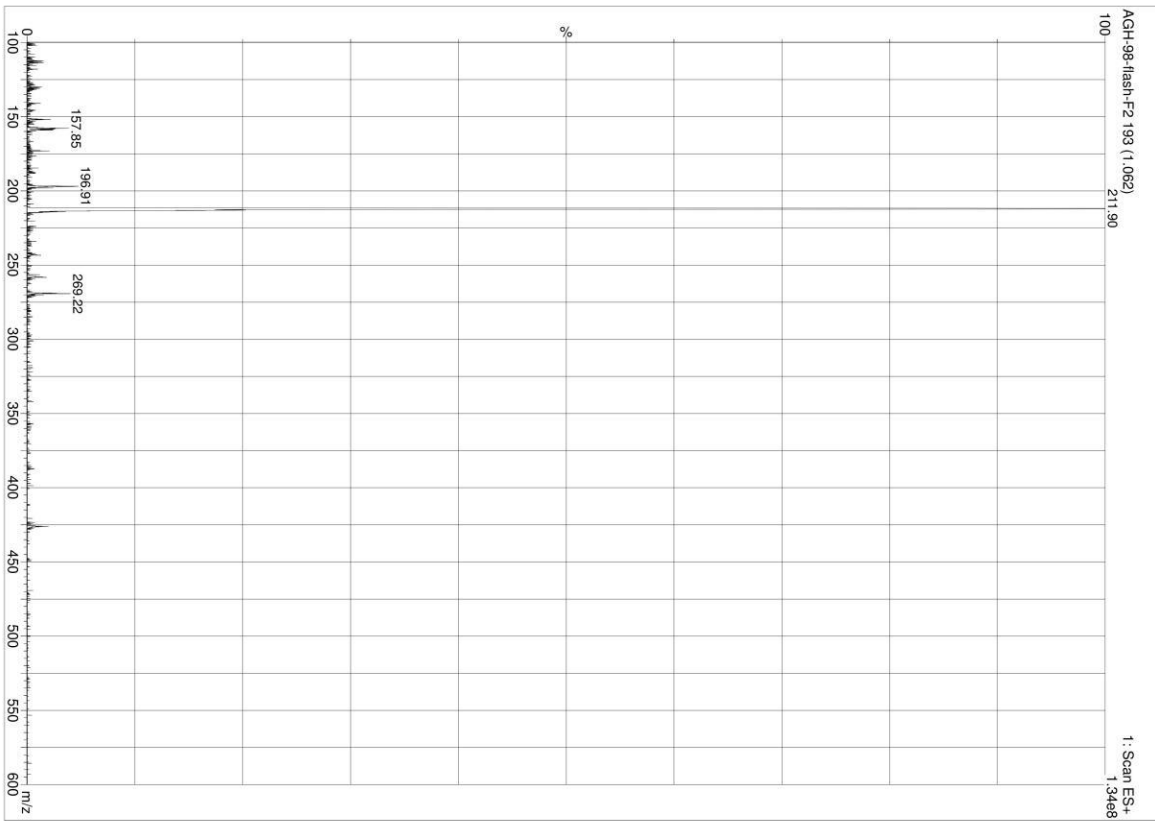
AGH-98-flash-F2 193 (1.062)
1: Scan ES+
1.34e8
%
211.90
157.85
196.91
269.22
m/z

3d: 6-(1-ethyl-1*H*-imidazol-5-yl)-1*H*-indole (AGH-99)

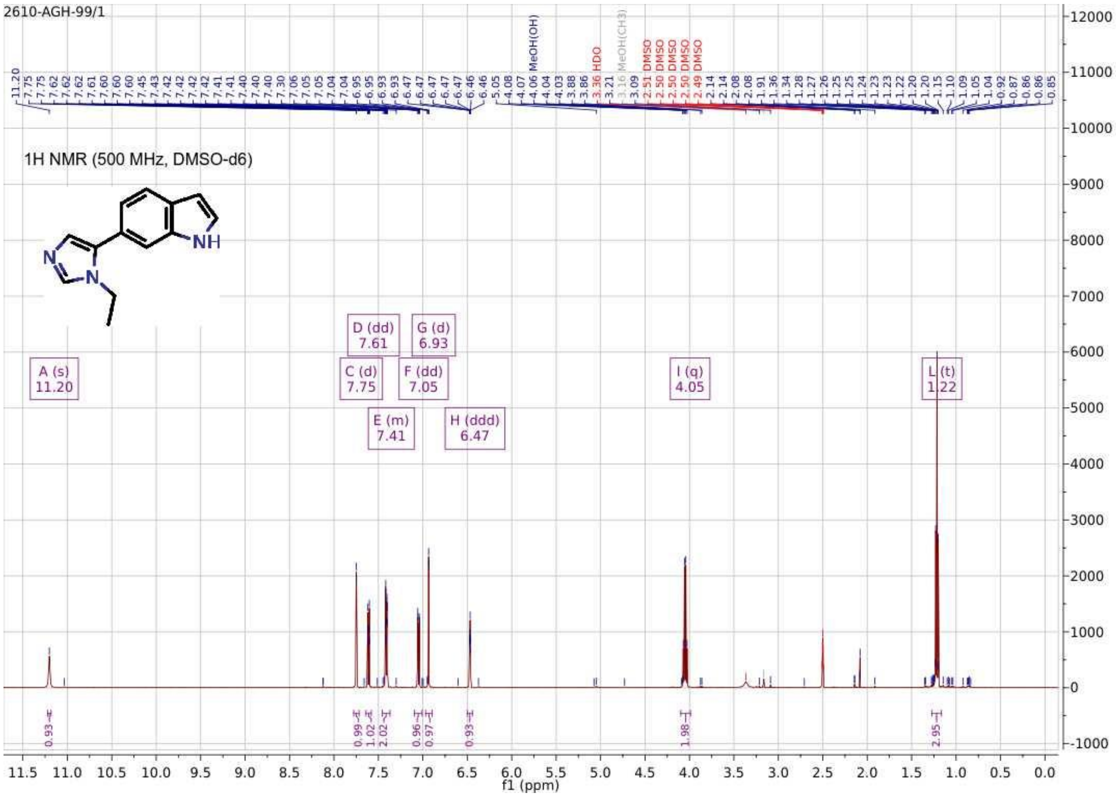

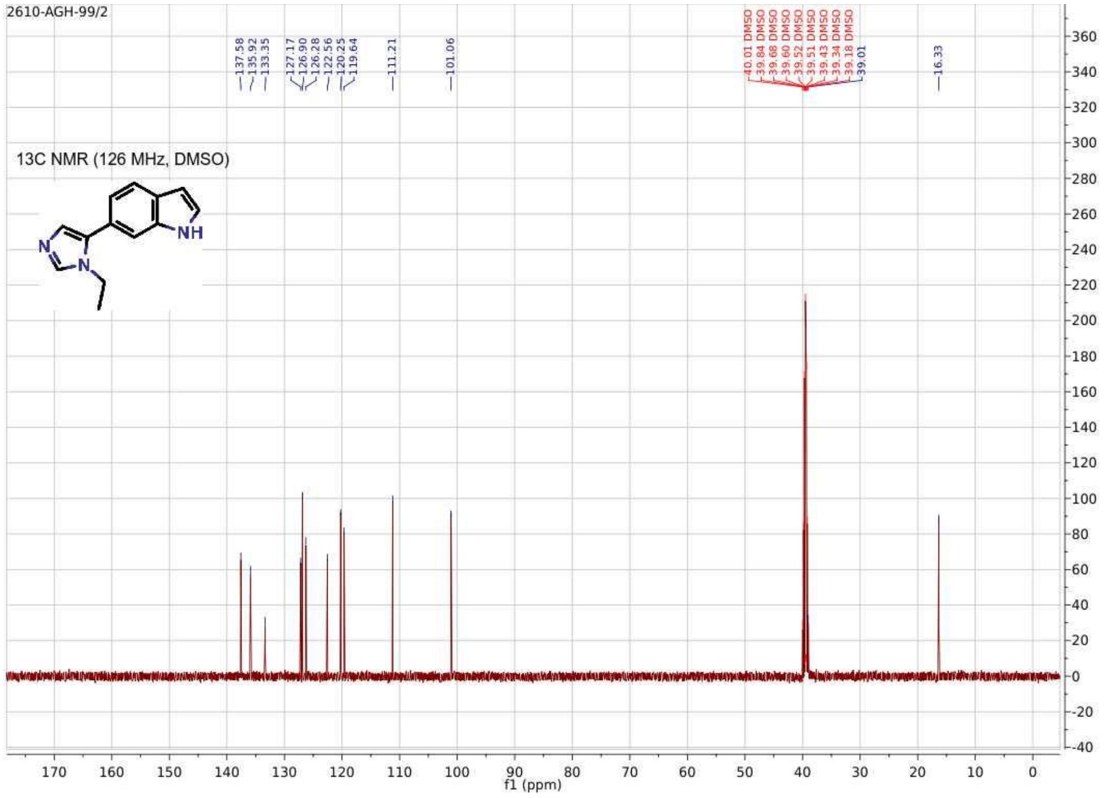

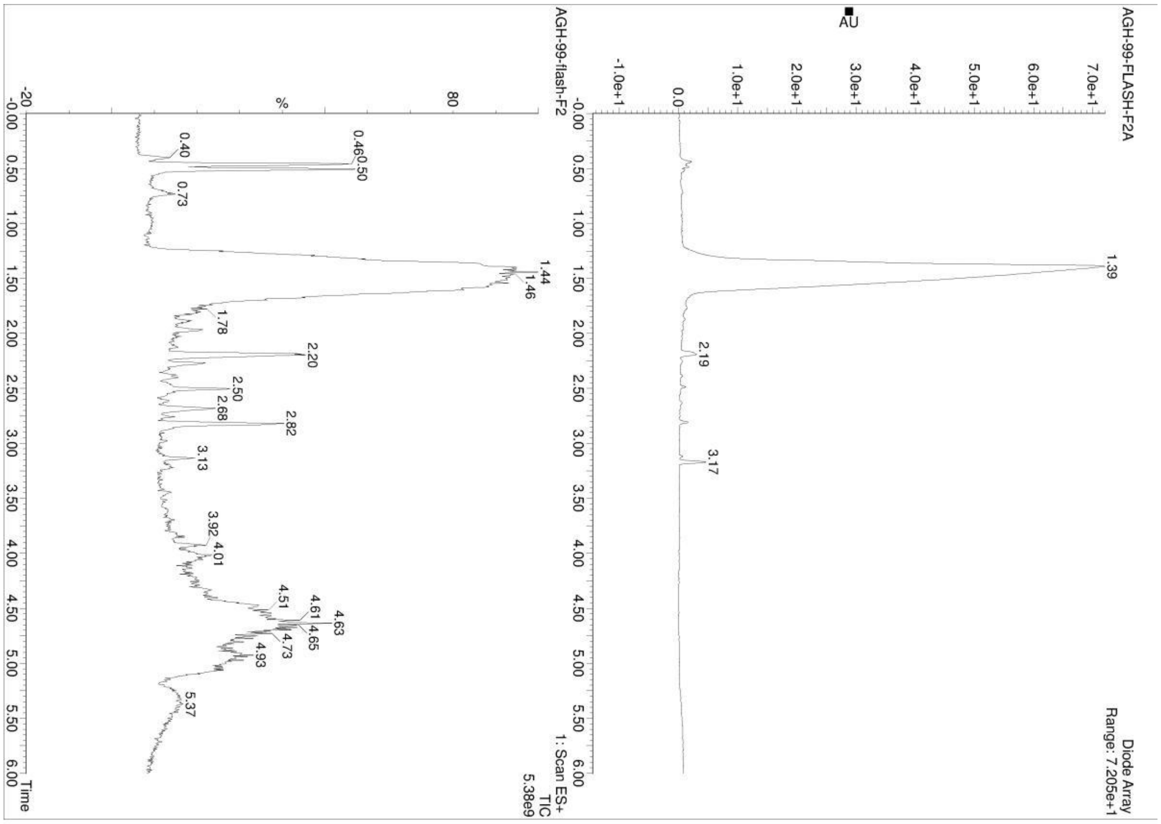
AGH-99-FLASH-F2A
Diode Array
Range: 7.205e+1
AU
1.39
2.19
3.17
AGH-99-flash-F2
1: Scan ES+
TIC
5.38e9
0.40
0.46
0.50
0.73
1.44
1.46
1.78
2.20
2.50
2.68
2.82
3.13
3.92
4.01
4.51
4.61
4.63
4.65
4.73
4.93
5.37
Time

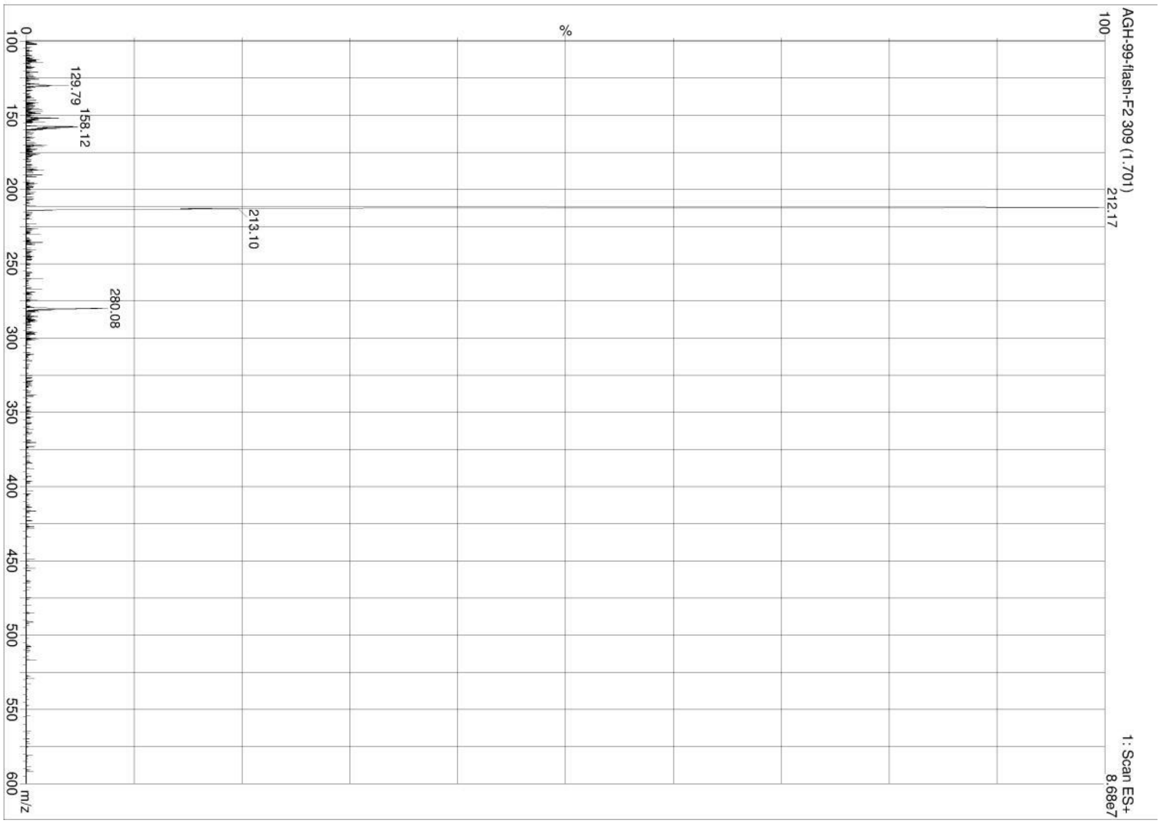
AGH-99-flash-F2 309 (1.701)
1: Scan ES+
8.68e7
212.17
213.10
129.79
158.12
280.08
m/z

(4) Tert-butyl-5-(benzyloxy)-3-(1-ethyl-1*H*-imidazol-5-yl)-1*H*-indole-1-carboxylate

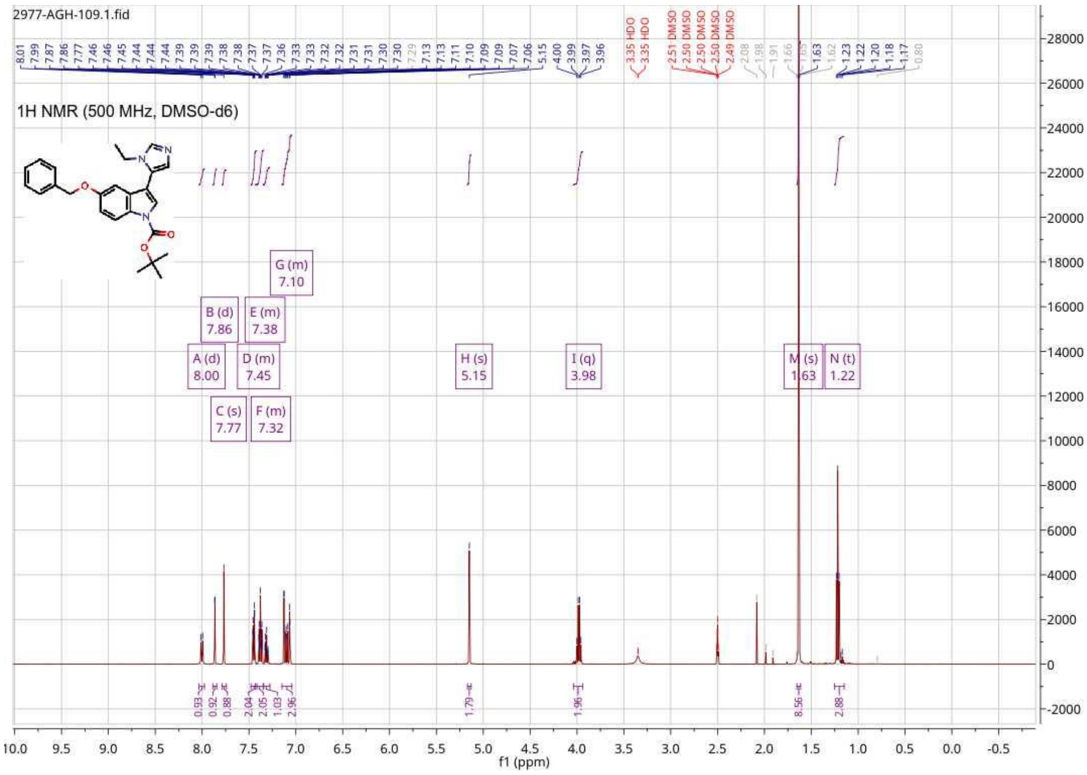

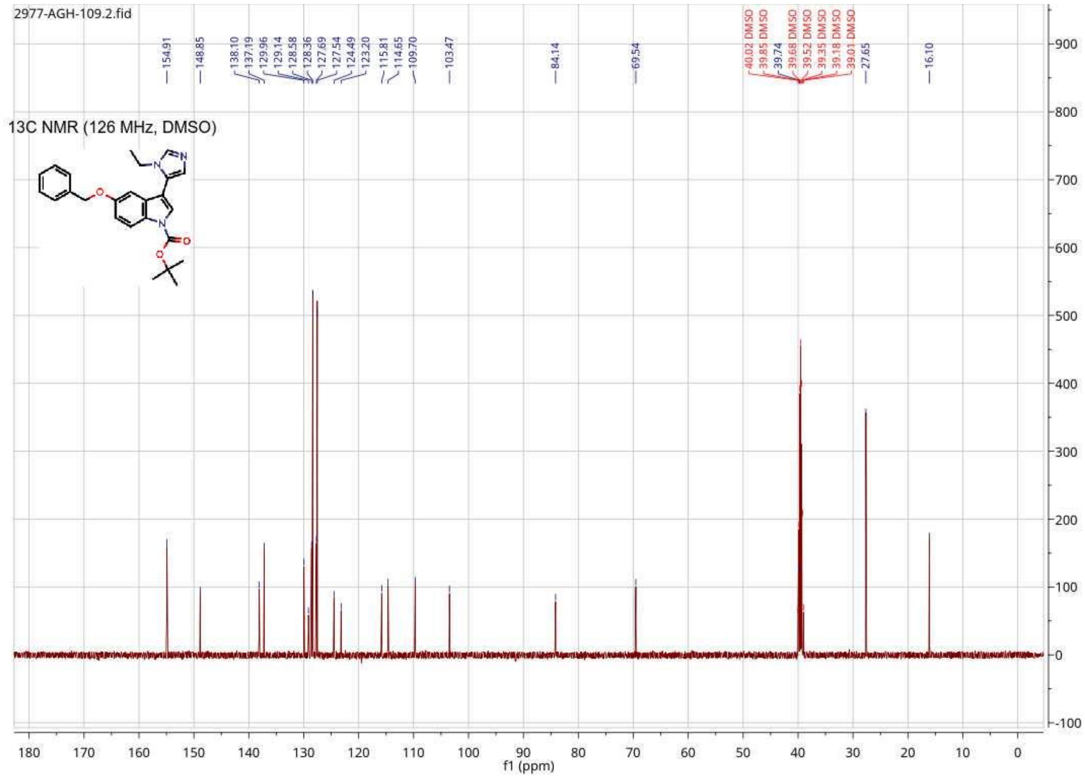

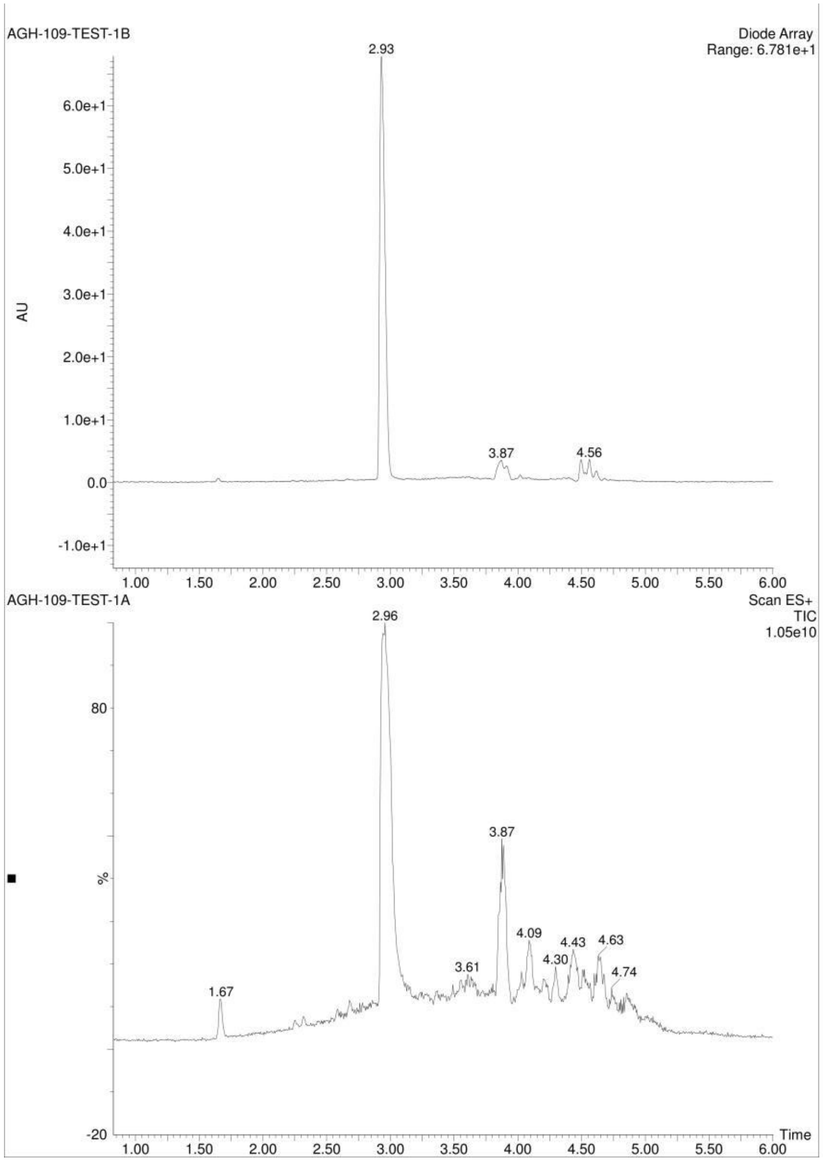
AGH-109-TEST-1B
Diode Array
Range: 6.781e+1
AU
2.93
3.87
4.56
AGH-109-TEST-1A
Scan ES+
TIC
1.05e10
%
1.67
2.96
3.61
3.87
4.09
4.30
4.43
4.63
4.74
Time

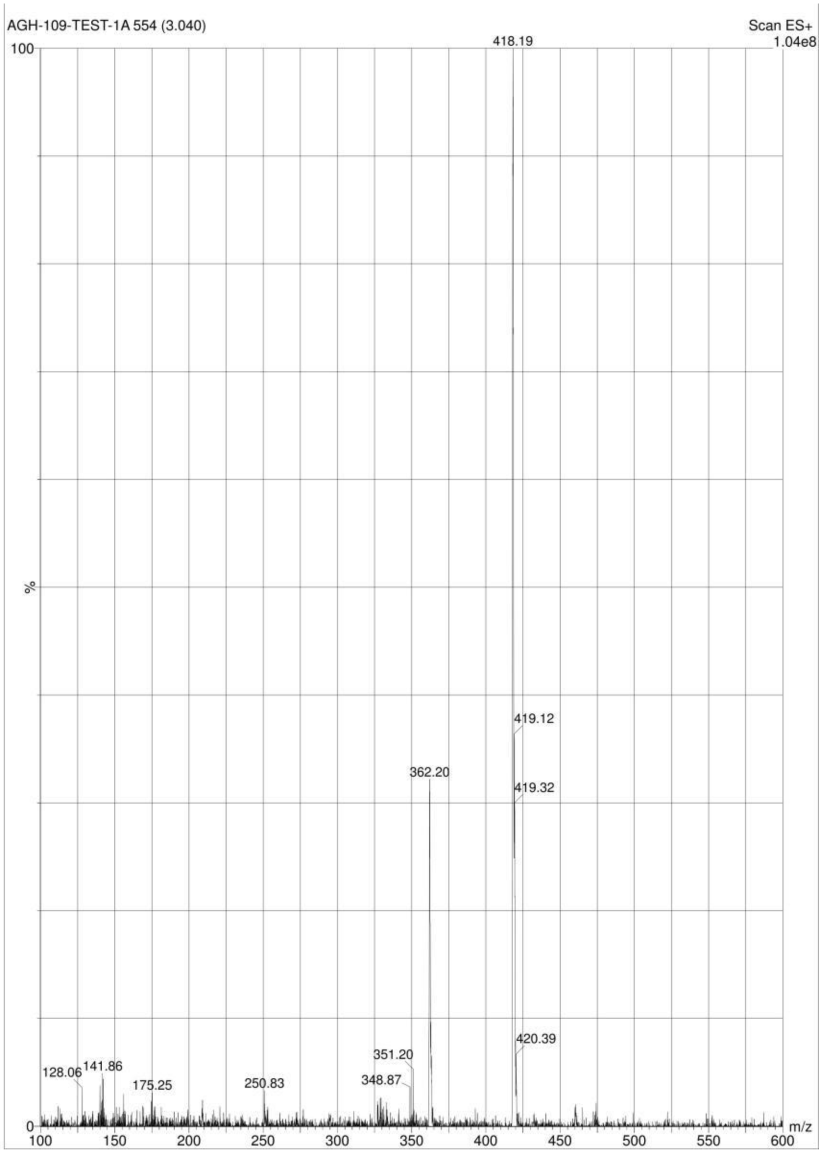
AGH-109-TEST-1A 554 (3.040)
Scan ES+
1.04e8
%
418.19
419.12
419.32
362.20
420.39
351.20
348.87
128.06
141.86
175.25
250.83
m/z

(5) Tert-butyl-3-(1-ethyl-1*H*-imidazol-5-yl)-5-hydroxy-1*H*-indole-1-carboxylate

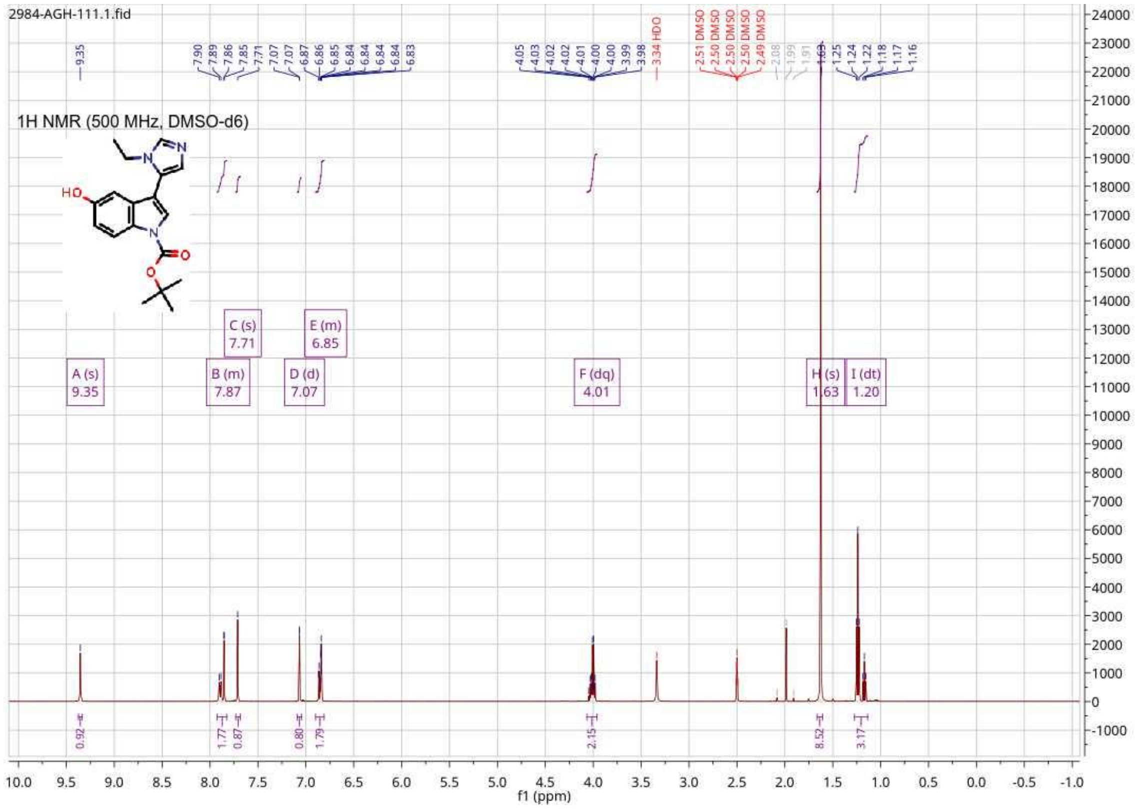

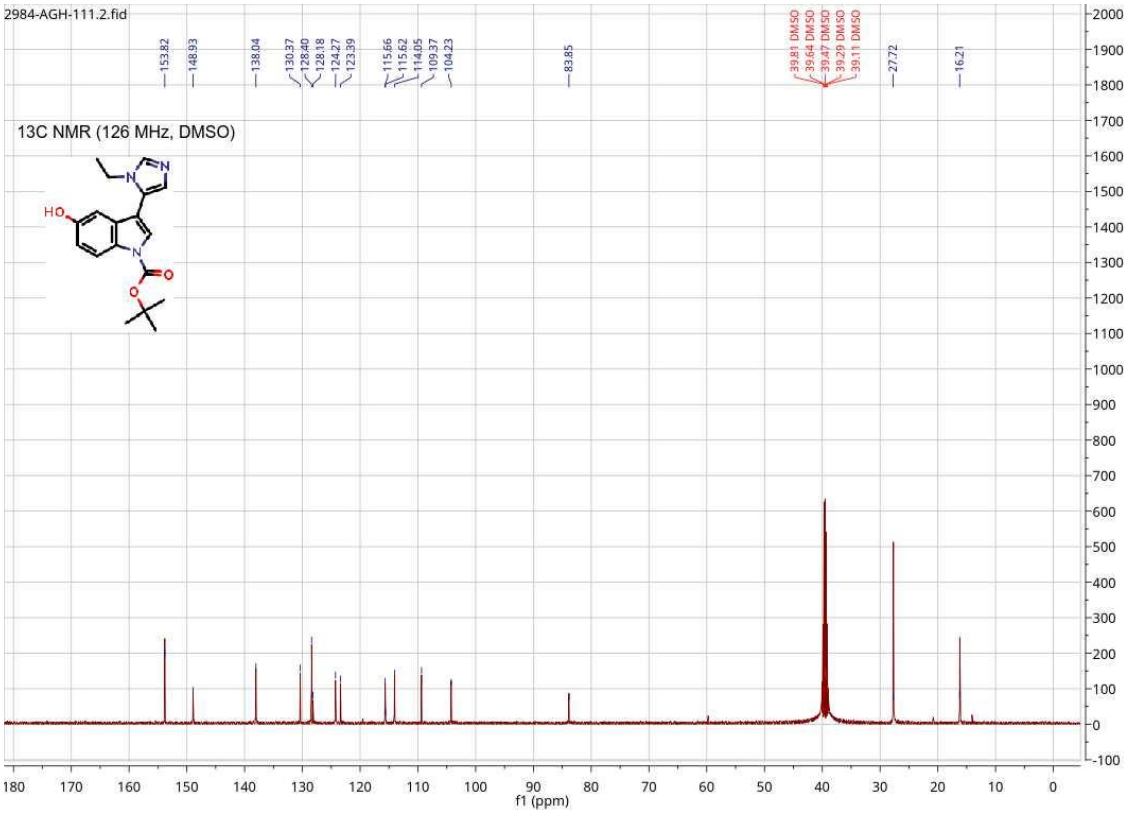

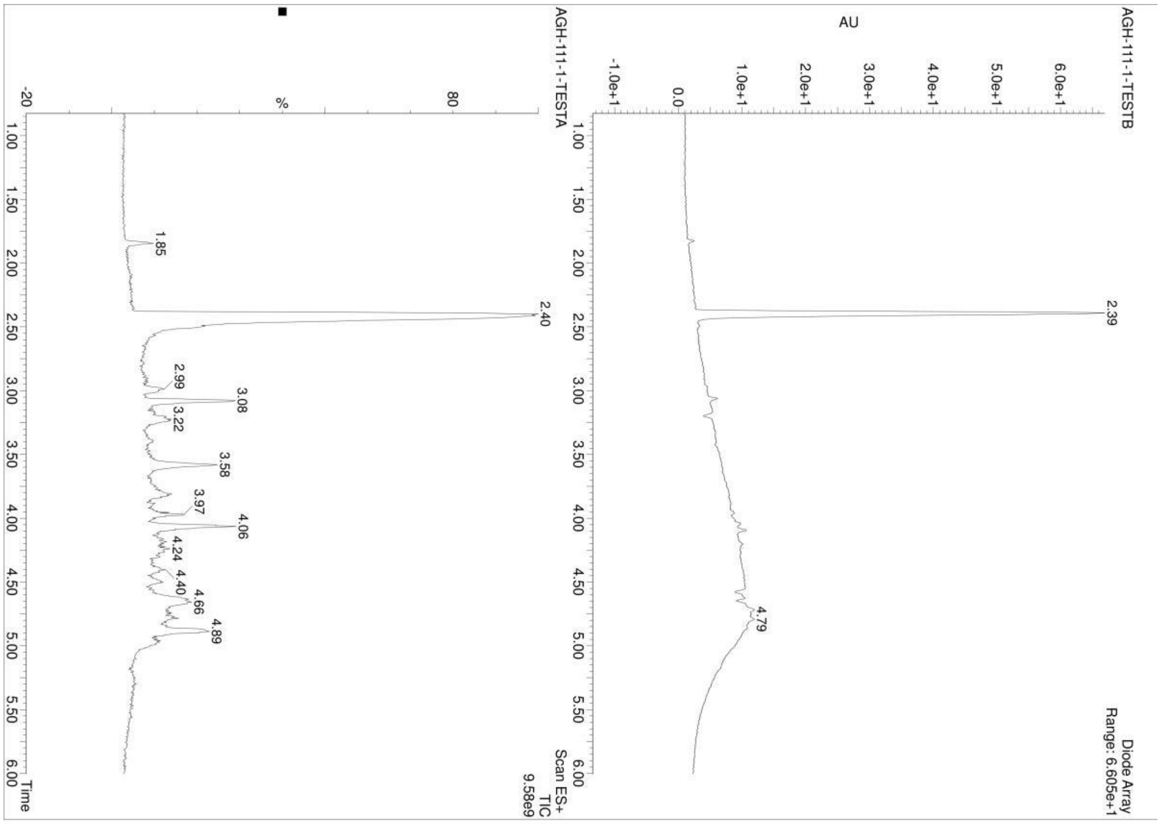
AGH-111-1-TESTB
Diode Array
Range: 6.605e+1
AU
2.39
4.79
AGH-111-1-TESTA
Scan ES+
TIC
9.58e9
%
1.85
2.40
2.99
3.08
3.22
3.58
3.97
4.06
4.24
4.40
4.66
4.89
Time

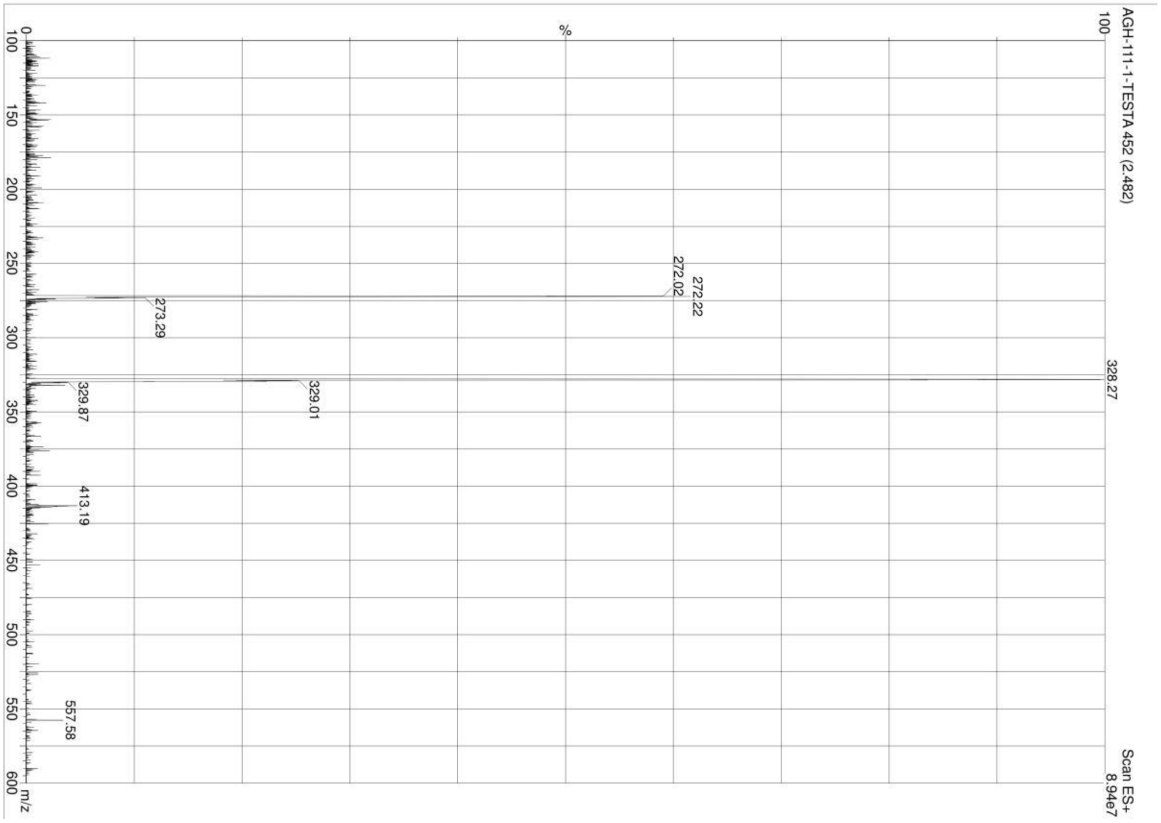
AGH-111-1-TESTA 452 (2.482)
Scan ES+
8.94e7
%
272.22
272.02
273.29
328.27
329.01
329.87
413.19
557.58
m/z
